# Synthesis and Optical Properties of Near-Infrared *meso*-Phenyl-Substituted Symmetric Heptamethine Cyanine Dyes

**DOI:** 10.3390/molecules23020226

**Published:** 2018-01-24

**Authors:** Andrew Levitz, Fahad Marmarchi, Maged Henary

**Affiliations:** 1 Department of Chemistry, Georgia State University, 50 Decatur St., Atlanta, GA 30303, USA; alevitz1@student.gsu.edu (A.L.); fjrjes1@student.gsu.edu (F.M.); 2 Center for Diagnostics and Therapeutics, Georgia State University, Petit Science Center, 100 Piedmont Ave SE, Atlanta, GA 30303, USA

**Keywords:** heptamethine cyanine dyes, absorbance, fluorescence, NIRF, physiochemical properties, HSA binding

## Abstract

Heptamethine cyanine dyes are a class of near infrared fluorescence (NIRF) probes of great interest in bioanalytical and imaging applications due to their modifiability, allowing them to be tailored for particular applications. Generally, modifications at the *meso*-position of these dyes are achieved through Suzuki-Miyaura C-C coupling and S_RN_1 nucleophilic substitution of the chlorine atom at the *meso*-position of the dye. Herein, a series of 15 *meso* phenyl-substituted heptamethine cyanines was synthesized utilizing a modified dianil linker. Their optical properties, including molar absorptivity, fluorescence, Stokes shift, and quantum yield were measured. The HSA binding affinities of two representative compounds were measured and compared to that of a series of trimethine cyanines previously synthesized by our lab. The results indicate that the binding of these compounds to HSA is not only dependent on hydrophobicity, but may also be dependent on steric interferences in the binding site and structural dynamics of the NIRF compounds.

## 1. Introduction

Heptamethine cyanine dyes are a class of near infared fluorescence (NIRF) probes that have shown great potential in numerous applications due to their versatility, low toxicity, narrow absorption band, and high extinction coefficients [1,2,3,4,5,6]. These dyes are comprised of two terminal nitrogen-containing heterocycles linked together by a conjugated polymethine chain. The heterocycles act as both electron donors and acceptors creating an electron deficient system throughout the molecule, allowing for long wavelength absorption [7,8,9,10]. Heptamethine cyanines have been used in medical imaging targeting of: cartilage, bone, endocrine gland, biomolecular labeling and much more all serving as contrast agents to aid in surgical application [5,11,12,13,14,15,16,17]. Modifications to the cyanine dye scaffold can alter optical properties, solubility, and allow for specific tailoring of dyes for their desired application.

A main contributing factor in the successful application of heptamethine cyanine dyes is their modifiability. Most commonly cyanines are modified by the use of different heterocycles and with different substituents on the nitrogen atom of the heterocycle. One point of modification that has not been thoroughly investigated is the central (*meso*) carbon of the methine bridge. Many derivatives described in literature have been made by replacing the chlorine atom at this position via a S_RN_1 mechanism [12,13,14]. The most common method of carbon-coupling at the *meso*-position thus far has been done by first synthesizing a heptamethine cyanine dye containing a chlorine atom at the *meso*-position followed by Suzuki-Miyaura coupling [18,19,20]. While this method is successful in synthesizing many scaffolds, it requires tedious purification and the use of an expensive palladium catalyst [12,13,14]. Although the phenyl-substituted dianil linker has been previously synthesized, it has not been thoroughly investigated for its effects on the optical properties on the NIRF dye [21,22].

Many of these cyanine dyes are administered via IV injection in which the dyes are transported to their target through the bloodstream [23]. Human serum albumin (HSA) is the most abundant protein in human blood plasma, serving an important role of transporting substances throughout the body [23,24,25]. It is synthesized in the liver and has great binding capacity for hydrophobic compounds [23,26]. HSA has been a widely studied protein because of its importance in drug delivery [15]. HSA contains four binding pockets and does not require biomolecular ligand specificity, increasing its versatility and usefulness in medical research [23,24,25,26]. It is well described in literature that HSA binds to hydrophobic entities. A unique attribute of HSA is that it forms reversible covalent bonds with the binding agent, this allows for stable complex formation; however, since the bonds are reversible also allows for localization and deposit [27].

Our lab has previously designed and synthesized a series of trimethine dyes and studied their hydrophobicity and its effect on their interactions with HSA [28]. In this paper, a series of heptamethine cyanines with varying degrees of hydrophobicity containing a *meso*-phenyl substituent were synthesized through the use of a phenyl-substituted dianil linker. This method not only allows for a more facile synthesis, but a wider array of dyes can be made and can serve for various applications. The effect of the phenyl ring on the dyes hydrophobicity, optical properties, and binding to HSA was studied and compared to the results from our previous study [28].

## 2. Results and Discussion

### 2.1. Synthesis

**Scheme 1:**
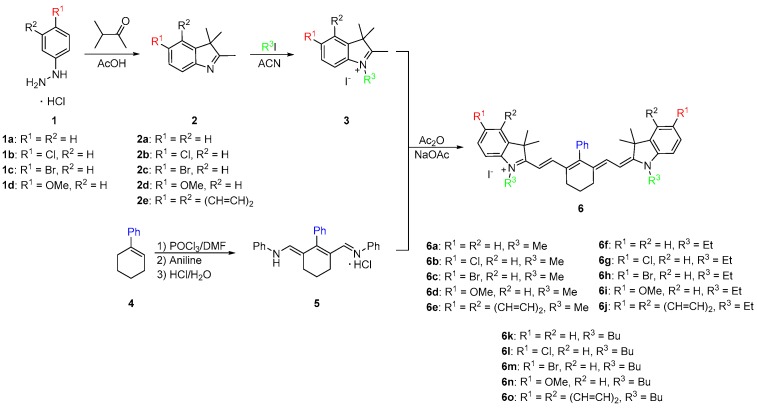
Synthetic routes of heptamethine dyes containing a phenyl ring at the *meso*-position.

As shown in Scheme 1, the synthesis began with a Fischer indole cyclization by refluxing 4-substituted phenylhydrazines **1** overnight with 3-methyl-2-butanone in glacial acetic acid. After cooling to room temperature, the reaction mixture was neutralized and the substituted indoles **2** were extracted with dichloromethane to give brown oils. The oils were dissolved in acetonitrile and refluxed overnight with various alkyl halides to yield quaternary ammonium salts **3**. In parallel to salt formation, a phenyl-substituted dianil compound was synthesized through a Vilsmeier Haack formylation with 1-phenylcyclohexene (**4**). The ends of the dianil linker were capped with aniline for stability to yield dianil compound **5** [21,22]. Various quaternary ammonium salts **3** and dianil compound **5** are then condensed in a 2:1 ratio in acetic anhydride to yield the final phenyl substituted heptamethine cyanines **6**. Pure compounds were obtained in good yield by simply washing with methanol.

The synthetic route described in Scheme 1 provided a new carbon-carbon linked substituent position at the *meso* center adding to the versatility of heptamethine cyanine. The phenyl group was added before the dye was made. This allowed for an efficient method of preparation of the dye, which required no catalysts or complex purification methods and allowed for a wider array hydrophobic compounds to be made.

**Figure 1:**
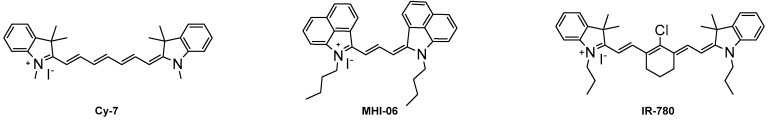
The structures of the three NIR standards used for the study.

Once the dyes have been successfully synthesized, the optical properties were measured, and representative dyes **6a** and **6k** were studied for their binding affinity to HSA. The optical properties were compared to commercially available heptamethine dyes **Cy-7** and **IR-780** due to the similar absorbance and emission wavelengths. The binding studies of compounds **6a** and **6k** were compared to **MHI-06**, a dye previously reported as a strong HSA binding agent [28]. Figure 1 shows the three dye structures of the standards used in our study.

### 2.2. Optical Properties

As described in Scheme 1, fifteen final NIRF contrast agents were synthesized using the dianil linker to yield symmetrical heptamethine cyanines **6a**–**o**. The compounds are broken down to three sets of five. Dyes **6a**–**e** all contain a methyl substituent off the nitrogen of the heterocycle with varying substitutions at the six position of the heterocyclic ring.

Dyes **6f**–**j** and **6k**–**o** contain ethyl and butyl *N*-alkyl substituents, respectively. In comparison to **Cy-7**, the optical properties of the new compounds were found to be superior (Table 1). The addition of the cyclohexene ring provided rigidity to the compounds, increasing the molar absorptivity and quantum yield by 60,000 M^−1^·cm^−1^ and 5%, respectively, for **6a** [29]. To determine the effects the phenyl ring had on the optical properties, the dyes were compared to **IR-780** [30]. Although the molar absorptivity of the studied dyes were within the same range as the commercially available dye, the quantum yield was dramatically increased with the introduction of the electron rich phenyl ring, observing a 23%–47% increase in quantum yield. The chlorine atom at the *meso*-position of dye **IR-780** promotes intersystem crossing due to the heavy atom effect, and allows for the molecule to relax in non-radiative means and decreases the fluorescence [31].

**Figure 2:**
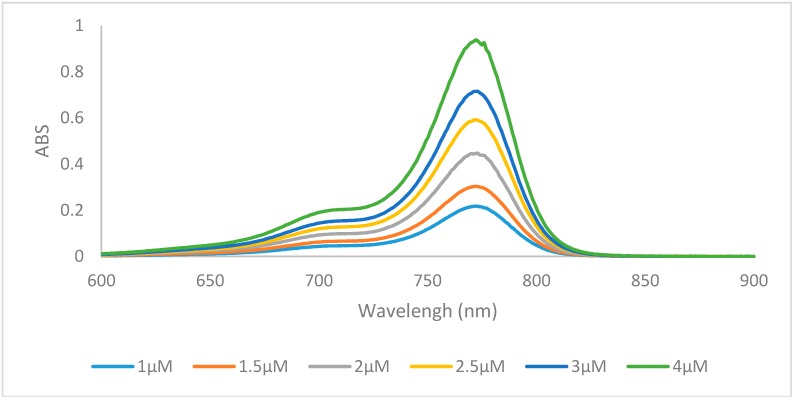
Absorption spectra of dye **6m** in ethanol.

Increasing the length of *N*-alkyl substituents from a methyl to ethyl did not result in any significant changes in optical properties, but an increase in size to the butyl group generally lowered the molar absorptivity. Dyes containing hydrogen **6a**, **6f**, **6k**, and halogens **6b**, **6c**, **6g**, **6h**, **6l**, **6m** at the **6** position of the heterocycle displayed absorption λ_max_ values of 759–773 nm with redshifts from the hydrogen to the halogens from 6 nm to 10 nm which has previously been described [32]. Absorption spectra of representative compound **6m** was shown in Figure 2. All 15 compounds have Stokes shifts ranging from 12 to 21 with the benz[*e*]indolenine containing compounds **6e**, **6j**, **6o** having the shortest Stokes shifts. All three methoxy-substituted compounds **6d**, **6i**, **6n** had redshifted absorption λ_max_ values from 16 nm to 26 nm and lower quantum yields than the other compounds while dyes **6e**, **6j**, **6o** were redshifted 37–39 nm around 800 nm due to the increased conjugation of the benz[*e*]indolenine heterocycle.

**Figure 3:**
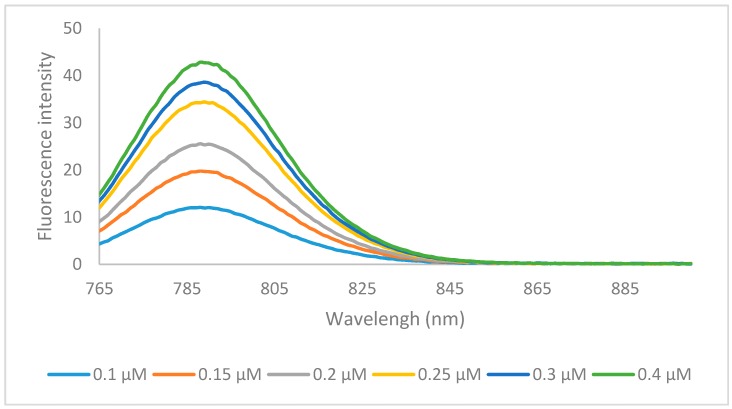
Emission spectra of dye **6m** in ethanol with excitation wavelength of 750 nm.

The emission data of all the dyes followed the same trends, whereby compounds containing the hydrogen **6a**, **6f**, **6k**, and halogen **6b**, **6c**, **6g**, **6h**, **6l**, **6m** substituents had the highest quantum yields, and the longer *N-*alkyl chains increased the quantum yield by 4%–8% from methyl to ethyl and 5%–7% from ethyl to butyl. Emission spectra of representative compound **6m** was shown in Figure 3. There was no significant difference in quantum yield between the hydrogen and halogens within the same set. Compounds **6e**, **6j**, **6o** containing the benz[*e*]indolenine heterocycle and **6d**, **6i**, **6n** containing the methoxy substituent displayed lower quantum yields at 16%–17% and 10%–12%, respectively, which is consistent with previous reports [12].

Although molar absorptivity and quantum yield are important properties of fluorophores, in regards to application, the molecular brightness gives a more useful indication of the dye utility. Molecular brightness takes into account both molar absorptivity and quantum yield [33,34]. Dyes that have high quantum yield, but do not absorb light efficiently (low molar absorptivity) are still not emitting as many photons and are less useful for fluorescent applications. *N*-alkyl substituents from a methyl to ethyl increased the molecular brightness by approximately 20,000 M^−1^·cm^−1^ for the hydrogen-**6a**, **6f**, **6k**, and halogen-**6b**, **6c**, **6g**, **6h**, **6l**, **6m** substituted compounds while the butyl compounds showed lower molecular brightness. Due to the low quantum yield of the benz[*e*]indolenine heterocycle **6e**, **6j**, **6o** and the methoxy **6d**, **6i**, **6n** substituted compounds, the two sets showed the lowest molecular brightness of 38,000–40,000 M^−1^·cm^−1^ and 17,000–20,000 M^−1^·cm^−1^, respectively.

### 2.3. Physiochemical Properties

In our previous study of trimethine cyanine dyes it was shown that **MHI-06** (Figure 1) bound HSA with an affinity of 1.0 × 10^6^ M^−1^ [28]. In that study of the binding affinity of trimethine cyanines, a trend was observed correlating hydrophobicity to the binding affinity. As the dyes became more hydrophobic greater binding affinity was observed. However, the correlation did not hold when the large *N*-phenylpropyl side chain was introduced in the trimethine series [28]. It was hypothesized that the binding affinity decreased due to the increased size of the *N*-phenylpropyl side chain hindering the dye from entering the HSA binding pocket. The heptamethine cyanines **6a**–**o** synthesized in this work were tailored to be hydrophobic in order to observe if binding increases due to increase of hydrophobicity or decreases due to the size and steric hindrance.

The physicochemical properties were calculated using ChemAxon for the 15 heptamethine dyes synthesized and compared to our pervious reported compound **MHI-06** (Table 2) [28]. Physicochemical trends were observed in each series with the same heterocyclic substituents as well as with the same *N*-alkyl side chain. Compounds **6d**, **6i**, **6n** with the methoxy substituent showed the lowest log*D*, due to its ability to form hydrogen bonds. Slightly higher values were observed for compounds with the hydrogen-substitued **6a**, **6f**, **6k**. A 1.2 and 1.5 increase of log*D* was observed from the hydrogen to the chloro-and bromo-substituted compounds, respectively. As expected, the series with the benz[*e*]indolenine heterocycle **6e**, **6j**, **6o** had the highest log*D* values due to the addition of another phenyl ring. All heptamethine cyanines **6a**–**o** had significantly higher log*D* values compared to that of **MHI-06** due to the presence of the phenyl substituent at the *meso*-position and the increased size of the hydrocarbon skeleton. The dipole moments decreased as the length of the alkyl chain increased from methyl to butyl, with methoxy-substituted dyes having significantly higher values. In comparison to **MHI-06** most of the compounds, especially that of the methyl series **6a**–**e**, had a higher dipole moment, but for **6k** which had fairly similar results to **MHI-06** at 2.85 and 2.48, respectively. The number of rotatable bonds increased by 2 from methyl to ethyl and by 4 from ethyl to butyl. Only the methoxy heterocyclic substitution affected the number of rotatable bonds with an additional rotatable bond for each methoxy in compounds **6d**, **6i**, **6n**. **MHI-06** had eight rotatable bonds. All of the dyes with the exception of those containing the methoxy substituent had a total polar surface area (TPSA) of 6.25, 0 hydrogen bond donors, and 1 hydrogen bond acceptor (ChemAxon). Dyes **6d**, **6i**, **6n** containing the methoxy substituent had higher TPSA at 24.71 and three hydrogen bond acceptors. The presence of the polar oxygen increases the TPSA, and increases the number of hydrogen bond that can form by two.

**Figure 4:**
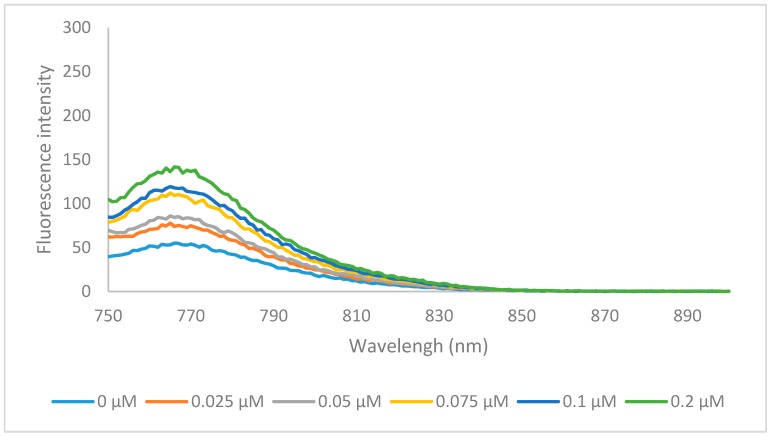
The emission specra of **6a** (0.2 μM) binding with various concentration of HSA, in PBS buffer at excitation wavelength of 740 nm.

In summary, the newly synthesized heptamethine dyes **6a**–**o** were significantly more hydrophobic then **MHI-06**, but had a much larger volume. Although hydrophobicity plays a key role in binding to HSA, reversibly the size of the compound could inhibit the ability of its binding to HSA pocket. Compounds **6a** and **6k** were tested for their ability to bind to HSA, to further determine if other factors than hydrophobicity, play an important role in HSA binding allowing a better understanding of binding nature of the larger heptamethine cyanines to HSA. The HSA binding spectra for **6a** is shown below (Figure 4) and binding data of **6k** is shown in the supplemental information (**6k** HSA binding).

### 2.4. HSA Binding

The formation of a dye/substrate conjugate was studied with HSA in phosphate buffered saline (PBS), pH 7.4. Previous research by Kim et al. suggested that cyanine dyes bind HSA in with a 1:1 stoichiometry which was confirmed by our and other research groups using trimethine cyanine dyes [3,28,35,36,37]. The binding interactions were studied by measuring the changes in emission intensities at a fixed concentration of dye with varying micromolar concentrations of HSA and using a double reciprocal plot of [HSA]^−1^ vs. **Δ**F^−1^, where **Δ**F is the change in emission intensity of the Dye/HSA conjugate, that should give a linear relationship. The binding affinity is then calculated by dividing the intercept by the slope of the line. Our lab has previously shown that both the *N*-alkyl substituents and the heterocyclic ring of the cyanines have profound effects on overall conjugation with the biomolecule [20]. It has also previously been shown that cyanine dyes aggregate in polar solvents such as PBS buffer due to strong intermolecular van der Waals interactions between the heterocycles that cause the dyes to form H-aggregates [38]. Generally, organic solvents are used to disrupt this aggregate formation, but organic solvents cannot be used in the presence of HSA due to their ability to denature the biomolecule. Conjugate formation with HSA disrupts the aggregation increasing monomer formation and thereby increasing fluorescence emission of the dyes. It was determined that heptamethine dye **6a** binds HSA with an affinity of 8 × 10^1^ M^−1^. This is 5 orders of magnitude lower than the previously tested trimethine cyanines, **MHI-06**, which bound on the order of 1 × 10^6^ M^−1^ [28]. This further confirms our previous hypothesis that the binding affinity of the dyes is not only hydrophobicity dependent, but dependent on steric interferences in the HSA binding site. It also confirms that the delocalized cationic nature of the dyes may has no electrostatic interference with the binding cavity as this heptamethine cyanine displays increased delocalization over the previously tested trimethine cyanines. Albumin is known to bind a variety of compounds including fatty acids, nucleic acids, and oligoproteins therefore this information on the steric specificity of these binding sites is of potential interest when developing methods to study them [28].

## 3. Experimental Section

### 3.1. General Information

All chemicals and solvents were of American Chemical Society grade or HPLC purity and were used as received. HPLC grade ethanol were purchased from Sigma-Aldrich (St. Louis, MO, USA). All other chemicals were purchased from Fisher Scientific (Pittsburgh, PA, USA) or Acros Organics (Pittsburgh, PA, USA). The reactions were followed using silica gel 60 F254 thin layer chromatography plates (Merck EMD Millipore, Darmstadt, Germany). The ^1^H NMR and ^13^C NMR spectra were obtained using high quality Kontes NMR tubes (Kimble Chase, Vineland, NJ, USA) rated to 500 MHz and were recorded on an Avance spectrometer (Bruker, Billerica, MA; 400 MHz for ^1^H and 100 MHz for ^13^C) in DMSO-*d*_6_, acetone-*d*_6_ CD_3_Cl-*d*_3_. High-resolution accurate mass spectra (HRMS) were obtained at the Georgia State University Mass Spectrometry Facility using a Q-TOF micro (ESI-Q-TOF) mass spectrometer (Waters, Milford, MA, USA). All compounds tested were >95% pure.

A solution of POCl_3_ (11 mL, 117.66 mmol) in dichloromethane (10 mL) was added dropwise to a solution of DMF (13 mL, 167.89 mmol) in dichloromethane (13 mL) at 0 °C for 30 min under inert conditions. Then 1-phenylcyclohexene (**4**, 5.5 mL, 32.81 mmol) was dissolved in dry dichloromethane (5 mL) and added dropwise to the solution which was then refluxed for 3 h. The solution was allowed to cool to room temperature and then poured over 500 mL of ice/water. Aniline (9 mL, 98.57 mmol) in ethanol (9 mL) was added to cap the ends. The crude solid was collected and washed with diethyl ether and hexanes. Resulting in the dianil linker **5** as a pure compound and used without further purification.

In parallel, substituted hydrazines **1** (4.0 g, 22.25 mmol) were reacted with 3-methylbutanone (3 mL, 28.04 mmol) in acetic acid and heated to a 100 °C for 24 h. The solution was then neutralized using sodium bicarbonate and extracted using dichloromethane; affording substituted indolenine heterocycles **2** which was dried under reduced pressure. The heterocycles **2** were then reacted with an alkyl halide in acetonitrile at 100 °C for 12–18 h. The quaternary ammonium salts **3** were precipitated with diethyl ether, and collected.

The various salts **3** (2 molar eq), the dianil linker **5** (1 molar eq), and sodium acetate (2 molar eq) were dissolved in acetic anhydride and heated to 60 °C for 2–3 h. The crude product was then precipitated with diethyl ether, collected, and washed with methanol to yield heptamethine dyes **6** as pure sample.

### 3.2. Characterization

*1,3,3-Trimethyl-2-((E)-2-((E)-6-(2-((E)-1,3,3-trimethylindolin-2-ylidene)ethylidene)-3,4,5,6-tetrahydro-[1,1′-biphenyl]-2-yl)vinyl)-3H-indol-1-ium iodide*, **6a**: Yield 77%; m.p. > 260 °C; ^1^H NMR (CDCl_3_) **δ** 1.11 (s, 12H), 1.95 (m, 2H), 2.68 (t, *J* = 6.0 Hz, 4H), 3.58 (s, 6H), 6.17 (d, *J* = 14.0 Hz, 2H), 7.16 (m, 4H), 7.25 (d, *J* = 8.4 Hz, 2H), 7.34 (m, 4H), 7.46 (d, *J* = 7.6 Hz, 2H), 7.62 (m, 3H). ^13^C NMR (CDCl_3_) **δ** 21.3, 24.5, 27.3, 31.5, 48.5, 100.7, 111.3, 122.7, 124.9, 128.5, 128.8, 129.0, 129.6, 131.0, 139.1, 140.9, 143.3, 147.3, 161.5, 172.1. HRMS (ESI) *m*/*z*: calcd. for C_38_H_41_N_2_^+^ 525.3264, obsd 525.3241.

*5-Chloro-2-((E)-2-((E)-6-(2-((E)-5-chloro-1,3,3-trimethylindolin-2-ylidene)ethylidene)-3,4,5,6-tetrahydro-[1,1′-biphenyl]-2-yl)vinyl)-1,3,3-trimethyl-3H-indol-1-ium iodide*, **6b**: Yield 78%; m.p. > 260 °C; ^1^H NMR (CDCl_3_) **δ** 1.17 (s, 12H), 2.07 (m, 2H), 2.74 (t, *J* = 6.4 Hz, 4H), 3.65 (s, 6H), 6.12 (d, *J* = 14.0 Hz, 2H), 7.05 (d, *J* = 8.4 Hz, 2H), 7.17 (m, 6H), 7.30 (t, *J* = 8.4 Hz, 2H), 7.56 (m, 3H); ^13^C NMR (CDCl_3_) **δ** 21.1, 25.0, 27.5, 32.5, 48.5, 100.8, 111.3, 122.5, 128.2, 128.5, 128.7, 129.4, 130.2, 133.0, 138.7, 141.5, 142.1, 148.2, 163.0, 171.4. HRMS (ESI) *m*/*z*: calcd. for C_38_H_39_Cl_2_N_2_^+^ 593.2485, obsd 593.2475.

*5-Bromo-2-((E)-2-((E)-6-(2-((E)-5-bromo-1,3,3-trimethylindolin-2-ylidene)ethylidene)-3,4,5,6-tetrahydro-[1,1′-biphenyl]-2-yl)vinyl)-1,3,3-trimethyl-3H-indol-1-ium iodide*, **6c**: Yield 75%; m.p. > 260 °C; ^1^H NMR (CDCl_3_) **δ** 1.12 (s, 12H), 2.08 (m, 2H), 2.76 (t, *J* = 6.4 Hz, 4H), 3.66 (s, 6H), 6.15 (d, *J* = 14.0 Hz, 2H), 6.99 (d, *J* = 8.4 Hz, 2H), 7.15 (d, *J* = 14.0 Hz, 2H), 7.21 (dd, *J* = 7.6 Hz, 2H), 7.46 (dd, *J* = 8.4 Hz, 2H), 7.56 (m, 3H). ^13^C NMR (CDCl_3_) **δ** 21.0, 25.0, 27.5, 32.5, 48.4, 100.9, 111.7, 117.7, 125.3, 128.2, 128.5, 129.4, 131.5, 133.2, 138.7, 142.0, 142.4, 148.1, 162.9, 171.2. HRMS (ESI) *m*/*z*: calcd. for C_38_H_39_Br_2_N_2_^+^ 681.1475, obsd 681.1475.

*5-Methoxy-2-((E)-2-((E)-6-(2-((E)-5-methoxy-1,3,3-trimethylindolin-2-ylidene)ethylidene)-3,4,5,6-tetrahydro-[1,1′-biphenyl]-2-yl)vinyl)-1,3,3-trimethyl-3H-indol-1-ium iodide*, **6d**: Yield 73%; m.p. 238–240 °C; ^1^H NMR (CDCl_3_) **δ** 1.17 (s, 12H), 2.06 (m, 2H), 2.70 (t, *J* = 6.4 Hz, 4H), 3.61 (s, 6H), 3.82 (s, 6H), 6.03 (d, *J* = 14.0 Hz, 2H), 6.76 (s, 2H), 6.86 (d, *J* = 8.8 Hz, 2H), 7.04 (d, *J* = 8.4 Hz, 2H), 7.10 (d, *J* = 14.0 Hz, 2H), 7.21 (d, *J* = 6.8 Hz, 2H), 7.55 (m, 3H); ^13^C NMR (CDCl_3_) **δ** 21.2, 24.9, 27.5, 32.2, 48.5, 55.9, 99.8, 109.1, 110.9, 112.8, 128.0, 128.4, 129.5, 131.4, 136.5, 139.1, 142.2, 146.8, 157.8, 161.3, 171.0. HRMS (ESI) *m*/*z*: calcd. for C_40_H_75_N_2_O_2_^+^ 585.3476, obsd 585.3469.

*1,1,3-Trimethyl-2-((E)-2-((E)-6-((E)-2-(1,1,3-trimethyl-1,3-dihydro-2H-benzo[e]indol-2-ylidene)ethylidene)-3,4,5,6-tetrahydro-[1,1′-biphenyl]-2-yl)vinyl)-1H-benzo[e]indol-3-ium iodide*, **6e**: Yield 63%; m.p. > 260 °C; ^1^H NMR (CDCl_3_) **δ** 1.50 (s, 12H), 2.11 (m, 2H), 2.79 (t, *J* = 6.4 Hz, 4H), 3.77 (s, 6H), 6.16 (d, *J* = 14.0 Hz, 2H), 7.30 (m, 2H), 7.44 (m, 4H), 7.55 (t, *J* = 7.6 Hz, 2H), 7.66 (m, 3H), 7.92 (m, 6H); ^13^C NMR (CDCl_3_) **δ** 21.2, 25.0, 27.1, 32.5, 50.2, 99.9, 110.5, 121.9, 124.7, 127.5, 128.0, 128.3, 128.6, 129.5, 130.1, 130.5, 131.7, 132.2, 132.9, 139.0, 140.2, 147.1, 162.0, 173.3. HRMS (ESI) *m*/*z*: calcd. for C_46_H_45_N_2_^+^ 625.3577, obsd 625.3570.

*1-Ethyl-2-((E)-2-((E)-6-(2-((E)-1-ethyl-3,3-dimethylindolin-2-ylidene)ethylidene)-3,4,5,6-tetrahydro-[1,1′-biphenyl]-2-yl)vinyl)-3,3-dimethyl-3H-indol-1-ium iodide*, **6f**; Yield: 70%; m.p. > 260 °C; ^1^H NMR (acetone-d_6_) **δ** 1.11 (s, 12H), 1.24 (t, *J* = 7.2 Hz, 6H), 1.96 (m, 2H), 2.97 (t, 4H), 4.14 ( m, 4H) 6.20 (d, *J* = 14 Hz, 2H), 7.18 (m, 4H), 7.35 (m, 4H), 7.47 (d, *J* = 7.6 Hz, 2H), 7.61 (m, 3H); ^13^C NMR (acetone-d_6_) **δ** 12.5, 21.3, 24.6, 27.4, 39.8, 40.0, 40.2, 48.7, 100.2, 111.2, 123.0, 125.0, 128.6, 129.0, 129.1, 129.6, 131.1, 141.1, 142.1, 147.7, 171.2. HRMS (ESI) *m*/*z*: calcd. for C_40_H_45_N_2_^+^ 553.3577, obsd 553.1566.

*5-Chloro-2-((E)-2-((E)-6-(2-((E)-5-chloro-1-ethyl-3,3-dimethylindolin-2-ylidene)ethylidene)-3,4,5,6-tetrahydro-[1,1′-biphenyl]-2-yl)vinyl)-1-ethyl-3,3-dimethyl-3H-indol-1-ium iodide*, **6g**; Yield 64%; m.p. > 260 °C; ^1^H NMR (CDCl_3_) **δ** 1.16 (s, 12H), 1.38 (t, *J* = 7.2 Hz, 6H), 2.07 (t, *J* = 5.6, 2H), 2.73 (t, *J* = 6.0 Hz, 4H), 4.142 (m, 4H), 6.10 (d, *J* = 14.0 Hz, 2H), 7.07 (d, *J* = 8.4 Hz, 2H), 7.28 (m, 8H) 7.59 (m, 3H); ^13^C NMR (CDCl_3_) **δ** 12.2, 21.1, 25.0, 27.5, 40.7, 48.6, 100.1, 111.4, 122.6, 128.3, 128.7, 128.8, 129.4, 130.3, 132.6, 138.7, 140.5, 142.4, 148.3, 163.1, 170.5. HRMS (ESI) *m*/*z*: calcd. for C_40_H_43_Cl_2_N_2_^+^ 621.2798, obsd 621.2788.

*5-Bromo-2-((E)-2-((E)-6-(2-((E)-5-chloro-1-ethyl-3,3-dimethylindolin-2-ylidene)ethylidene)-3,4,5,6-tetrahydro-[1,1′-biphenyl]-2-yl)vinyl)-1-ethyl-3,3-dimethyl-3H-indol-1-ium iodide*, **6h**; Yield 71%; m.p. > 260 °C; ^1^H NMR (CDCl_3_) **δ** 1.18 (s, 12H), 1.41 (t, *J* = 7.2 Hz, 6H), 2.10 (t, *J* = 6.4 Hz, 2H), 2.75 (t, 5.6 Hz, 4H), 4.16 (m, 4H), 6.13 (d, *J* = 14.4 Hz, 2H), 7.22 (m, 10H), 7.59 (m, 3H); ^13^C NMR (CDCl_3_) **δ** 12.2, 21.1, 25.1, 27.5, 40.1, 48.6, 100.3, 111.7, 117.7, 125.5, 128.3, 128.6, 129.4, 131.6, 133.0, 138.8, 141.0, 142.7, 148.2, 162.9, 170.3. HRMS (ESI) *m*/*z*: calcd. for C_40_H_43_Br_2_N_2_^+^ 709.1788, obsd 709.1780.

*1-Ethyl-2-((E)-2-((E)-6-(2-((E)-1-ethyl-5-methoxy-3,3-dimethylindolin-2-ylidene)ethylidene)-3,4,5,6-tetrahydro-[1,1′-biphenyl]-2-yl)vinyl)-5-methoxy-3,3-dimethyl-3H-indol-1-iumiodide*, **6i**; Yield 61%; m.p. > 260 °C; ^1^H NMR (acetone-d_6_) **δ** 1.22 (s; 12H), 1.34 (t, *J* = 7.2 Hz, 6H), 2.02 (m, 2H), 2.73 (t, *J* = 6.0 Hz, 4H), 3.84 (s, 6H), 4.21 (m, 4H), 6.23 (d, *J* = 14 Hz, 2H), 6.23 (d, *J* = 2.8 Hz, 2H) 6.94 (d, *J* = 2.4 Hz 2H) 7.22 (s, 2H) 7.28 (m, 6H) 7.66 (m, 3H); ^13^C NMR (acetone-d_6_) **δ** 11.6, 21.3 24.5, 26.8, 39.0, 48.8, 55.4, 99.1, 109.0, 111.2, 113.4, 128.0, 128.7, 129.5, 130.3, 135.5, 139.4, 142.8, 145.0, 158.1, 161.0, 170.6. HRMS (ESI) *m*/*z*: calcd. for C_42_H_49_N_2_O_2_^+^ 613.3789, obsd 613.3777.

*3-Ethyl-2-((E)-2-((E)-6-((E)-2-(3-ethyl-1,1-dimethyl-1,3-dihydro-2H-benzo[e]indol-2-ylidene)ethylidene)-3,4,5,6-tetrahydro-[1,1′-biphenyl]-2-yl)vinyl)-1,1-dimethyl-1H-benzo[e]indol-3-ium iodide*, **6j**; Yield 65%; m.p. > 260 °C; ^1^H NMR (CDCl_3_) **δ** 1.46 (t, *J* = 6.8 Hz, 6H), 1.51 (s, 12H), 2.13 (t, *J* = 5.6 Hz, 2H), 2.79 (t, *J* = 6.0 Hz, 4H), 4.28 (m, 4H), 6.17 (d, *J* = 14.0 Hz, 2H), 7.45 (m, 13H), 7.94 (m, 6H); ^13^C NMR (CDCl_3_) **δ** 99.3, 110.4, 122.0, 124.8, 127.6, 128.1, 128.3, 128.7, 129.5, 130.1, 130.7, 131.7, 132.0, 133.3, 139.1, 139.3, 147.3, 162.0, 172.4. HRMS (ESI) *m*/*z*: calcd. for C_48_H_49_Cl_2_N_2_^+^ 593.2485, obsd 593.2475.

*1,3,3-Trimethyl-2-((E)-2-((E)-6-(2-((E)-1,3,3-trimethylindolin-2-ylidene)ethylidene)-3,4,5,6-tetrahydro-[1,1′-biphenyl]-2-yl)vinyl)-3H-indol-1-ium iodide*, **6k**; yield 68%; m.p. > 260 °C; ^1^H-NMR (CDCl_3_) **δ** 1.04 (t, *J* = 7.2 Hz, 6H), 1.23 (s, 12H), 1.49 (m, 4H), 1.80 (m, 4H), 2.11 (t, *J* = 6.0 Hz, 2H), 2.745 (s, 4H), 4.04 (t, *J* = 7.2 Hz, 4H), 6.10 (d, *J* = 14 Hz, 2H), 7.07 (d, *J* = 11.6 Hz, 2H), 7.22 (m, 6H), 7.33 (t, *J* = 7.2 Hz, 2H), 7.61 (m, 3H); ^13^C NMR (CDCl_3_) 14.0, 20.5, 21.3, 25.0, 27.7, 29.4, 110.4, 122.1, 124.8, 128.2, 128.6, 129.6, 140.8, 142.3. HRMS (ESI) *m*/*z*: calcd. for C_44_H_53_N_2_^+^ 609.4203, obsd 609.4198.

*1-Butyl-2-((E)-2-((E)-6-(2-((E)-1-butyl-5-chloro-3,3-dimethylindolin-2-ylidene)ethylidene)-3,4,5,6-tetra-hydro-[1,1′-biphenyl]-2-yl)vinyl)-5-chloro-3,3-dimethyl-3H-indol-1-ium iodide*, **6l**: Yield 67%; m.p. > 260; ^1^H NMR (CDCl_3_) **δ** 1.03 (t, *J* = 7.2 Hz, 6H), 1.191 (s, 12H), 1.47 (m, 4H), 1.78 (t, *J* = 6.8 Hz, 4H), 2.12 (s, 2H), 2.75 (s, 4H), 4.02 (s, 4H), 6.09 (d, *J* = 12.4 Hz, 2H), 6.98 (d, *J* = 8.0 Hz, 2H), 7.28 (m, 8H), 7.86 (m, 3H); ^13^C NMR (CDCl_3_) **δ** 14.0, 20.4, 21.3, 25.0, 27.7, 29.3, 44.7, 48.6, 100.4, 111.3, 122.7, 128.3, 128.6, 129.6, 130.2, 138.7, 141.0, 142.4, 148.5, 171.0. HRMS (ESI) *m*/*z*: calcd. for C_44_H_51_Cl_2_N_2_^+^ 677.3424, obsd 677.3421.

*1-Butyl-2-((E)-2-((E)-6-(2-((E)-1-butyl-3,3-dimethylindolin-2-ylidene)ethylidene)-3,4,5,6-tetrahydro-[1,1′-biphenyl]-2-yl)vinyl)-3,3-dimethyl-3H-indol-1-ium iodide*, **6m**: Yield 65%; m.p. > 260 °C; ^1^H NMR (CDCl_3_), **δ** 1.04 (t, *J* = 7.2 Hz, 6H), 1.20 (s, 12H), 1.51 (m, 4H), 1.81 (m, 4H), 2.13 (t, *J* = 5.2 Hz, 2H), 2.75 (s, 4H), 4.04 (t, *J* =7.2 Hz, 4H), 6.10 (d, *J* = 14.0 Hz, 2H), 7.07 (d, *J* = 8 Hz, 2H), 7.20 (m, 8H), 7.61 (m, 3H); ^13^C NMR (CDCl_3_) **δ** 14.0, 20.5, 21.3, 25.0, 27.7, 29.4, 44.4, 48.6, 100.0, 110.3, 122.1, 124.7, 128.2, 128.6, 129.6, 132.1, 138.8, 140.8, 142.3, 148.4, 162.9, 171.4. HRMS (ESI) *m*/*z*: calcd. for C_44_H_51_Br_2_N_2_^+^ 765.2414, obsd 765.2422.

*1-Butyl-2-((E)-2-((E)-6-(2-((E)-1-butyl-5-methoxy-3,3-dimethylindolin-2-ylidene)ethylidene)-3,4,5,6-tetrahydro-[1,1′-biphenyl]-2-yl)vinyl)-5-methoxy-3,3-dimethyl-3H-indol-1-ium iodide*, **6n**: Yield 66%; m.p. > 260 °C; ^1^H NMR (CDCl_3_) **δ** 1.01 (t, *J* = 6.8 Hz, 6H), 1.17 (s, 12H), 1.46 (m, 4H), 1.80 (m, 4H), 2.07 (s, 2H), 2.68 (s, 4H), 3.82 (s, 6H), 4.03 (t, *J* = 7.2 Hz, 4H), 6.00 (d, *J* = 14.0 Hz, 2H) 6.78 (s, 2H), 6.88 (d, *J* = 8.4 Hz, 2H), 7.03 (d, *J* = 8.8 Hz, 2H), 7.11 (d, *J* = 14.0 Hz, 2H), 7.21 (d, *J* = 6.8 Hz, 2H), 7.57 (m, 3H); ^13^C NMR (CDCl_3_) **δ** 13.9, 20.4, 21.3, 24.8, 27.7, 29.4, 44.6, 48.7, 56.0, 99.4, 109.1, 111.1, 113.1, 128.1, 128.6, 129.4, 130.9, 135.9, 139.0, 142.4, 146.9, 157.9, 161.4, 170.6. HRMS (ESI) *m*/*z*: calcd. for C_46_H_57_N_2_O_2_^+^ 669.4415, obsd 669.4404.

*3-Butyl-2-((E)-2-((E)-6-((E)-2-(3-butyl-1,1-dimethyl-1,3-dihydro-2H-benzo[e]indol-2-ylidene)ethylidene)-3,4,5,6-tetrahydro-[1,1′-biphenyl]-2-yl)vinyl)-1,1-dimethyl-1H-benzo[e]indol-3-ium iodide*, **6o**: Yield 70%; m.p. >260 °C; ^1^H NMR (CDCl_3_) **δ** 1.03 (t, *J* = 7.2 Hz, 6H), 1.52 (m, 16H), 1.86 (m, 4H), 2.16 (t, *J* = 6.0 Hz, 2H), 2.79 (s, 4H), 4.17 (s, 4H), 6.15 (d, *J* = 14.4 Hz, 2H), 7.34 (m, 6H), 7.45 (d, *J* = 7.6 Hz, 2H), 7.53 (m, 2H), 7.68 (t, *J* = 2.8 Hz, 3H), 7.93 (m, 6H); ^13^C NMR (CDCl_3_) **δ** 14.0, 20.4, 21.4, 25.0, 27.2, 29.7, 44.6, 50.4, 99.6, 110.6, 122.0, 124.8, 127.6, 128.1, 128.4, 128.7, 129.6, 130.1, 130.6, 131.7, 133.2, 139.0, 139.7, 147.3, 172.8. HRMS (ESI) *m*/*z*: calcd. for C_52_H_57_N_2_^+^ 709.4516, obsd 709.4540.

### 3.3. Stock Solutions

Stock solutions of the dyes and standard were prepared by weighing the solid on a 5-digit analytical balance in an amber vial and adding solvent via a class A volumetric pipette to a final concentration of 1.0 mM. The vials were vortexed for 20 s and then sonicated for 15 min to ensure complete dissolution. The stock solutions were stored in a dark freezer at 4 °C when not in use. Working solutions were prepared just prior to use by dilution of the stock to final concentrations.

### 3.4. Method of Determining Molar Absorptivity and Fluorescence Quantum Yield

Stock solutions were used to prepare six dilutions of dyes in ethanol and the standard with concentrations ranging from 1 μM to 4 μM using a class A volumetric pipette in order to maintain absorption between 0.1 and 1.0. The dye solutions were diluted ten-fold for fluorescence in order to minimize inner filter effect. The absorbance spectra of each sample was measured in triplicate from 400 nm to 900 nm. The emission spectrum of each sample was measured in triplicate with a 750 nm excitation wavelength.

For molar absorptivity, the absorbance at the wavelength of maximum absorbance (λ_max_) was determined and the absorbance of each sample at λ_max_ was plotted as a function of dye concentration. The linear regression equation was computed using Microsoft Excel.

The fluorescence quantum yields were determined relative to the indocyanine green standard utilizing the gradient from the plot of integrated fluorescence intensity vs. absorbance (Grad) and the published quantum yield of the standard (φ_S_, 13.2% [29]) as per Equation (1):

φ_D_ = φ_S_ * Grad_D_/Grad_S_ ∗ η^2^_S_/η^2^_D_ (1)

### 3.5. HSA Binding Study

A stock solution of **6a** (4 × 10^−5^ M) and HSA (4 × 10^−5^ M; Sigma Aldrich, St. Louis, MO, USA) were prepared in PBS buffer. Fluorescence titration with HSA concentrations (0–2 μM) were made by mixing 35 μL dye solution with PBS buffer solution with and without HSA to a total volume of 4000 μL in a fluorescence cuvette to make working solutions of 2 μM dye. Fluorescence spectra were measured in duplicate with excitation at 740 nm and slit widths of 5 nm for both excitation and emission.

## 4. Conclusions

A series of 15 phenyl-substituted heptamethine cyanines was synthesized in good yields and characterized by ^1^H and ^13^C NMR. Their optical properties including molar absorptivity, fluorescence, Stokes shift, and quantum yield were measured. The optical properties followed similar trends to previously published cyanine dye **MHI-06**. The binding affinity of one of these heptamethine cyanine dyes to HSA was measured to be 5 orders of magnitude lower than our previous synthesized trimethine cyanines further confirming the hypothesis that the binding affinity of the dyes is not only hydrophobicity dependent, but dependent on steric interferences in the binding site [28]. Because albumin is known to bind a variety of compounds, this information on the steric specificity of these binding sites is of potential interest when developing methods to study them.

## Figures and Tables

**Table 1:** Spectral Characteristics of dyes **Cy-7**, **IR-780** and **6a**–**o**. All optical properties of the dyes were measured in ethanol.

Dye	λ_max_ (nm)	λ_emission_ (nm)	Stokes Shift (nm)	ε (L·mol^−1^·cm^−1^)	Φ (%)	Molecular Brightness (M^−1^·cm^−1^)
**Cy-7**	753	775	22	200,000	28	56,000
**IR-780**	779	799	20	274,000	8.0	20,800
**6a**	759	774	15	265,700	31	82,000
**6b**	765	780	15	261,000	34	88,700
**6c**	767	783	16	275,600	35	96,500
**6d**	782	802	20	249,500	10	25,000
**6e**	798	810	12	255,400	16	40,900
**6f**	760	781	21	263,900	39	102,300
**6g**	769	785	16	286,600	38	109,300
**6h**	770	786	16	282,900	42	119,200
**6i**	786	804	18	143,500	12	17,200
**6j**	797	810	13	231,800	17	39,400
**6k**	763	780	17	198,500	45	89,300
**6l**	772	787	15	123,400	47	58,000
**6m**	773	788	15	239,200	48	113,600
**6n**	789	805	16	249,900	11	27,100
**6o**	800	812	12	226,600	17	38,500

**Table 2:** Physicochemical properties (*in silico*) of dyes **MHI-06** and **6a**–**o** calculated using ChemAxon. The data calculated (at pH 7.4) include: logD, polarizability, dipole moment (debye), number of rotatable bonds, volume (Å^3^), molecular surface area (Å^3^), and molar mass (g/mol).

Dye	log*D*	Polarizability	Dipole Moment	Rot. Bonds	Volume	Molec. Surface Area	Molar Mass
**MHI-06**	4.97	59.45	2.48	8	441.44	693.216	584.54
**6a**	6.07	65.57	8.35	4	519.85	814.001	652.66
**6b**	7.28	69.15	12.3	4	547.45	845.982	721.55
**6c**	7.61	70.67	13.02	4	556.15	854.732	810.46
**6d**	5.75	70.51	27.66	6	569.71	907.778	712.72
**6e**	8.05	80.82	11.87	4	604.27	936.392	752.78
**6f**	6.75	69.26	3.2	6	552.14	874.685	680.72
**6g**	7.99	72.84	4.81	6	579.48	906.491	749.6
**6h**	8.32	74.35	4.97	6	588.47	915.629	838.51
**6i**	6.47	74.2	25.29	8	603.83	969.908	740.77
**6j**	8.76	84.51	4.59	6	638.63	999.272	7803.84
**6k**	8.72	76.65	2.85	10	620.01	996.744	736.81
**6l**	9.92	80.23	4.63	10	647.91	1029.597	805.71
**6m**	10.25	81.71	4.96	10	656.64	1038.143	894.62
**6n**	8.40	81.59	26.66	12	672.63	1094.66	796.88
**6o**	10.69	91.9	3.96	10	708.29	1124.8	836.95

## Supplementary Materials

Supplementary materials are available online.
