# Supplementary material for: Synthesis and Optical Properties of Near-Infrared meso-Phenyl-Substituted Symmetric Heptamethine Cyanine Dyes
Source: Molecules. 2018 Jan 24;23(2):226. doi: 10.3390/molecules23020226 (PMC6017188; doi:10.3390/molecules23020226)
Supplement: Supplementary file 1 [file molecules-23-00226-s001.pdf]

Synthesis and optical properties of heptamethine cyanine dyes containing a phenyl ring at the *meso*-position.

Andrew Levitz[#], Fahad Marmarchi[#], and Maged Henary[*]

Department of Chemistry, Georgia State University, Atlanta, GA 30302, USA

[*]To whom correspondence should be addressed. [#]These authors contributed equally to this work.

***Corresponding Author**:

Maged Henary, Ph.D.

100 Piedmont Ave. #315

Atlanta, GA 30303

Office: 404-413-5566; Fax: 404-413-5505

Email: mhenary1@gsu.edu

Supplemental Information

Table of content:

^{1}H and ^{13}C NMR spectra of the dyes synthesized in this study

Absorbance and fluorescence spectra of the of the dyes synthesized in this study

Beer-Lambert plots of the of the dyes synthesized in this study

HSA binding spectra of 6k

Mass spectra of the dyes synthesized in this study

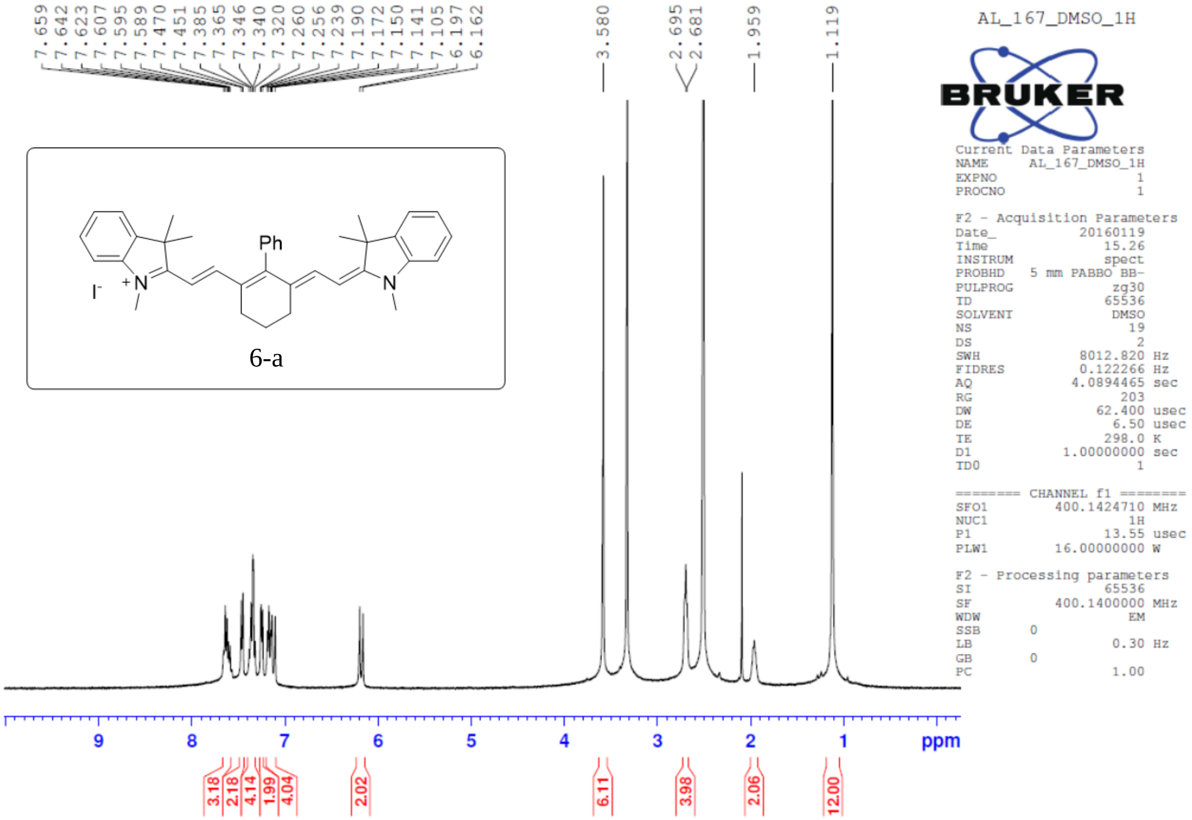

7.659
7.642
7.623
7.607
7.595
7.589
7.470
7.451
7.385
7.365
7.346
7.340
7.320
7.260
7.256
7.239
7.190
7.172
7.150
7.141
7.105
6.197
6.162
3.580
2.695
2.681
1.959
1.119
I⁻
+N
Ph
N
6-a
9
8
7
6
5
4
3
2
1
ppm
3.18
2.18
4.14
1.99
4.04
2.02
6.11
3.98
2.06
12.00
AL_167_DMSO_1H
BRUKER
Current Data Parameters
NAME AL_167_DMSO_1H
EXPNO 1
PROCNO 1
F2 - Acquisition Parameters
Date_ 20160119
Time 15.26
INSTRUM spect
PROBHD 5 mm PABBO BB-
PULPROG zg30
TD 65536
SOLVENT DMSO
NS 19
DS 2
SWH 8012.820 Hz
FIDRES 0.122266 Hz
AQ 4.0894465 sec
RG 203
DW 62.400 usec
DE 6.50 usec
TE 298.0 K
D1 1.00000000 sec
TD0 1
======== CHANNEL f1 ========
SFO1 400.1424710 MHz
NUC1 1H
P1 13.55 usec
PLW1 16.00000000 W
F2 - Processing parameters
SI 65536
SF 400.1400000 MHz
WDW EM
SSB 0
LB 0.30 Hz
GB 0
PC 1.00

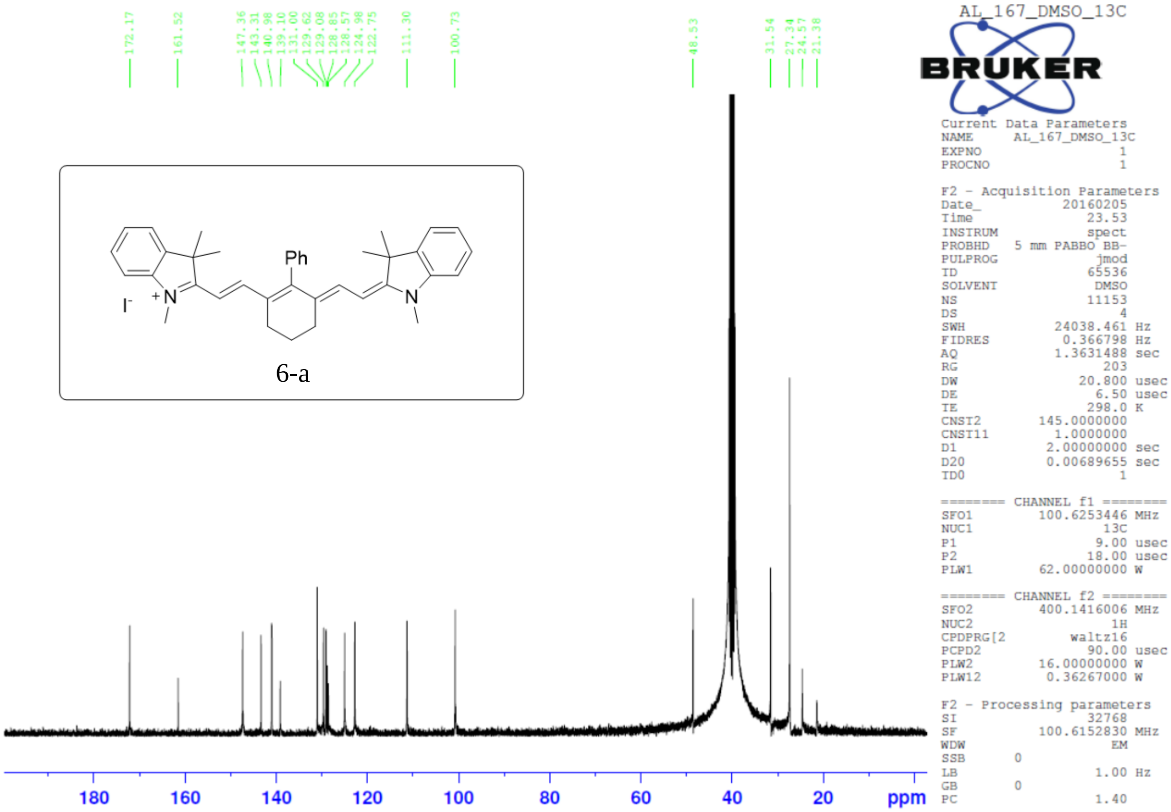

172.17
161.52
147.36
143.31
140.98
139.10
131.00
129.62
129.08
128.85
128.57
124.98
122.75
111.30
100.73
48.53
31.54
27.34
24.57
21.38
I⁻
+N
Ph
N
6-a
180
160
140
120
100
80
60
40
20
ppm
AL_167_DMSO_13C
BRUKER
Current Data Parameters
NAME AL_167_DMSO_13C
EXPNO 1
PROCNO 1
F2 - Acquisition Parameters
Date_ 20160205
Time 23.53
INSTRUM spect
PROBHD 5 mm PABBO BB-
PULPROG jmod
TD 65536
SOLVENT DMSO
NS 11153
DS 4
SWH 24038.461 Hz
FIDRES 0.366798 Hz
AQ 1.3631488 sec
RG 203
DW 20.800 usec
DE 6.50 usec
TE 298.0 K
CNST2 145.0000000
CNST11 1.0000000
D1 2.00000000 sec
D20 0.00689655 sec
TD0 1
======== CHANNEL f1 ========
SFO1 100.6253446 MHz
NUC1 13C
P1 9.00 usec
P2 18.00 usec
PLW1 62.00000000 W
======== CHANNEL f2 ========
SFO2 400.1416006 MHz
NUC2 1H
CPDPRG[2 waltz16
PCPD2 90.00 usec
PLW2 16.00000000 W
PLW12 0.36267000 W
F2 - Processing parameters
SI 32768
SF 100.6152830 MHz
WDW EM
SSB 0
LB 1.00 Hz
GB 0
PC 1.40

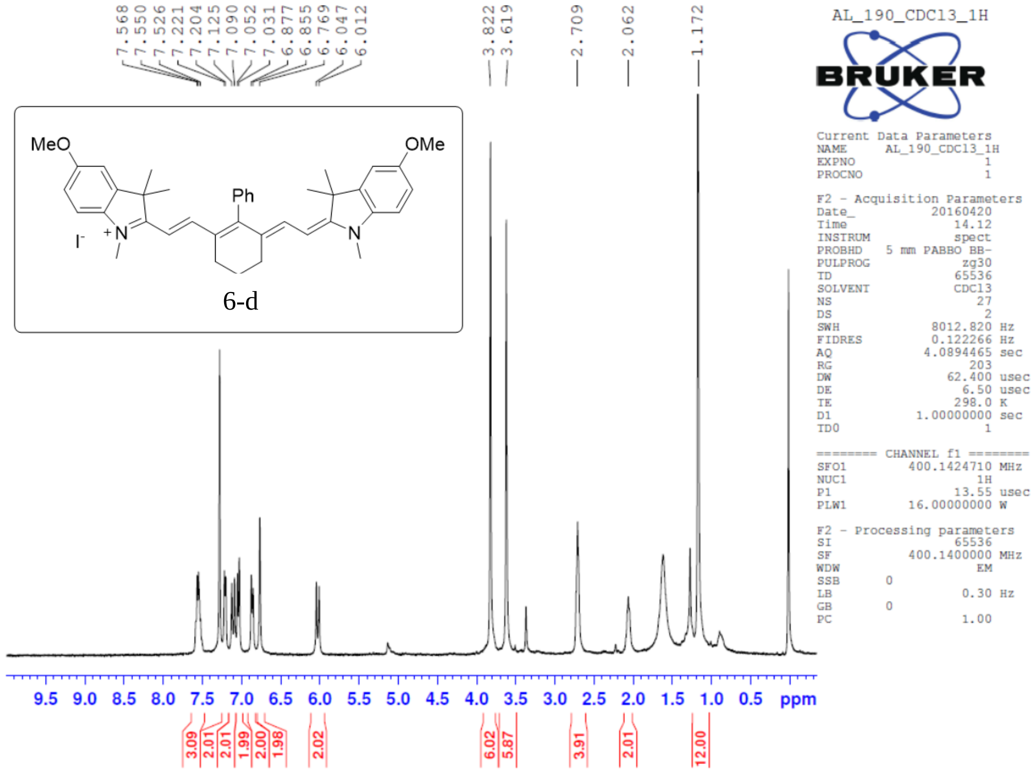

7.568 7.550 7.526 7.221 7.204 7.125 7.090 7.052 7.031 6.877 6.855 6.769 6.047 6.012
3.822 3.619
2.709
2.062
1.172
MeO
OMe
Ph
I⁻
+N
N
6-d
9.5 9.0 8.5 8.0 7.5 7.0 6.5 6.0 5.5 5.0 4.5 4.0 3.5 3.0 2.5 2.0 1.5 1.0 0.5 ppm
3.09 2.01 2.01 1.99 2.00 1.98 2.02 6.02 5.87 3.91 2.01 12.00
AL_190_CDC13_1H
Current Data Parameters
NAME AL_190_CDC13_1H
EXPNO 1
PROCNO 1
F2 - Acquisition Parameters
Date_ 20160420
Time 14.12
INSTRUM spect
PROBHD 5 mm PABBO BB-
PULPROG zg30
TD 65536
SOLVENT CDC13
NS 27
DS 2
SWH 8012.820 Hz
FIDRES 0.122266 Hz
AQ 4.0894465 sec
RG 203
DW 62.400 usec
DE 6.50 usec
TE 298.0 K
D1 1.00000000 sec
TD0 1
======== CHANNEL f1 ========
SFO1 400.1424710 MHz
NUC1 1H
P1 13.55 usec
PLW1 16.00000000 W
F2 - Processing parameters
SI 65536
SF 400.1400000 MHz
WDW EM
SSB 0
LB 0.30 Hz
GB 0
PC 1.00

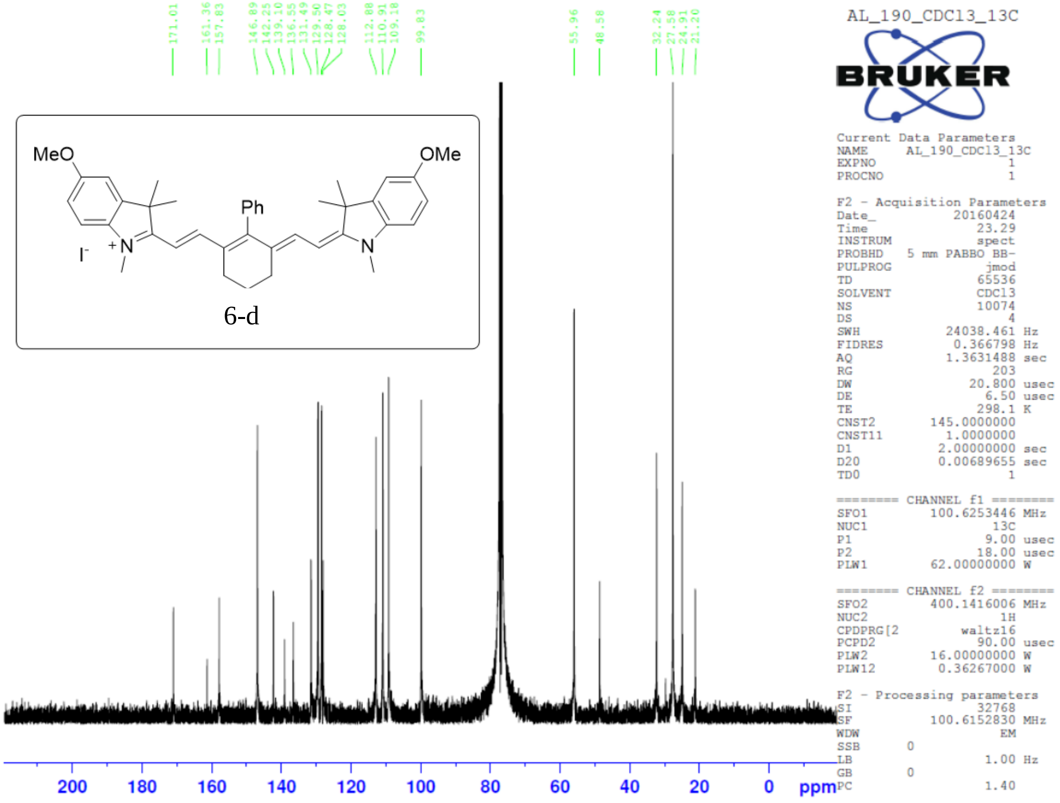

171.01 161.36 157.83 146.89 142.25 139.10 136.55 131.49 129.50 128.47 128.03 112.88 110.91 109.18 99.83
55.96 48.58 32.24 27.58 24.91 21.20
MeO
OMe
Ph
I⁻
+N
N
6-d
200 180 160 140 120 100 80 60 40 20 0 ppm
AL_190_CDC13_13C
Current Data Parameters
NAME AL_190_CDC13_13C
EXPNO 1
PROCNO 1
F2 - Acquisition Parameters
Date_ 20160424
Time 23.29
INSTRUM spect
PROBHD 5 mm PABBO BB-
PULPROG jmod
TD 65536
SOLVENT CDC13
NS 10074
DS 4
SWH 24038.461 Hz
FIDRES 0.366798 Hz
AQ 1.3631488 sec
RG 203
DW 20.800 usec
DE 6.50 usec
TE 298.1 K
CNST2 145.0000000
CNST11 1.0000000
D1 2.00000000 sec
D20 0.00689655 sec
TD0 1
======== CHANNEL f1 ========
SFO1 100.6253446 MHz
NUC1 13C
P1 9.00 usec
P2 18.00 usec
PLW1 62.00000000 W
======== CHANNEL f2 ========
SFO2 400.1416006 MHz
NUC2 1H
CPDPRG[2 waltz16
PCPD2 90.00 usec
PLW2 16.00000000 W
PLW12 0.36267000 W
F2 - Processing parameters
SI 32768
SF 100.6152830 MHz
WDW EM
SSB 0
LB 1.00 Hz
GB 0
PC 1.40

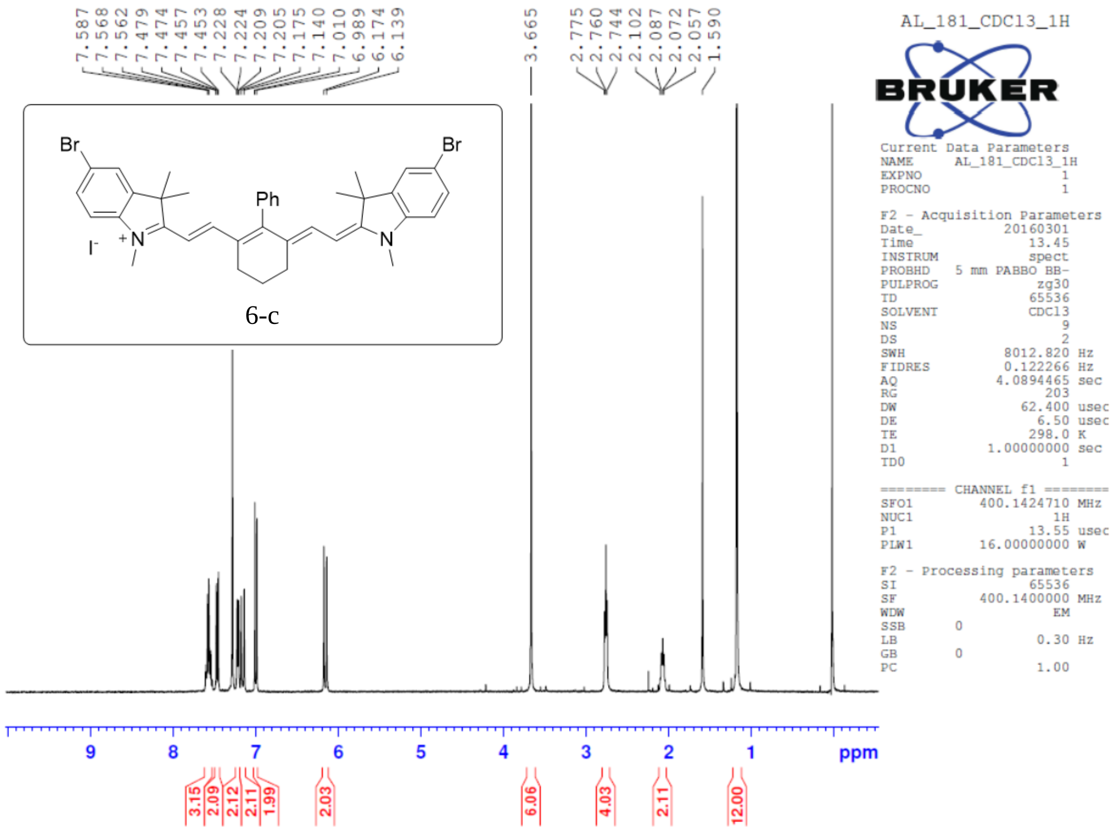
7.587
7.568
7.562
7.479
7.474
7.457
7.453
7.228
7.224
7.209
7.205
7.175
7.140
7.010
6.989
6.174
6.139
3.665
2.775
2.760
2.744
2.102
2.087
2.072
2.057
1.590
Br
Br
Ph
I-
+N
N
6-c
9
8
7
6
5
4
3
2
1
ppm
3.15
2.09
2.12
2.11
1.99
2.03
6.06
4.03
2.11
12.00
AL_181_CDCl3_1H
BRUKER
Current Data Parameters
NAME AL_181_CDCl3_1H
EXPNO 1
PROCNO 1
F2 - Acquisition Parameters
Date_ 20160301
Time 13.45
INSTRUM spect
PROBHD 5 mm PABBO BB-
PULPROG zg30
TD 65536
SOLVENT CDCl3
NS 9
DS 2
SWH 8012.820 Hz
FIDRES 0.122266 Hz
AQ 4.0894465 sec
RG 203
DW 62.400 usec
DE 6.50 usec
TE 298.0 K
D1 1.00000000 sec
TD0 1
======== CHANNEL f1 ========
SFO1 400.1424710 MHz
NUC1 1H
P1 13.55 usec
PLW1 16.00000000 W
F2 - Processing parameters
SI 65536
SF 400.1400000 MHz
WDW EM
SSB 0
LB 0.30 Hz
GB 0
PC 1.00

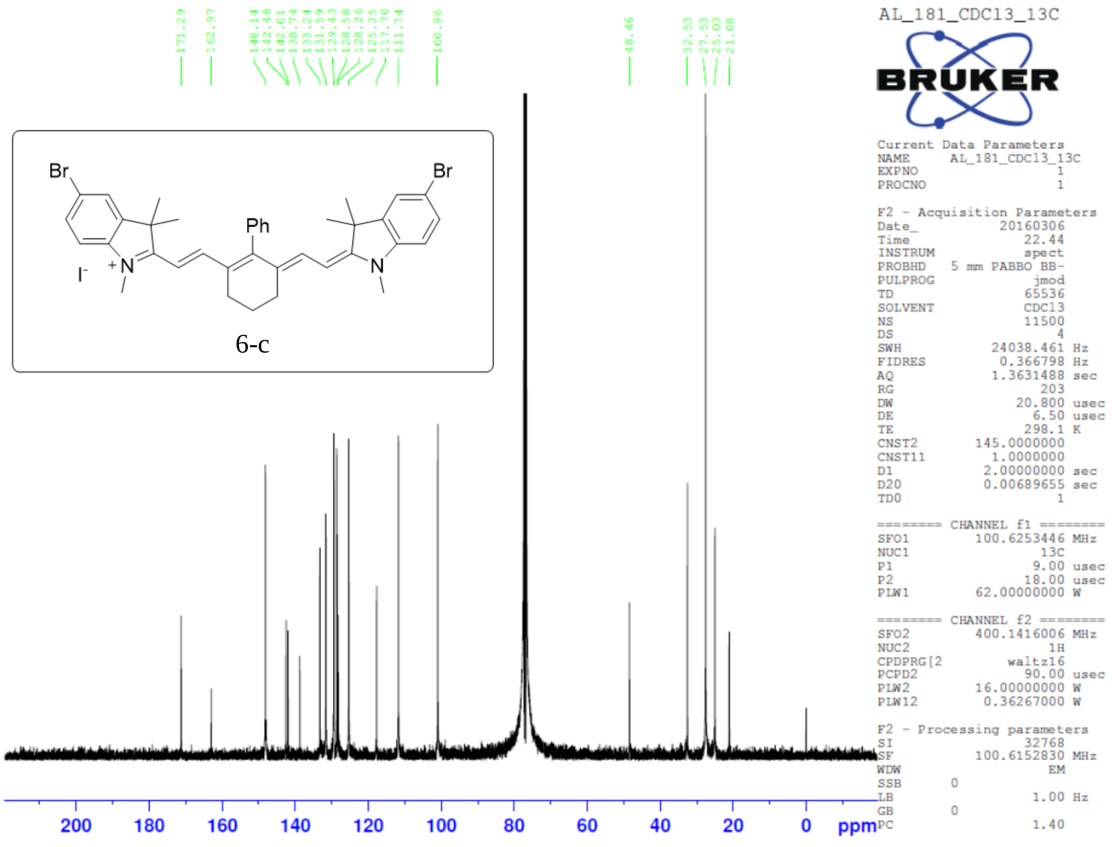
171.29
162.97
148.14
142.48
138.74
133.24
131.59
129.43
128.58
128.26
125.35
117.90
111.74
100.96
48.46
32.53
27.53
25.03
21.08
Br
Br
Ph
I-
+N
N
6-c
200
180
160
140
120
100
80
60
40
20
0
ppm
AL_181_CDCl3_13C
BRUKER
Current Data Parameters
NAME AL_181_CDCl3_13C
EXPNO 1
PROCNO 1
F2 - Acquisition Parameters
Date_ 20160306
Time 22.44
INSTRUM spect
PROBHD 5 mm PABBO BB-
PULPROG jmod
TD 65536
SOLVENT CDCl3
NS 11500
DS 4
SWH 24038.461 Hz
FIDRES 0.366798 Hz
AQ 1.3631488 sec
RG 203
DW 20.800 usec
DE 6.50 usec
TE 298.1 K
CNST2 145.0000000
CNST11 1.0000000
D1 2.00000000 sec
D20 0.00689655 sec
TD0 1
======== CHANNEL f1 ========
SFO1 100.6253446 MHz
NUC1 13C
P1 9.00 usec
P2 18.00 usec
PLW1 62.00000000 W
======== CHANNEL f2 ========
SFO2 400.1416006 MHz
NUC2 1H
CPDPRG[2 waltz16
PCPD2 90.00 usec
PLW2 16.00000000 W
PLW12 0.36267000 W
F2 - Processing parameters
SI 32768
SF 100.6152830 MHz
WDW EM
SSB 0
LB 1.00 Hz
GB 0
PC 1.40

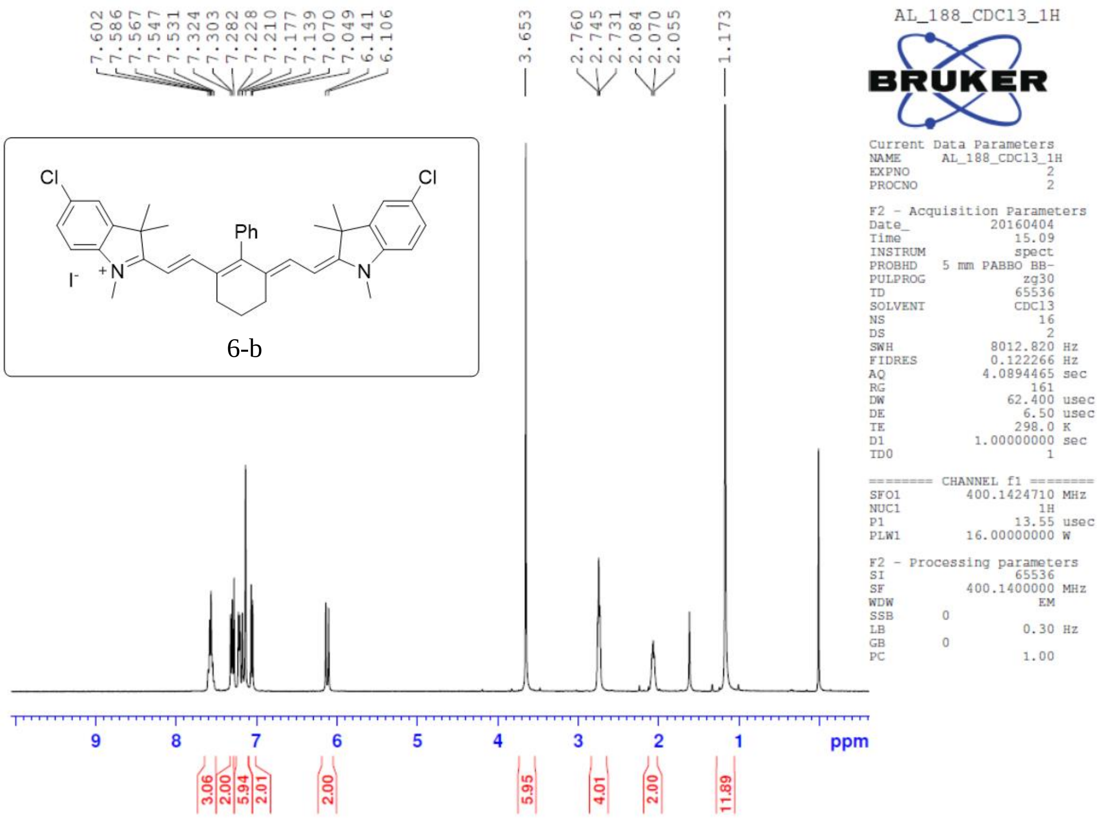

7.602
7.586
7.567
7.547
7.531
7.324
7.303
7.282
7.228
7.210
7.177
7.139
7.070
7.049
6.141
6.106
3.653
2.760
2.745
2.731
2.084
2.070
2.055
1.173
AL_188_CDCl3_1H
BRUKER
Cl
Cl
Ph
I-
+N
N
6-b
Current Data Parameters
NAME AL_188_CDCl3_1H
EXPNO 2
PROCNO 2
F2 - Acquisition Parameters
Date_ 20160404
Time 15.09
INSTRUM spect
PROBHD 5 mm PABBO BB-
PULPROG zg30
TD 65536
SOLVENT CDCl3
NS 16
DS 2
SWH 8012.820 Hz
FIDRES 0.122266 Hz
AQ 4.0894465 sec
RG 161
DW 62.400 usec
DE 6.50 usec
TE 298.0 K
D1 1.00000000 sec
TD0 1
======== CHANNEL f1 ========
SFO1 400.1424710 MHz
NUC1 1H
P1 13.55 usec
PLW1 16.00000000 W
F2 - Processing parameters
SI 65536
SF 400.1400000 MHz
WDW EM
SSB 0
LB 0.30 Hz
GB 0
PC 1.00
9
8
7
6
5
4
3
2
1
ppm
3.06
2.00
5.94
2.01
2.00
5.95
4.01
2.00
11.89

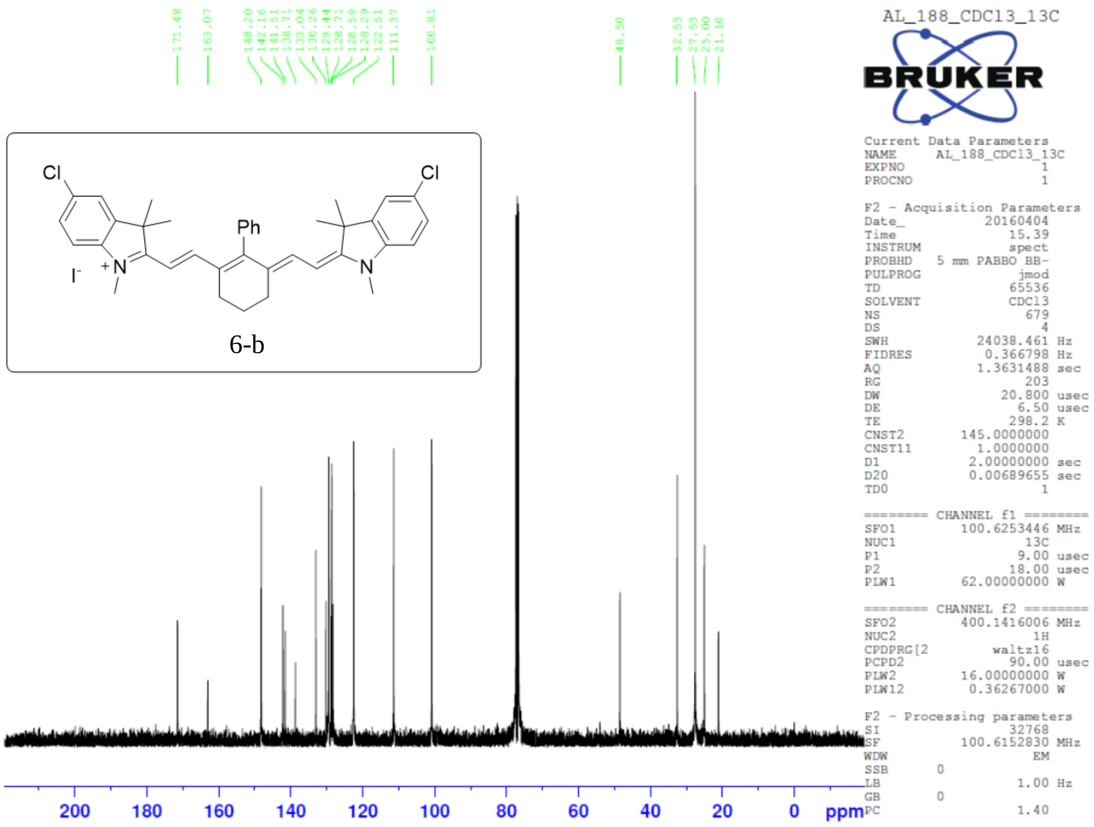

171.48
163.07
148.20
142.16
141.51
138.71
133.04
130.26
129.44
128.71
128.59
128.29
122.51
111.37
100.81
48.50
32.53
27.53
25.00
21.10
AL_188_CDCl3_13C
BRUKER
Cl
Cl
Ph
I-
+N
N
6-b
Current Data Parameters
NAME AL_188_CDCl3_13C
EXPNO 1
PROCNO 1
F2 - Acquisition Parameters
Date_ 20160404
Time 15.39
INSTRUM spect
PROBHD 5 mm PABBO BB-
PULPROG jmod
TD 65536
SOLVENT CDCl3
NS 679
DS 4
SWH 24038.461 Hz
FIDRES 0.366798 Hz
AQ 1.3631488 sec
RG 203
DW 20.800 usec
DE 6.50 usec
TE 298.2 K
CNST2 145.0000000
CNST11 1.0000000
D1 2.00000000 sec
D20 0.00689655 sec
TD0 1
======== CHANNEL f1 ========
SFO1 100.6253446 MHz
NUC1 13C
P1 9.00 usec
P2 18.00 usec
PLW1 62.00000000 W
======== CHANNEL f2 ========
SFO2 400.1416006 MHz
NUC2 1H
CPDPRG[2 waltz16
PCPD2 90.00 usec
PLW2 16.00000000 W
PLW12 0.36267000 W
F2 - Processing parameters
SI 32768
SF 100.6152830 MHz
WDW EM
SSB 0
LB 1.00 Hz
GB 0
PC 1.40
200
180
160
140
120
100
80
60
40
20
0
ppm

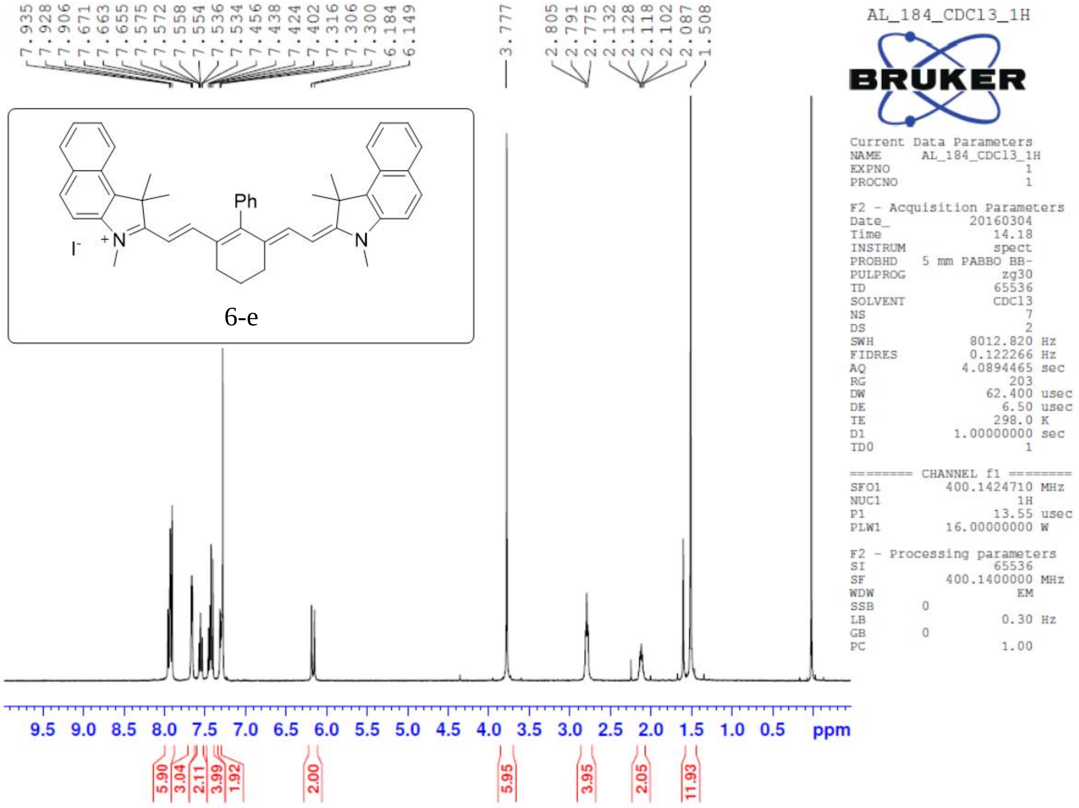
7.935
7.928
7.906
7.671
7.663
7.655
7.575
7.572
7.558
7.554
7.536
7.534
7.456
7.438
7.424
7.402
7.316
7.306
7.300
6.184
6.149
3.777
2.805
2.791
2.775
2.132
2.128
2.118
2.102
2.087
1.508
Ph
I⁻
+N
N
6-e
9.5 9.0 8.5 8.0 7.5 7.0 6.5 6.0 5.5 5.0 4.5 4.0 3.5 3.0 2.5 2.0 1.5 1.0 0.5 ppm
5.90
3.04
2.11
3.99
1.92
2.00
5.95
3.95
2.05
11.93
AL_184_CDCl3_1H
BRUKER
Current Data Parameters
NAME AL_184_CDCl3_1H
EXPNO 1
PROCNO 1
F2 - Acquisition Parameters
Date_ 20160304
Time 14.18
INSTRUM spect
PROBHD 5 mm PABBO BB-
PULPROG zg30
TD 65536
SOLVENT CDCl3
NS 7
DS 2
SWH 8012.820 Hz
FIDRES 0.122266 Hz
AQ 4.0894465 sec
RG 203
DW 62.400 usec
DE 6.50 usec
TE 298.0 K
D1 1.00000000 sec
TD0 1
======== CHANNEL f1 ========
SFO1 400.1424710 MHz
NUC1 1H
P1 13.55 usec
PLW1 16.00000000 W
F2 - Processing parameters
SI 65536
SF 400.1400000 MHz
WDW EM
SSB 0
LB 0.30 Hz
GB 0
PC 1.00

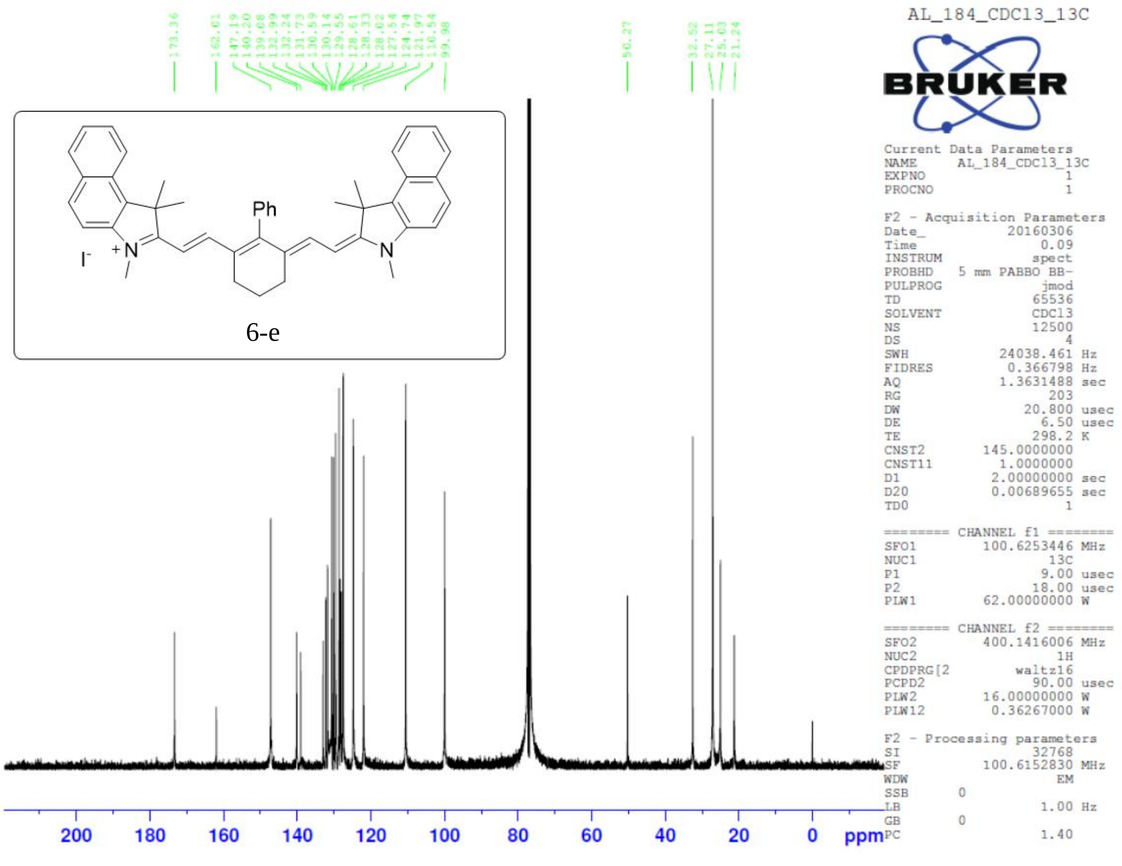
173.36
162.01
147.19
140.20
139.68
132.99
132.24
131.73
130.59
130.14
129.55
128.61
128.33
128.02
127.54
124.74
121.97
110.54
99.98
50.27
32.52
27.11
25.03
21.24
Ph
I⁻
+N
N
6-e
200 180 160 140 120 100 80 60 40 20 0 ppm
AL_184_CDCl3_13C
BRUKER
Current Data Parameters
NAME AL_184_CDCl3_13C
EXPNO 1
PROCNO 1
F2 - Acquisition Parameters
Date_ 20160306
Time 0.09
INSTRUM spect
PROBHD 5 mm PABBO BB-
PULPROG jmod
TD 65536
SOLVENT CDCl3
NS 12500
DS 4
SWH 24038.461 Hz
FIDRES 0.366798 Hz
AQ 1.3631488 sec
RG 203
DW 20.800 usec
DE 6.50 usec
TE 298.2 K
CNST2 145.0000000
CNST11 1.0000000
D1 2.00000000 sec
D20 0.00689655 sec
TD0 1
======== CHANNEL f1 ========
SFO1 100.6253446 MHz
NUC1 13C
P1 9.00 usec
P2 18.00 usec
PLW1 62.00000000 W
======== CHANNEL f2 ========
SFO2 400.1416006 MHz
NUC2 1H
CPDPRG[2 waltz16
PCPD2 90.00 usec
PLW2 16.00000000 W
PLW12 0.36267000 W
F2 - Processing parameters
SI 32768
SF 100.6152830 MHz
WDW EM
SSB 0
LB 1.00 Hz
GB 0
PC 1.40

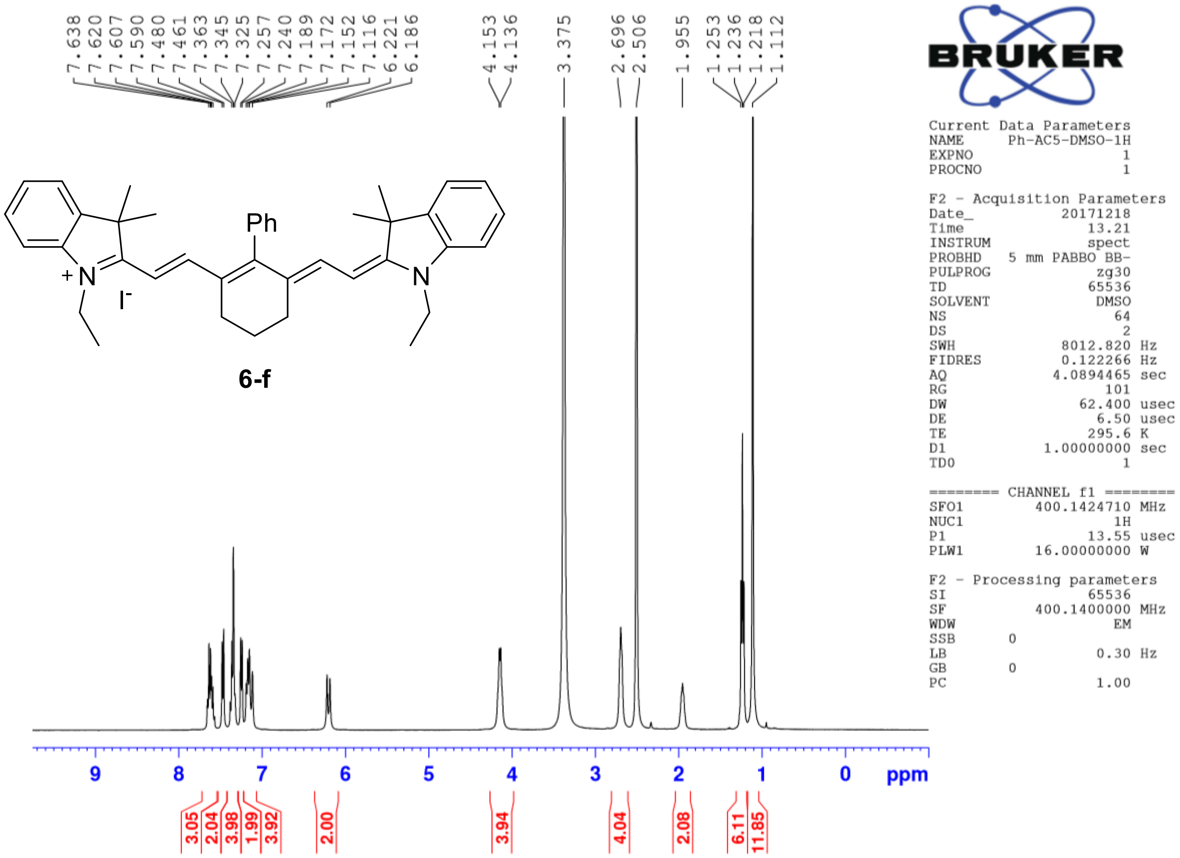
Ph
N
+N
I-
6-f
7.638
7.620
7.607
7.590
7.480
7.461
7.363
7.345
7.325
7.257
7.240
7.189
7.172
7.152
7.116
6.221
6.186
4.153
4.136
3.375
2.696
2.506
1.955
1.253
1.236
1.218
1.112
9 8 7 6 5 4 3 2 1 0 ppm
3.05 2.04 3.98 1.99 3.92 2.00 3.94 4.04 2.08 6.11 11.85
BRUKER
Current Data Parameters
NAME Ph-AC5-DMSO-1H
EXPNO 1
PROCNO 1
F2 - Acquisition Parameters
Date_ 20171218
Time 13.21
INSTRUM spect
PROBHD 5 mm PABBO BB-
PULPROG zg30
TD 65536
SOLVENT DMSO
NS 64
DS 2
SWH 8012.820 Hz
FIDRES 0.122266 Hz
AQ 4.0894465 sec
RG 101
DW 62.400 usec
DE 6.50 usec
TE 295.6 K
D1 1.00000000 sec
TD0 1
======== CHANNEL f1 ========
SFO1 400.1424710 MHz
NUC1 1H
P1 13.55 usec
PLW1 16.00000000 W
F2 - Processing parameters
SI 65536
SF 400.1400000 MHz
WDW EM
SSB 0
LB 0.30 Hz
GB 0
PC 1.00

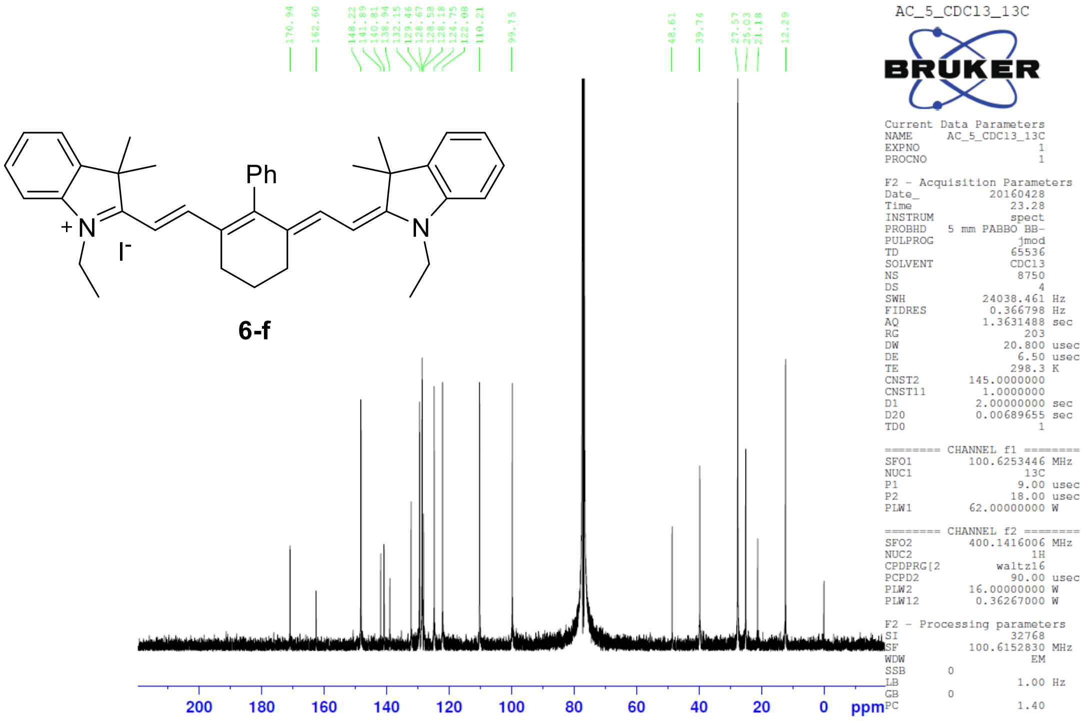
Ph
N
+N
I-
6-f
170.94
162.60
148.22
141.89
140.81
138.94
132.15
129.46
128.67
128.58
128.18
124.75
122.08
110.21
99.75
48.61
39.74
27.57
25.03
21.18
12.29
200 180 160 140 120 100 80 60 40 20 0 ppm
AC_5_CDC13_13C
BRUKER
Current Data Parameters
NAME AC_5_CDC13_13C
EXPNO 1
PROCNO 1
F2 - Acquisition Parameters
Date_ 20160428
Time 23.28
INSTRUM spect
PROBHD 5 mm PABBO BB-
PULPROG jmod
TD 65536
SOLVENT CDC13
NS 8750
DS 4
SWH 24038.461 Hz
FIDRES 0.366798 Hz
AQ 1.3631488 sec
RG 203
DW 20.800 usec
DE 6.50 usec
TE 298.3 K
CNST2 145.0000000
CNST11 1.0000000
D1 2.00000000 sec
D20 0.00689655 sec
TD0 1
======== CHANNEL f1 ========
SFO1 100.6253446 MHz
NUC1 13C
P1 9.00 usec
P2 18.00 usec
PLW1 62.00000000 W
======== CHANNEL f2 ========
SFO2 400.1416006 MHz
NUC2 1H
CPDPRG[2 waltz16
PCPD2 90.00 usec
PLW2 16.00000000 W
PLW12 0.36267000 W
F2 - Processing parameters
SI 32768
SF 100.6152830 MHz
WDW EM
SSB 0
LB 1.00 Hz
GB 0
PC 1.40

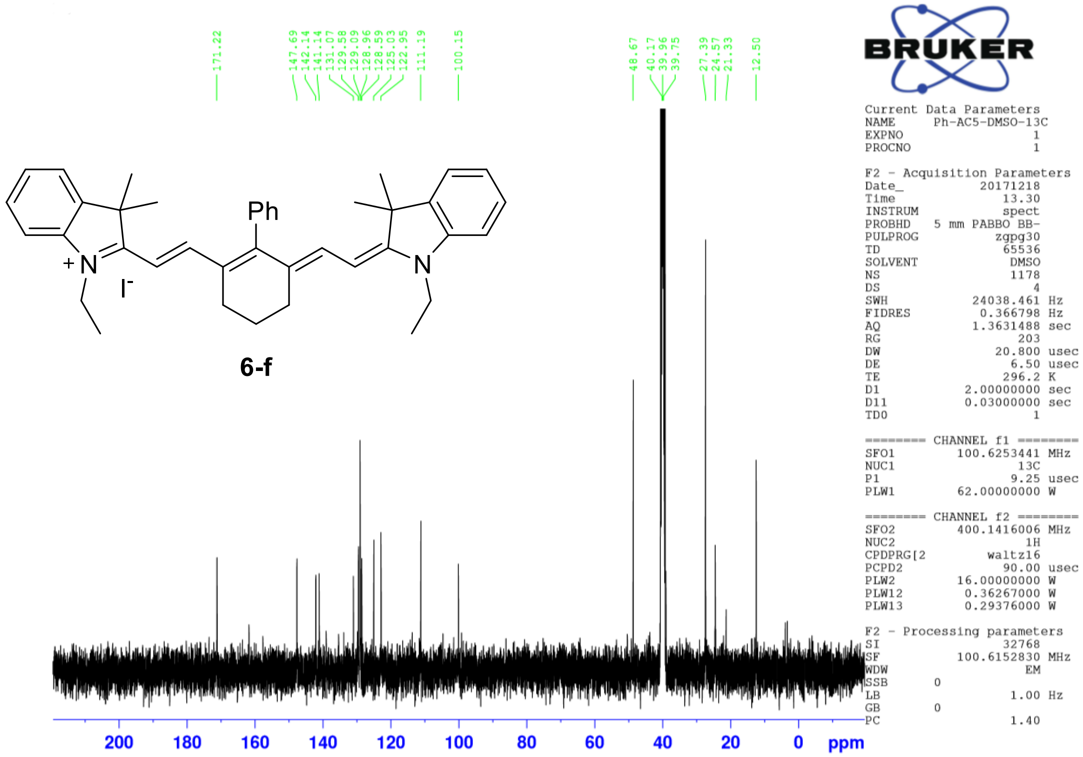
171.22
147.69
142.14
141.14
131.07
129.58
129.09
128.96
128.59
125.03
122.95
111.19
100.15
48.67
40.17
39.96
39.75
27.39
24.57
21.33
12.50
Ph
N
I-
N
6-f
200 180 160 140 120 100 80 60 40 20 0 ppm
BRUKER
Current Data Parameters
NAME Ph-AC5-DMSO-13C
EXPNO 1
PROCNO 1
F2 - Acquisition Parameters
Date_ 20171218
Time 13.30
INSTRUM spect
PROBHD 5 mm PABBO BB-
PULPROG zgpg30
TD 65536
SOLVENT DMSO
NS 1178
DS 4
SWH 24038.461 Hz
FIDRES 0.366798 Hz
AQ 1.3631488 sec
RG 203
DW 20.800 usec
DE 6.50 usec
TE 296.2 K
D1 2.00000000 sec
D11 0.03000000 sec
TD0 1
======== CHANNEL f1 ========
SFO1 100.6253441 MHz
NUC1 13C
P1 9.25 usec
PLW1 62.00000000 W
======== CHANNEL f2 ========
SFO2 400.1416006 MHz
NUC2 1H
CPDPRG[2 waltz16
PCPD2 90.00 usec
PLW2 16.00000000 W
PLW12 0.36267000 W
PLW13 0.29376000 W
F2 - Processing parameters
SI 32768
SF 100.6152830 MHz
WDW EM
SSB 0
LB 1.00 Hz
GB 0
PC 1.40

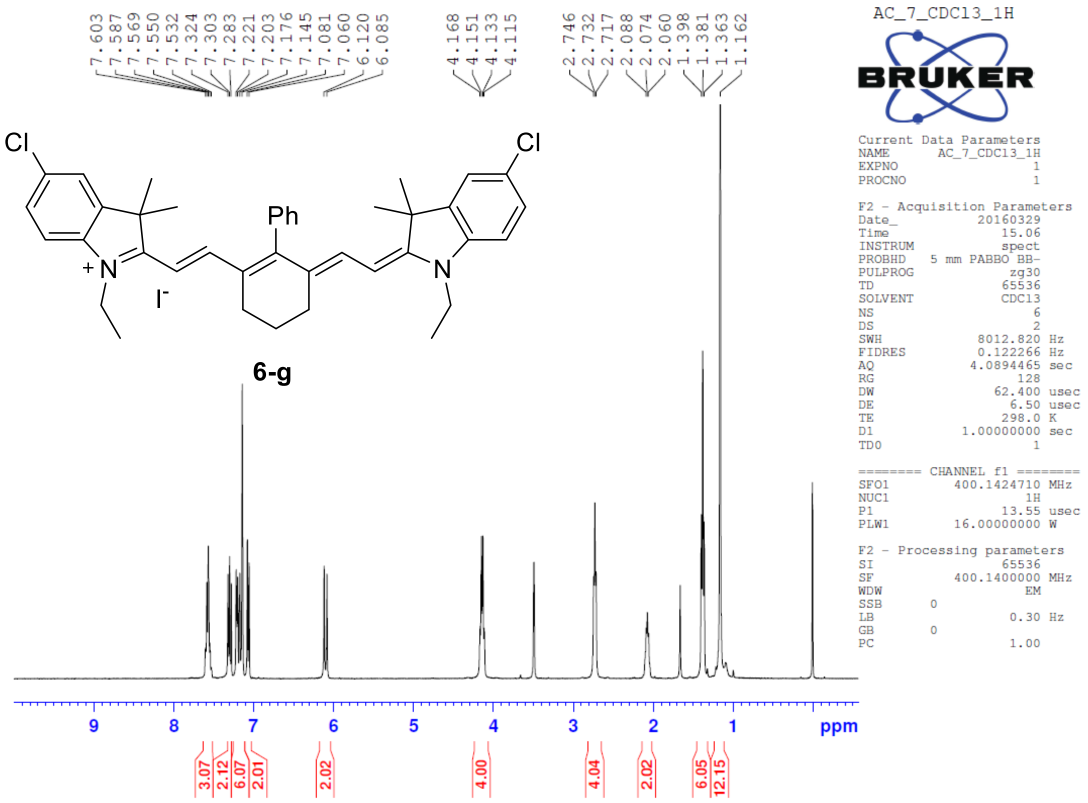
7.603 7.587 7.569 7.550 7.532 7.324 7.303 7.283 7.221 7.203 7.176 7.145 7.081 7.060 6.120 6.085
4.168 4.151 4.133 4.115
2.746 2.732 2.717 2.088 2.074 2.060 1.398 1.381 1.363 1.162
Cl
Cl
Ph
N
+N
I-
6-g
9 8 7 6 5 4 3 2 1 ppm
3.07 2.12 6.07 2.01 2.02 4.00 4.04 2.02 6.05 12.15
AC_7_CDCl3_1H
BRUKER
Current Data Parameters
NAME AC_7_CDCl3_1H
EXPNO 1
PROCNO 1
F2 - Acquisition Parameters
Date_ 20160329
Time 15.06
INSTRUM spect
PROBHD 5 mm PABBO BB-
PULPROG zg30
TD 65536
SOLVENT CDCl3
NS 6
DS 2
SWH 8012.820 Hz
FIDRES 0.122266 Hz
AQ 4.0894465 sec
RG 128
DW 62.400 usec
DE 6.50 usec
TE 298.0 K
D1 1.00000000 sec
TD0 1
======== CHANNEL f1 ========
SFO1 400.1424710 MHz
NUC1 1H
P1 13.55 usec
PLW1 16.00000000 W
F2 - Processing parameters
SI 65536
SF 400.1400000 MHz
WDW EM
SSB 0
LB 0.30 Hz
GB 0
PC 1.00

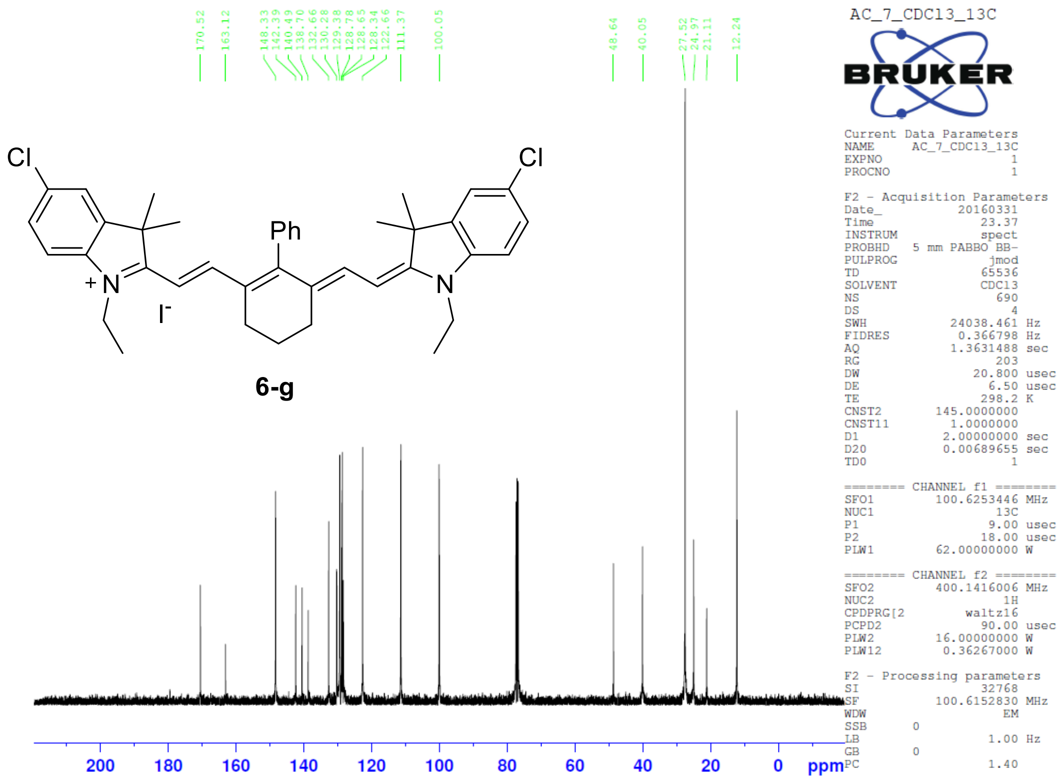
170.52 163.12 148.33 142.39 140.49 138.76 132.66 130.28 129.38 128.78 128.65 128.34 122.66 111.37 100.05
48.64 40.05 27.52 24.97 21.11 12.24
Cl
Cl
Ph
N
+N
I-
6-g
200 180 160 140 120 100 80 60 40 20 0 ppm
AC_7_CDCl3_13C
BRUKER
Current Data Parameters
NAME AC_7_CDCl3_13C
EXPNO 1
PROCNO 1
F2 - Acquisition Parameters
Date_ 20160331
Time 23.37
INSTRUM spect
PROBHD 5 mm PABBO BB-
PULPROG jmod
TD 65536
SOLVENT CDCl3
NS 690
DS 4
SWH 24038.461 Hz
FIDRES 0.366798 Hz
AQ 1.3631488 sec
RG 203
DW 20.800 usec
DE 6.50 usec
TE 298.2 K
CNST2 145.0000000
CNST11 1.0000000
D1 2.00000000 sec
D20 0.00689655 sec
TD0 1
======== CHANNEL f1 ========
SFO1 100.6253446 MHz
NUC1 13C
P1 9.00 usec
P2 18.00 usec
PLW1 62.00000000 W
======== CHANNEL f2 ========
SFO2 400.1416006 MHz
NUC2 1H
CPDPRG[2 waltz16
PCPD2 90.00 usec
PLW2 16.00000000 W
PLW12 0.36267000 W
F2 - Processing parameters
SI 32768
SF 100.6152830 MHz
WDW EM
SSB 0
LB 1.00 Hz
GB 0
PC 1.40

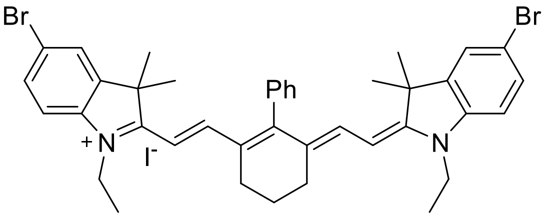
Br
Br
Ph
+N
I-
N

6-h

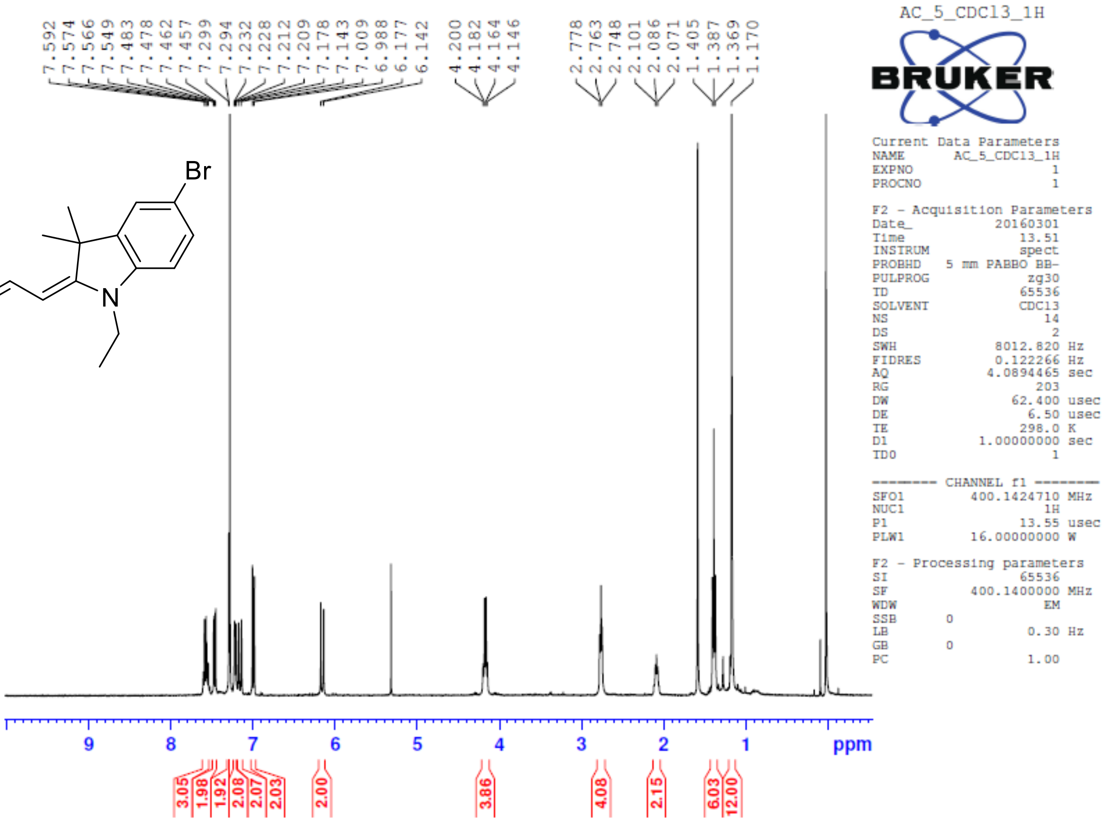
7.592
7.574
7.566
7.549
7.483
7.478
7.462
7.457
7.299
7.294
7.232
7.228
7.212
7.209
7.178
7.143
7.009
6.988
6.177
6.142
4.200
4.182
4.164
4.146
2.778
2.763
2.748
2.101
2.086
2.071
1.405
1.387
1.369
1.170
9
8
7
6
5
4
3
2
1
ppm
3.05
1.98
1.92
2.08
2.07
2.03
2.00
3.86
4.08
2.15
6.03
12.00
AC_5_CDC13_1H
BRUKER
Current Data Parameters
NAME AC_5_CDC13_1H
EXPNO 1
PROCNO 1
F2 - Acquisition Parameters
Date_ 20160301
Time 13.51
INSTRUM spect
PROBHD 5 mm PABBO BB-
PULPROG zg30
TD 65536
SOLVENT CDC13
NS 14
DS 2
SWH 8012.820 Hz
FIDRES 0.122266 Hz
AQ 4.0894465 sec
RG 203
DW 62.400 usec
DE 6.50 usec
TE 298.0 K
D1 1.00000000 sec
TD0 1
======== CHANNEL f1 ========
SFO1 400.1424710 MHz
NUC1 1H
P1 13.55 usec
PLW1 16.00000000 W
F2 - Processing parameters
SI 65536
SF 400.1400000 MHz
WDW EM
SSB 0
LB 0.30 Hz
GB 0
PC 1.00

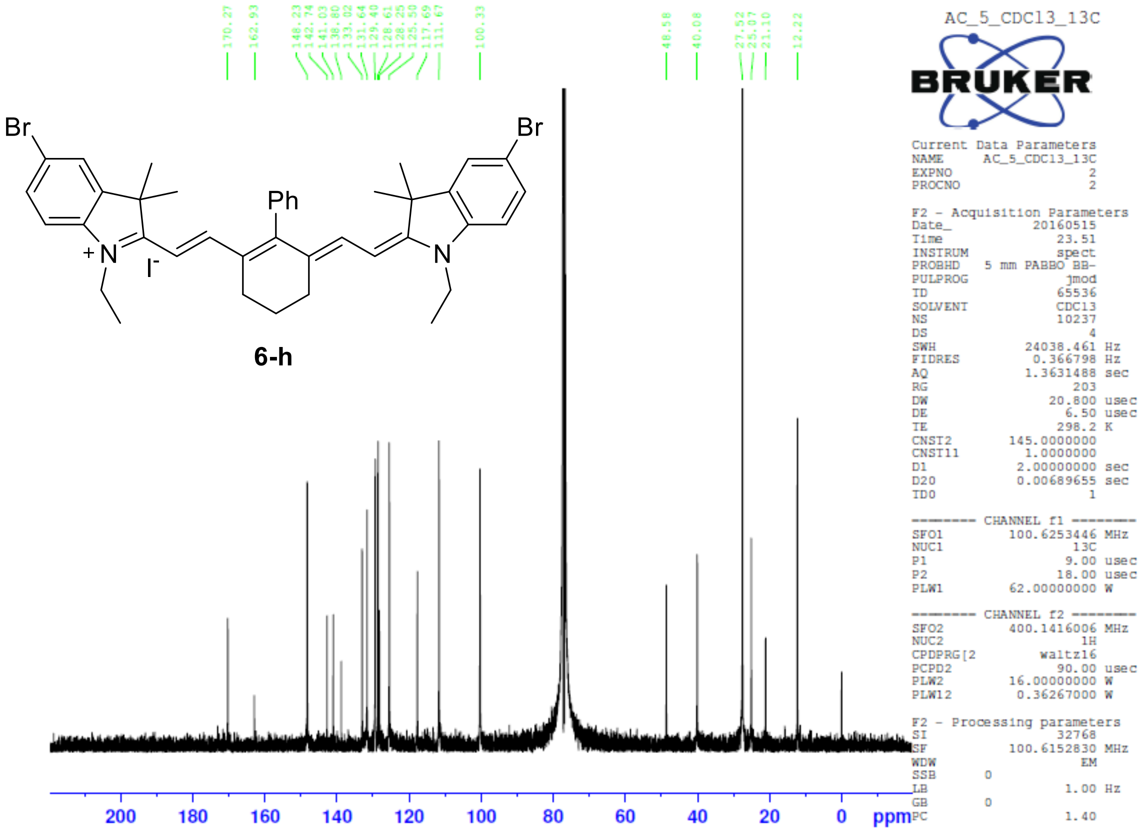
Br
Br
Ph
+N
I-
N
170.27
162.93
148.23
142.74
141.03
138.80
133.02
131.64
129.40
128.61
128.25
125.50
117.69
111.67
100.33
48.58
40.08
27.52
25.07
21.10
12.22
200
180
160
140
120
100
80
60
40
20
0
ppm
AC_5_CDC13_13C
BRUKER
Current Data Parameters
NAME AC_5_CDC13_13C
EXPNO 2
PROCNO 2
F2 - Acquisition Parameters
Date_ 20160515
Time 23.51
INSTRUM spect
PROBHD 5 mm PABBO BB-
PULPROG jmod
TD 65536
SOLVENT CDC13
NS 10237
DS 4
SWH 24038.461 Hz
FIDRES 0.366798 Hz
AQ 1.3631488 sec
RG 203
DW 20.800 usec
DE 6.50 usec
TE 298.2 K
CNST2 145.0000000
CNST11 1.0000000
D1 2.00000000 sec
D20 0.00689655 sec
TD0 1
======== CHANNEL f1 ========
SFO1 100.6253446 MHz
NUC1 13C
P1 9.00 usec
P2 18.00 usec
PLW1 62.00000000 W
======== CHANNEL f2 ========
SFO2 400.1416006 MHz
NUC2 1H
CPDPRG[2 waltz16
PCPD2 90.00 usec
PLW2 16.00000000 W
PLW12 0.36267000 W
F2 - Processing parameters
SI 32768
SF 100.6152830 MHz
WDW EM
SSB 0
LB 1.00 Hz
GB 0
PC 1.40

6-h

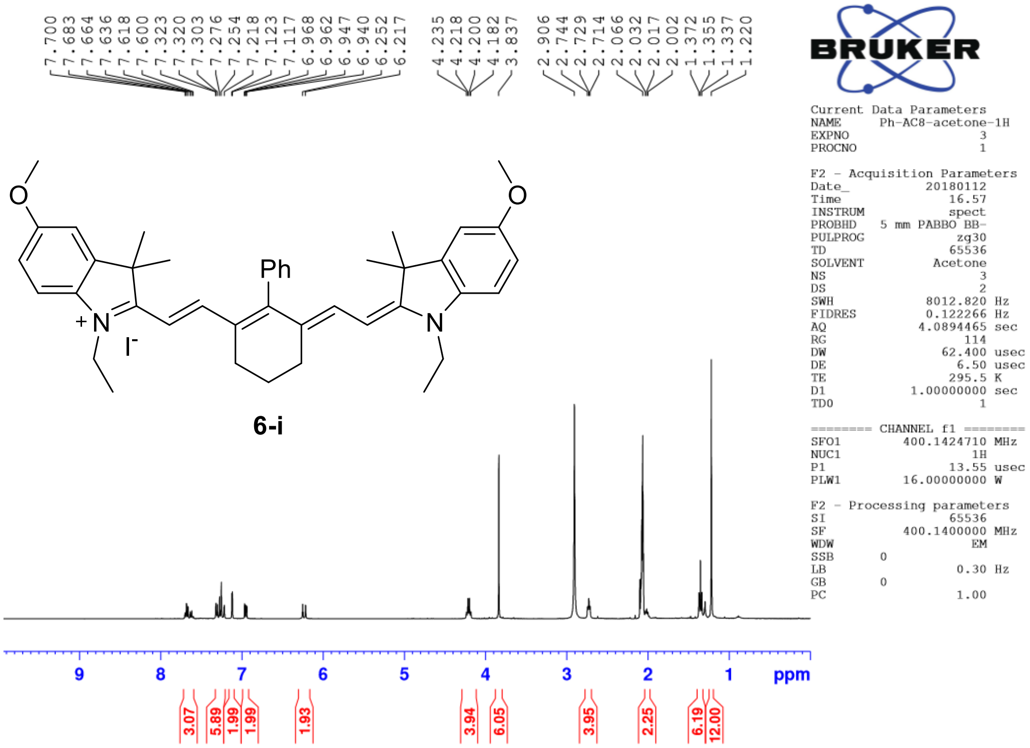

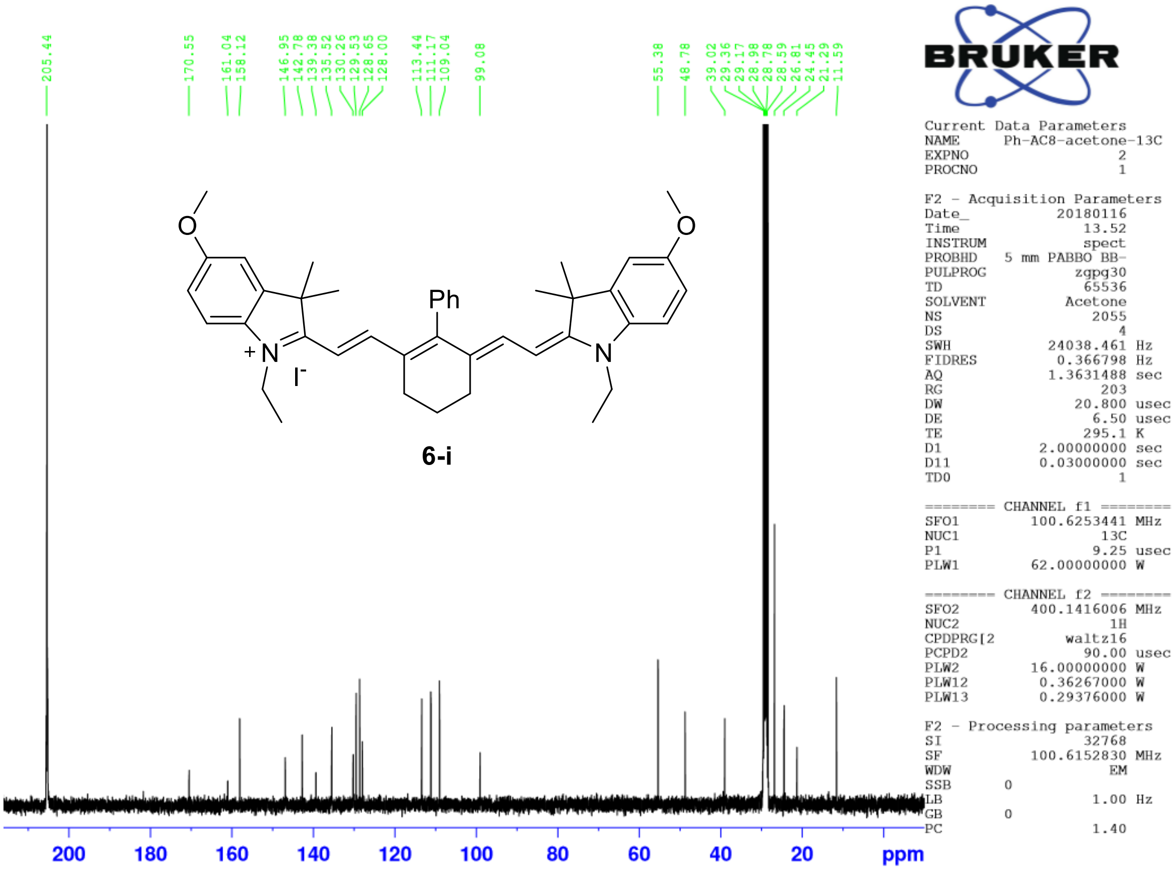

Signal seen at **δ** 205.44 ppm corresponds to the carbonyl group of acetone-*d*6

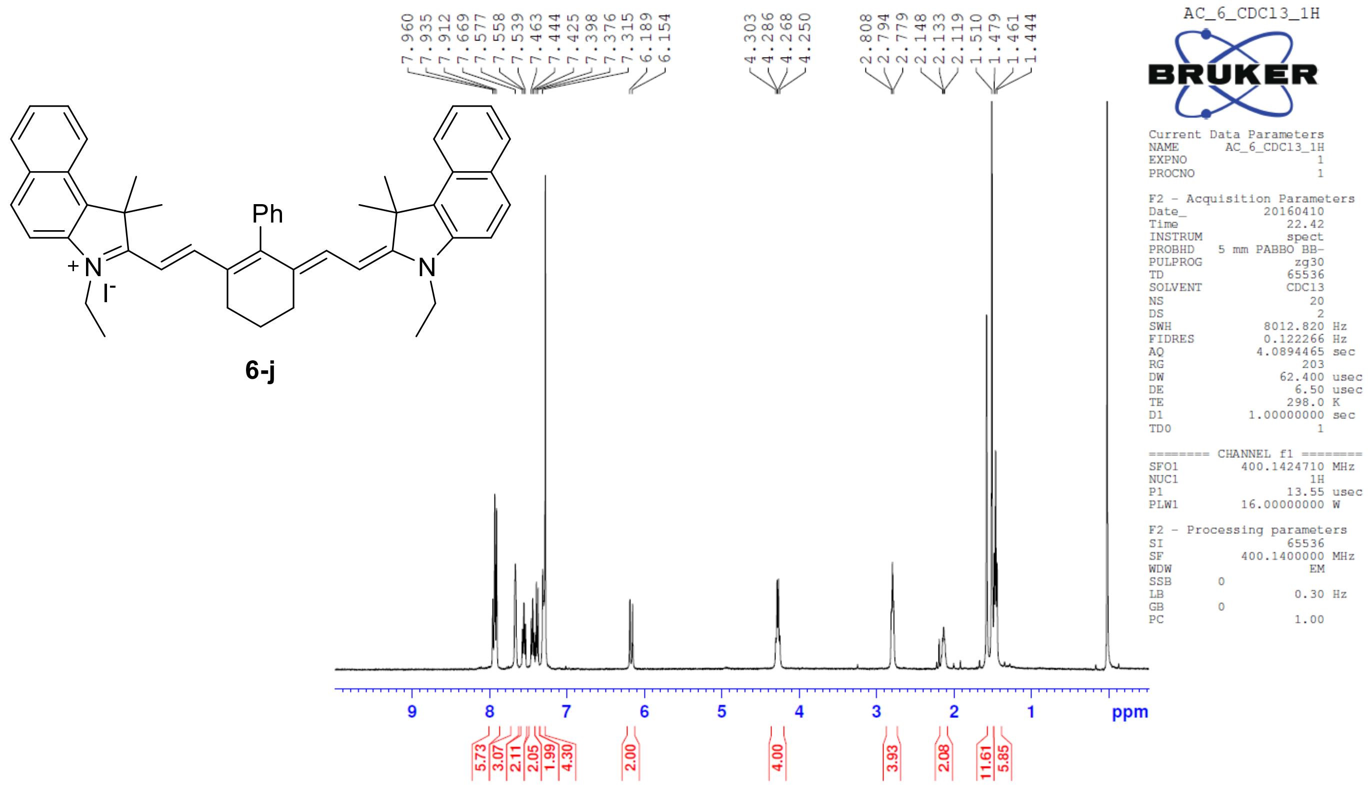
7.960
7.935
7.912
7.669
7.577
7.558
7.539
7.463
7.444
7.425
7.398
7.376
7.315
6.189
6.154
4.303
4.286
4.268
4.250
2.808
2.794
2.779
2.148
2.133
2.119
1.510
1.479
1.461
1.444
Ph
N
I-
6-j
9 8 7 6 5 4 3 2 1 ppm
5.73 3.07 2.11 2.05 1.99 4.30 2.00 4.00 3.93 2.08 11.61 5.85
AC_6_CDCl3_1H
BRUKER
Current Data Parameters
NAME AC_6_CDCl3_1H
EXPNO 1
PROCNO 1
F2 - Acquisition Parameters
Date_ 20160410
Time 22.42
INSTRUM spect
PROBHD 5 mm PABBO BB-
PULPROG zg30
TD 65536
SOLVENT CDCl3
NS 20
DS 2
SWH 8012.820 Hz
FIDRES 0.122266 Hz
AQ 4.0894465 sec
RG 203
DW 62.400 usec
DE 6.50 usec
TE 298.0 K
D1 1.00000000 sec
TD0 1
======== CHANNEL f1 ========
SFO1 400.1424710 MHz
NUC1 1H
P1 13.55 usec
PLW1 16.00000000 W
F2 - Processing parameters
SI 65536
SF 400.1400000 MHz
WDW EM
SSB 0
LB 0.30 Hz
GB 0
PC 1.00

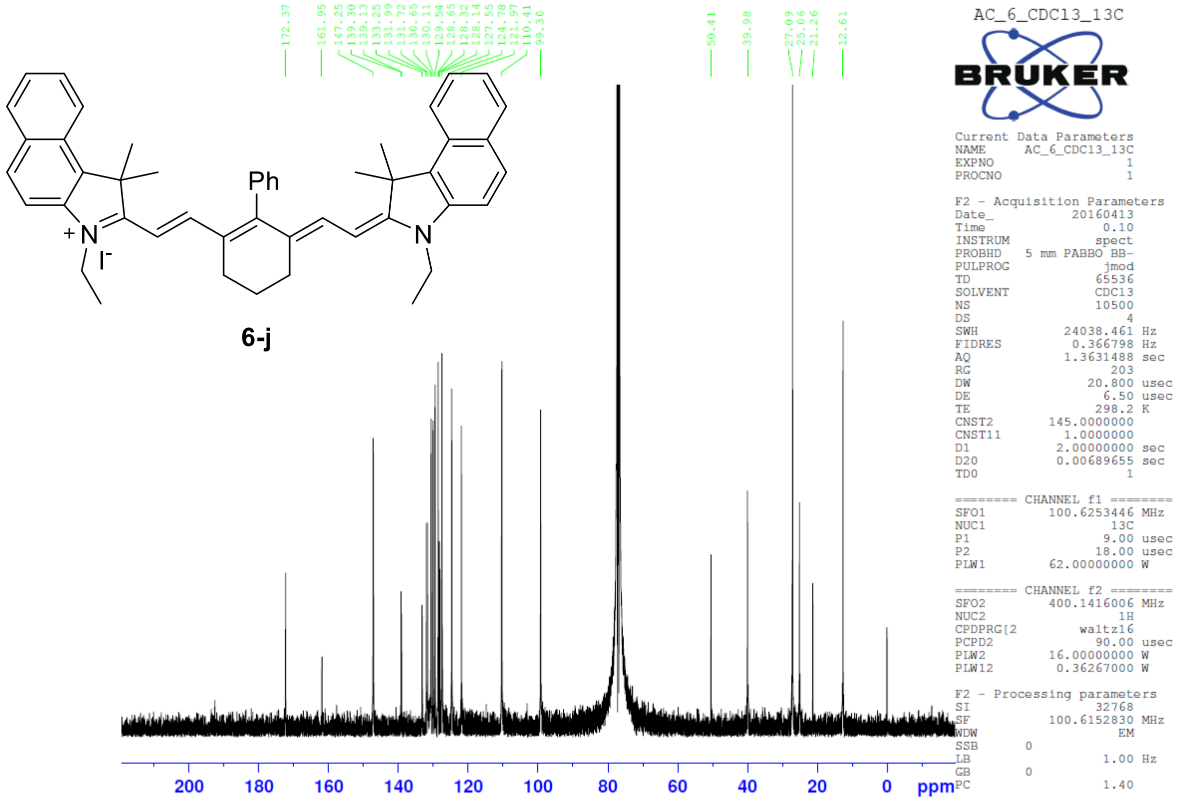
172.37
161.95
147.25
139.30
139.13
133.25
131.99
131.72
130.65
130.11
129.54
128.65
128.32
128.14
127.55
124.78
121.97
110.41
99.30
50.41
39.98
27.09
25.66
21.26
12.61
Ph
N
I-
6-j
200 180 160 140 120 100 80 60 40 20 0 ppm
AC_6_CDCl3_13C
BRUKER
Current Data Parameters
NAME AC_6_CDCl3_13C
EXPNO 1
PROCNO 1
F2 - Acquisition Parameters
Date_ 20160413
Time 0.10
INSTRUM spect
PROBHD 5 mm PABBO BB-
PULPROG jmod
TD 65536
SOLVENT CDCl3
NS 10500
DS 4
SWH 24038.461 Hz
FIDRES 0.366798 Hz
AQ 1.3631488 sec
RG 203
DW 20.800 usec
DE 6.50 usec
TE 298.2 K
CNST2 145.0000000
CNST11 1.0000000
D1 2.00000000 sec
D20 0.00689655 sec
TD0 1
======== CHANNEL f1 ========
SFO1 100.6253446 MHz
NUC1 13C
P1 9.00 usec
P2 18.00 usec
PLW1 62.00000000 W
======== CHANNEL f2 ========
SFO2 400.1416006 MHz
NUC2 1H
CPDPRG[2 waltz16
PCPD2 90.00 usec
PLW2 16.00000000 W
PLW12 0.36267000 W
F2 - Processing parameters
SI 32768
SF 100.6152830 MHz
WDW EM
SSB 0
LB 1.00 Hz
GB 0
PC 1.40

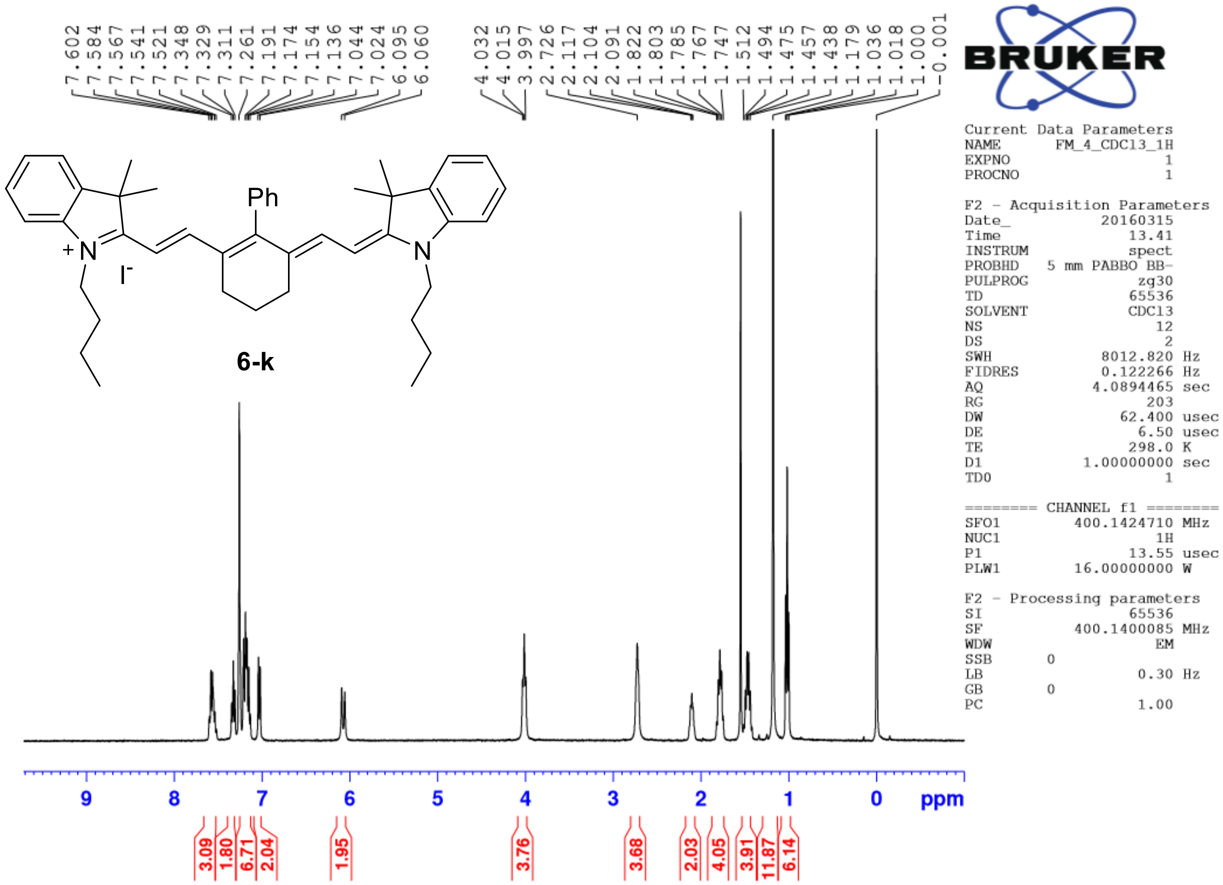
7.602 7.584 7.567 7.541 7.521 7.348 7.329 7.311 7.261 7.191 7.174 7.154 7.136 7.044 7.024 6.095 6.060
4.032 4.015 3.997 2.726 2.117 2.104 2.091 1.822 1.803 1.785 1.767 1.747 1.512 1.494 1.475 1.457 1.438 1.179 1.036 1.018 1.000 -0.001
Ph
I-
6-k
BRUKER
Current Data Parameters
NAME FM_4_CDCl3_1H
EXPNO 1
PROCNO 1
F2 - Acquisition Parameters
Date_ 20160315
Time 13.41
INSTRUM spect
PROBHD 5 mm PABBO BB-
PULPROG zg30
TD 65536
SOLVENT CDCl3
NS 12
DS 2
SWH 8012.820 Hz
FIDRES 0.122266 Hz
AQ 4.0894465 sec
RG 203
DW 62.400 usec
DE 6.50 usec
TE 298.0 K
D1 1.00000000 sec
TD0 1
======== CHANNEL f1 ========
SFO1 400.1424710 MHz
NUC1 1H
P1 13.55 usec
PLW1 16.00000000 W
F2 - Processing parameters
SI 65536
SF 400.1400085 MHz
WDW EM
SSB 0
LB 0.30 Hz
GB 0
PC 1.00
9 8 7 6 5 4 3 2 1 0 ppm
3.09 1.80 6.71 2.04 1.95 3.76 3.68 2.03 4.05 3.91 11.87 6.14

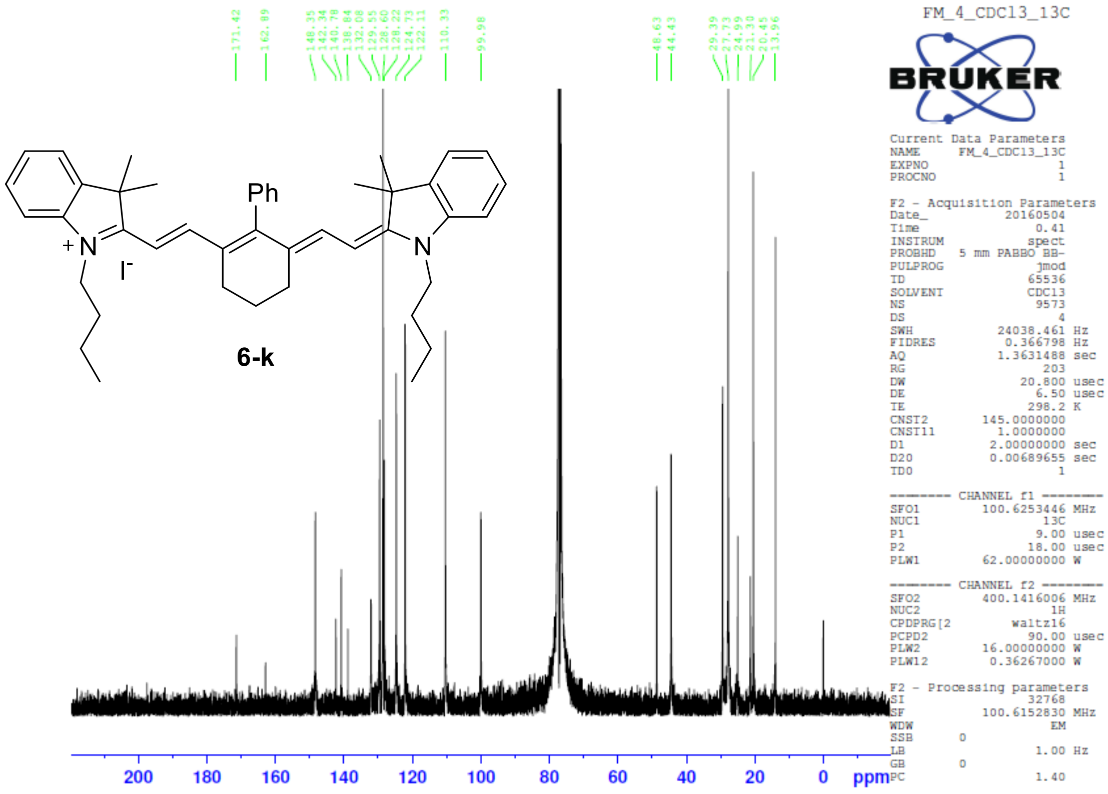
FM_4_CDCl3_13C
171.42 162.89 148.35 142.34 140.78 138.84 132.08 129.55 128.60 128.22 124.73 122.11 110.33 99.98 48.63 44.43 29.39 27.73 24.99 21.30 20.45 13.96
Ph
I-
6-k
BRUKER
Current Data Parameters
NAME FM_4_CDCl3_13C
EXPNO 1
PROCNO 1
F2 - Acquisition Parameters
Date_ 20160504
Time 0.41
INSTRUM spect
PROBHD 5 mm PABBO BB-
PULPROG jmod
TD 65536
SOLVENT CDCl3
NS 9573
DS 4
SWH 24038.461 Hz
FIDRES 0.366798 Hz
AQ 1.3631488 sec
RG 203
DW 20.800 usec
DE 6.50 usec
TE 298.2 K
CNST2 145.0000000
CNST11 1.0000000
D1 2.00000000 sec
D20 0.00689655 sec
TD0 1
======== CHANNEL f1 ========
SFO1 100.6253446 MHz
NUC1 13C
P1 9.00 usec
P2 18.00 usec
PLW1 62.00000000 W
======== CHANNEL f2 ========
SFO2 400.1416006 MHz
NUC2 1H
CPDPRG[2 waltz16
PCPD2 90.00 usec
PLW2 16.00000000 W
PLW12 0.36267000 W
F2 - Processing parameters
SI 32768
SF 100.6152830 MHz
WDW EM
SSB 0
LB 1.00 Hz
GB 0
PC 1.40
200 180 160 140 120 100 80 60 40 20 0 ppm

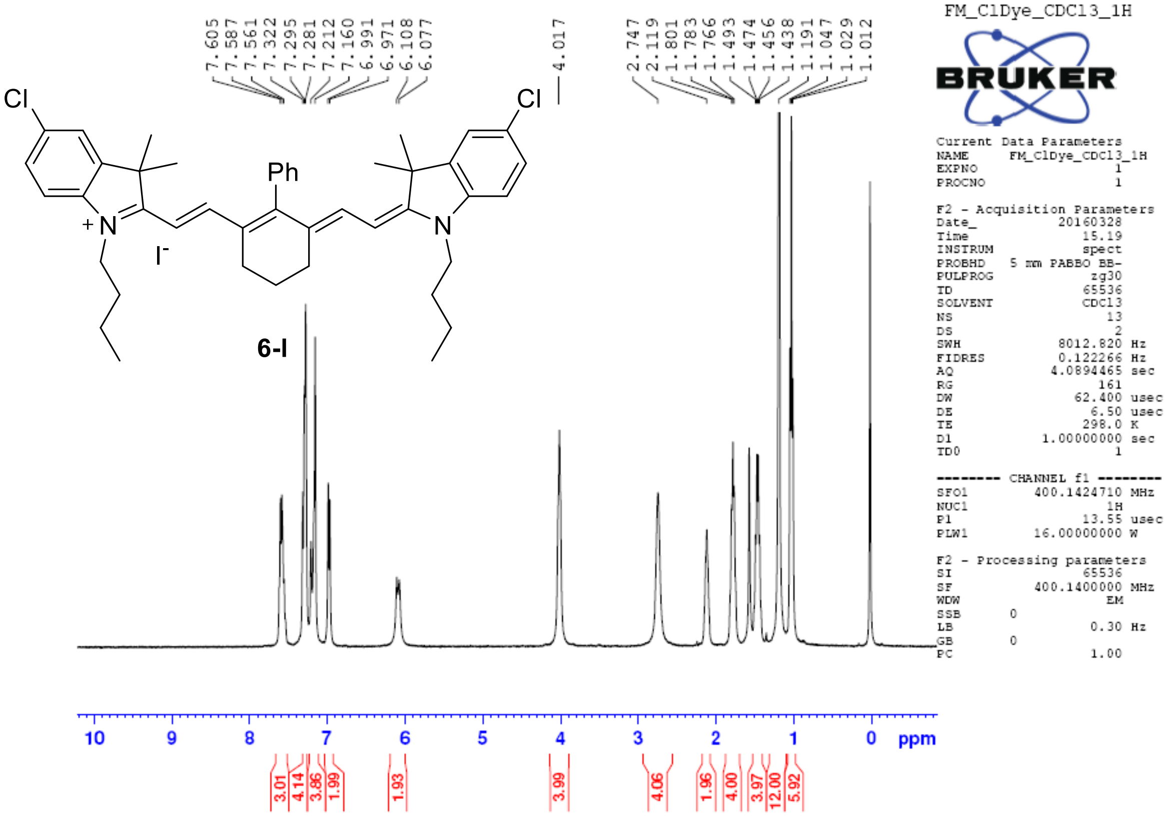
Cl
Cl
Ph
+N
N
I-
6-I
7.605
7.587
7.561
7.322
7.295
7.281
7.212
7.160
6.991
6.971
6.108
6.077
4.017
2.747
2.119
1.801
1.783
1.766
1.493
1.474
1.456
1.438
1.191
1.047
1.029
1.012
FM_ClDye_CDCl3_1H
BRUKER
Current Data Parameters
NAME FM_ClDye_CDCl3_1H
EXPNO 1
PROCNO 1
F2 - Acquisition Parameters
Date_ 20160328
Time 15.19
INSTRUM spect
PROBHD 5 mm PABBO BB-
PULPROG zg30
TD 65536
SOLVENT CDCl3
NS 13
DS 2
SWH 8012.820 Hz
FIDRES 0.122266 Hz
AQ 4.0894465 sec
RG 161
DW 62.400 usec
DE 6.50 usec
TE 298.0 K
D1 1.00000000 sec
TD0 1
======== CHANNEL f1 ========
SFO1 400.1424710 MHz
NUC1 1H
P1 13.55 usec
PLW1 16.00000000 W
F2 - Processing parameters
SI 65536
SF 400.1400000 MHz
WDW EM
SSB 0
LB 0.30 Hz
GB 0
PC 1.00
10 9 8 7 6 5 4 3 2 1 0 ppm
3.01 4.14 3.86 1.99 1.93 3.99 4.06 1.96 4.00 3.97 12.00 5.92

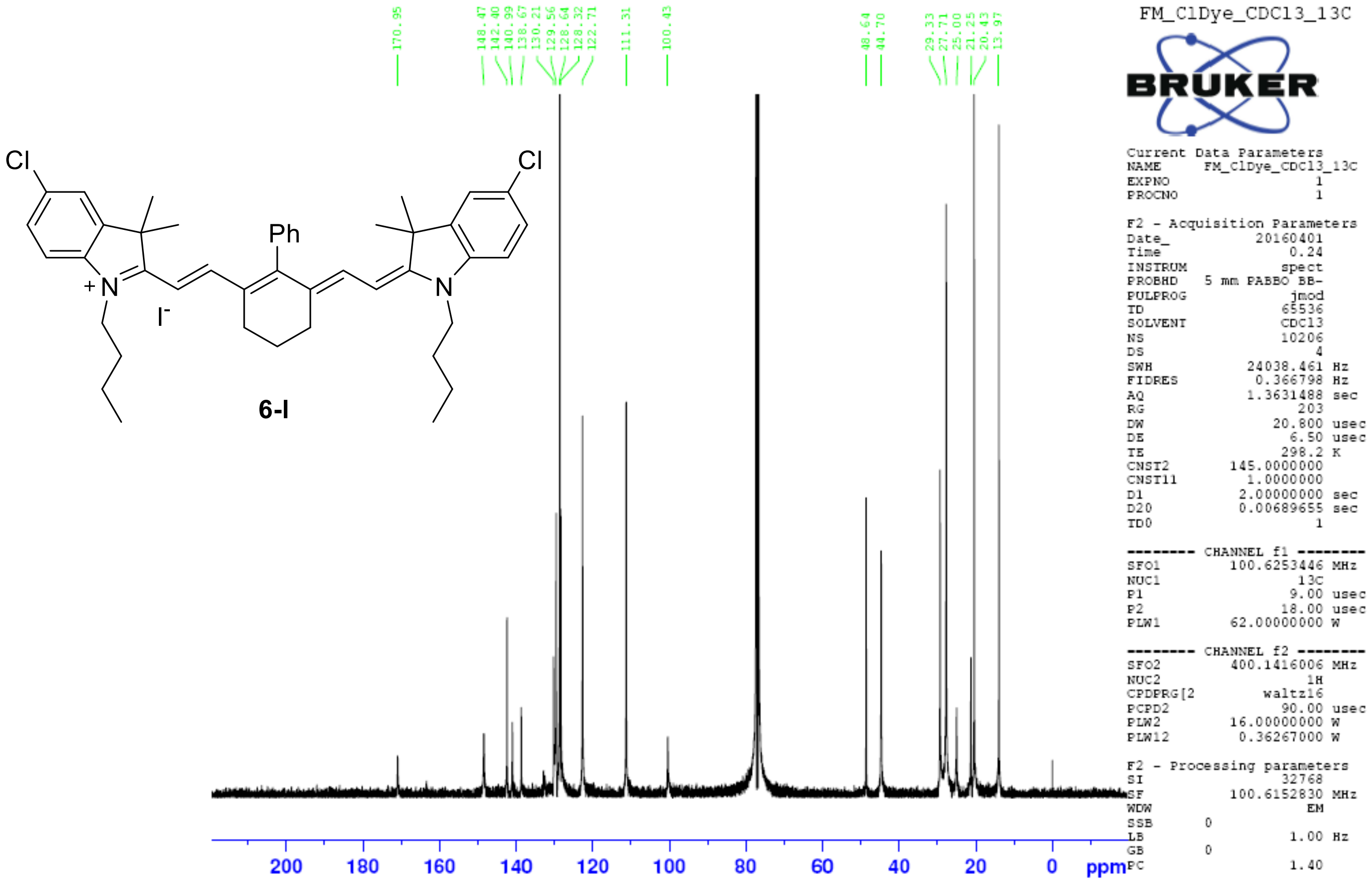
Cl
Cl
Ph
+N
N
I-
6-I
170.95
148.47
142.40
140.99
138.67
130.21
129.56
128.64
128.32
122.71
111.31
100.43
48.64
44.70
29.33
27.71
25.00
21.25
20.43
13.97
FM_ClDye_CDCl3_13C
BRUKER
Current Data Parameters
NAME FM_ClDye_CDCl3_13C
EXPNO 1
PROCNO 1
F2 - Acquisition Parameters
Date_ 20160401
Time 0.24
INSTRUM spect
PROBHD 5 mm PABBO BB-
PULPROG jmod
TD 65536
SOLVENT CDCl3
NS 10206
DS 4
SWH 24038.461 Hz
FIDRES 0.366798 Hz
AQ 1.3631488 sec
RG 203
DW 20.800 usec
DE 6.50 usec
TE 298.2 K
CNST2 145.0000000
CNST11 1.0000000
D1 2.00000000 sec
D20 0.00689655 sec
TD0 1
======== CHANNEL f1 ========
SFO1 100.6253446 MHz
NUC1 13C
P1 9.00 usec
P2 18.00 usec
PLW1 62.00000000 W
======== CHANNEL f2 ========
SFO2 400.1416006 MHz
NUC2 1H
CPDPRG[2 waltz16
PCPD2 90.00 usec
PLW2 16.00000000 W
PLW12 0.36267000 W
F2 - Processing parameters
SI 32768
SF 100.6152830 MHz
WDW EM
SSB 0
LB 1.00 Hz
GB 0
PC 1.40
200 180 160 140 120 100 80 60 40 20 0 ppm

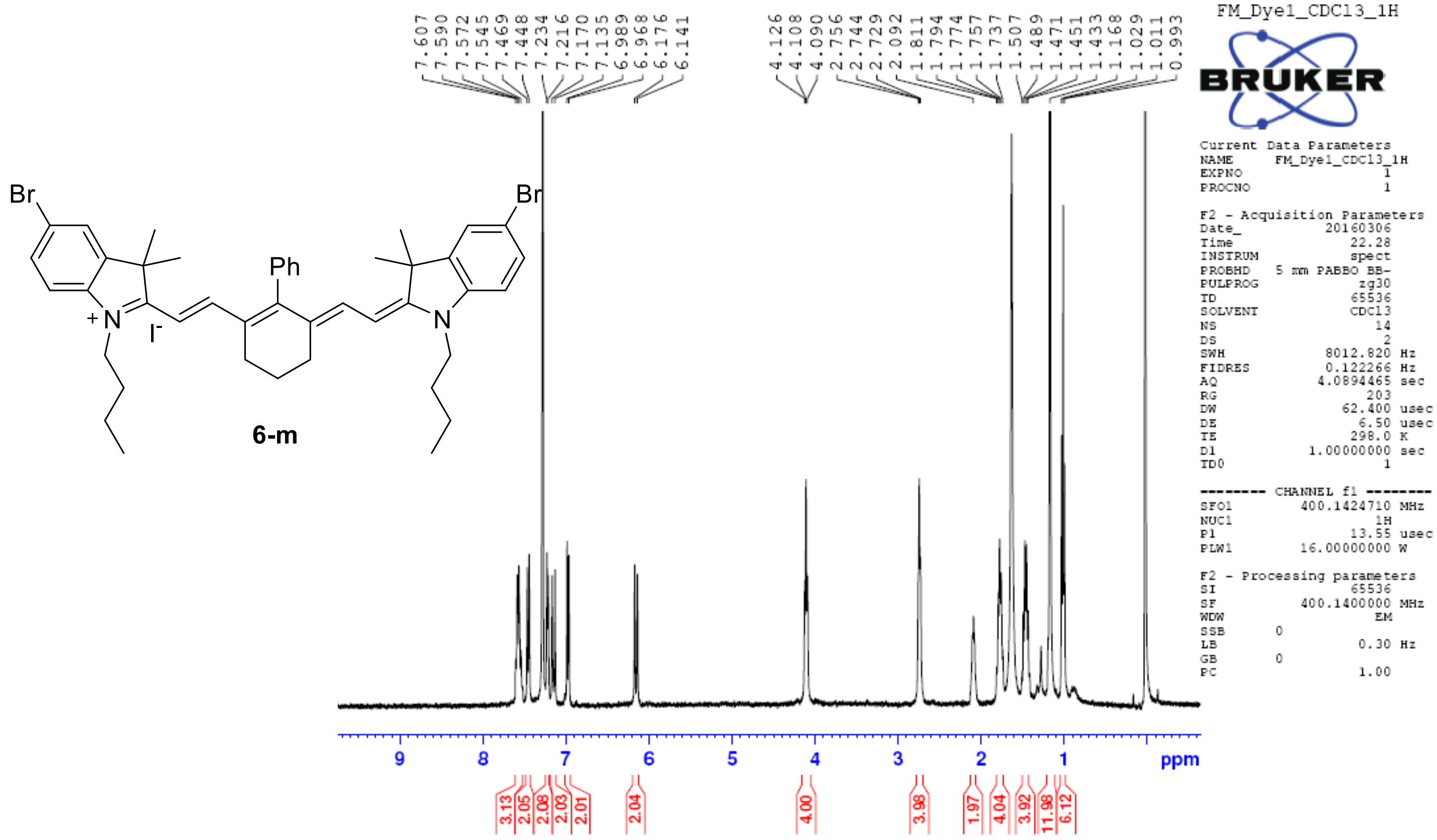

7.607 7.590 7.572 7.545 7.469 7.448 7.234 7.216 7.170 7.135 6.989 6.968 6.176 6.141
4.126 4.108 4.090 2.756 2.744 2.729 2.092 1.811 1.794 1.774 1.757 1.737 1.507 1.489 1.471 1.451 1.433 1.168 1.029 1.011 0.993
Br
Br
Ph
N
N
I-
6-m
3.13 2.05 2.08 2.03 2.01 2.04 4.00 3.98 1.97 4.04 3.92 11.98 6.12
9 8 7 6 5 4 3 2 1 ppm
FM_Dye1_CDCl3_1H
Current Data Parameters
NAME FM_Dye1_CDCl3_1H
EXPNO 1
PROCNO 1
F2 - Acquisition Parameters
Date_ 20160306
Time 22.28
INSTRUM spect
PROBHD 5 mm PABBO BB-
PULPROG zg30
TD 65536
SOLVENT CDCl3
NS 14
DS 2
SWH 8012.820 Hz
FIDRES 0.122266 Hz
AQ 4.0894465 sec
RG 203
DW 62.400 usec
DE 6.50 usec
TE 298.0 K
D1 1.00000000 sec
TD0 1
-------- CHANNEL f1 --------
SFO1 400.1424710 MHz
NUC1 1H
P1 13.55 usec
PLW1 16.00000000 W
F2 - Processing parameters
SI 65536
SF 400.1400000 MHz
WDW EM
SSB 0
LB 0.30 Hz
GB 0
PC 1.00

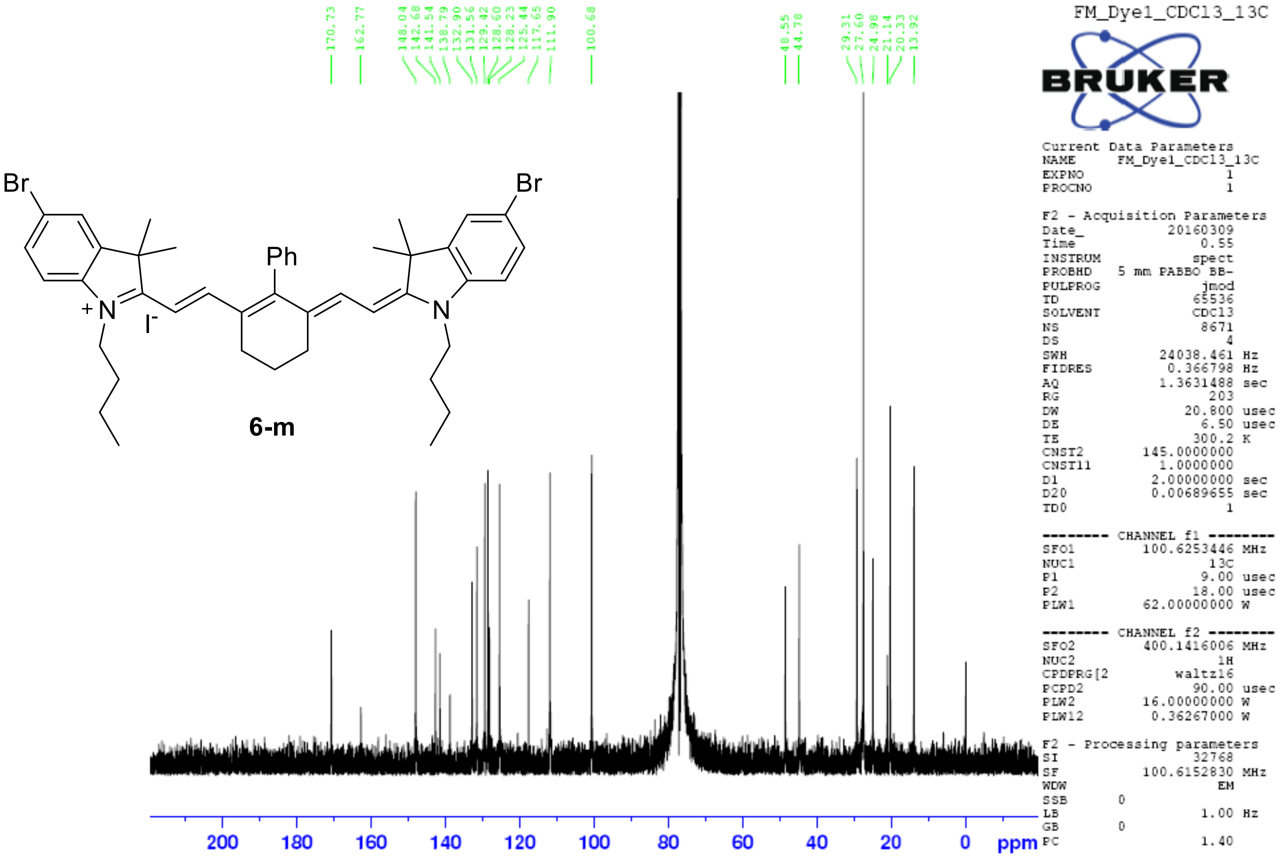

170.73 162.77 148.04 142.68 141.54 138.79 132.90 131.56 129.42 128.60 128.23 125.44 117.65 111.90 100.68
48.55 44.78 29.31 27.60 24.98 21.14 20.33 13.92
Br
Br
Ph
N
N
I-
6-m
200 180 160 140 120 100 80 60 40 20 0 ppm
FM_Dye1_CDCl3_13C
Current Data Parameters
NAME FM_Dye1_CDCl3_13C
EXPNO 1
PROCNO 1
F2 - Acquisition Parameters
Date_ 20160309
Time 0.55
INSTRUM spect
PROBHD 5 mm PABBO BB-
PULPROG jmod
TD 65536
SOLVENT CDCl3
NS 8671
DS 4
SWH 24038.461 Hz
FIDRES 0.366798 Hz
AQ 1.3631488 sec
RG 203
DW 20.800 usec
DE 6.50 usec
TE 300.2 K
CNST2 145.0000000
CNST11 1.0000000
D1 2.00000000 sec
D20 0.00689655 sec
TD0 1
-------- CHANNEL f1 --------
SFO1 100.6253446 MHz
NUC1 13C
P1 9.00 usec
P2 18.00 usec
PLW1 62.00000000 W
-------- CHANNEL f2 --------
SFO2 400.1416006 MHz
NUC2 1H
CPDPRG[2 waltz16
PCPD2 90.00 usec
PLW2 16.00000000 W
PLW12 0.36267000 W
F2 - Processing parameters
SI 32768
SF 100.6152830 MHz
WDW EM
SSB 0
LB 1.00 Hz
GB 0
PC 1.40

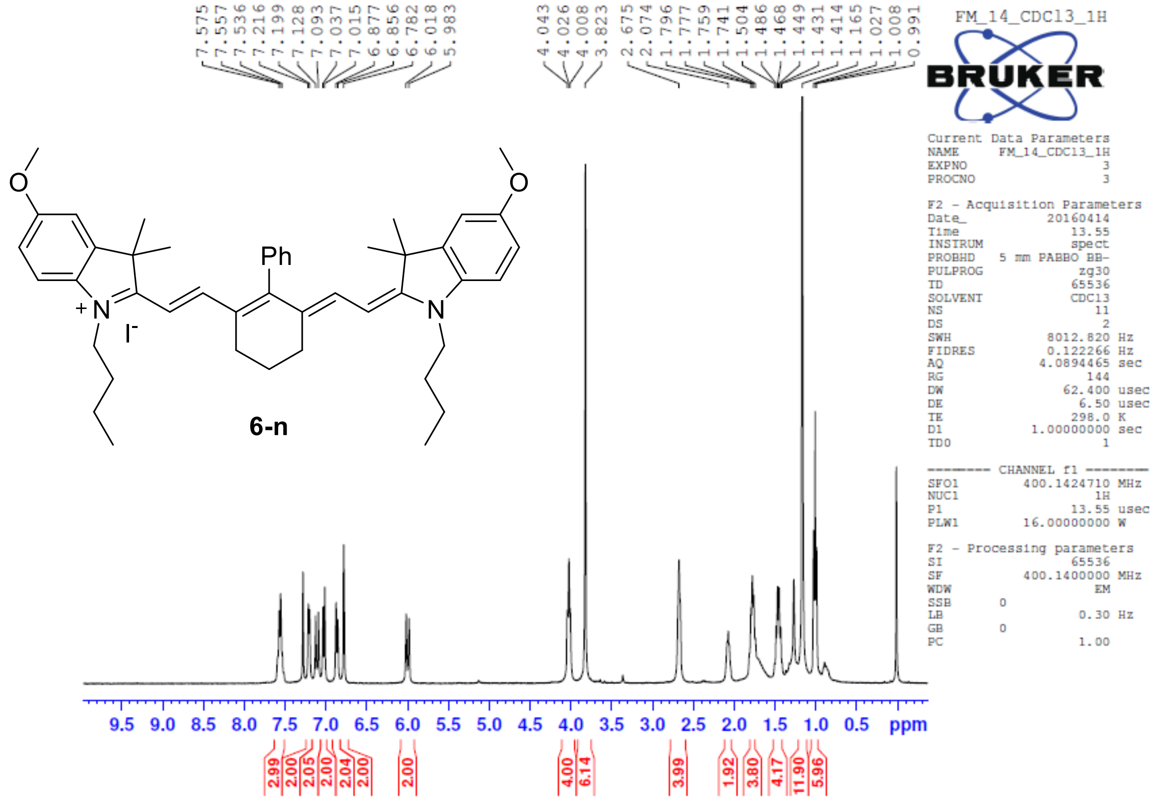

6-n
Ph
FM_14_CDC13_1H
BRUKER
Current Data Parameters
NAME FM_14_CDC13_1H
EXPNO 3
PROCNO 3
F2 - Acquisition Parameters
Date_ 20160414
Time 13.55
INSTRUM spect
PROBHD 5 mm PABBO BB-
PULPROG zg30
TD 65536
SOLVENT CDC13
NS 11
DS 2
SWH 8012.820 Hz
FIDRES 0.122266 Hz
AQ 4.0894465 sec
RG 144
DW 62.400 usec
DE 6.50 usec
TE 298.0 K
D1 1.00000000 sec
TD0 1
======== CHANNEL f1 ========
SFO1 400.1424710 MHz
NUC1 1H
P1 13.55 usec
PLW1 16.00000000 W
F2 - Processing parameters
SI 65536
SF 400.1400000 MHz
WDW EM
SSB 0
LB 0.30 Hz
GB 0
PC 1.00
ppm

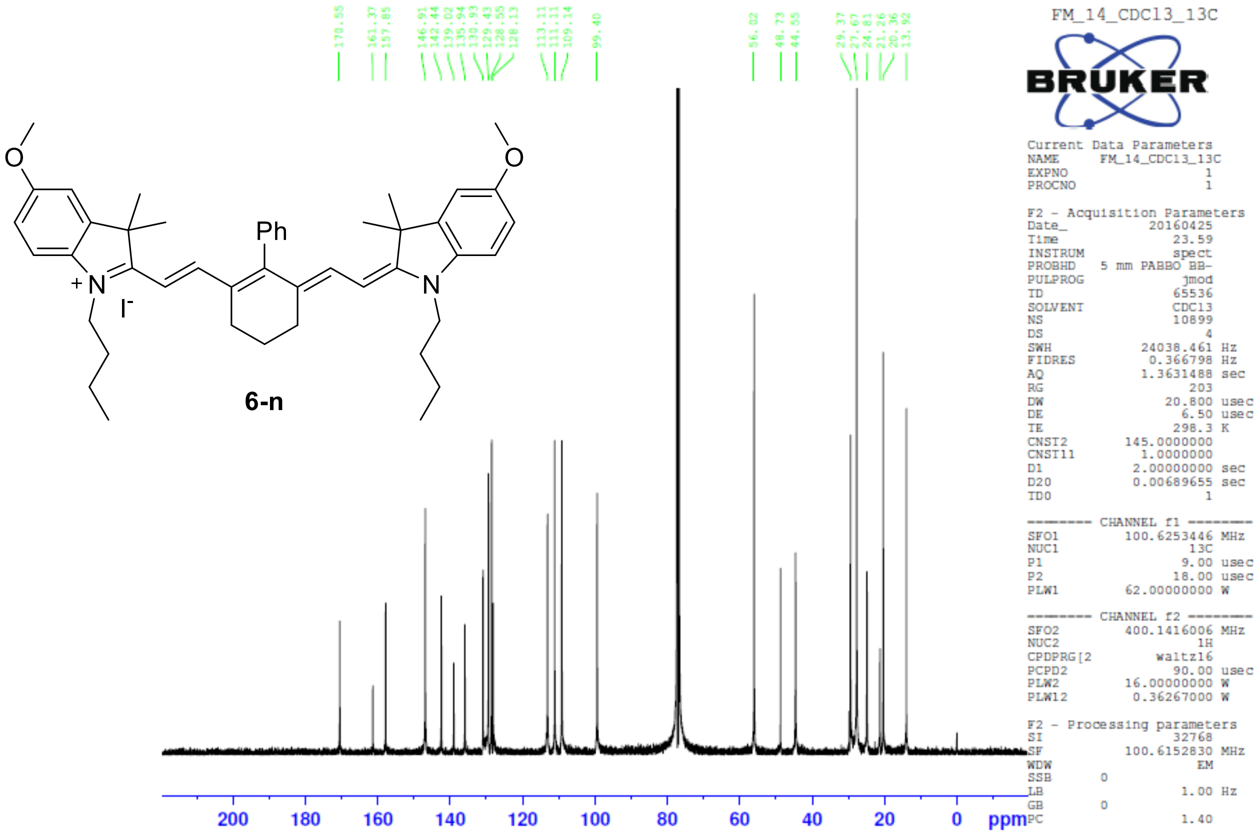

6-n
Ph
FM_14_CDC13_13C
BRUKER
Current Data Parameters
NAME FM_14_CDC13_13C
EXPNO 1
PROCNO 1
F2 - Acquisition Parameters
Date_ 20160425
Time 23.59
INSTRUM spect
PROBHD 5 mm PABBO BB-
PULPROG jmod
TD 65536
SOLVENT CDC13
NS 10899
DS 4
SWH 24038.461 Hz
FIDRES 0.366798 Hz
AQ 1.3631488 sec
RG 203
DW 20.800 usec
DE 6.50 usec
TE 298.3 K
CNST2 145.0000000
CNST11 1.0000000
D1 2.00000000 sec
D20 0.00689655 sec
TD0 1
======== CHANNEL f1 ========
SFO1 100.6253446 MHz
NUC1 13C
P1 9.00 usec
P2 18.00 usec
PLW1 62.00000000 W
======== CHANNEL f2 ========
SFO2 400.1416006 MHz
NUC2 1H
CPDPRG[2 waltz16
PCPD2 90.00 usec
PLW2 16.00000000 W
PLW12 0.36267000 W
F2 - Processing parameters
SI 32768
SF 100.6152830 MHz
WDW EM
SSB 0
LB 1.00 Hz
GB 0
PC 1.40
ppm

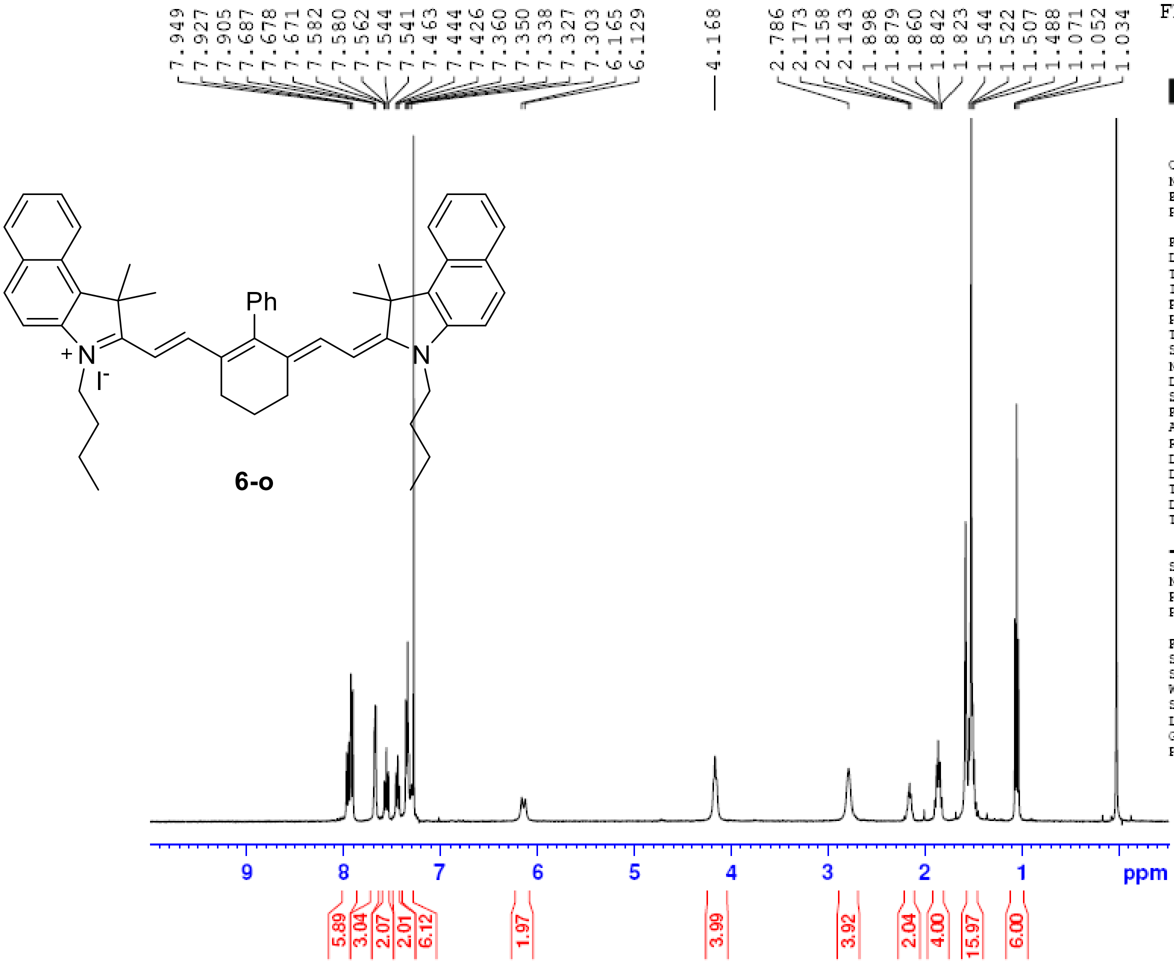

6-o

FM_BenzEDye_CDCl3_1H

```
Current Data Parameters
NAME      FM_BenzEDye_CDCl3_1H
EXPNO                    1
PROCNO                   1

F2 - Acquisition Parameters
Date_             20160401
Time                 16.14
INSTRUM              spect
PROBHD    5 mm PABBO BB-
PULPROG               zg30
TD                   65536
SOLVENT              CDCl3
NS                      15
DS                       2
SWH               8012.820 Hz
FIDRES            0.122266 Hz
AQ               4.0894465 sec
RG                     203
DW                  62.400 usec
DE                    6.50 usec
TE                   298.0 K
D1             1.00000000 sec
TD0                      1

======== CHANNEL f1 ========
SFO1           400.1424710 MHz
NUC1                    1H
P1                   13.55 usec
PLW1           16.00000000 W

F2 - Processing parameters
SI                   65536
SF             400.1400000 MHz
WDW                     EM
SSB       0
LB                    0.30 Hz
GB        0
PC                    1.00
```

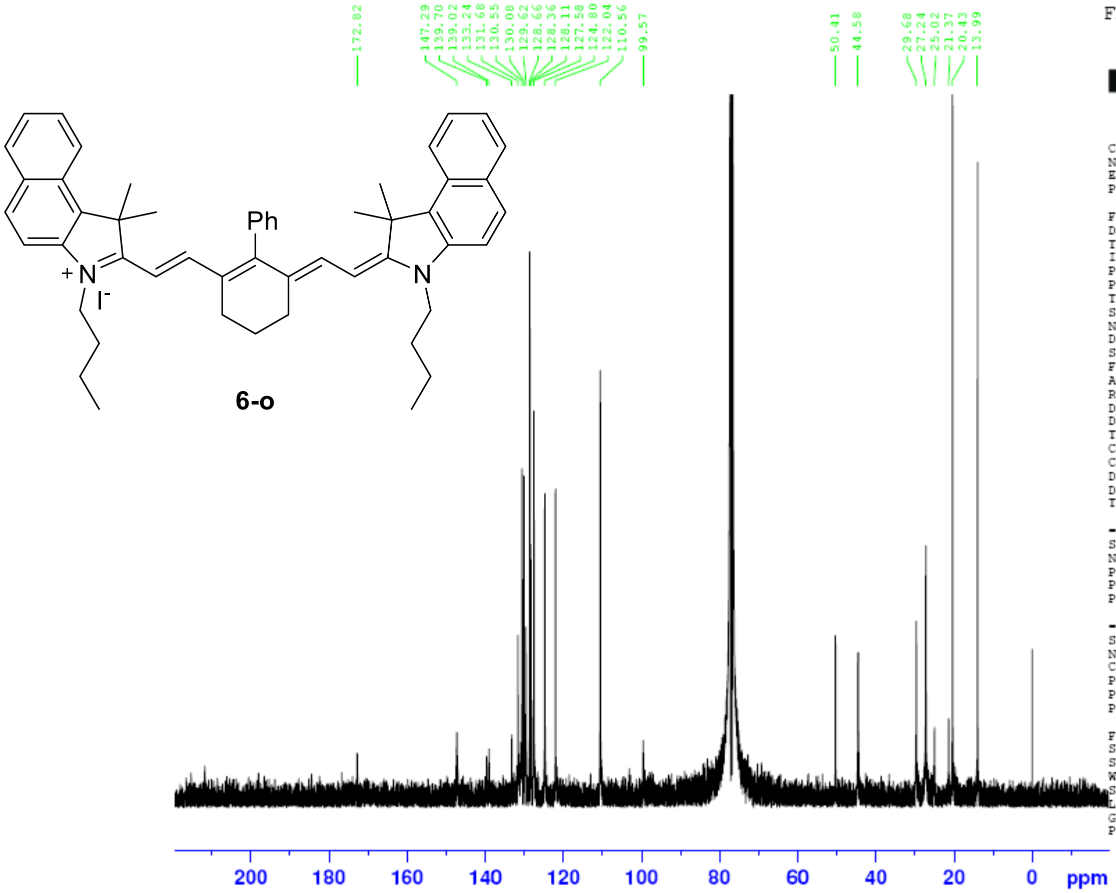

6-o

FM_BenzEDye_CDCl3_13C

```
Current Data Parameters
NAME      FM_BenzEDye_CDCl3_13C
EXPNO                    1
PROCNO                   1

F2 - Acquisition Parameters
Date_             20160402
Time                  2.09
INSTRUM              spect
PROBHD    5 mm PABBO BB-
PULPROG               jmod
TD                   65536
SOLVENT              CDCl3
NS                    8831
DS                       4
SWH              24038.461 Hz
FIDRES            0.366798 Hz
AQ               1.3631488 sec
RG                     203
DW                  20.800 usec
DE                    6.50 usec
TE                   298.2 K
CNST2          145.0000000
CNST11           1.0000000
D1              2.00000000 sec
D20             0.00689655 sec
TD0                      1

======== CHANNEL f1 ========
SFO1           100.6253446 MHz
NUC1                   13C
P1                    9.00 usec
P2                   18.00 usec
PLW1           62.00000000 W

======== CHANNEL f2 ========
SFO2           400.1416006 MHz
NUC2                    1H
CPDPRG[2            waltz16
PCPD2                90.00 usec
PLW2           16.00000000 W
PLW12           0.36267000 W

F2 - Processing parameters
SI                   32768
SF             100.6152830 MHz
WDW                     EM
SSB       0
LB                    1.00 Hz
GB        0
PC                    1.40
```

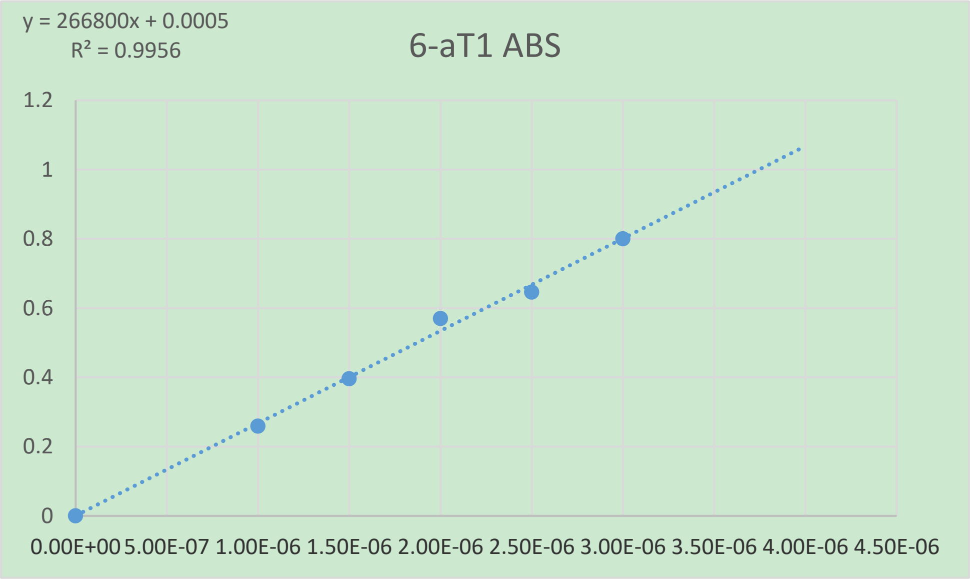
y = 266800x + 0.0005
R² = 0.9956
6-aT1 ABS
1.2
1
0.8
0.6
0.4
0.2
0
0.00E+00 5.00E-07 1.00E-06 1.50E-06 2.00E-06 2.50E-06 3.00E-06 3.50E-06 4.00E-06 4.50E-06

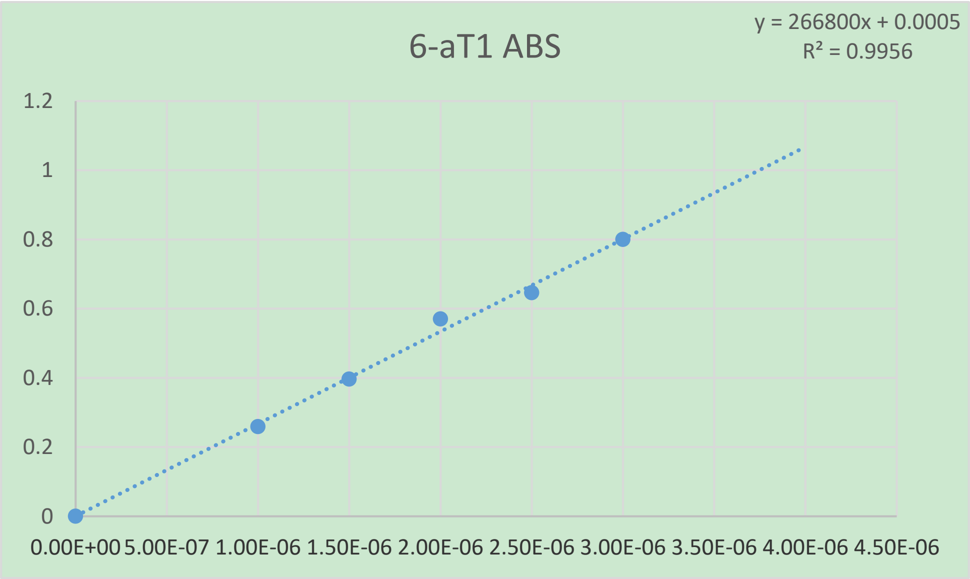
6-aT1 ABS
y = 266800x + 0.0005
R² = 0.9956
1.2
1
0.8
0.6
0.4
0.2
0
0.00E+00 5.00E-07 1.00E-06 1.50E-06 2.00E-06 2.50E-06 3.00E-06 3.50E-06 4.00E-06 4.50E-06

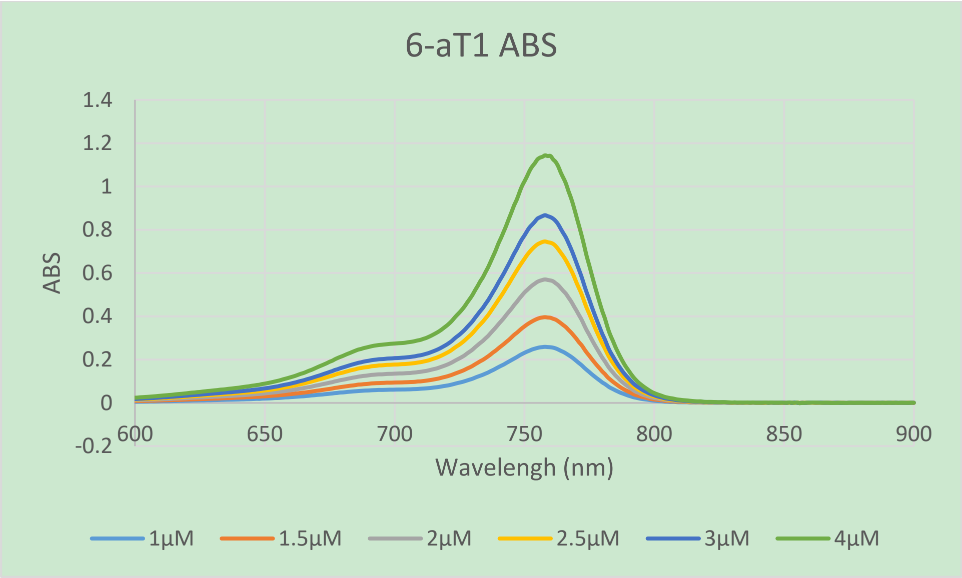
6-aT1 ABS
ABS
1.4
1.2
1
0.8
0.6
0.4
0.2
0
-0.2
600
650
700
750
800
850
900
Wavelengh (nm)
1µM
1.5µM
2µM
2.5µM
3µM
4µM

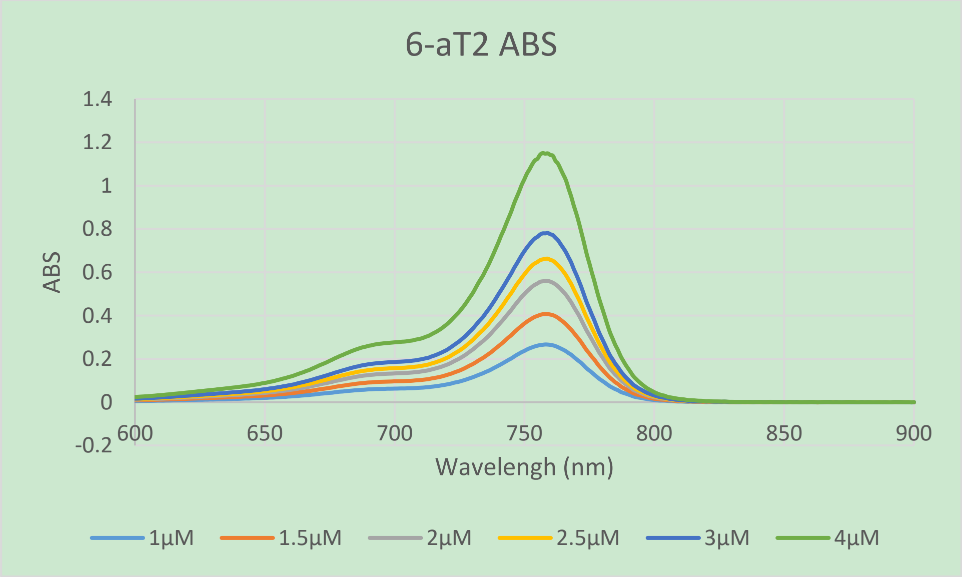
6-aT2 ABS
ABS
1.4
1.2
1
0.8
0.6
0.4
0.2
0
-0.2
600
650
700
750
800
850
900
Wavelengh (nm)
1µM
1.5µM
2µM
2.5µM
3µM
4µM

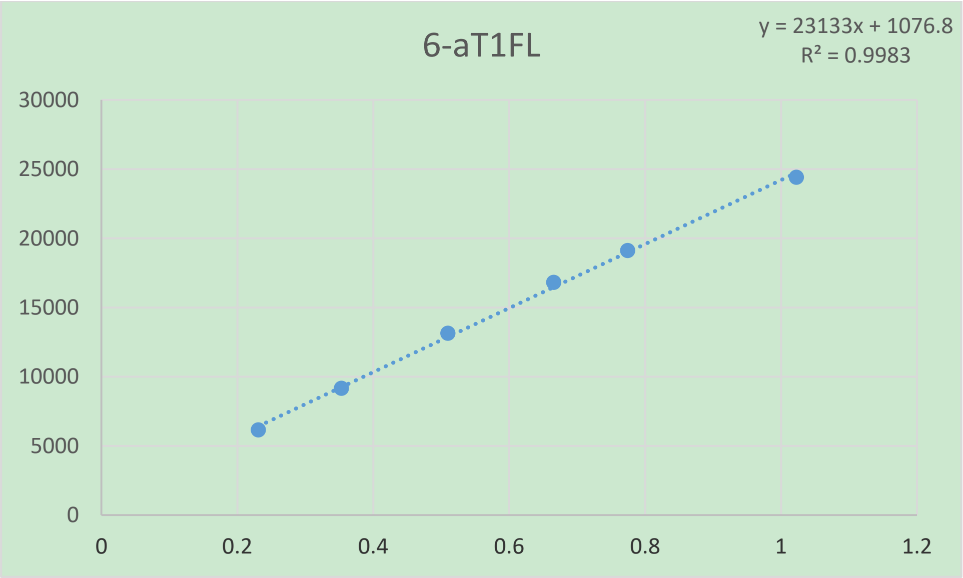
6-aT1FL
y = 23133x + 1076.8
R² = 0.9983
30000
25000
20000
15000
10000
5000
0
0
0.2
0.4
0.6
0.8
1
1.2

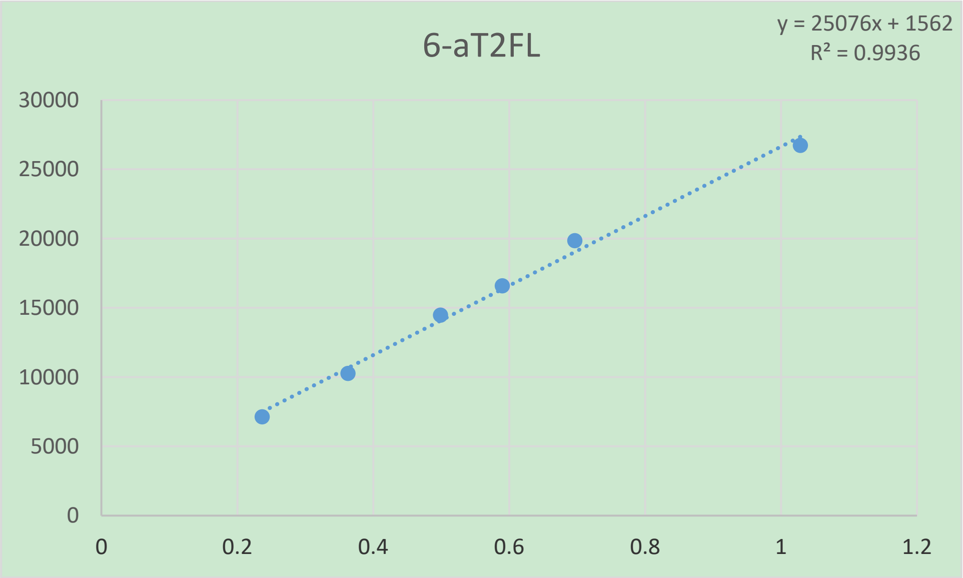
6-aT2FL
y = 25076x + 1562
R² = 0.9936
30000
25000
20000
15000
10000
5000
0
0
0.2
0.4
0.6
0.8
1
1.2

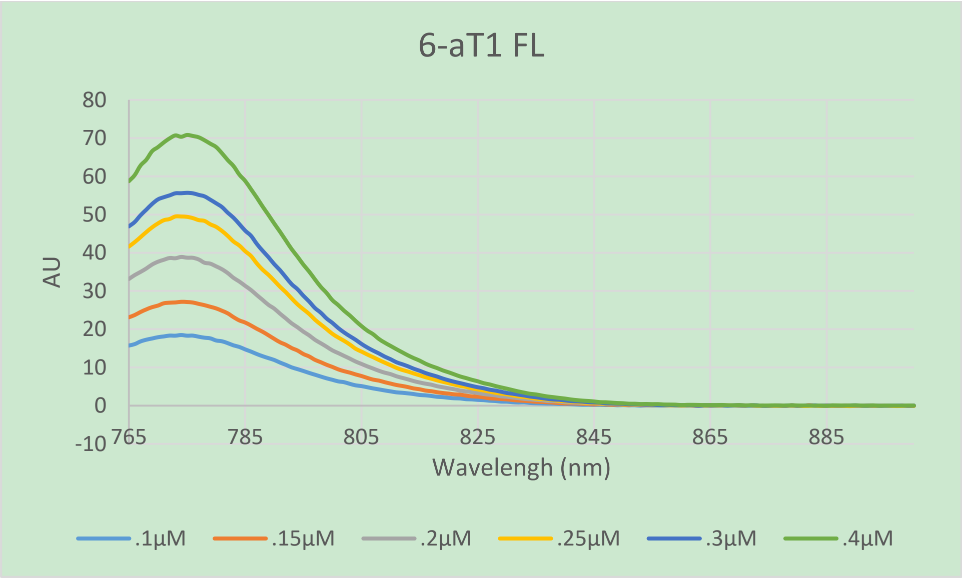
6-aT1 FL
AU
80
70
60
50
40
30
20
10
0
-10
765
785
805
825
845
865
885
Wavelengh (nm)
.1μM
.15μM
.2μM
.25μM
.3μM
.4μM

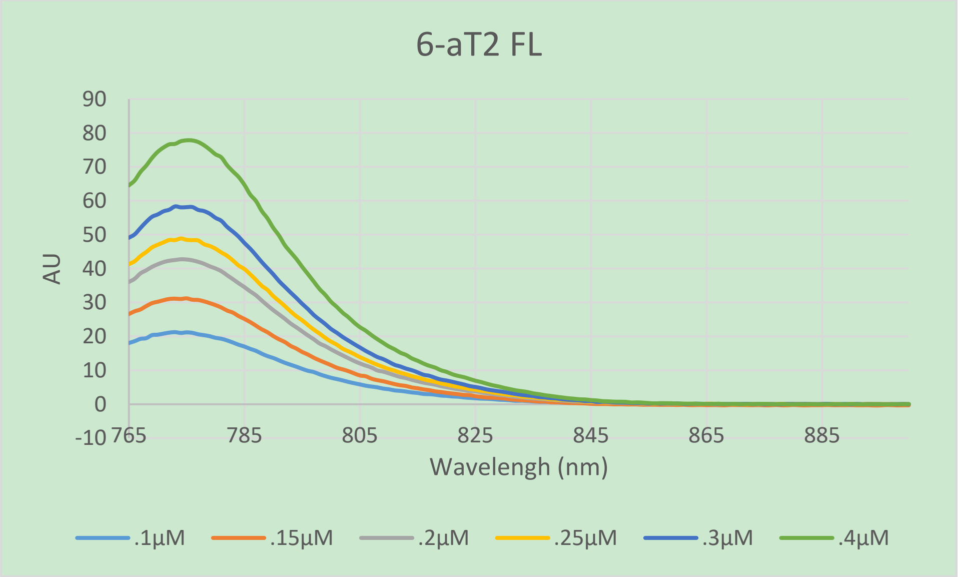
6-aT2 FL
AU
90
80
70
60
50
40
30
20
10
0
-10
765
785
805
825
845
865
885
Wavelengh (nm)
.1μM
.15μM
.2μM
.25μM
.3μM
.4μM

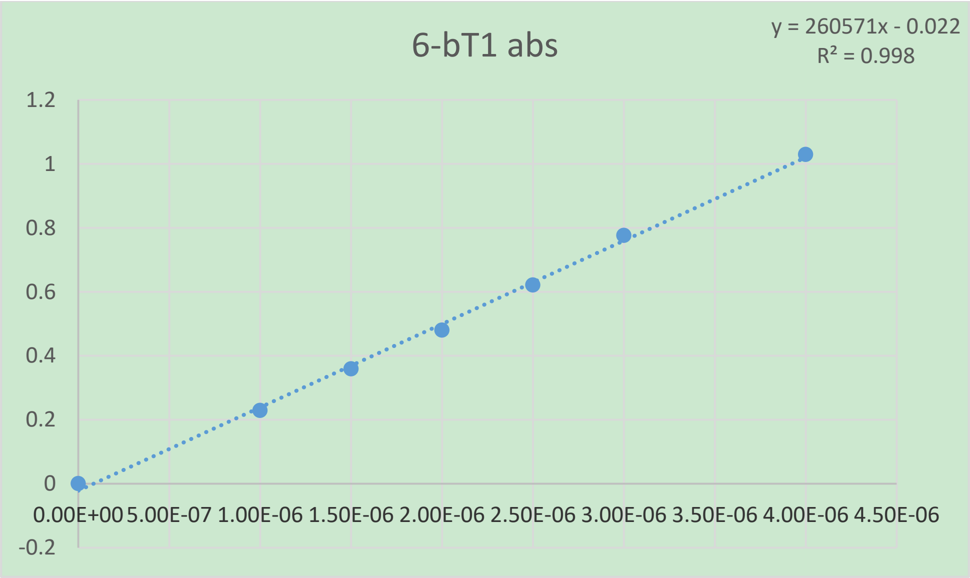
6-bT1 abs
y = 260571x - 0.022
R² = 0.998
1.2
1
0.8
0.6
0.4
0.2
0
-0.2
0.00E+00 5.00E-07 1.00E-06 1.50E-06 2.00E-06 2.50E-06 3.00E-06 3.50E-06 4.00E-06 4.50E-06

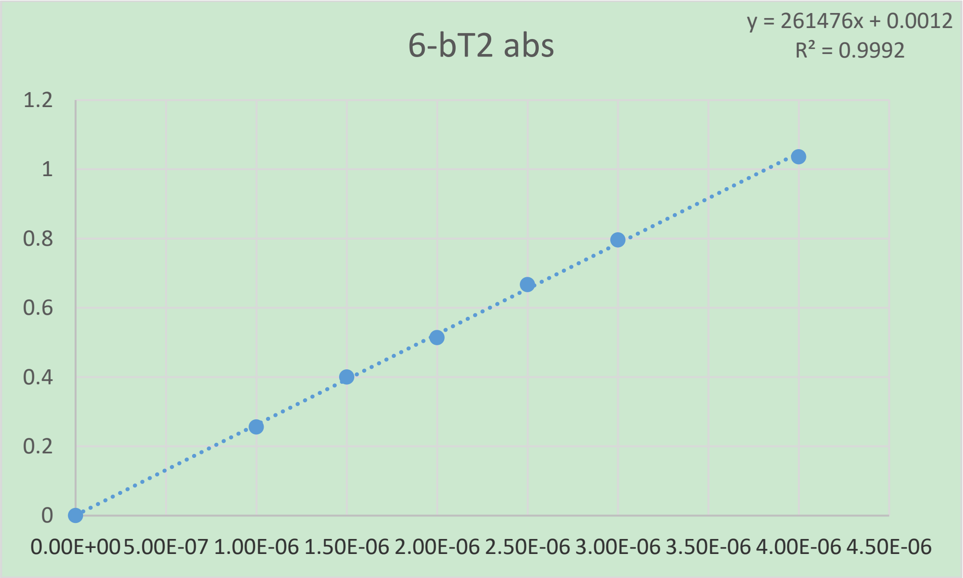
6-bT2 abs
y = 261476x + 0.0012
R² = 0.9992
1.2
1
0.8
0.6
0.4
0.2
0
0.00E+00 5.00E-07 1.00E-06 1.50E-06 2.00E-06 2.50E-06 3.00E-06 3.50E-06 4.00E-06 4.50E-06

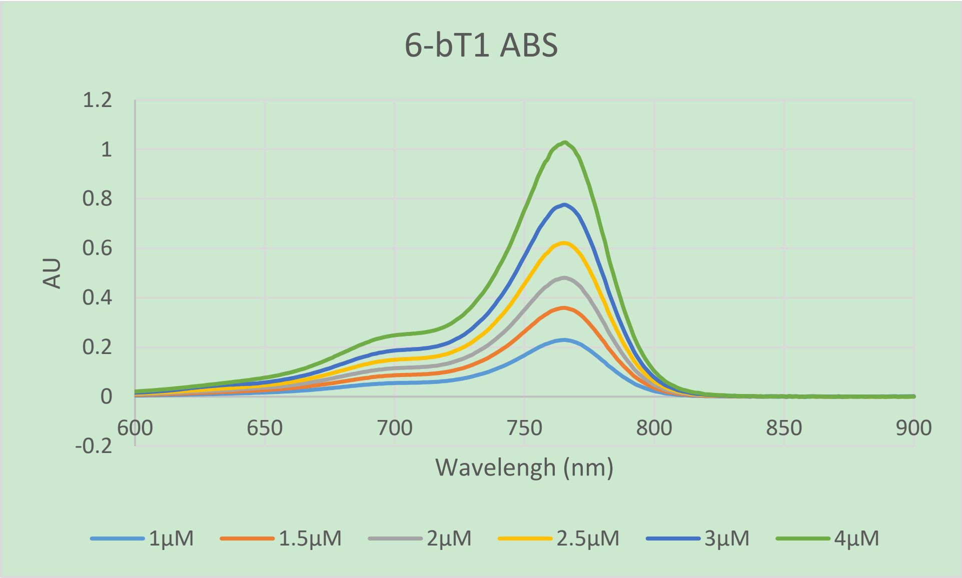
6-bT1 ABS
AU
1.2
1
0.8
0.6
0.4
0.2
0
-0.2
600
650
700
750
800
850
900
Wavelengh (nm)
1µM
1.5µM
2µM
2.5µM
3µM
4µM

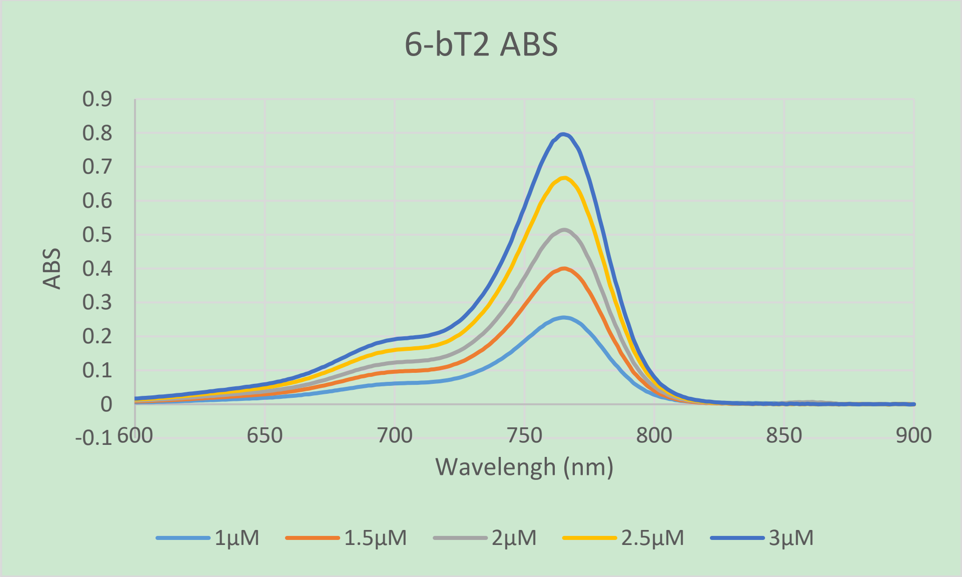
6-bT2 ABS
ABS
0.9
0.8
0.7
0.6
0.5
0.4
0.3
0.2
0.1
0
-0.1
600
650
700
750
800
850
900
Wavelengh (nm)
1µM
1.5µM
2µM
2.5µM
3µM

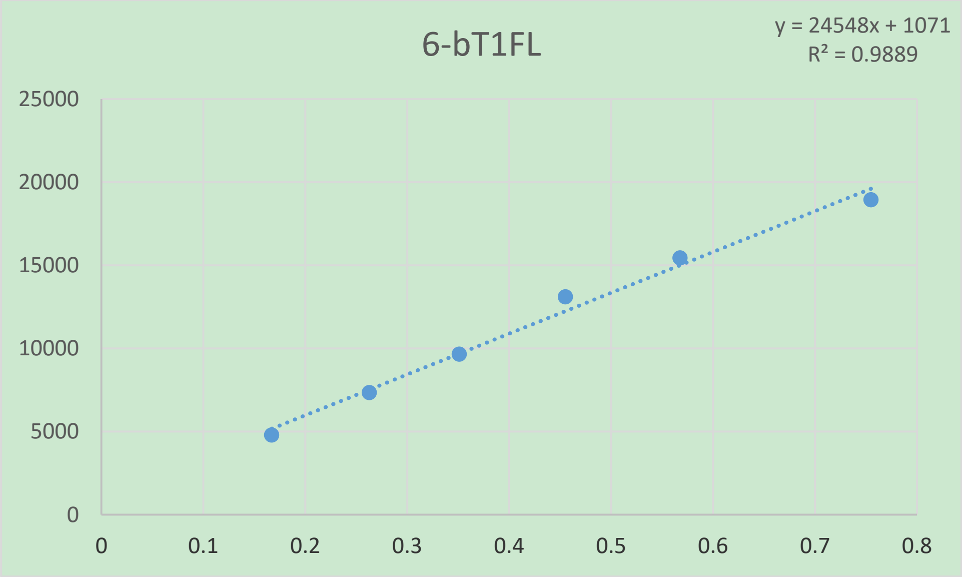
6-bT1FL
y = 24548x + 1071
R² = 0.9889
25000
20000
15000
10000
5000
0
0
0.1
0.2
0.3
0.4
0.5
0.6
0.7
0.8

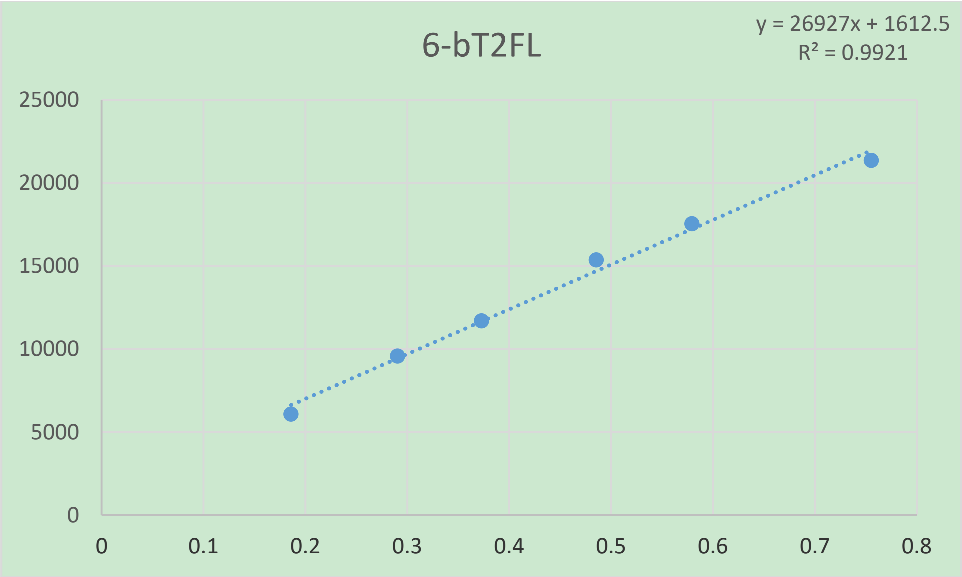
6-bT2FL
y = 26927x + 1612.5
R² = 0.9921
25000
20000
15000
10000
5000
0
0
0.1
0.2
0.3
0.4
0.5
0.6
0.7
0.8

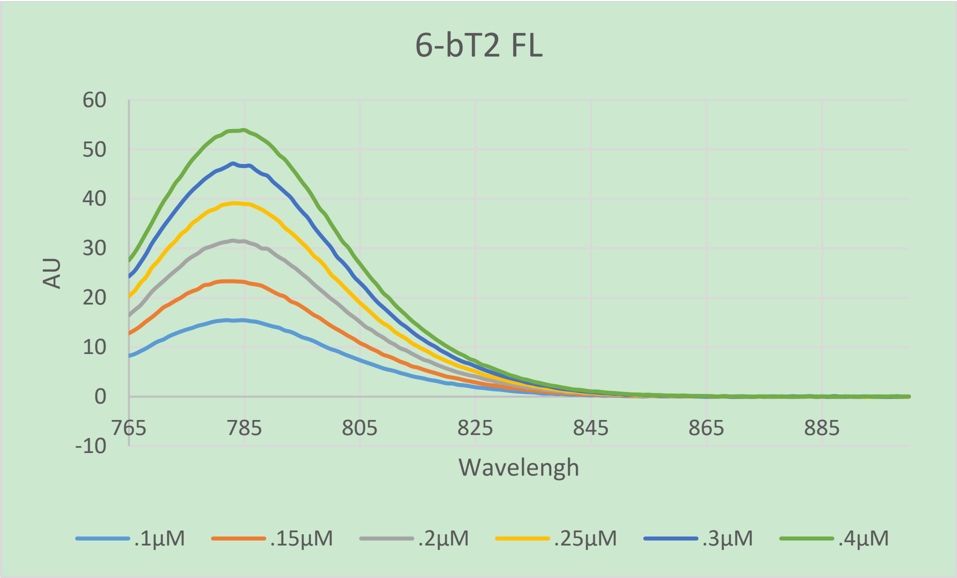
6-bT2 FL
60
50
40
30
20
10
0
-10
AU
765
785
805
825
845
865
885
Wavelengh
.1µM
.15µM
.2µM
.25µM
.3µM
.4µM

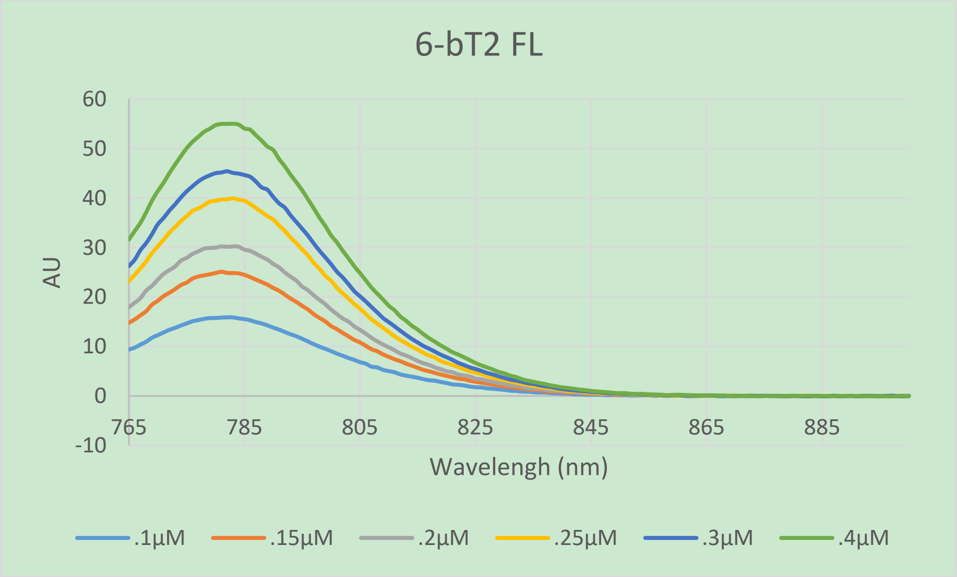
6-bT2 FL
60
50
40
30
20
10
0
-10
AU
765
785
805
825
845
865
885
Wavelengh (nm)
.1µM
.15µM
.2µM
.25µM
.3µM
.4µM

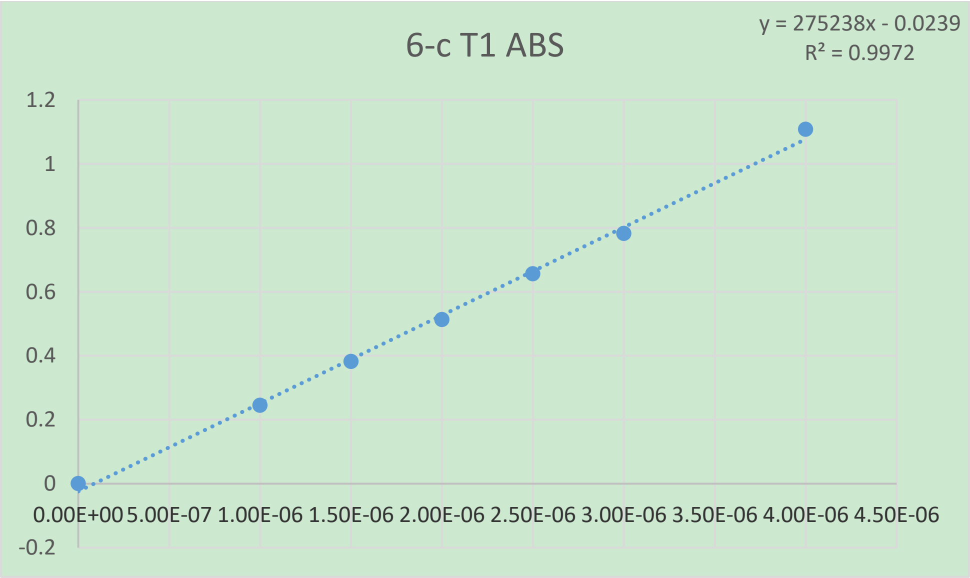
6-c T1 ABS
y = 275238x - 0.0239
R² = 0.9972
1.2
1
0.8
0.6
0.4
0.2
0
-0.2
0.00E+00 5.00E-07 1.00E-06 1.50E-06 2.00E-06 2.50E-06 3.00E-06 3.50E-06 4.00E-06 4.50E-06

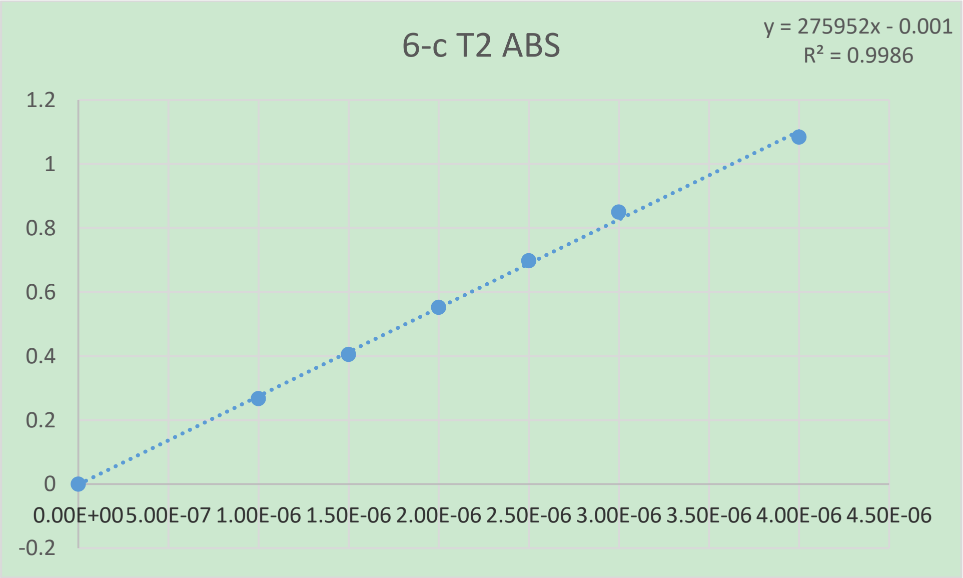
6-c T2 ABS
y = 275952x - 0.001
R² = 0.9986
1.2
1
0.8
0.6
0.4
0.2
0
-0.2
0.00E+00 5.00E-07 1.00E-06 1.50E-06 2.00E-06 2.50E-06 3.00E-06 3.50E-06 4.00E-06 4.50E-06

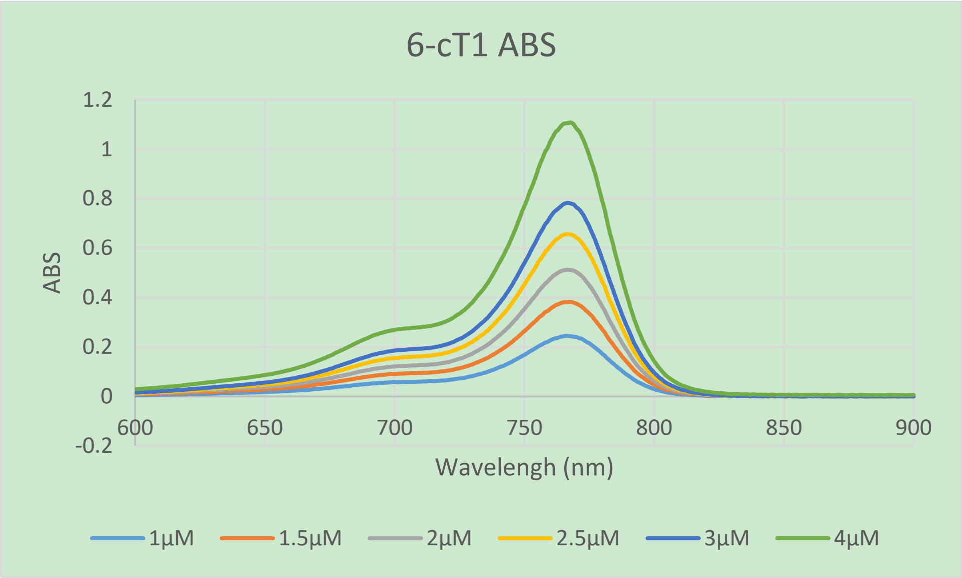
6-cT1 ABS
1.2
1
0.8
0.6
0.4
0.2
0
-0.2
ABS
600
650
700
750
800
850
900
Wavelengh (nm)
1μM
1.5μM
2μM
2.5μM
3μM
4μM

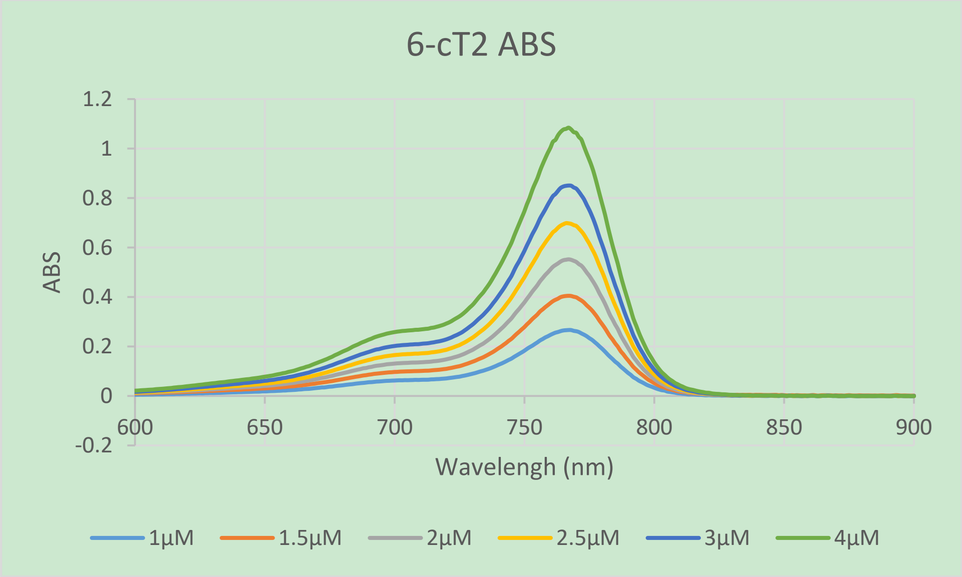
6-cT2 ABS
1.2
1
0.8
0.6
0.4
0.2
0
-0.2
ABS
600
650
700
750
800
850
900
Wavelengh (nm)
1μM
1.5μM
2μM
2.5μM
3μM
4μM

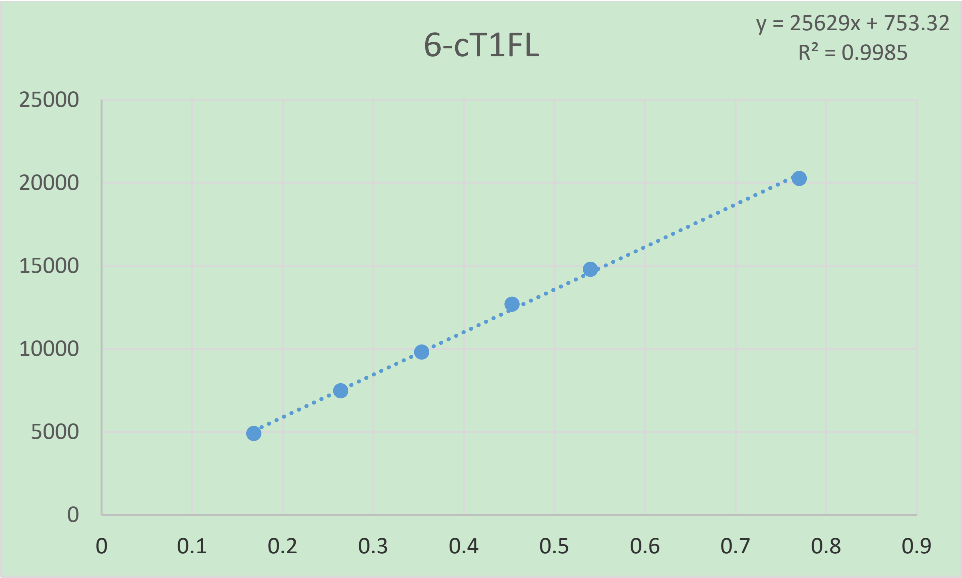

6-cT1FL
y = 25629x + 753.32
R² = 0.9985
25000
20000
15000
10000
5000
0
0
0.1
0.2
0.3
0.4
0.5
0.6
0.7
0.8
0.9

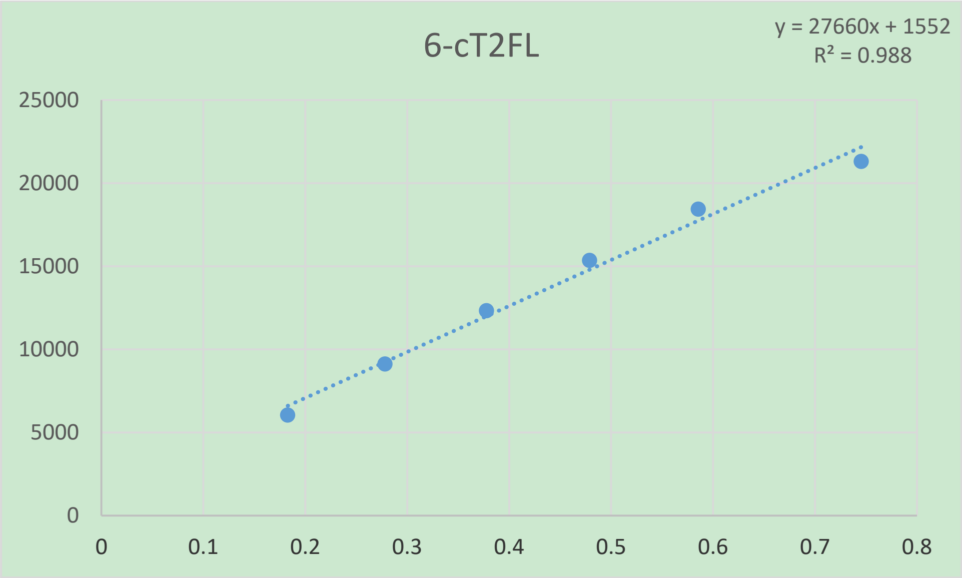

6-cT2FL
y = 27660x + 1552
R² = 0.988
25000
20000
15000
10000
5000
0
0
0.1
0.2
0.3
0.4
0.5
0.6
0.7
0.8

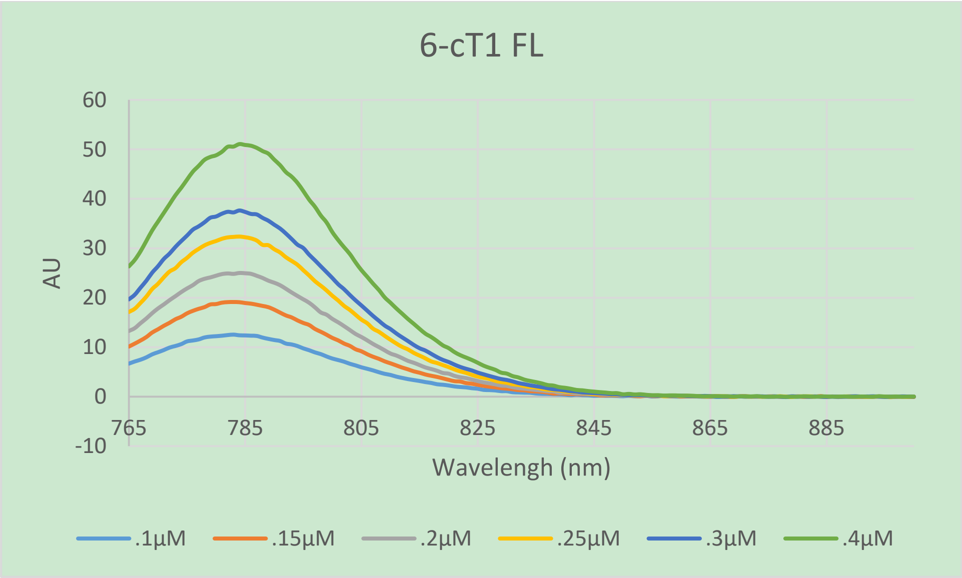
6-cT1 FL
60
50
40
30
20
10
0
-10
AU
765
785
805
825
845
865
885
Wavelengh (nm)
.1µM
.15µM
.2µM
.25µM
.3µM
.4µM

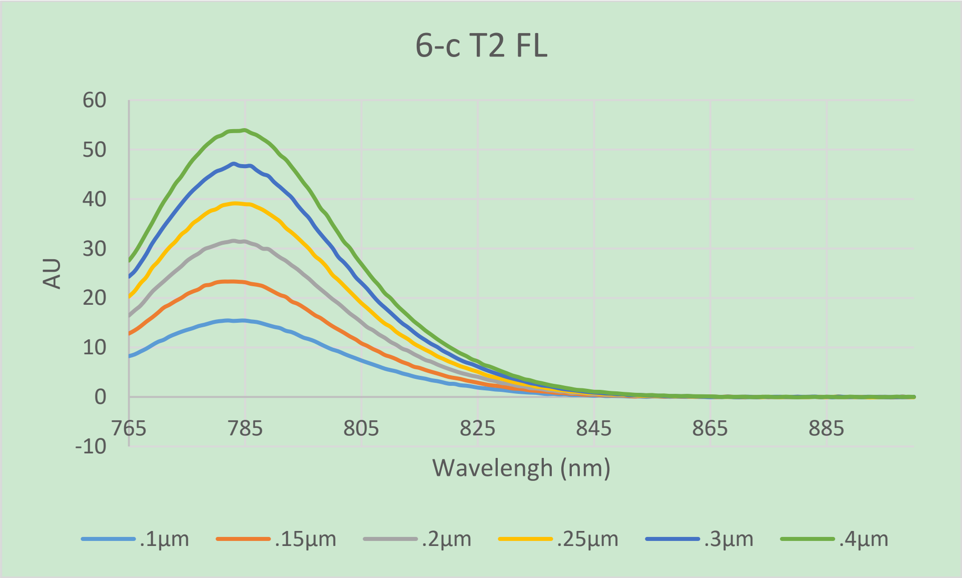
6-c T2 FL
60
50
40
30
20
10
0
-10
AU
765
785
805
825
845
865
885
Wavelengh (nm)
.1µm
.15µm
.2µm
.25µm
.3µm
.4µm

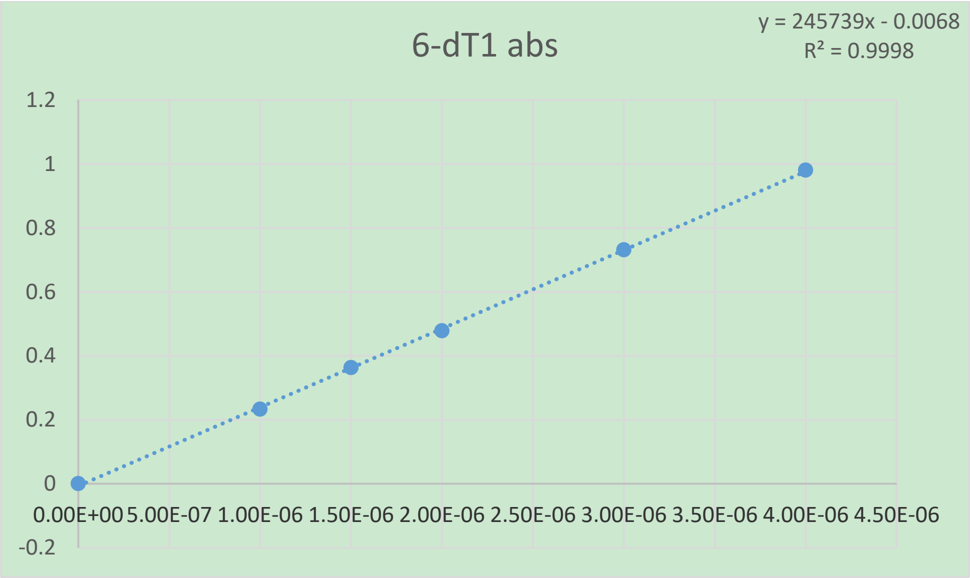
6-dT1 abs
y = 245739x - 0.0068
R² = 0.9998
1.2
1
0.8
0.6
0.4
0.2
0
-0.2
0.00E+00 5.00E-07 1.00E-06 1.50E-06 2.00E-06 2.50E-06 3.00E-06 3.50E-06 4.00E-06 4.50E-06

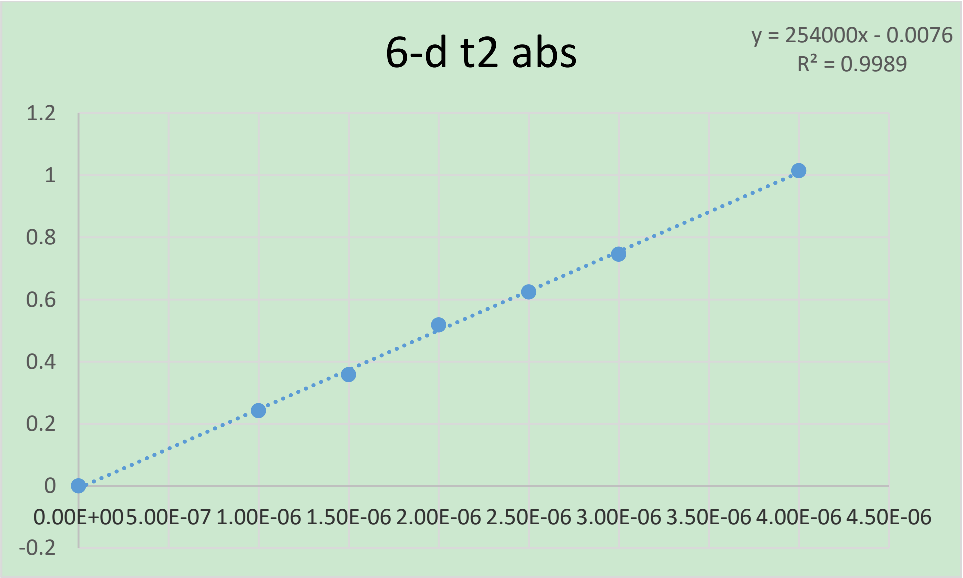
6-d t2 abs
y = 254000x - 0.0076
R² = 0.9989
1.2
1
0.8
0.6
0.4
0.2
0
-0.2
0.00E+00 5.00E-07 1.00E-06 1.50E-06 2.00E-06 2.50E-06 3.00E-06 3.50E-06 4.00E-06 4.50E-06

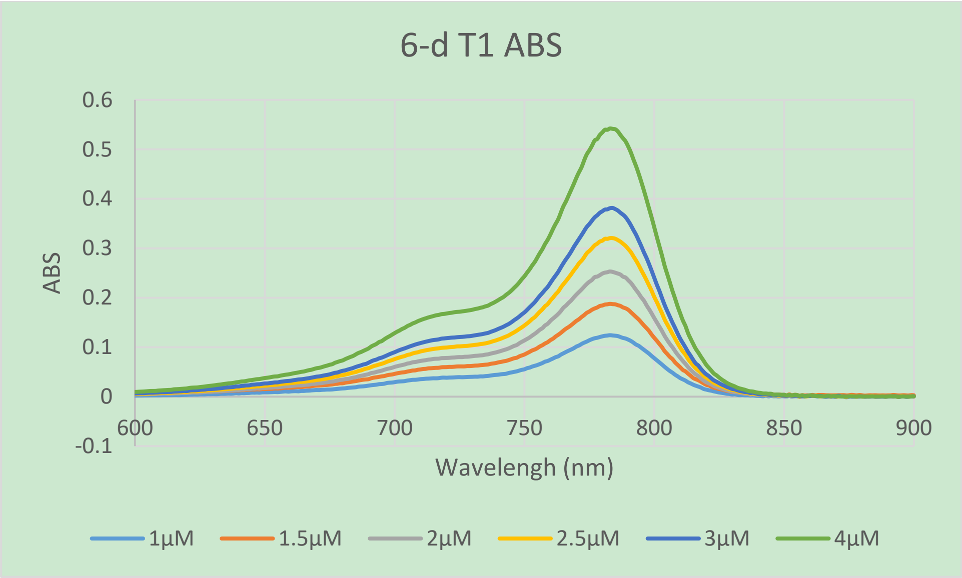
6-d T1 ABS
ABS
0.6
0.5
0.4
0.3
0.2
0.1
0
-0.1
600
650
700
750
800
850
900
Wavelengh (nm)
1μM
1.5μM
2μM
2.5μM
3μM
4μM

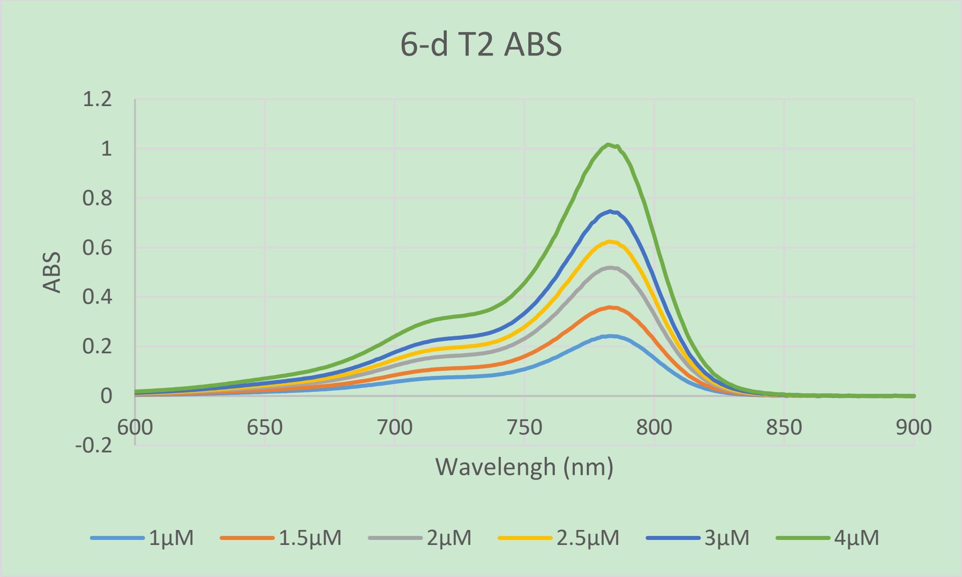
6-d T2 ABS
ABS
1.2
1
0.8
0.6
0.4
0.2
0
-0.2
600
650
700
750
800
850
900
Wavelengh (nm)
1μM
1.5μM
2μM
2.5μM
3μM
4μM

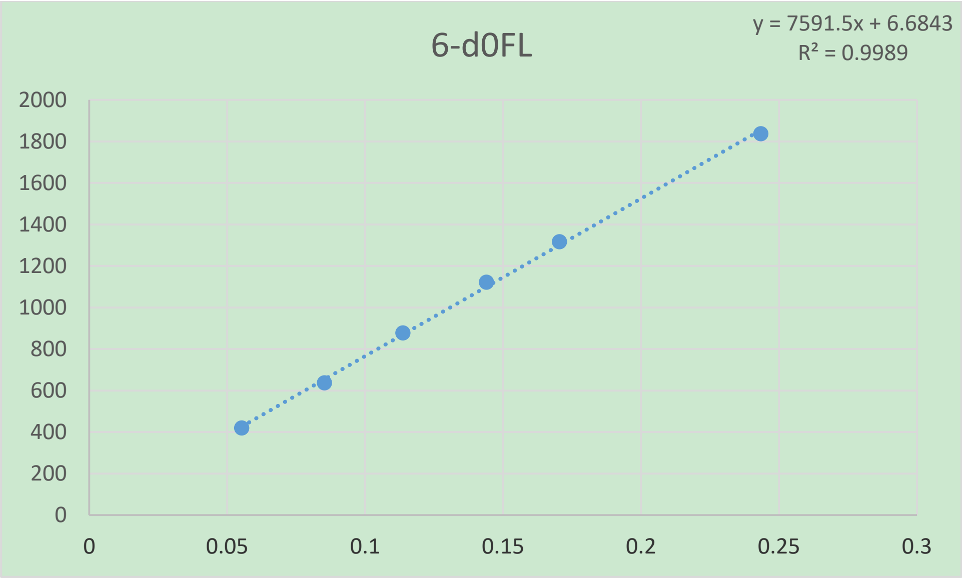
6-d0FL
y = 7591.5x + 6.6843
R² = 0.9989
2000
1800
1600
1400
1200
1000
800
600
400
200
0
0
0.05
0.1
0.15
0.2
0.25
0.3

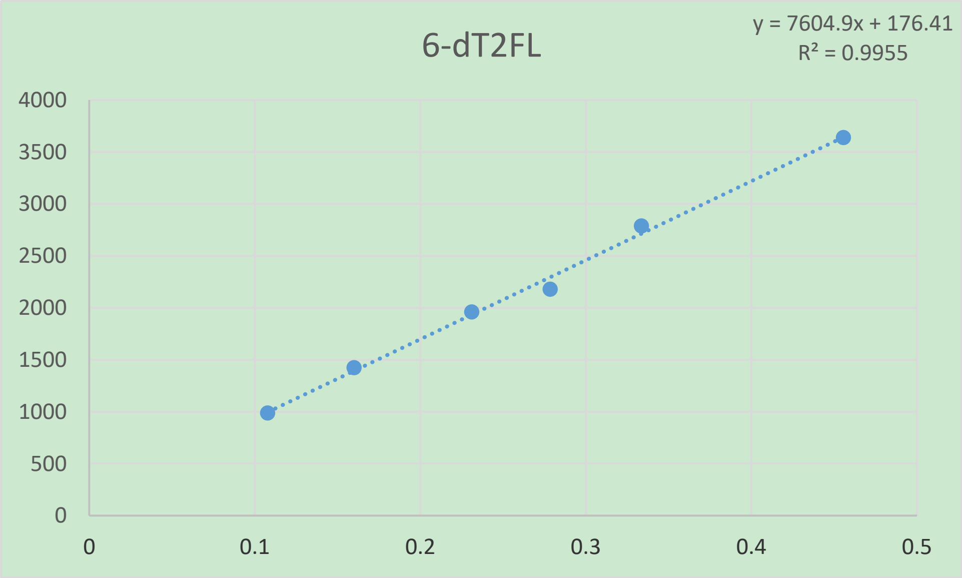
6-dT2FL
y = 7604.9x + 176.41
R² = 0.9955
4000
3500
3000
2500
2000
1500
1000
500
0
0
0.1
0.2
0.3
0.4
0.5

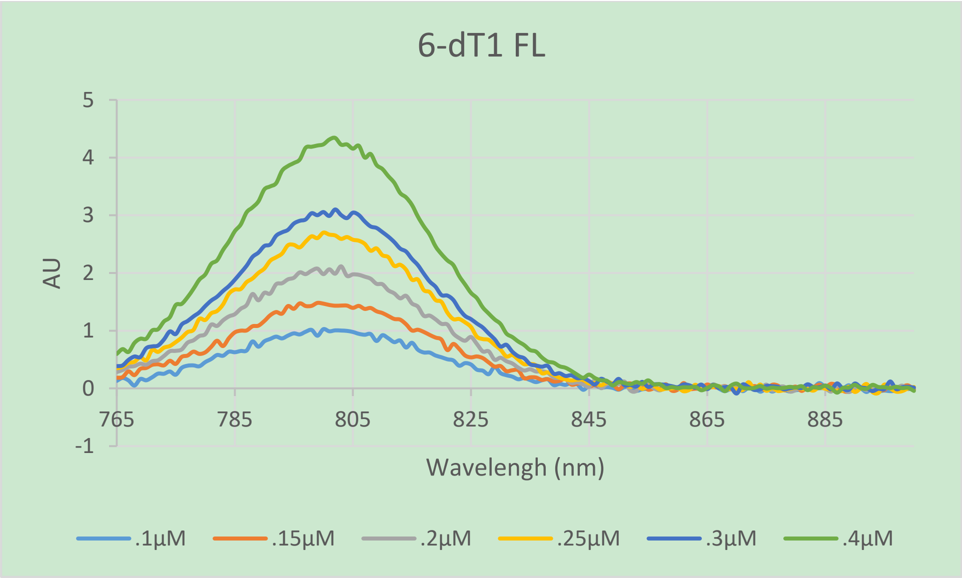
6-dT1 FL
5
4
3
2
1
0
-1
AU
765
785
805
825
845
865
885
Wavelengh (nm)
.1μM
.15μM
.2μM
.25μM
.3μM
.4μM

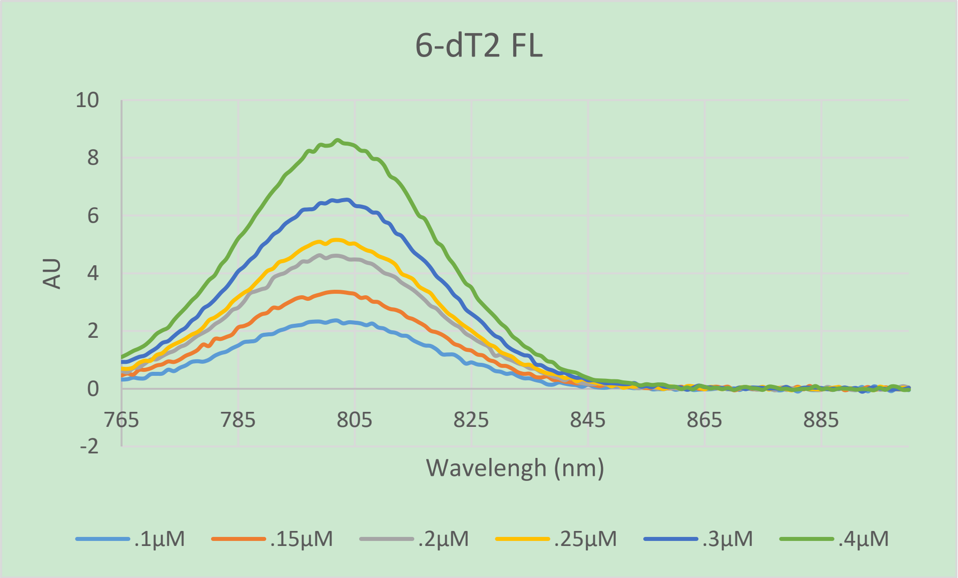
6-dT2 FL
10
8
6
4
2
0
-2
AU
765
785
805
825
845
865
885
Wavelengh (nm)
.1μM
.15μM
.2μM
.25μM
.3μM
.4μM

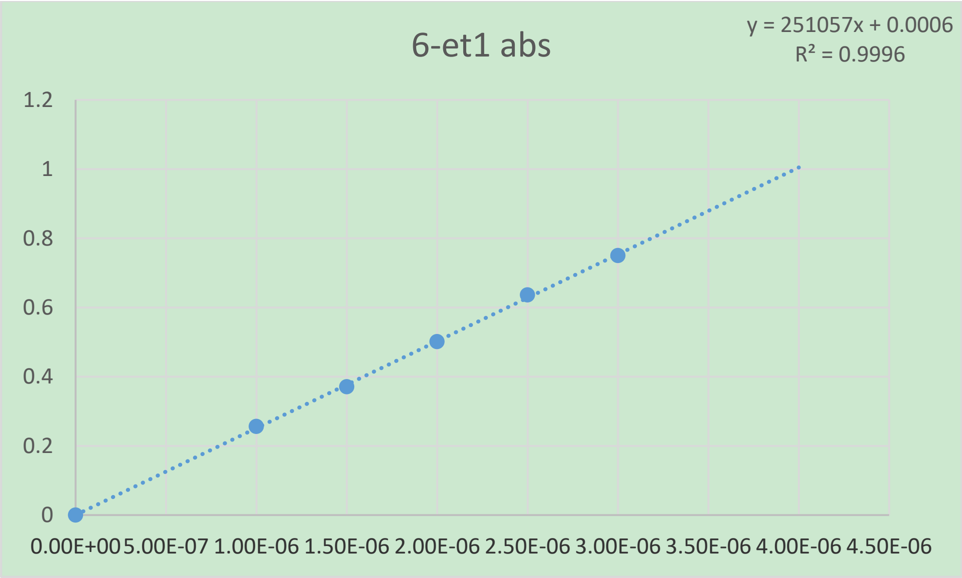
6-et1 abs
y = 251057x + 0.0006
R² = 0.9996
1.2
1
0.8
0.6
0.4
0.2
0
0.00E+00 5.00E-07 1.00E-06 1.50E-06 2.00E-06 2.50E-06 3.00E-06 3.50E-06 4.00E-06 4.50E-06

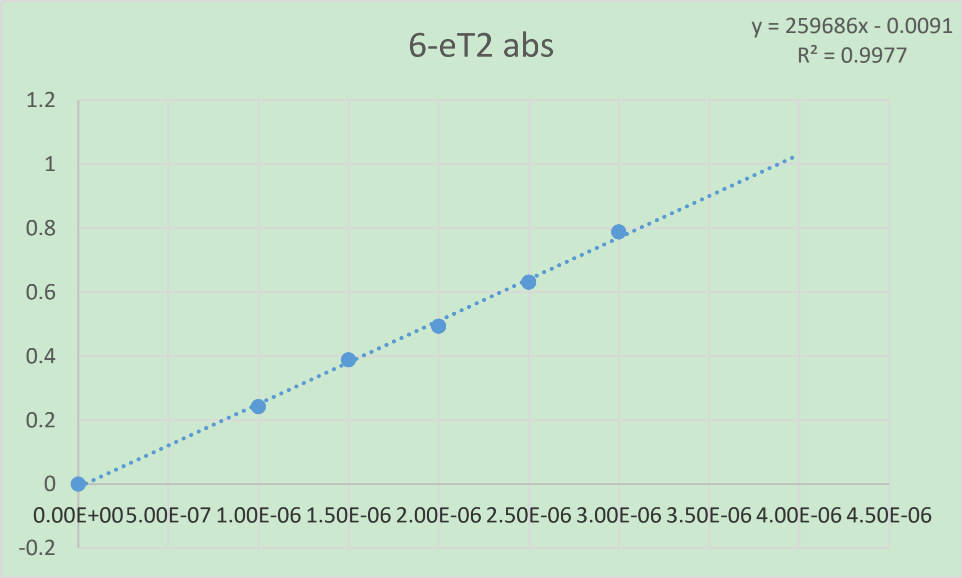
6-eT2 abs
y = 259686x - 0.0091
R² = 0.9977
1.2
1
0.8
0.6
0.4
0.2
0
-0.2
0.00E+00 5.00E-07 1.00E-06 1.50E-06 2.00E-06 2.50E-06 3.00E-06 3.50E-06 4.00E-06 4.50E-06

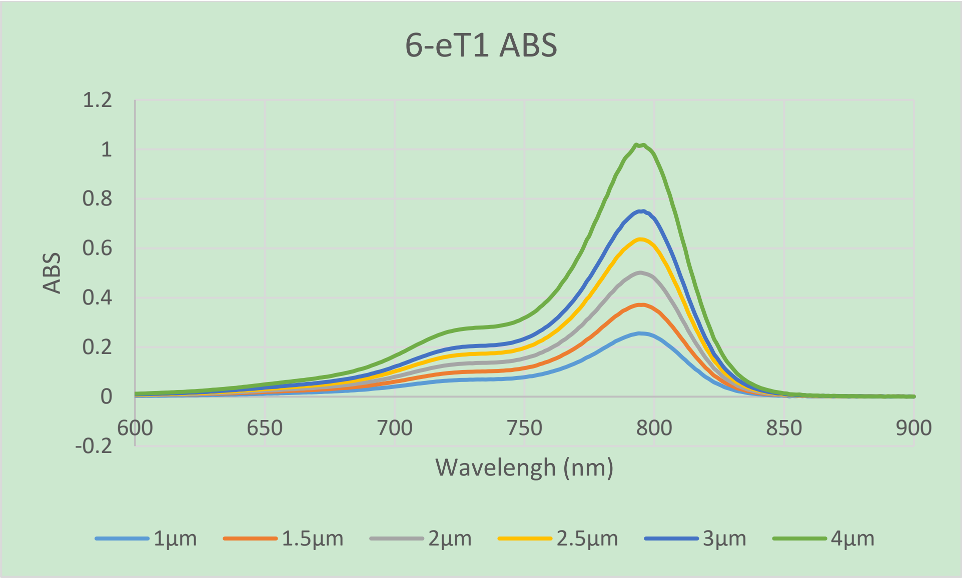
6-eT1 ABS
ABS
1.2
1
0.8
0.6
0.4
0.2
0
-0.2
600
650
700
750
800
850
900
Wavelengh (nm)
1µm
1.5µm
2µm
2.5µm
3µm
4µm

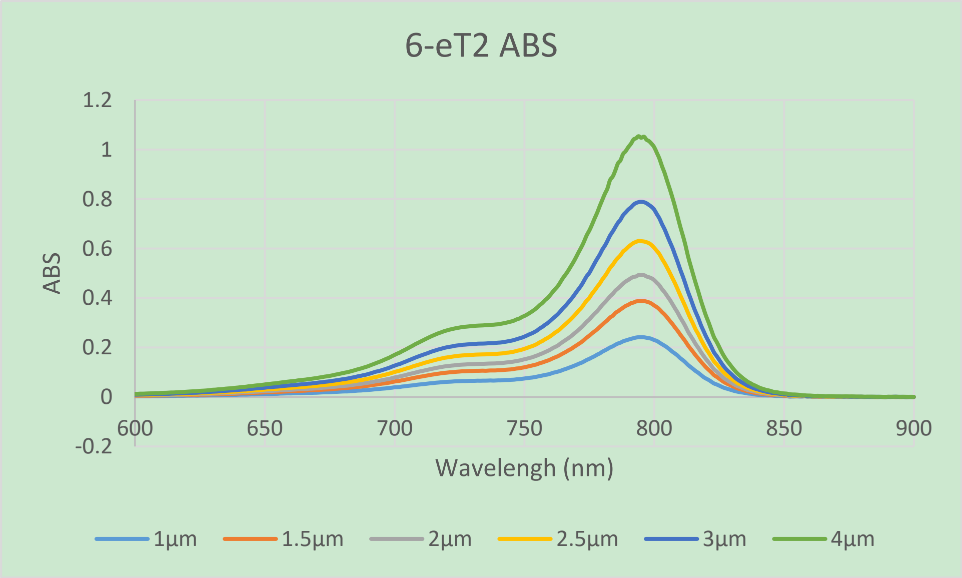
6-eT2 ABS
ABS
1.2
1
0.8
0.6
0.4
0.2
0
-0.2
600
650
700
750
800
850
900
Wavelengh (nm)
1µm
1.5µm
2µm
2.5µm
3µm
4µm

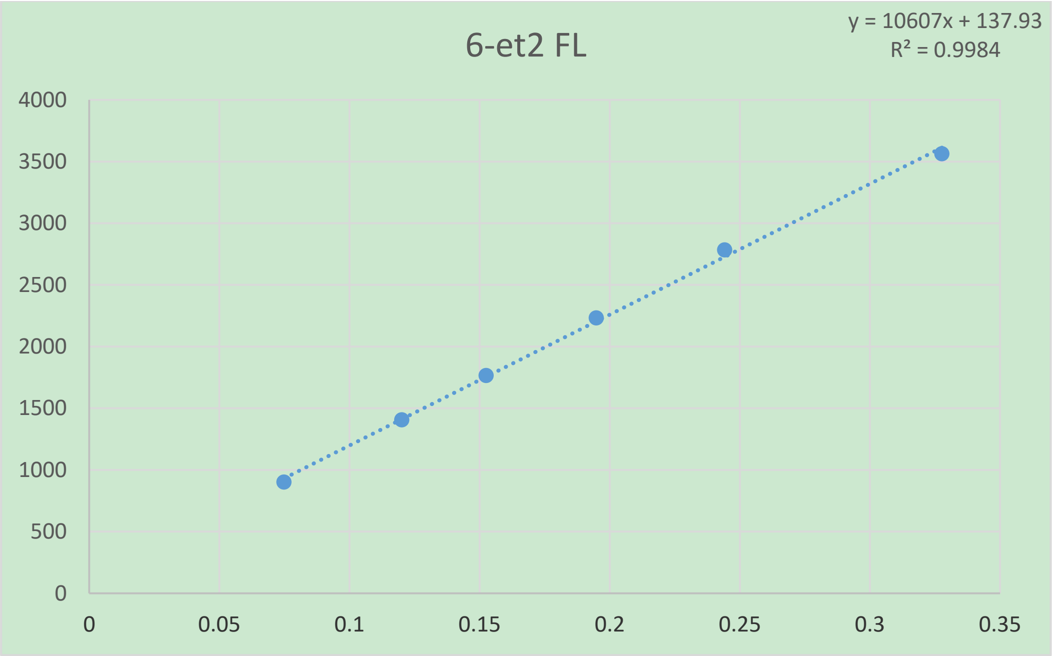
6-et2 FL
y = 10607x + 137.93
R² = 0.9984
4000
3500
3000
2500
2000
1500
1000
500
0
0
0.05
0.1
0.15
0.2
0.25
0.3
0.35

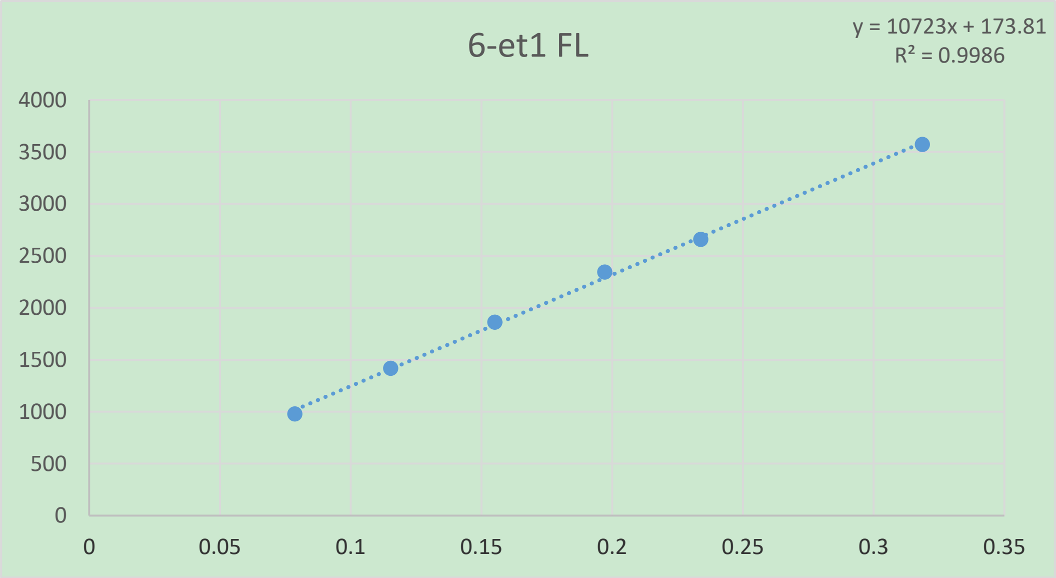
6-et1 FL
y = 10723x + 173.81
R² = 0.9986
4000
3500
3000
2500
2000
1500
1000
500
0
0
0.05
0.1
0.15
0.2
0.25
0.3
0.35

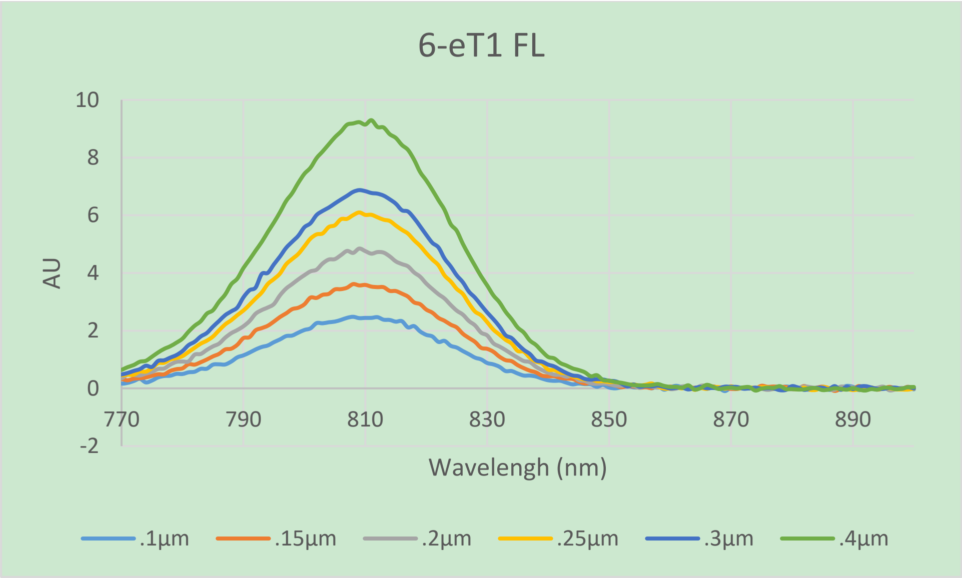
6-eT1 FL
10
8
6
4
2
0
-2
AU
770
790
810
830
850
870
890
Wavelengh (nm)
.1µm
.15µm
.2µm
.25µm
.3µm
.4µm

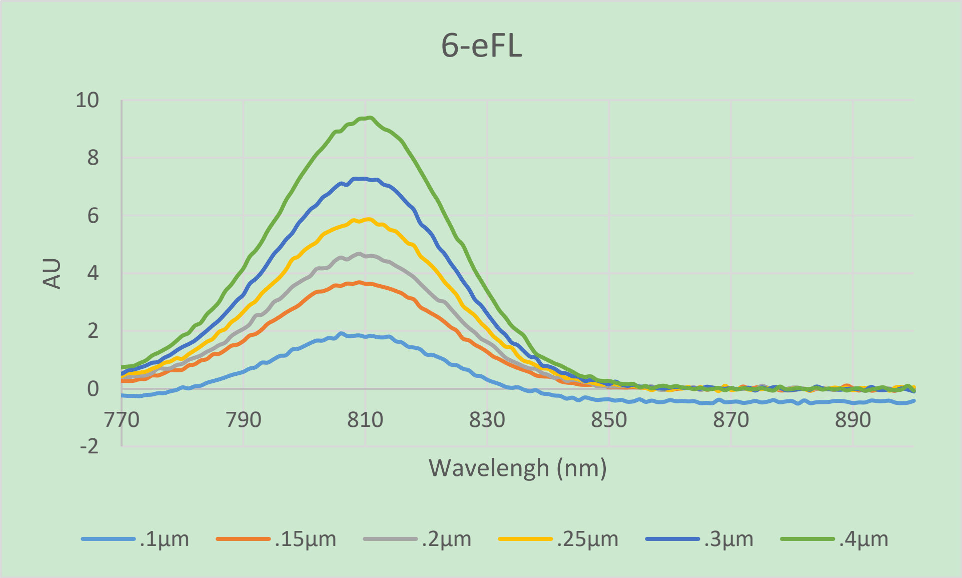
6-eFL
10
8
6
4
2
0
-2
AU
770
790
810
830
850
870
890
Wavelengh (nm)
.1µm
.15µm
.2µm
.25µm
.3µm
.4µm

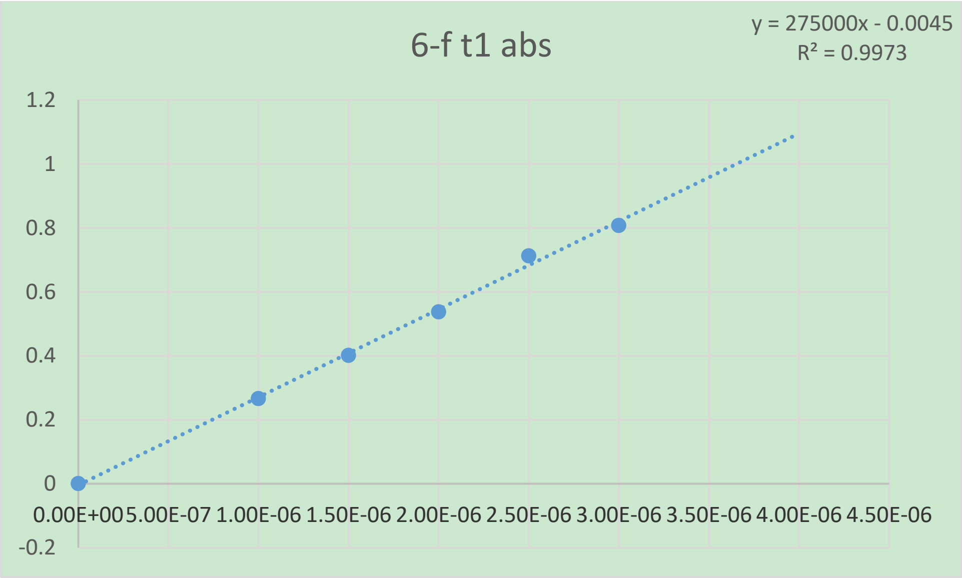
6-f t1 abs
y = 275000x - 0.0045
R^2 = 0.9973
1.2
1
0.8
0.6
0.4
0.2
0
-0.2
0.00E+00 5.00E-07 1.00E-06 1.50E-06 2.00E-06 2.50E-06 3.00E-06 3.50E-06 4.00E-06 4.50E-06

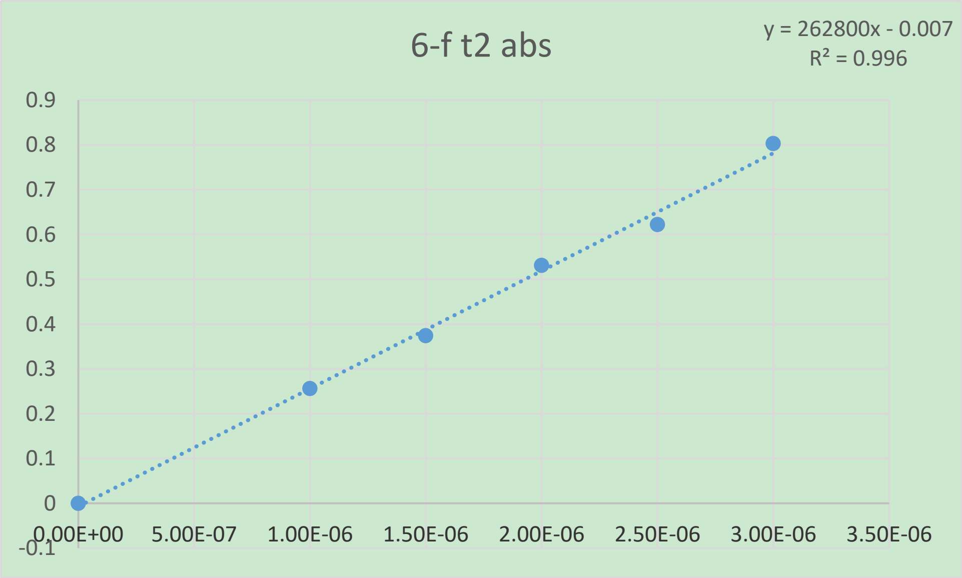
6-f t2 abs
y = 262800x - 0.007
R^2 = 0.996
0.9
0.8
0.7
0.6
0.5
0.4
0.3
0.2
0.1
0
-0.1
0.00E+00 5.00E-07 1.00E-06 1.50E-06 2.00E-06 2.50E-06 3.00E-06 3.50E-06

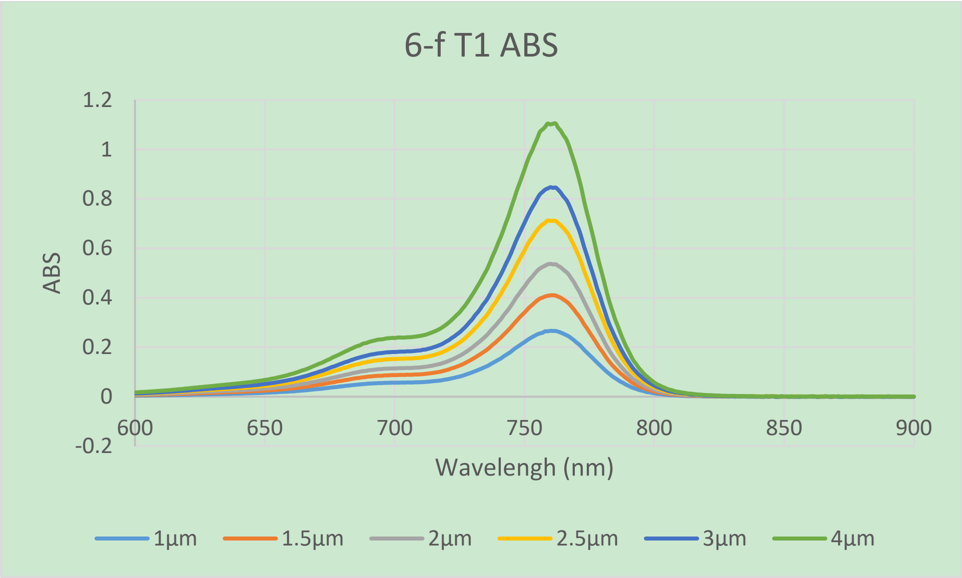

6-f T1 ABS
ABS
1.2
1
0.8
0.6
0.4
0.2
0
-0.2
600
650
700
750
800
850
900
Wavelengh (nm)
1µm
1.5µm
2µm
2.5µm
3µm
4µm

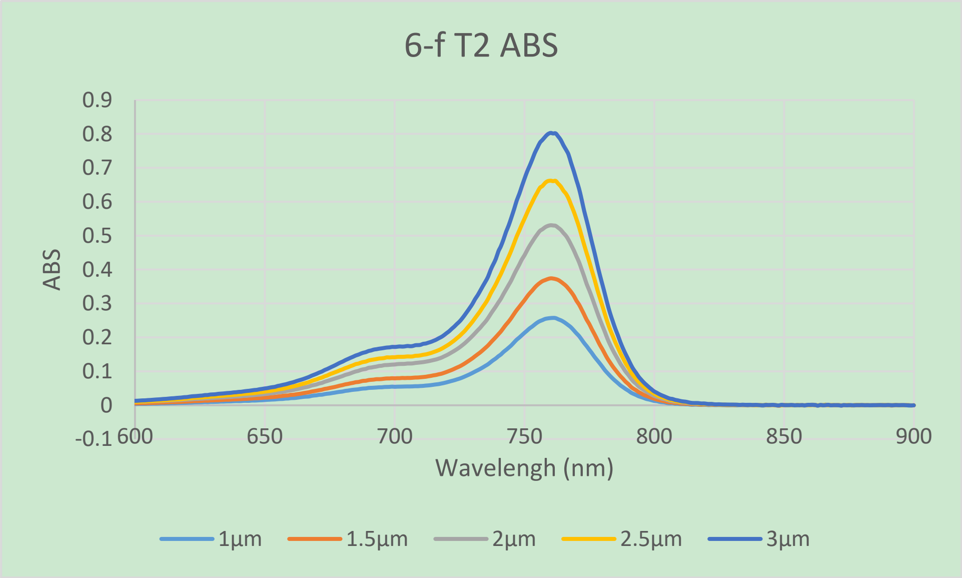

6-f T2 ABS
ABS
0.9
0.8
0.7
0.6
0.5
0.4
0.3
0.2
0.1
0
-0.1
600
650
700
750
800
850
900
Wavelengh (nm)
1µm
1.5µm
2µm
2.5µm
3µm

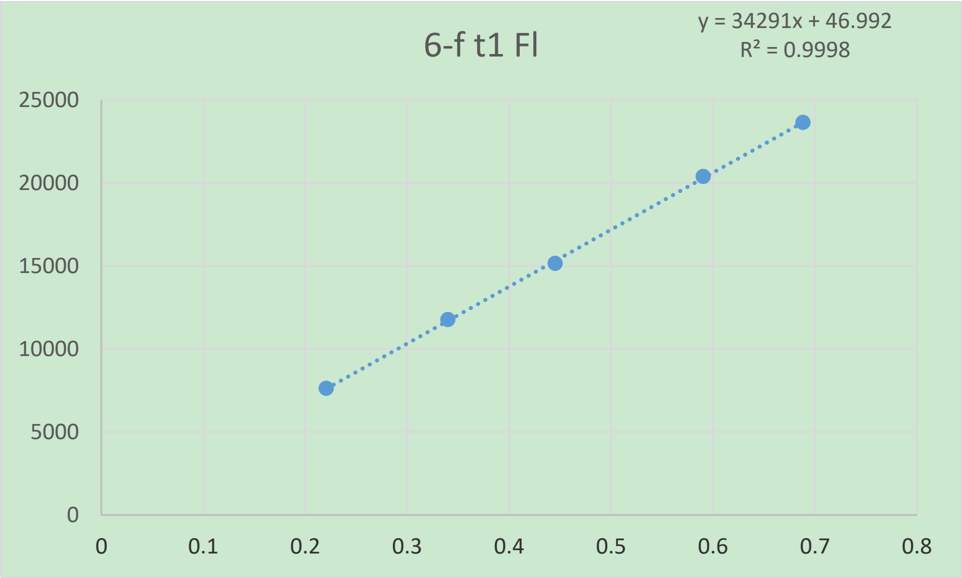
6-f t1 Fl
y = 34291x + 46.992
R² = 0.9998
25000
20000
15000
10000
5000
0
0
0.1
0.2
0.3
0.4
0.5
0.6
0.7
0.8

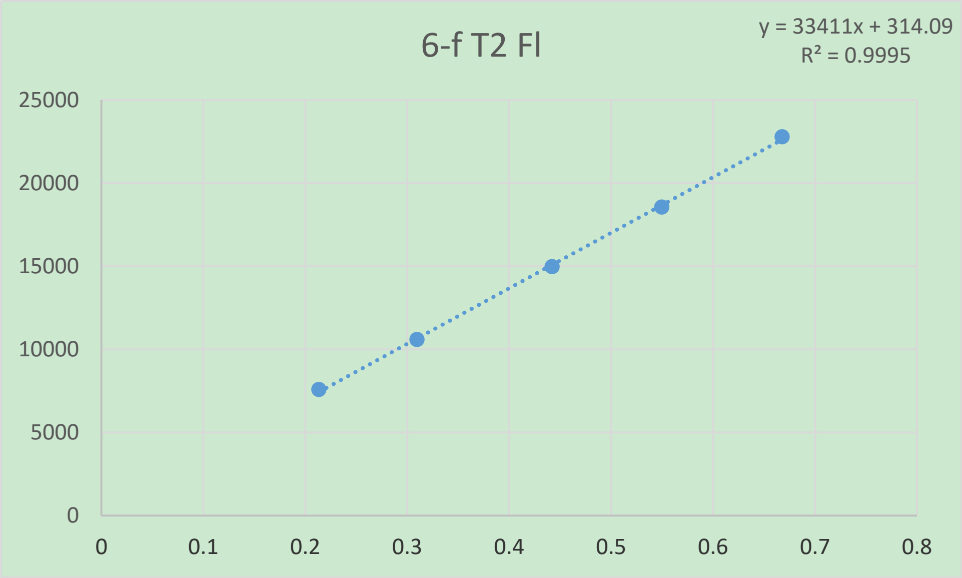
6-f T2 Fl
y = 33411x + 314.09
R² = 0.9995
25000
20000
15000
10000
5000
0
0
0.1
0.2
0.3
0.4
0.5
0.6
0.7
0.8

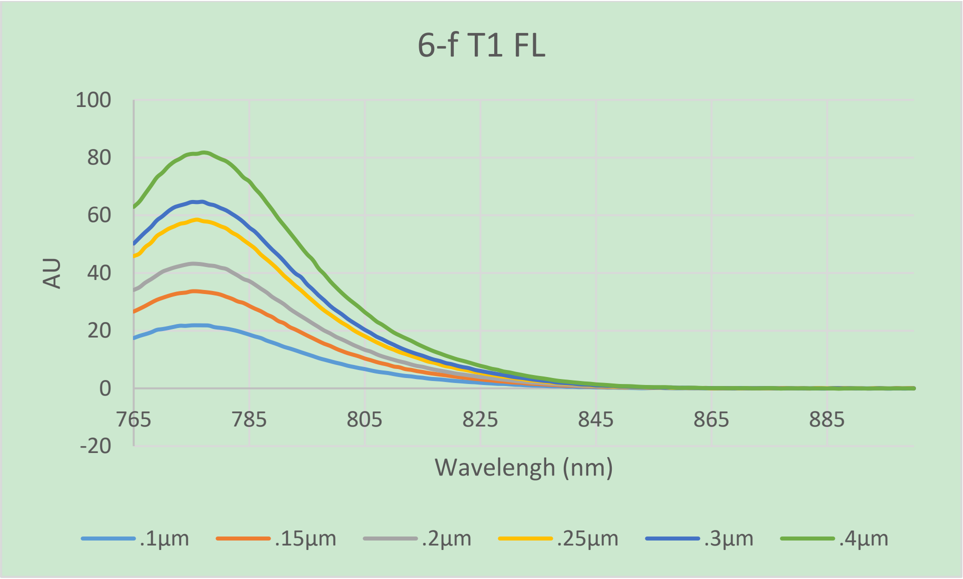
6-f T1 FL
100
80
60
40
20
0
-20
AU
765
785
805
825
845
865
885
Wavelengh (nm)
.1µm
.15µm
.2µm
.25µm
.3µm
.4µm

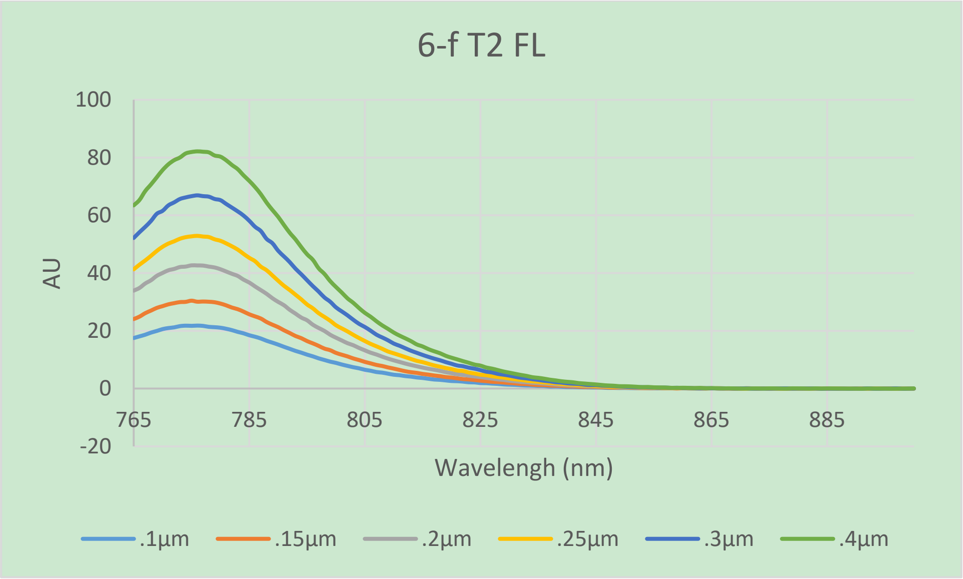
6-f T2 FL
100
80
60
40
20
0
-20
AU
765
785
805
825
845
865
885
Wavelengh (nm)
.1µm
.15µm
.2µm
.25µm
.3µm
.4µm

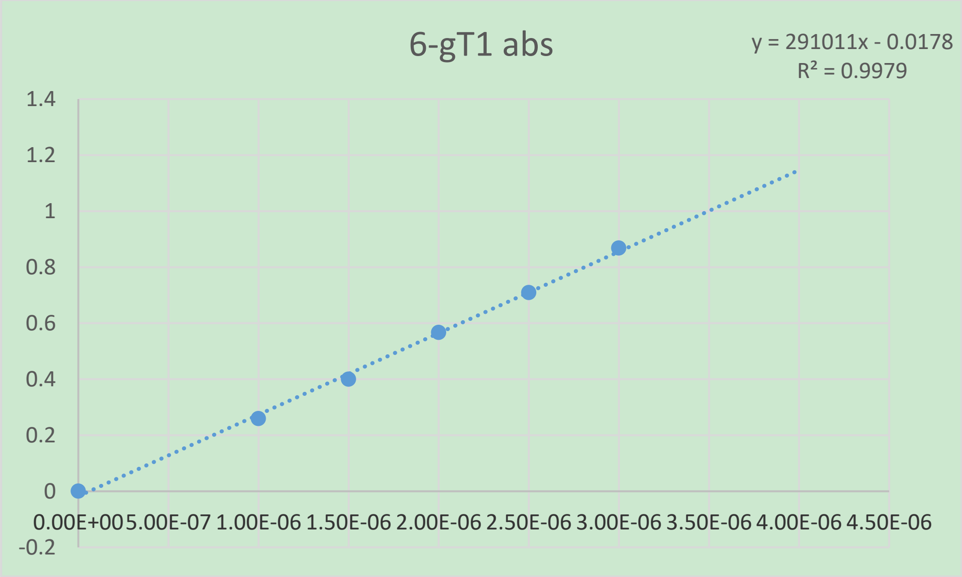
6-gT1 abs
y = 291011x - 0.0178
R² = 0.9979
1.4
1.2
1
0.8
0.6
0.4
0.2
0
-0.2
0.00E+00 5.00E-07 1.00E-06 1.50E-06 2.00E-06 2.50E-06 3.00E-06 3.50E-06 4.00E-06 4.50E-06

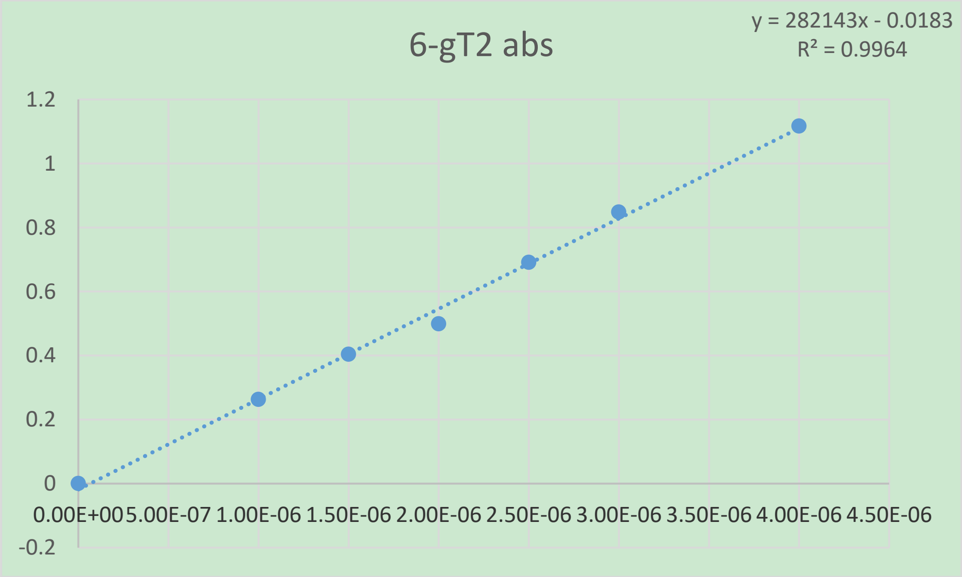
6-gT2 abs
y = 282143x - 0.0183
R² = 0.9964
1.2
1
0.8
0.6
0.4
0.2
0
-0.2
0.00E+00 5.00E-07 1.00E-06 1.50E-06 2.00E-06 2.50E-06 3.00E-06 3.50E-06 4.00E-06 4.50E-06

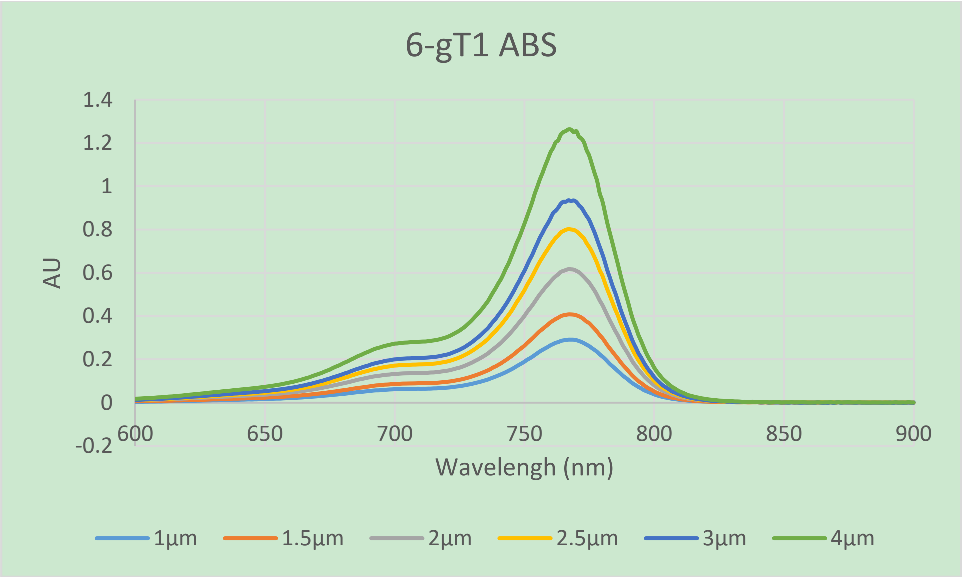
6-gT1 ABS
AU
1.4
1.2
1
0.8
0.6
0.4
0.2
0
-0.2
600
650
700
750
800
850
900
Wavelengh (nm)
1µm
1.5µm
2µm
2.5µm
3µm
4µm

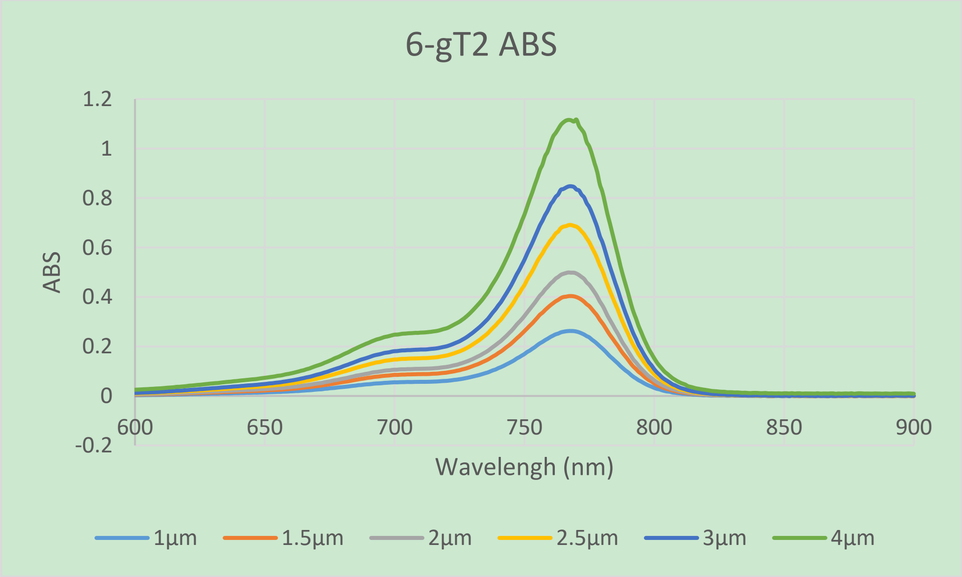
6-gT2 ABS
ABS
1.2
1
0.8
0.6
0.4
0.2
0
-0.2
600
650
700
750
800
850
900
Wavelengh (nm)
1µm
1.5µm
2µm
2.5µm
3µm
4µm

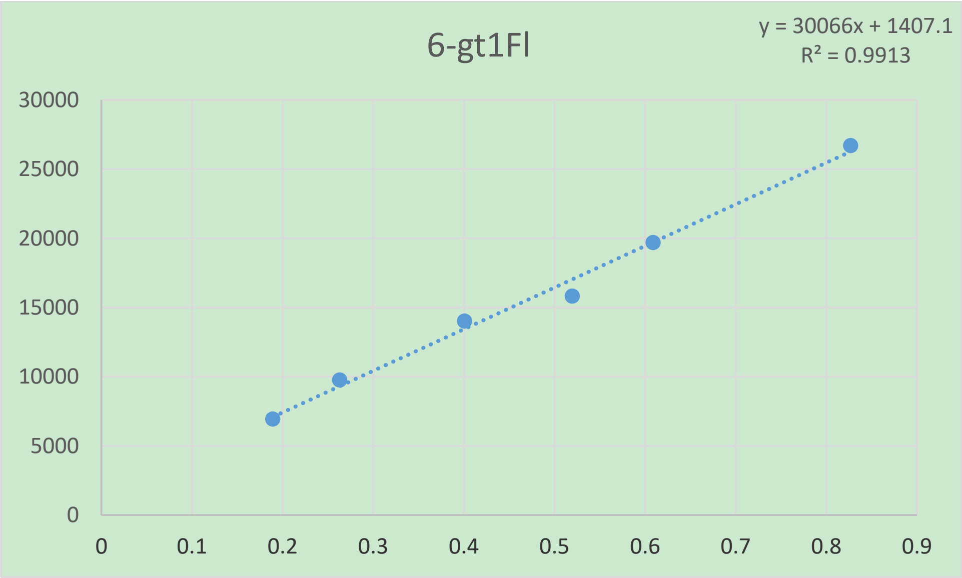
6-gt1Fl
y = 30066x + 1407.1
R² = 0.9913
30000
25000
20000
15000
10000
5000
0
0
0.1
0.2
0.3
0.4
0.5
0.6
0.7
0.8
0.9

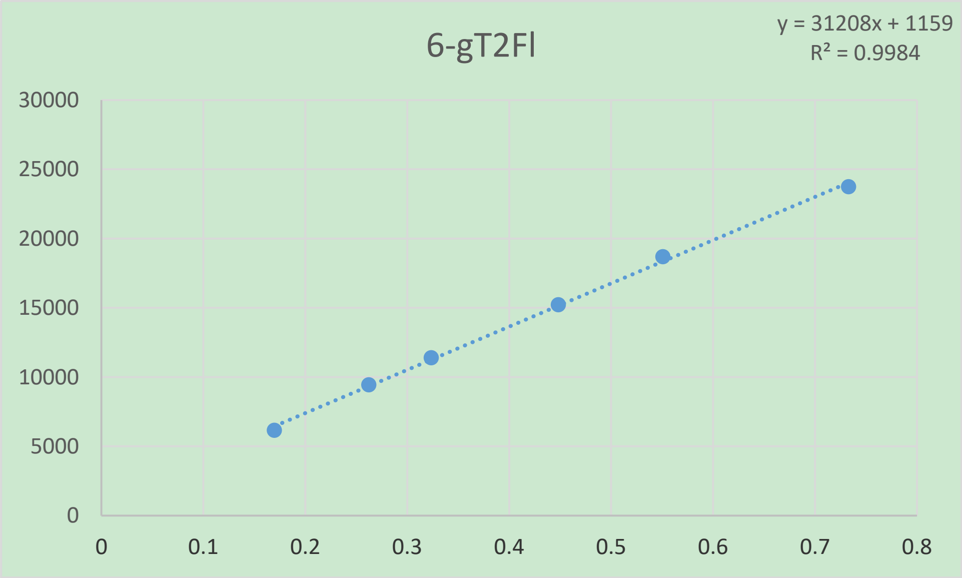
6-gT2Fl
y = 31208x + 1159
R² = 0.9984
30000
25000
20000
15000
10000
5000
0
0
0.1
0.2
0.3
0.4
0.5
0.6
0.7
0.8

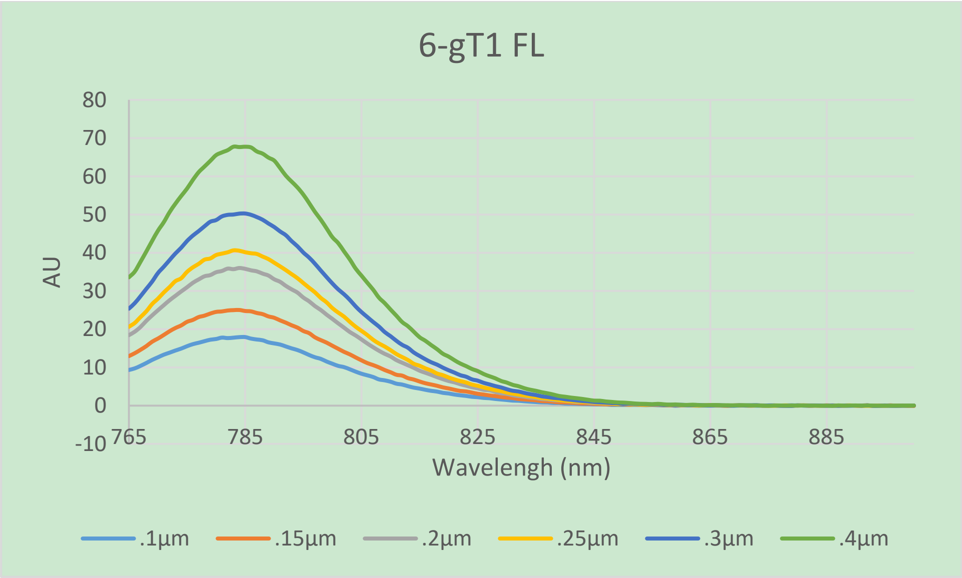
6-gT1 FL
80
70
60
50
40
30
20
10
0
-10
AU
765
785
805
825
845
865
885
Wavelengh (nm)
.1µm
.15µm
.2µm
.25µm
.3µm
.4µm

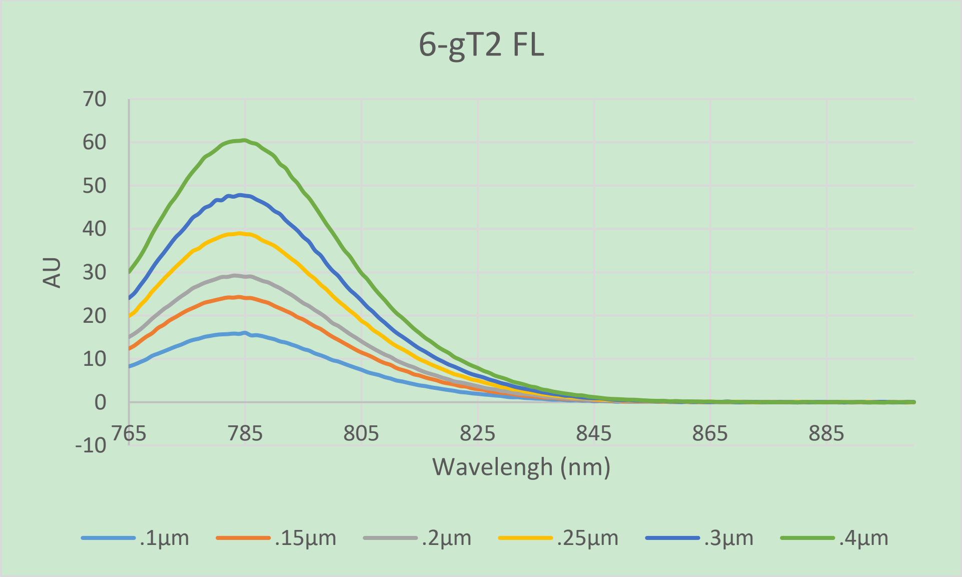
6-gT2 FL
70
60
50
40
30
20
10
0
-10
AU
765
785
805
825
845
865
885
Wavelengh (nm)
.1µm
.15µm
.2µm
.25µm
.3µm
.4µm

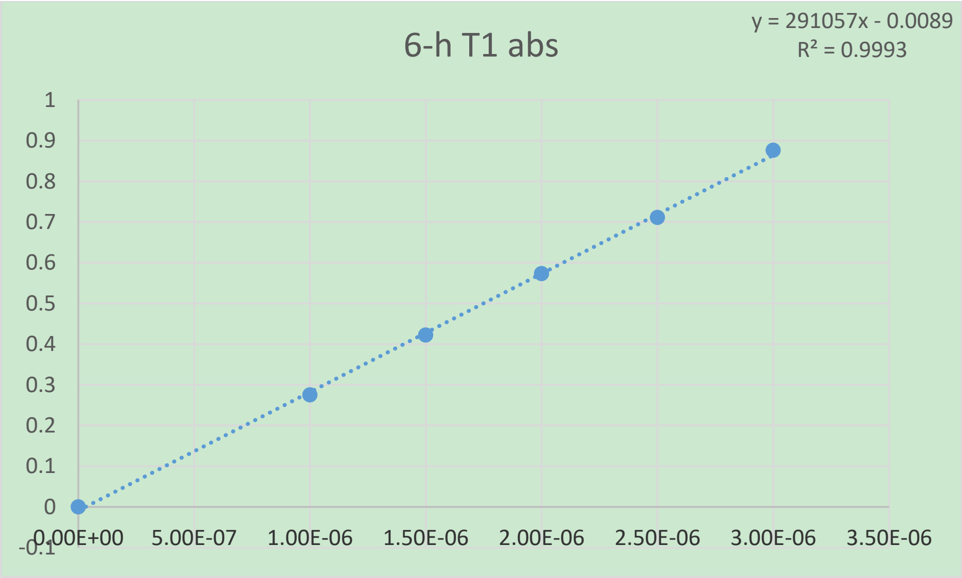
6-h T1 abs
y = 291057x - 0.0089
R² = 0.9993
1
0.9
0.8
0.7
0.6
0.5
0.4
0.3
0.2
0.1
0
-0.1
0.00E+00
5.00E-07
1.00E-06
1.50E-06
2.00E-06
2.50E-06
3.00E-06
3.50E-06

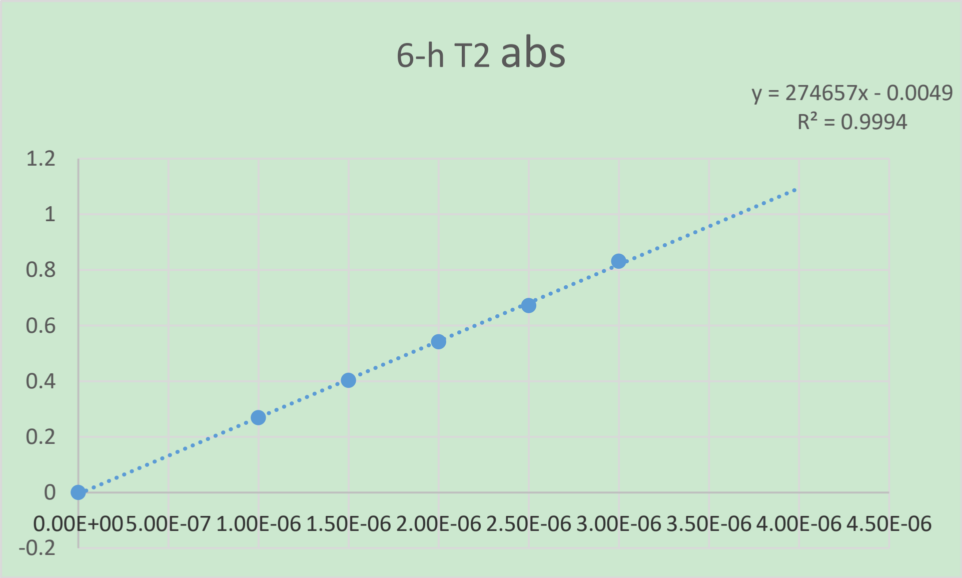
6-h T2 abs
y = 274657x - 0.0049
R² = 0.9994
1.2
1
0.8
0.6
0.4
0.2
0
-0.2
0.00E+00
5.00E-07
1.00E-06
1.50E-06
2.00E-06
2.50E-06
3.00E-06
3.50E-06
4.00E-06
4.50E-06

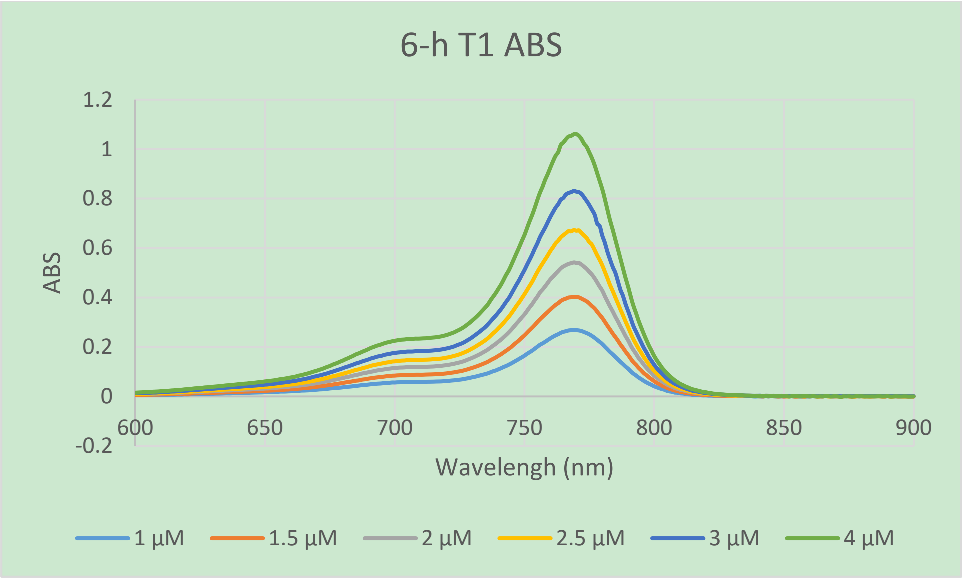
6-h T1 ABS
ABS
1.2
1
0.8
0.6
0.4
0.2
0
-0.2
600
650
700
750
800
850
900
Wavelengh (nm)
1 µM
1.5 µM
2 µM
2.5 µM
3 µM
4 µM

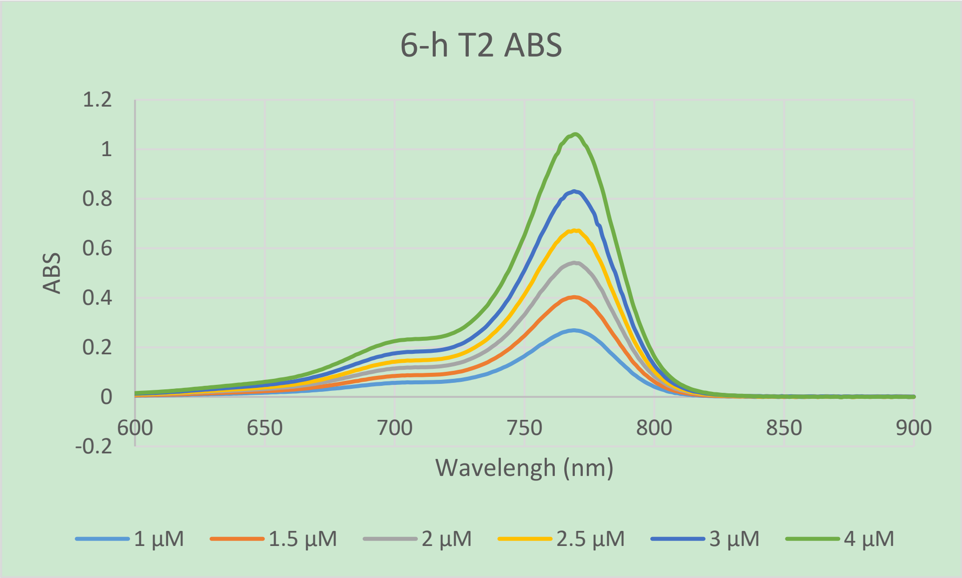
6-h T2 ABS
ABS
1.2
1
0.8
0.6
0.4
0.2
0
-0.2
600
650
700
750
800
850
900
Wavelengh (nm)
1 µM
1.5 µM
2 µM
2.5 µM
3 µM
4 µM

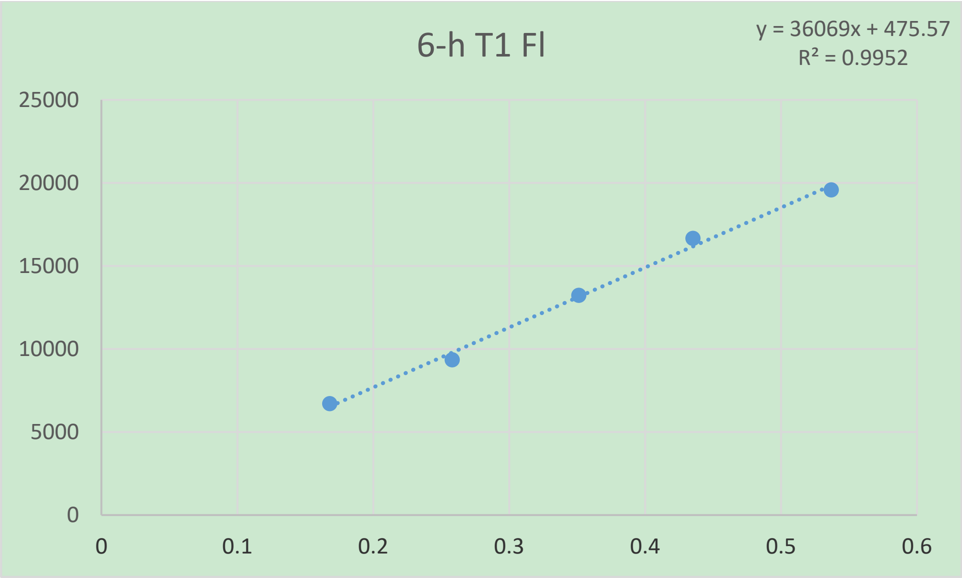
6-h T1 Fl
y = 36069x + 475.57
R² = 0.9952
25000
20000
15000
10000
5000
0
0
0.1
0.2
0.3
0.4
0.5
0.6

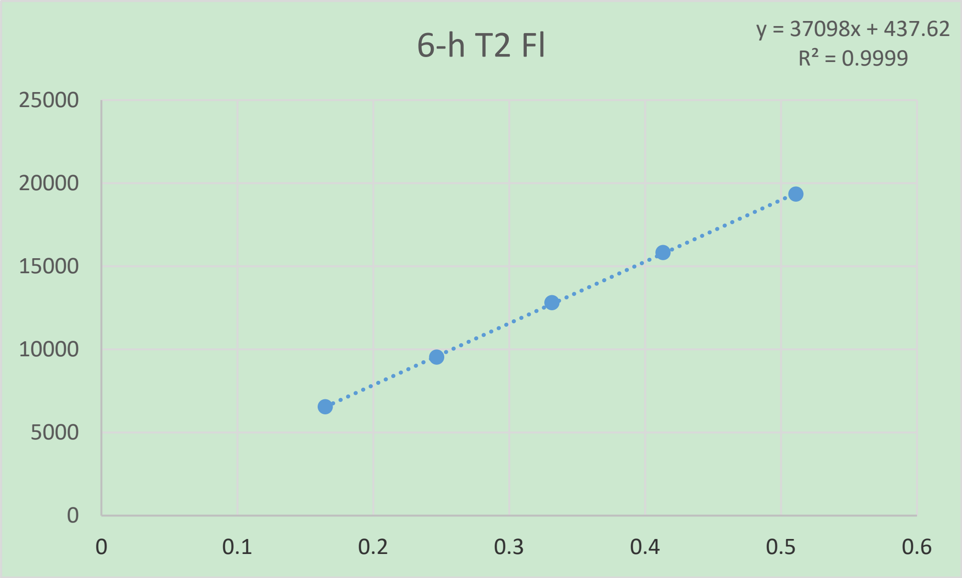
6-h T2 Fl
y = 37098x + 437.62
R² = 0.9999
25000
20000
15000
10000
5000
0
0
0.1
0.2
0.3
0.4
0.5
0.6

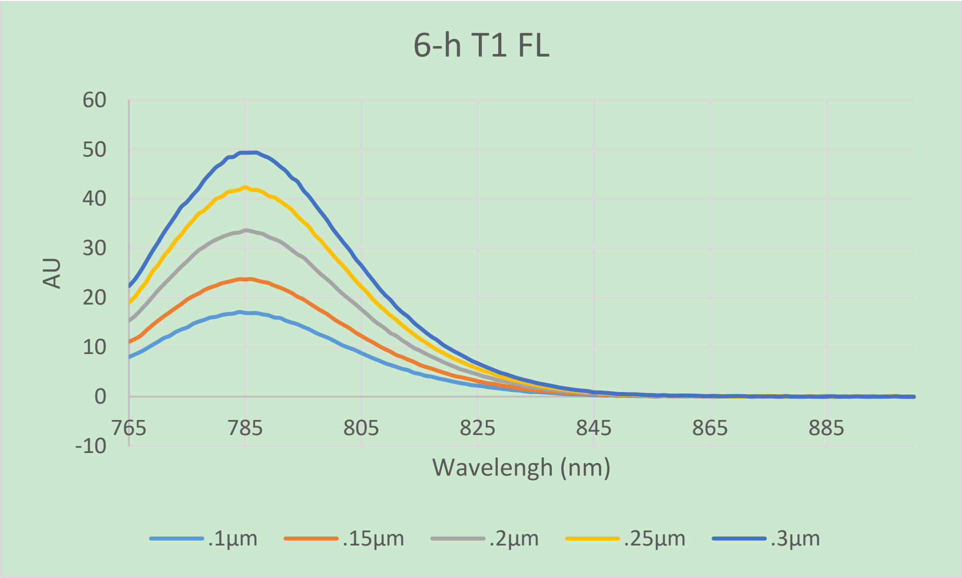
6-h T1 FL
60
50
40
30
20
10
0
-10
AU
765
785
805
825
845
865
885
Wavelengh (nm)
.1µm
.15µm
.2µm
.25µm
.3µm

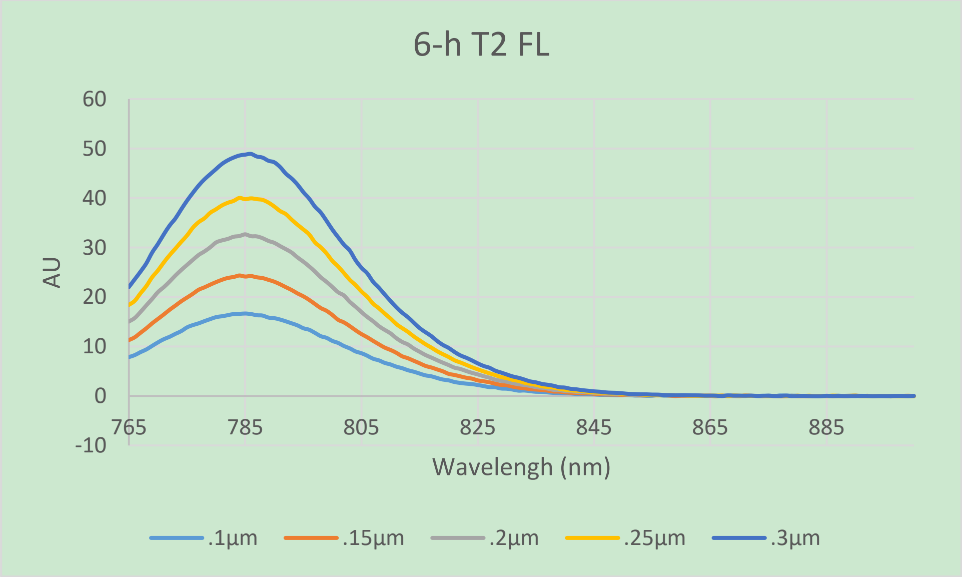
6-h T2 FL
60
50
40
30
20
10
0
-10
AU
765
785
805
825
845
865
885
Wavelengh (nm)
.1µm
.15µm
.2µm
.25µm
.3µm

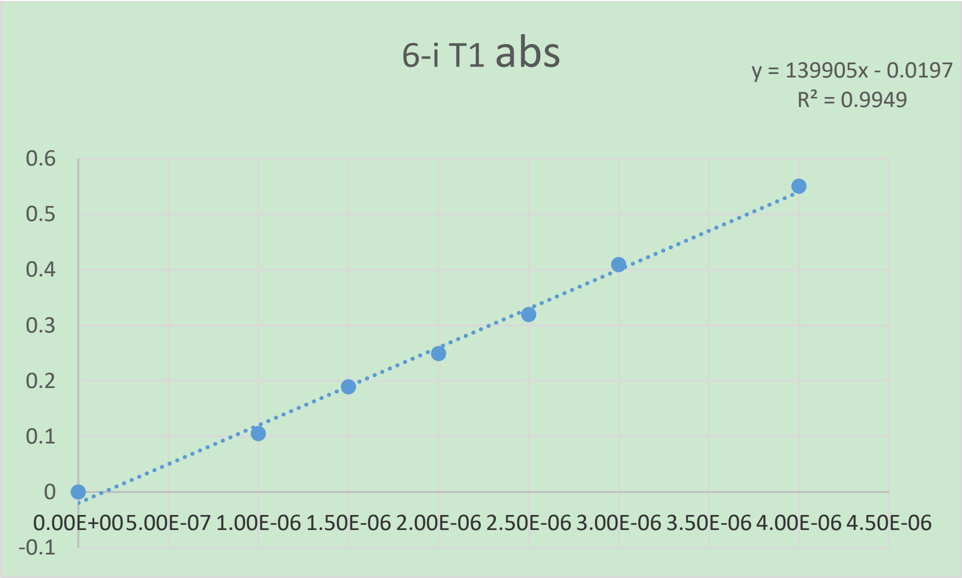
6-i T1 abs
y = 139905x - 0.0197
R² = 0.9949
0.6
0.5
0.4
0.3
0.2
0.1
0
-0.1
0.00E+00 5.00E-07 1.00E-06 1.50E-06 2.00E-06 2.50E-06 3.00E-06 3.50E-06 4.00E-06 4.50E-06

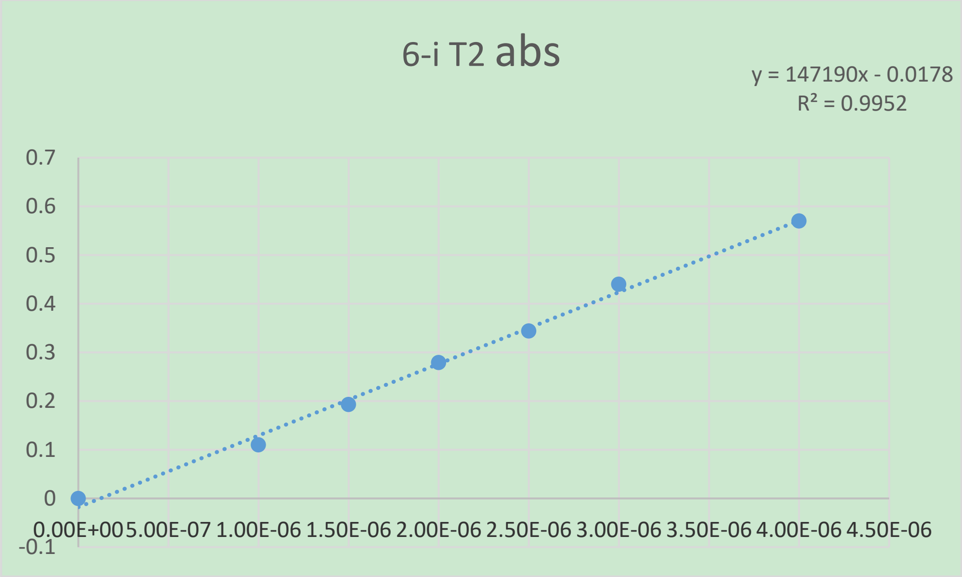
6-i T2 abs
y = 147190x - 0.0178
R² = 0.9952
0.7
0.6
0.5
0.4
0.3
0.2
0.1
0
-0.1
0.00E+00 5.00E-07 1.00E-06 1.50E-06 2.00E-06 2.50E-06 3.00E-06 3.50E-06 4.00E-06 4.50E-06

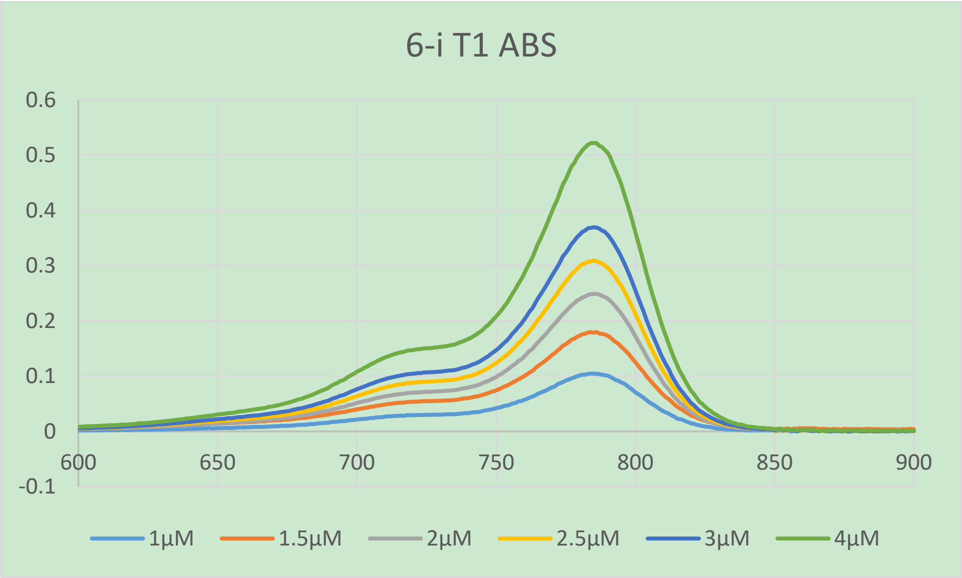
6-i T1 ABS
0.6
0.5
0.4
0.3
0.2
0.1
0
-0.1
600
650
700
750
800
850
900
1µM
1.5µM
2µM
2.5µM
3µM
4µM

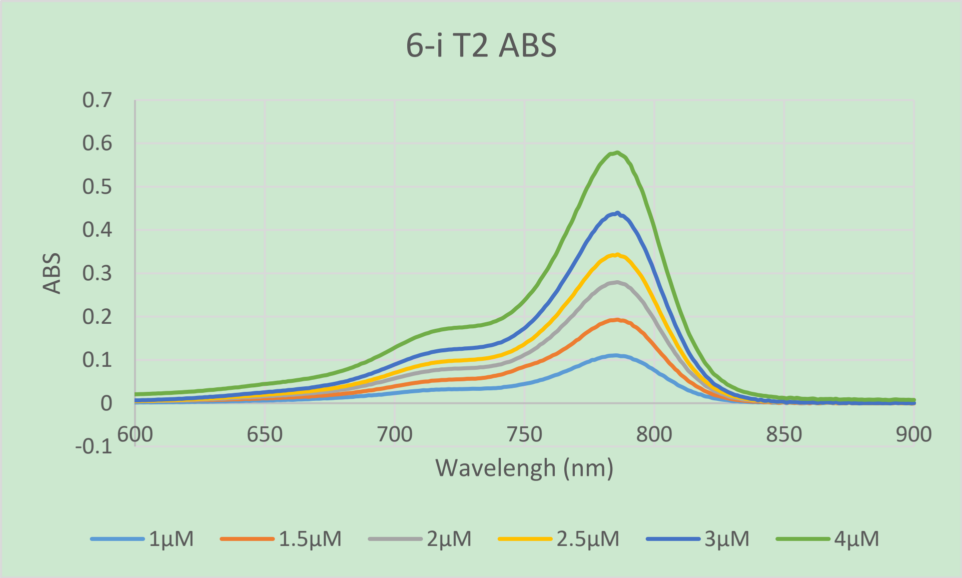
6-i T2 ABS
ABS
0.7
0.6
0.5
0.4
0.3
0.2
0.1
0
-0.1
600
650
700
750
800
850
900
Wavelengh (nm)
1µM
1.5µM
2µM
2.5µM
3µM
4µM

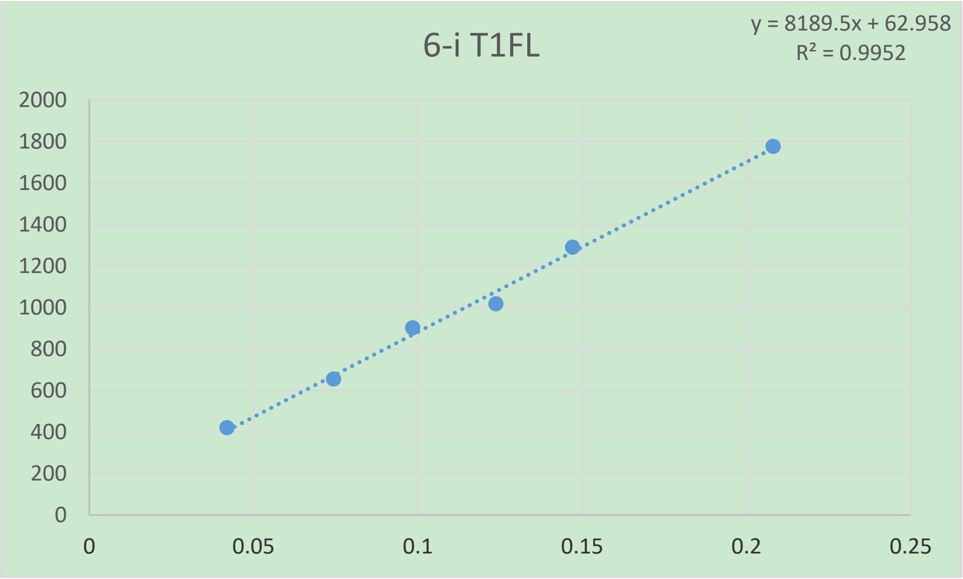
6-i T1FL
y = 8189.5x + 62.958
R² = 0.9952
2000
1800
1600
1400
1200
1000
800
600
400
200
0
0
0.05
0.1
0.15
0.2
0.25

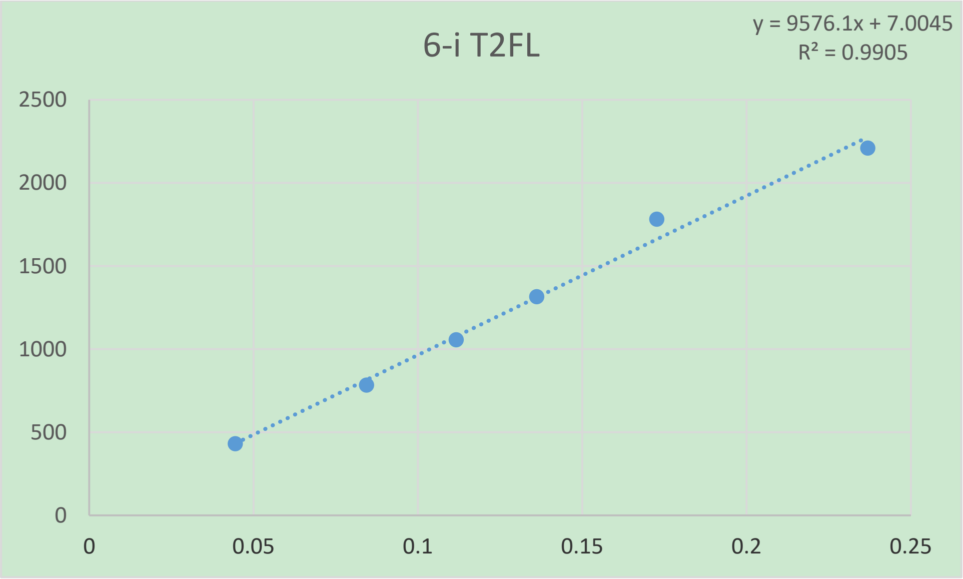
6-i T2FL
y = 9576.1x + 7.0045
R² = 0.9905
2500
2000
1500
1000
500
0
0
0.05
0.1
0.15
0.2
0.25

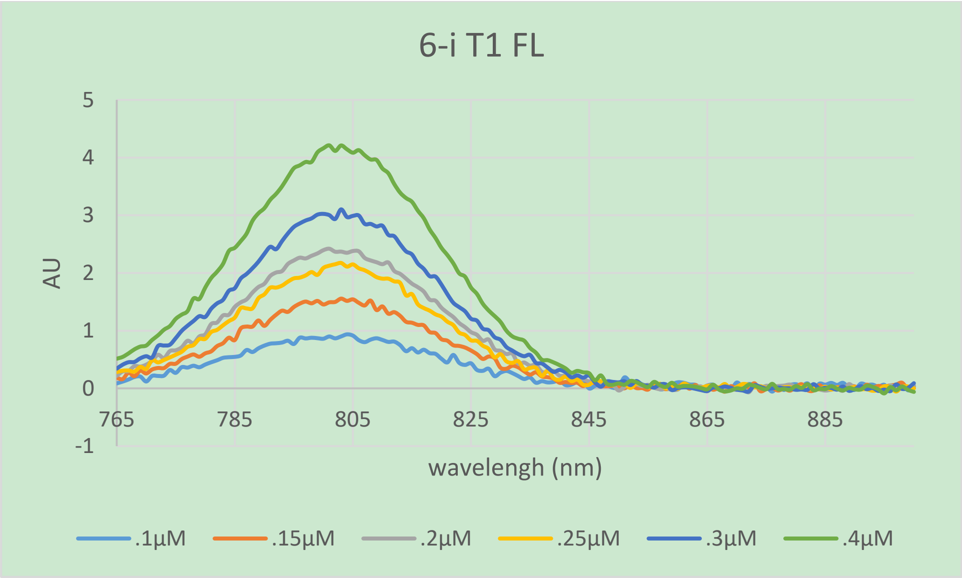
6-i T1 FL
AU
5
4
3
2
1
0
-1
765
785
805
825
845
865
885
wavelengh (nm)
.1μM
.15μM
.2μM
.25μM
.3μM
.4μM

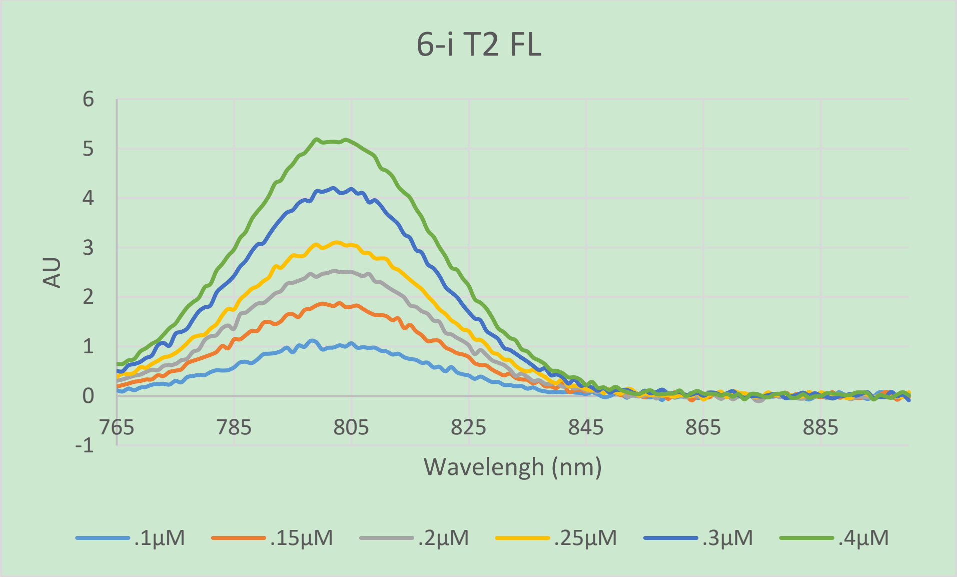
6-i T2 FL
AU
6
5
4
3
2
1
0
-1
765
785
805
825
845
865
885
Wavelengh (nm)
.1μM
.15μM
.2μM
.25μM
.3μM
.4μM

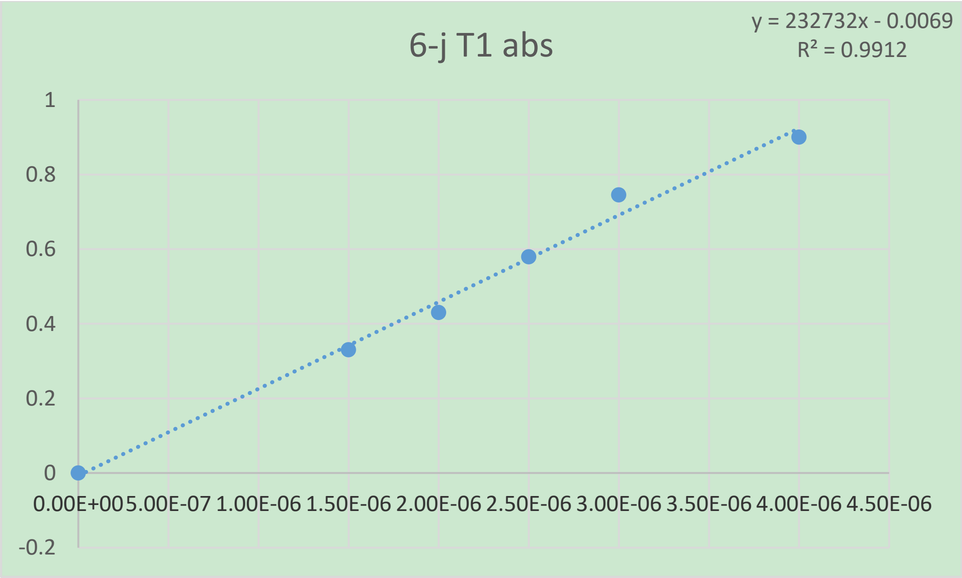

6-j T1 abs
y = 232732x - 0.0069
R² = 0.9912
1
0.8
0.6
0.4
0.2
0
-0.2
0.00E+00
5.00E-07
1.00E-06
1.50E-06
2.00E-06
2.50E-06
3.00E-06
3.50E-06
4.00E-06
4.50E-06

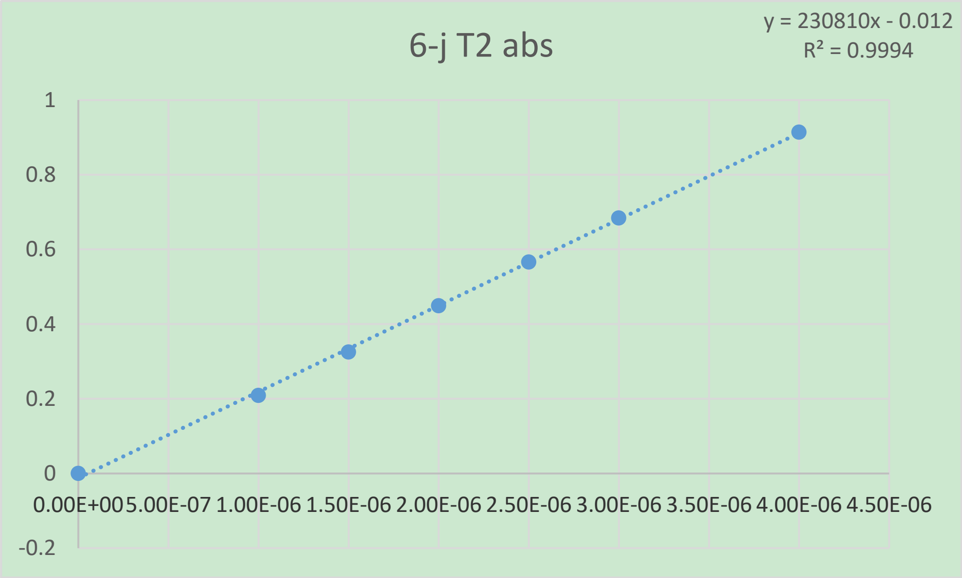

6-j T2 abs
y = 230810x - 0.012
R² = 0.9994
1
0.8
0.6
0.4
0.2
0
-0.2
0.00E+00
5.00E-07
1.00E-06
1.50E-06
2.00E-06
2.50E-06
3.00E-06
3.50E-06
4.00E-06
4.50E-06

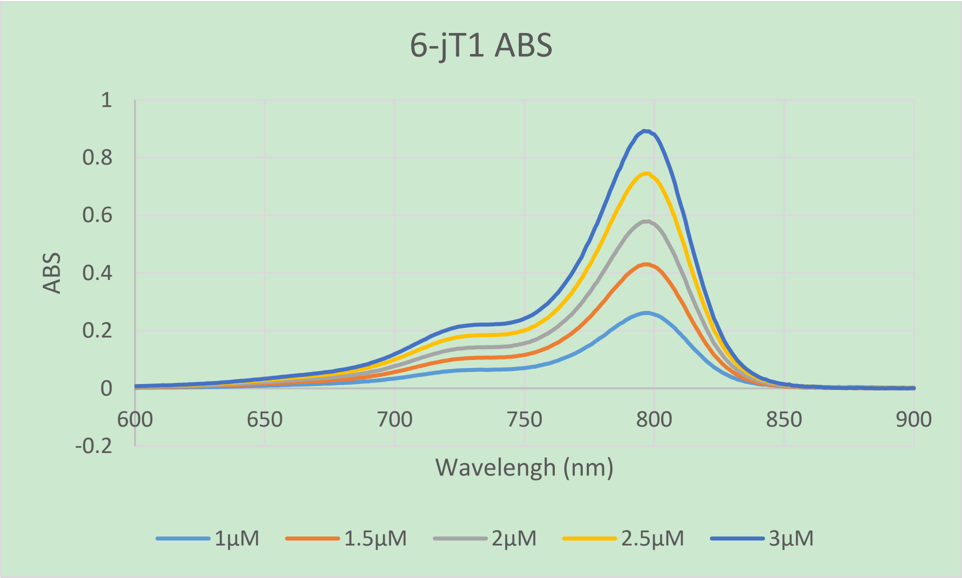
6-jT1 ABS
1
0.8
0.6
0.4
0.2
0
-0.2
ABS
600
650
700
750
800
850
900
Wavelengh (nm)
1µM
1.5µM
2µM
2.5µM
3µM

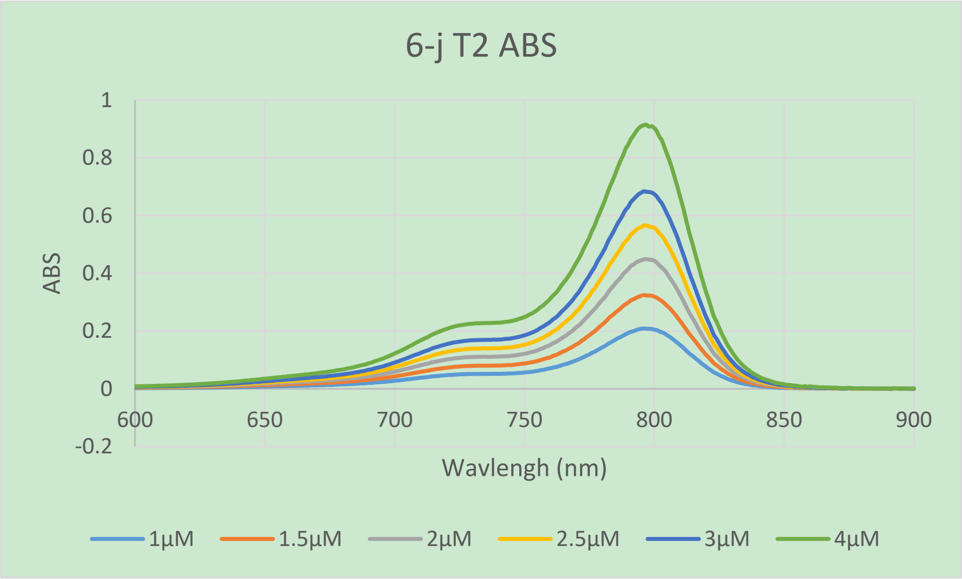
6-j T2 ABS
1
0.8
0.6
0.4
0.2
0
-0.2
ABS
600
650
700
750
800
850
900
Wavlengh (nm)
1µM
1.5µM
2µM
2.5µM
3µM
4µM

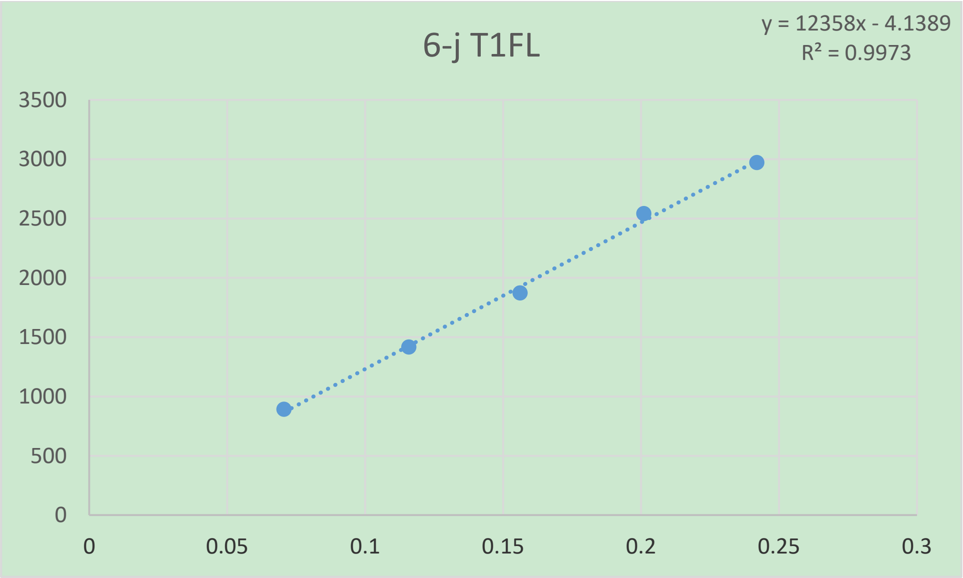
6-j T1FL
y = 12358x - 4.1389
R² = 0.9973
3500
3000
2500
2000
1500
1000
500
0
0
0.05
0.1
0.15
0.2
0.25
0.3

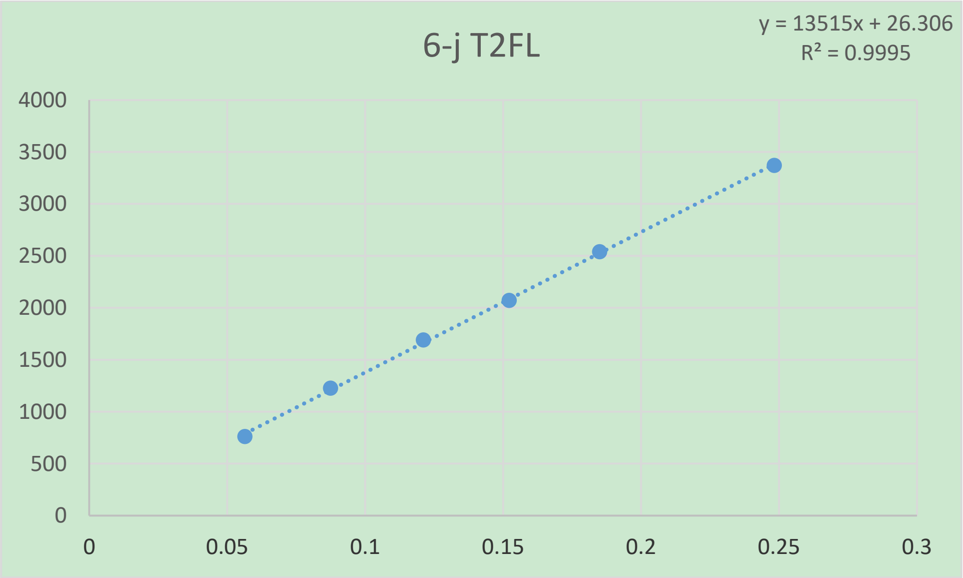
6-j T2FL
y = 13515x + 26.306
R² = 0.9995
4000
3500
3000
2500
2000
1500
1000
500
0
0
0.05
0.1
0.15
0.2
0.25
0.3

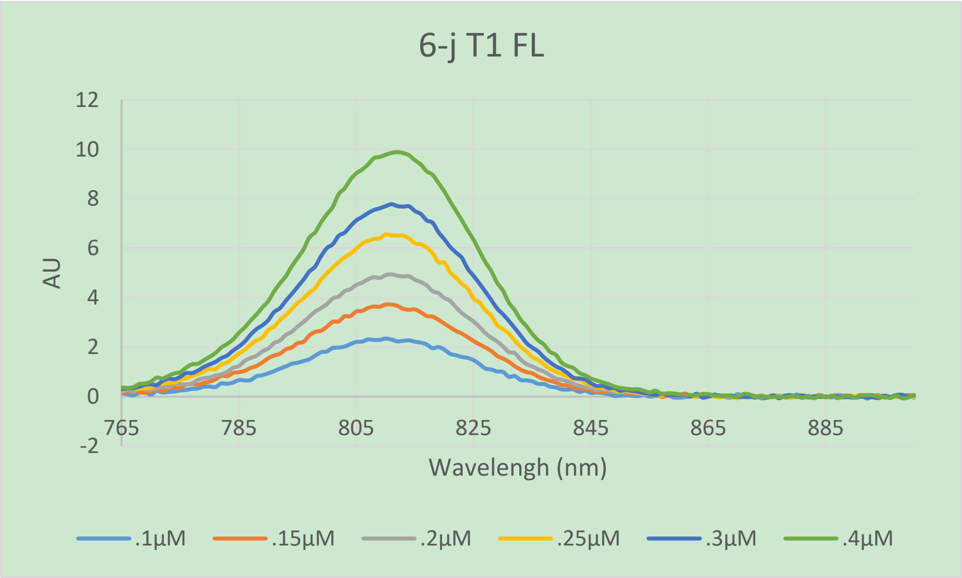
6-j T1 FL
12
10
8
6
4
2
0
-2
AU
765
785
805
825
845
865
885
Wavelengh (nm)
.1µM
.15µM
.2µM
.25µM
.3µM
.4µM

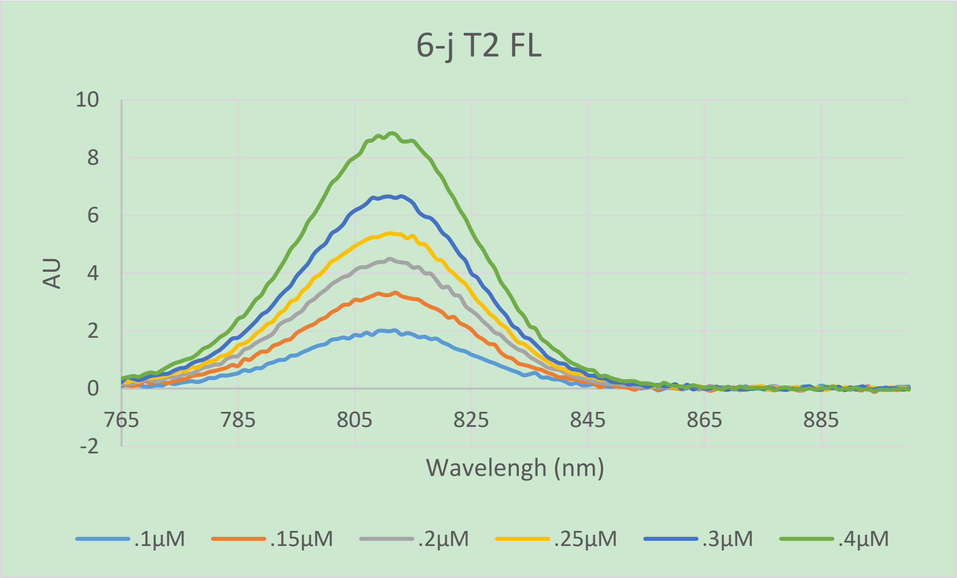
6-j T2 FL
10
8
6
4
2
0
-2
AU
765
785
805
825
845
865
885
Wavelengh (nm)
.1µM
.15µM
.2µM
.25µM
.3µM
.4µM

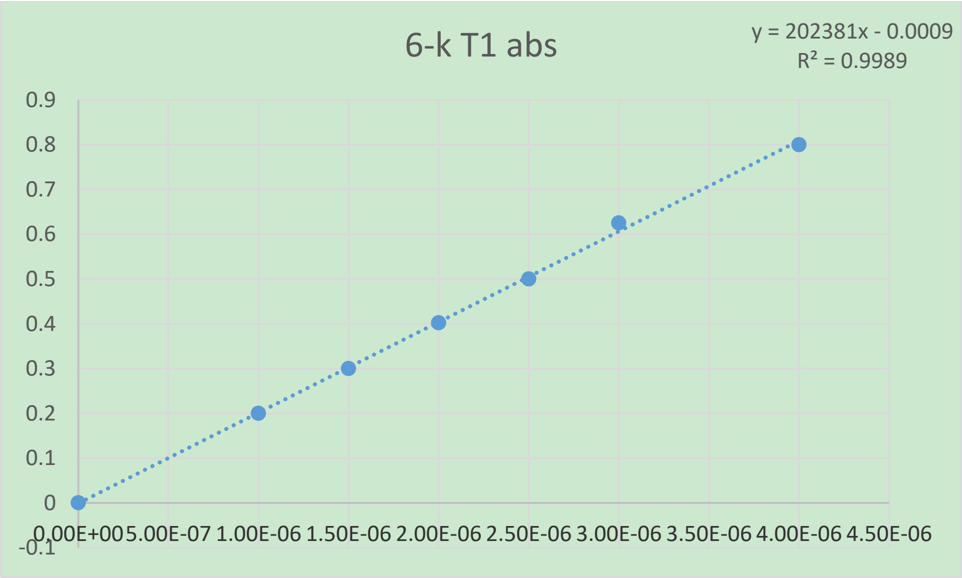
6-k T1 abs
y = 202381x - 0.0009
R² = 0.9989
0.9
0.8
0.7
0.6
0.5
0.4
0.3
0.2
0.1
0
-0.1
0.00E+00 5.00E-07 1.00E-06 1.50E-06 2.00E-06 2.50E-06 3.00E-06 3.50E-06 4.00E-06 4.50E-06

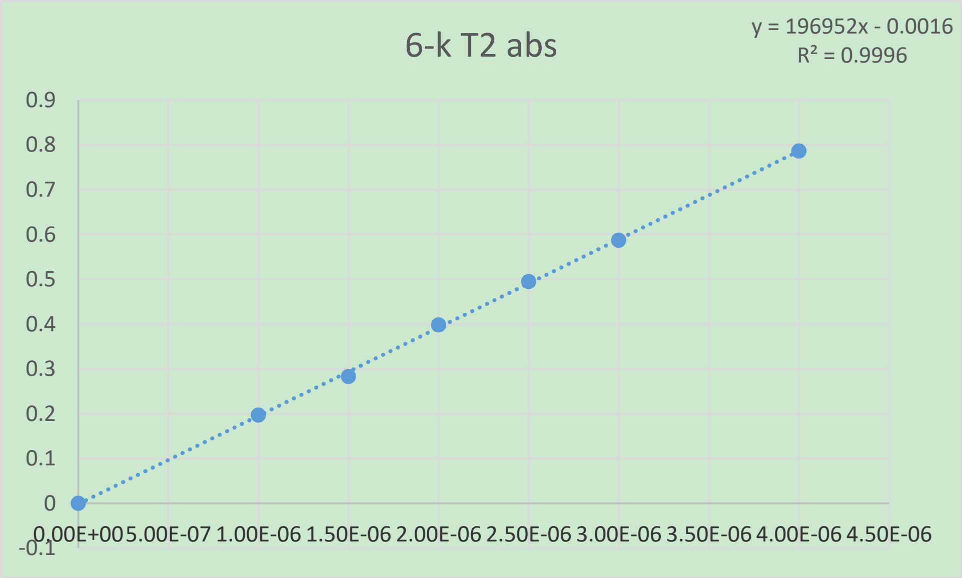
6-k T2 abs
y = 196952x - 0.0016
R² = 0.9996
0.9
0.8
0.7
0.6
0.5
0.4
0.3
0.2
0.1
0
-0.1
0.00E+00 5.00E-07 1.00E-06 1.50E-06 2.00E-06 2.50E-06 3.00E-06 3.50E-06 4.00E-06 4.50E-06

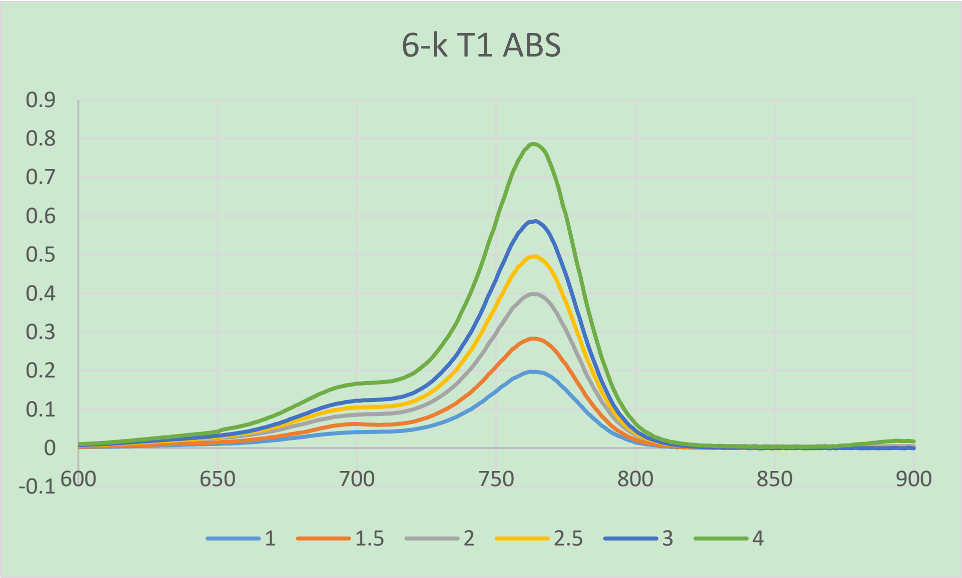
6-k T1 ABS
0.9
0.8
0.7
0.6
0.5
0.4
0.3
0.2
0.1
0
-0.1
600
650
700
750
800
850
900
1
1.5
2
2.5
3
4

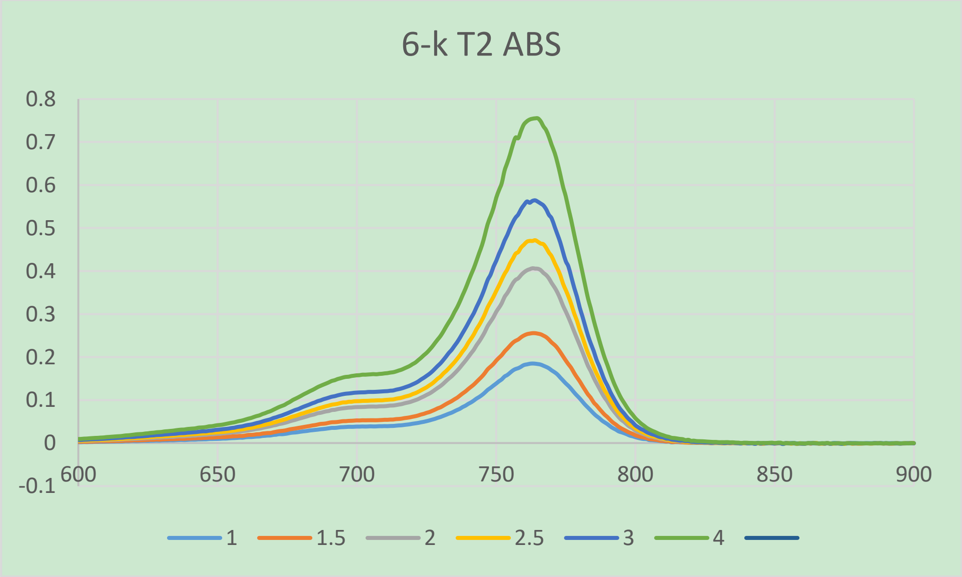
6-k T2 ABS
0.8
0.7
0.6
0.5
0.4
0.3
0.2
0.1
0
-0.1
600
650
700
750
800
850
900
1
1.5
2
2.5
3
4

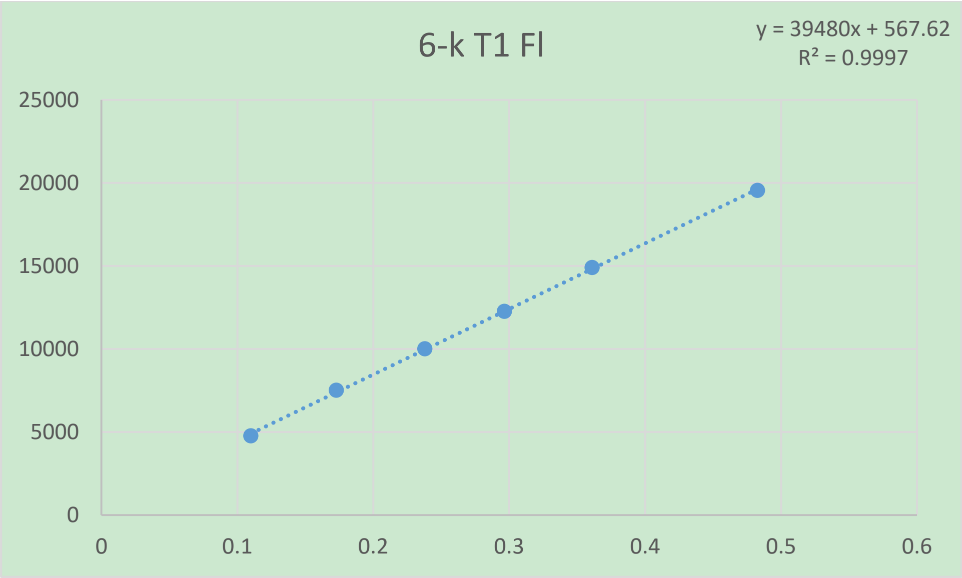
6-k T1 Fl
y = 39480x + 567.62
R² = 0.9997
25000
20000
15000
10000
5000
0
0
0.1
0.2
0.3
0.4
0.5
0.6

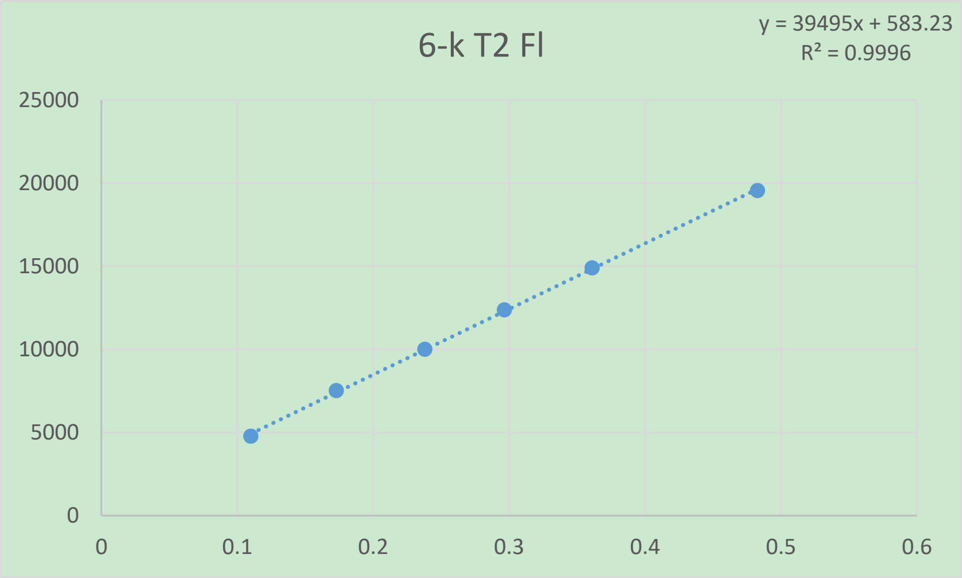
6-k T2 Fl
y = 39495x + 583.23
R² = 0.9996
25000
20000
15000
10000
5000
0
0
0.1
0.2
0.3
0.4
0.5
0.6

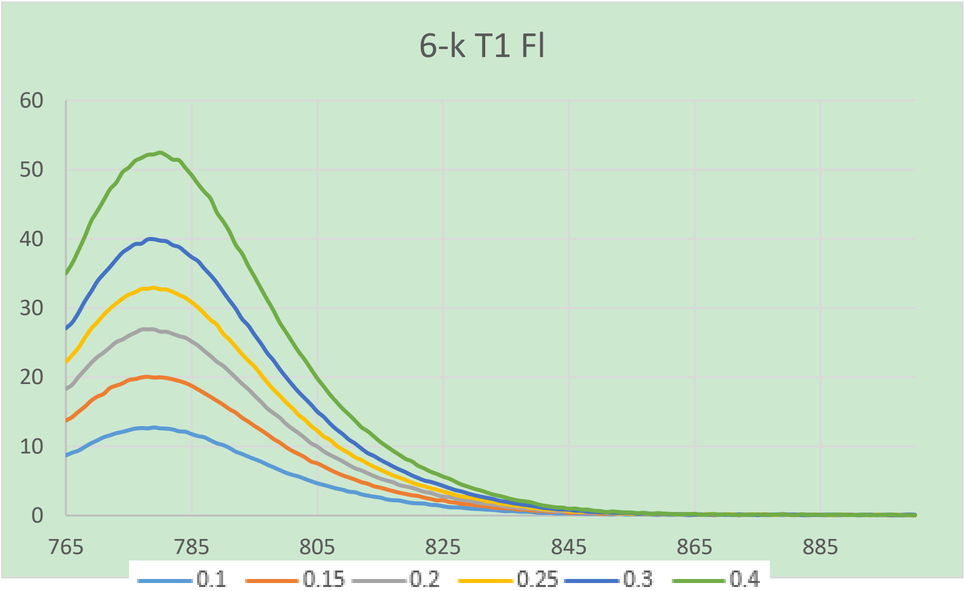
6-k T1 Fl
60
50
40
30
20
10
0
765
785
805
825
845
865
885
0.1
0.15
0.2
0.25
0.3
0.4

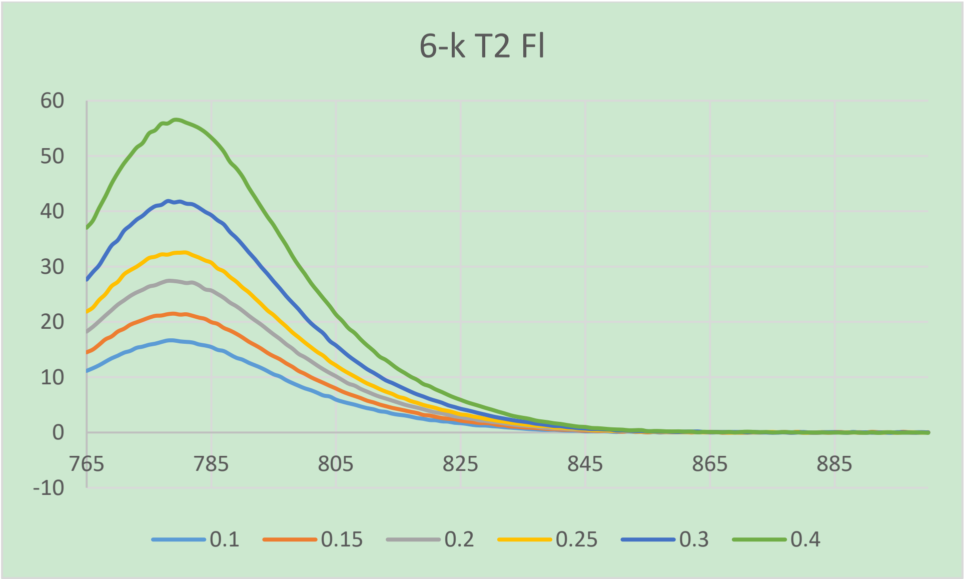
6-k T2 Fl
60
50
40
30
20
10
0
-10
765
785
805
825
845
865
885
0.1
0.15
0.2
0.25
0.3
0.4

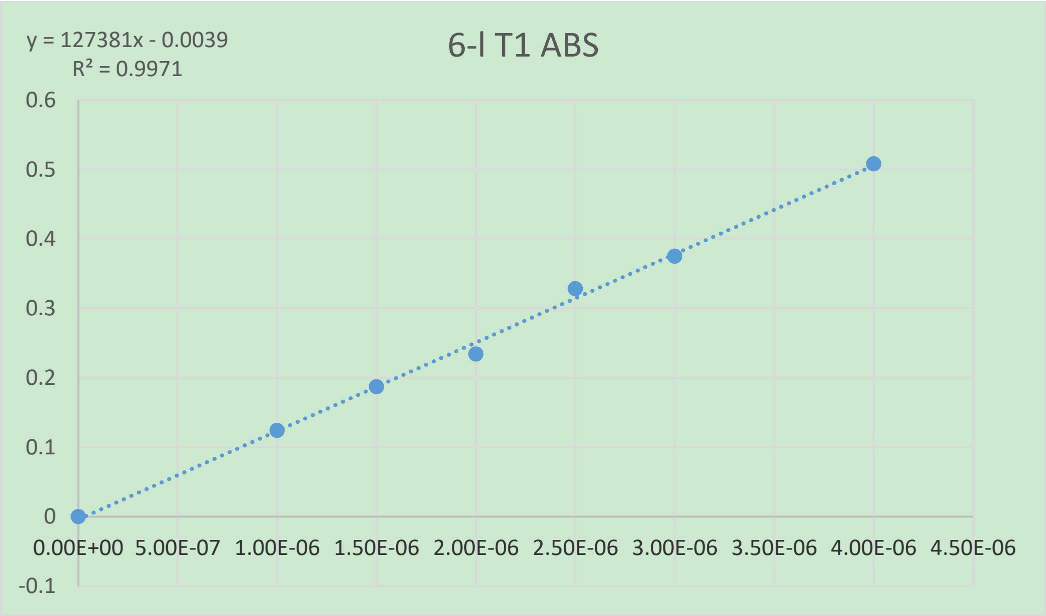
y = 127381x - 0.0039
R² = 0.9971
6-l T1 ABS
0.6
0.5
0.4
0.3
0.2
0.1
0
-0.1
0.00E+00 5.00E-07 1.00E-06 1.50E-06 2.00E-06 2.50E-06 3.00E-06 3.50E-06 4.00E-06 4.50E-06

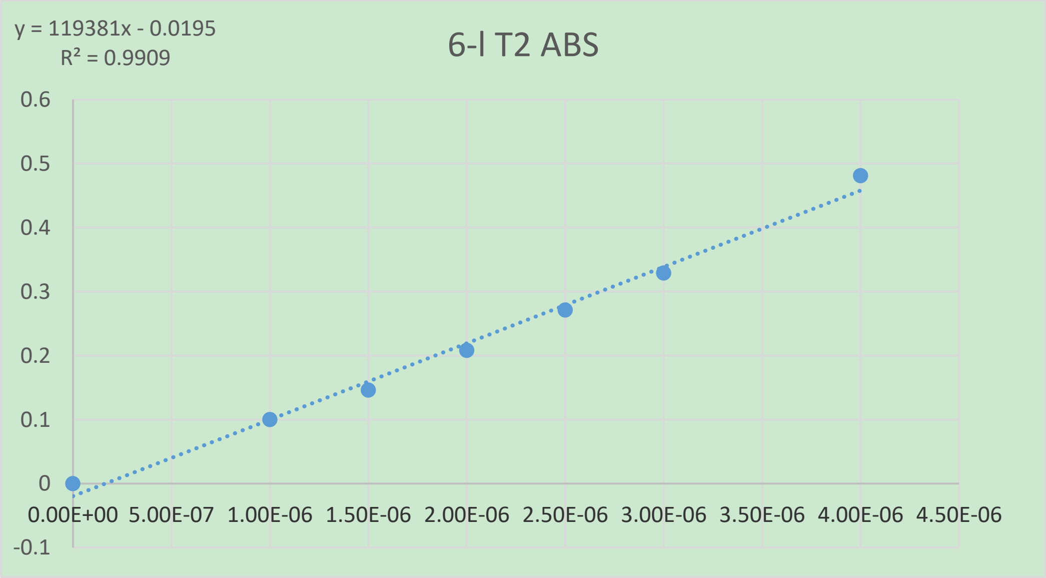
y = 119381x - 0.0195
R² = 0.9909
6-l T2 ABS
0.6
0.5
0.4
0.3
0.2
0.1
0
-0.1
0.00E+00 5.00E-07 1.00E-06 1.50E-06 2.00E-06 2.50E-06 3.00E-06 3.50E-06 4.00E-06 4.50E-06

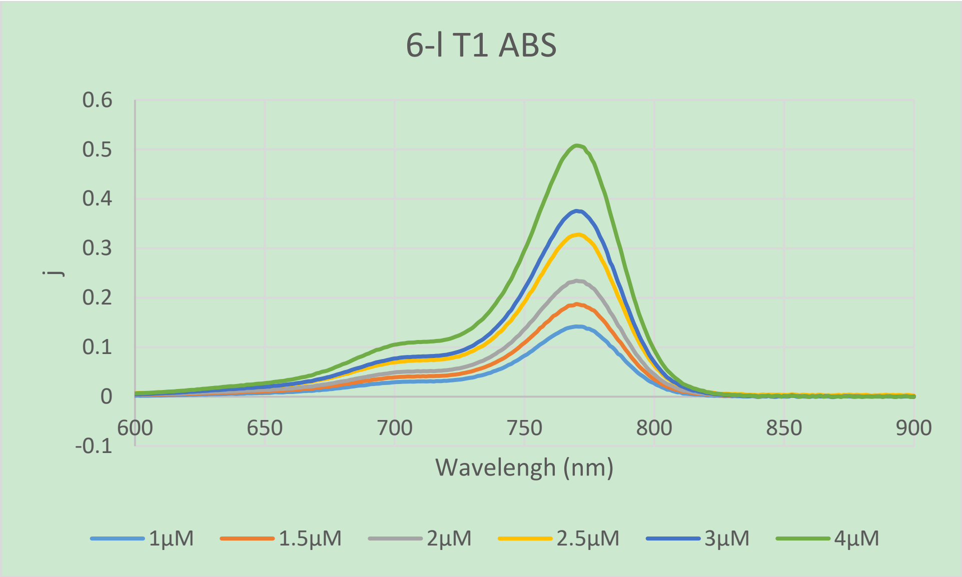
6-l T1 ABS
0.6
0.5
0.4
0.3
0.2
0.1
0
-0.1
j
600
650
700
750
800
850
900
Wavelengh (nm)
1μM
1.5μM
2μM
2.5μM
3μM
4μM

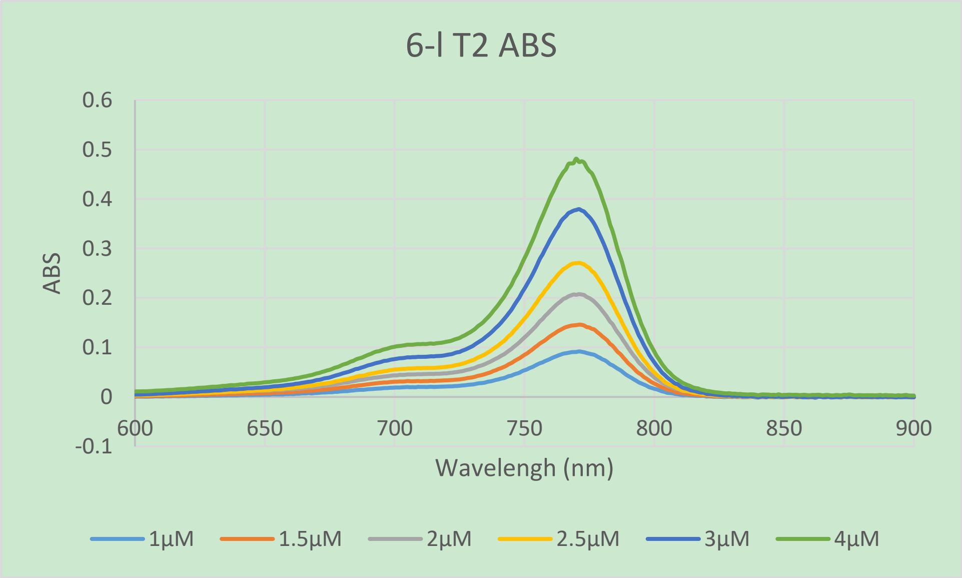
6-l T2 ABS
0.6
0.5
0.4
0.3
0.2
0.1
0
-0.1
ABS
600
650
700
750
800
850
900
Wavelengh (nm)
1μM
1.5μM
2μM
2.5μM
3μM
4μM

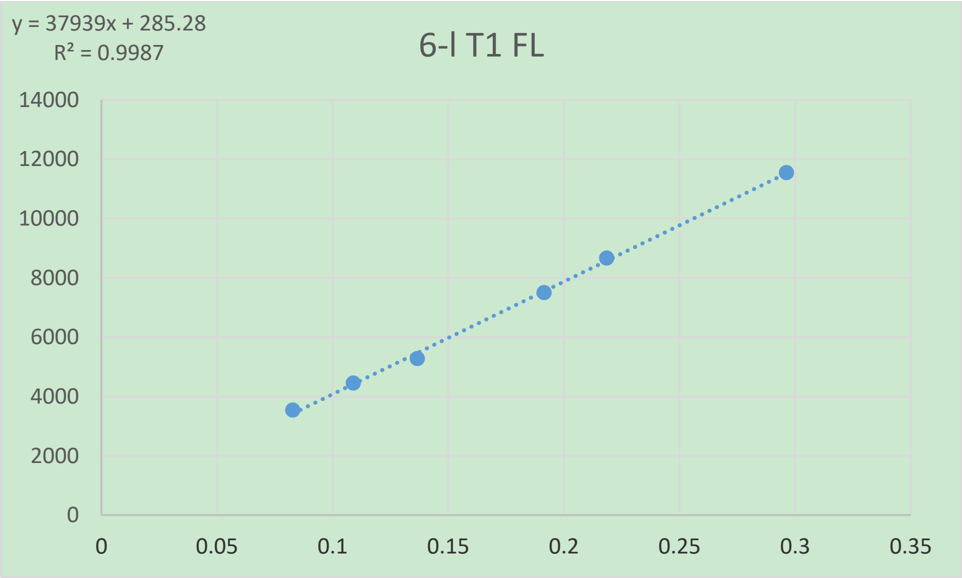
y = 37939x + 285.28
R² = 0.9987
6-l T1 FL
14000
12000
10000
8000
6000
4000
2000
0
0
0.05
0.1
0.15
0.2
0.25
0.3
0.35

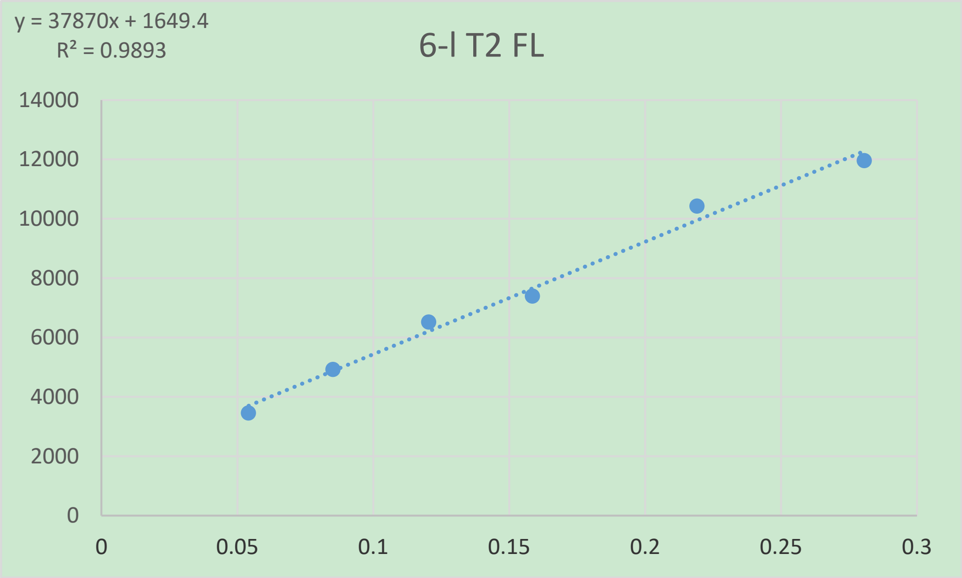
y = 37870x + 1649.4
R² = 0.9893
6-l T2 FL
14000
12000
10000
8000
6000
4000
2000
0
0
0.05
0.1
0.15
0.2
0.25
0.3

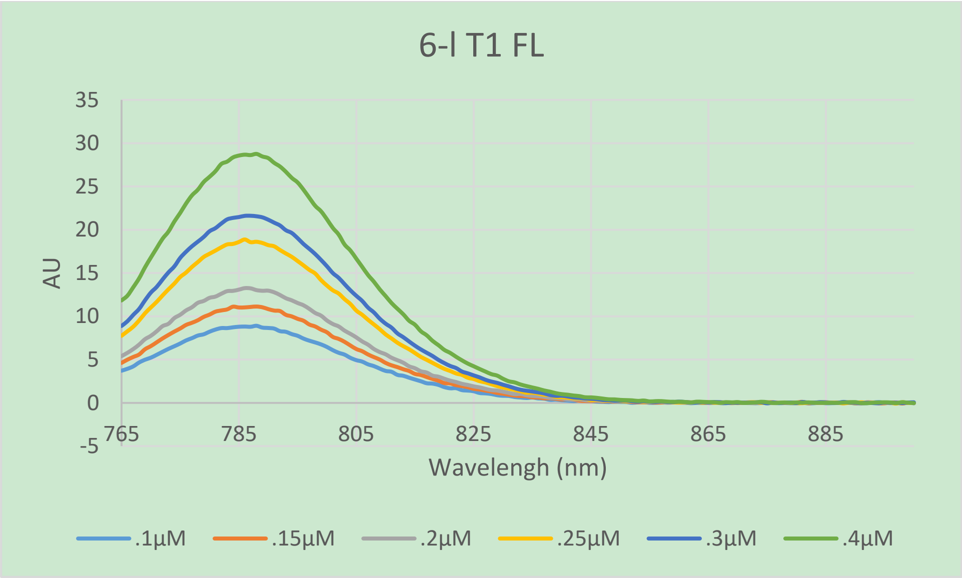
6-l T1 FL
AU
Wavelengh (nm)
35
30
25
20
15
10
5
0
-5
765
785
805
825
845
865
885
.1µM
.15µM
.2µM
.25µM
.3µM
.4µM

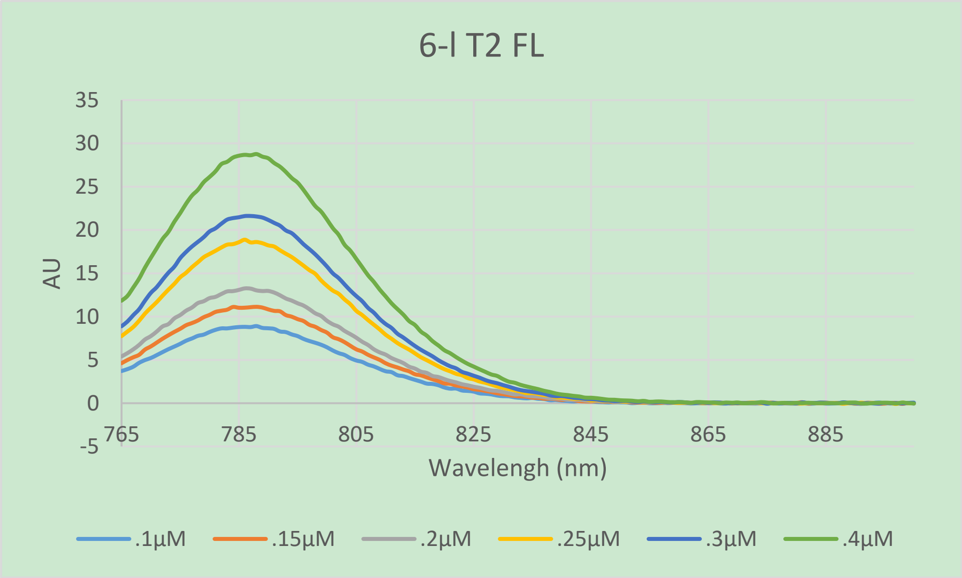
6-l T2 FL
AU
Wavelengh (nm)
35
30
25
20
15
10
5
0
-5
765
785
805
825
845
865
885
.1µM
.15µM
.2µM
.25µM
.3µM
.4µM

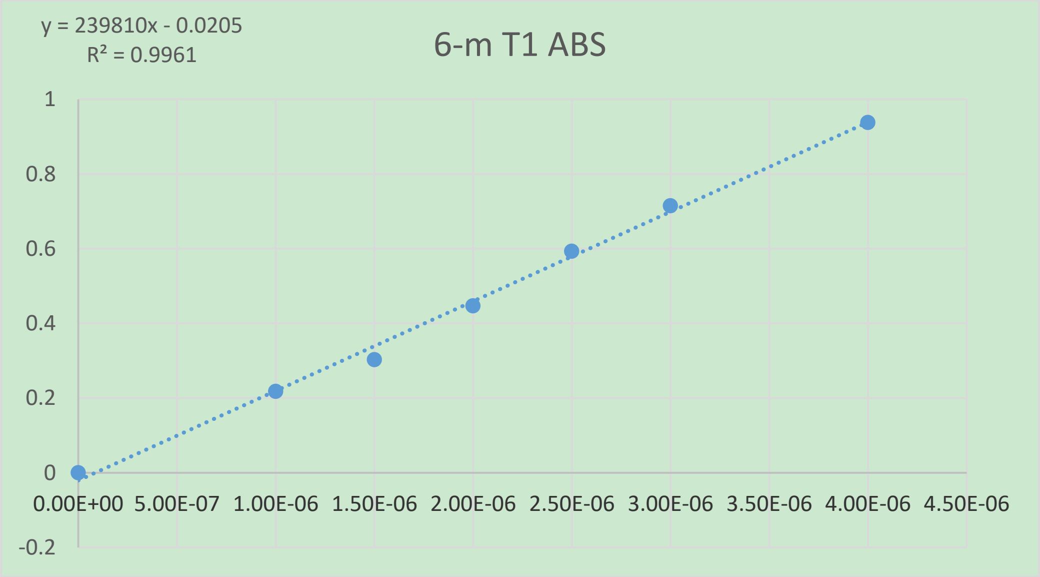
y = 239810x - 0.0205
R² = 0.9961
6-m T1 ABS
1
0.8
0.6
0.4
0.2
0
-0.2
0.00E+00
5.00E-07
1.00E-06
1.50E-06
2.00E-06
2.50E-06
3.00E-06
3.50E-06
4.00E-06
4.50E-06

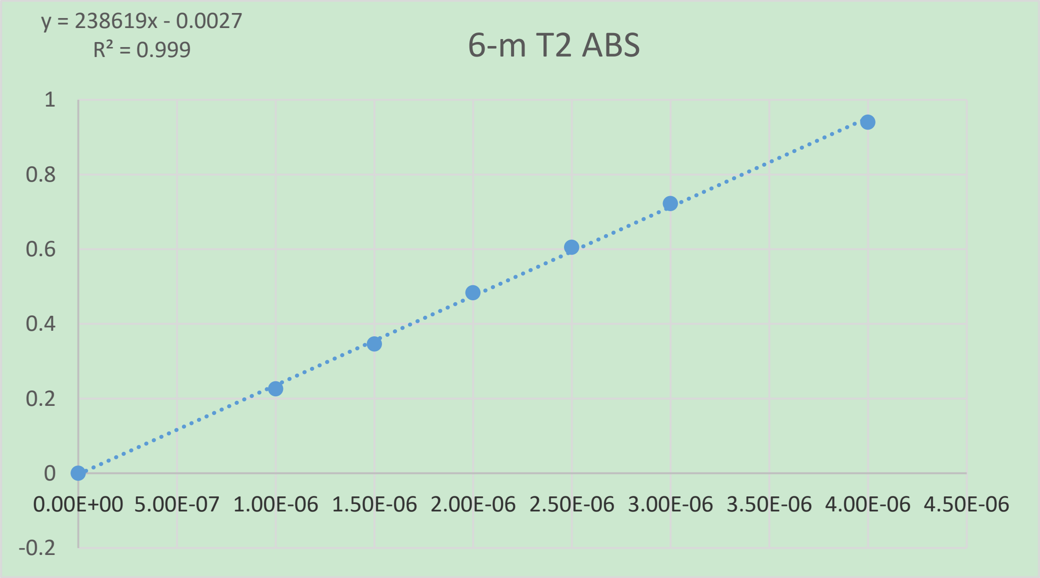
y = 238619x - 0.0027
R² = 0.999
6-m T2 ABS
1
0.8
0.6
0.4
0.2
0
-0.2
0.00E+00
5.00E-07
1.00E-06
1.50E-06
2.00E-06
2.50E-06
3.00E-06
3.50E-06
4.00E-06
4.50E-06

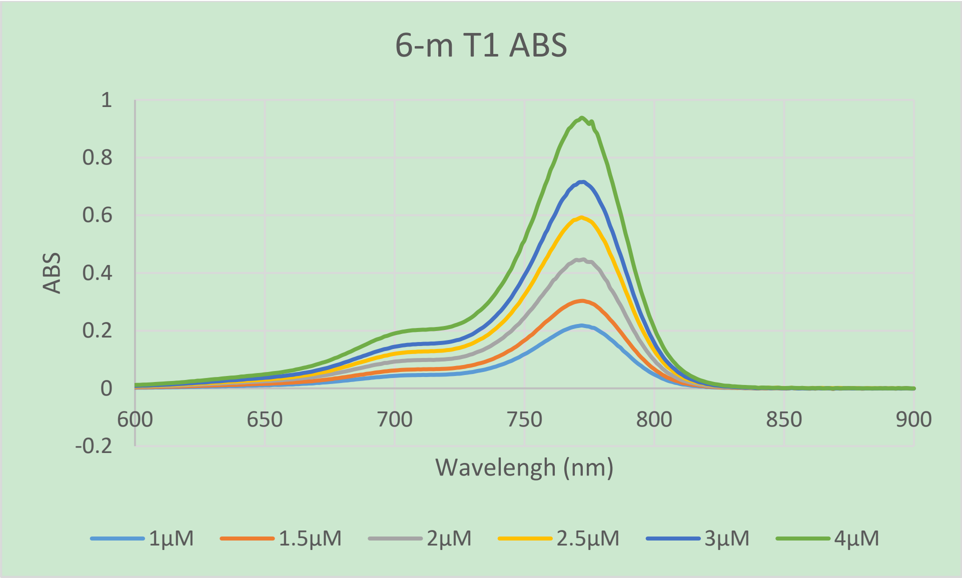
6-m T1 ABS
ABS
1
0.8
0.6
0.4
0.2
0
-0.2
600
650
700
750
800
850
900
Wavelengh (nm)
1µM
1.5µM
2µM
2.5µM
3µM
4µM

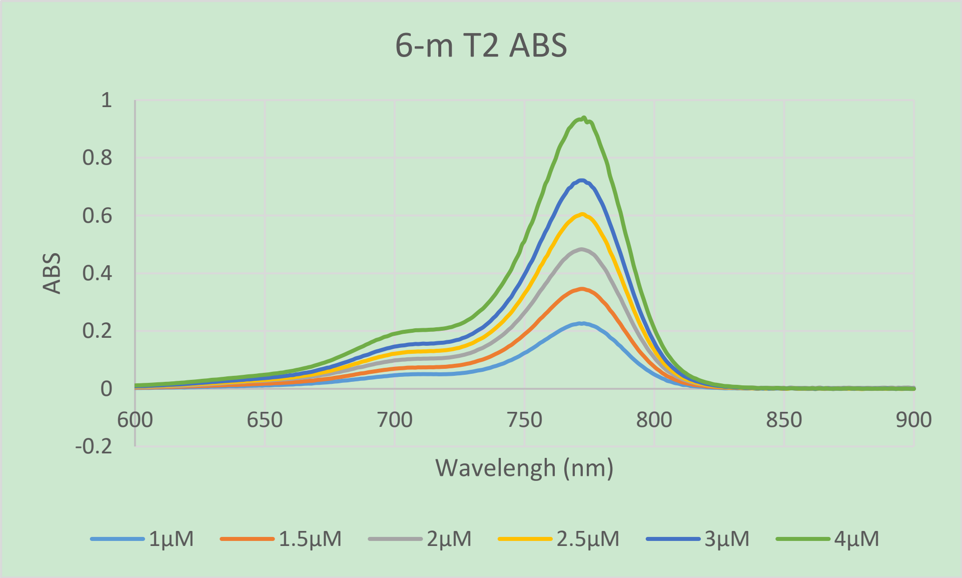
6-m T2 ABS
ABS
1
0.8
0.6
0.4
0.2
0
-0.2
600
650
700
750
800
850
900
Wavelengh (nm)
1µM
1.5µM
2µM
2.5µM
3µM
4µM

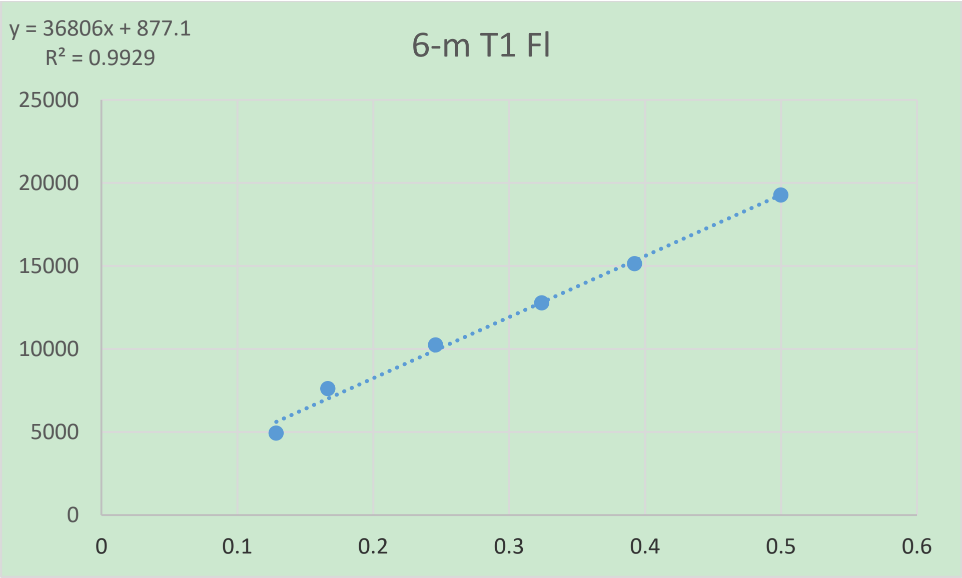
y = 36806x + 877.1
R² = 0.9929
6-m T1 Fl
25000
20000
15000
10000
5000
0
0
0.1
0.2
0.3
0.4
0.5
0.6

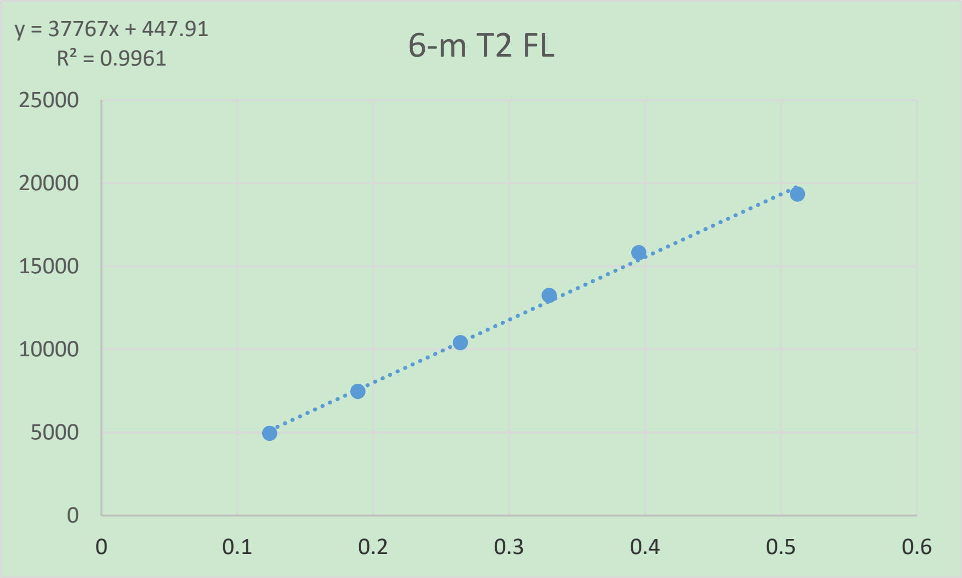
y = 37767x + 447.91
R² = 0.9961
6-m T2 FL
25000
20000
15000
10000
5000
0
0
0.1
0.2
0.3
0.4
0.5
0.6

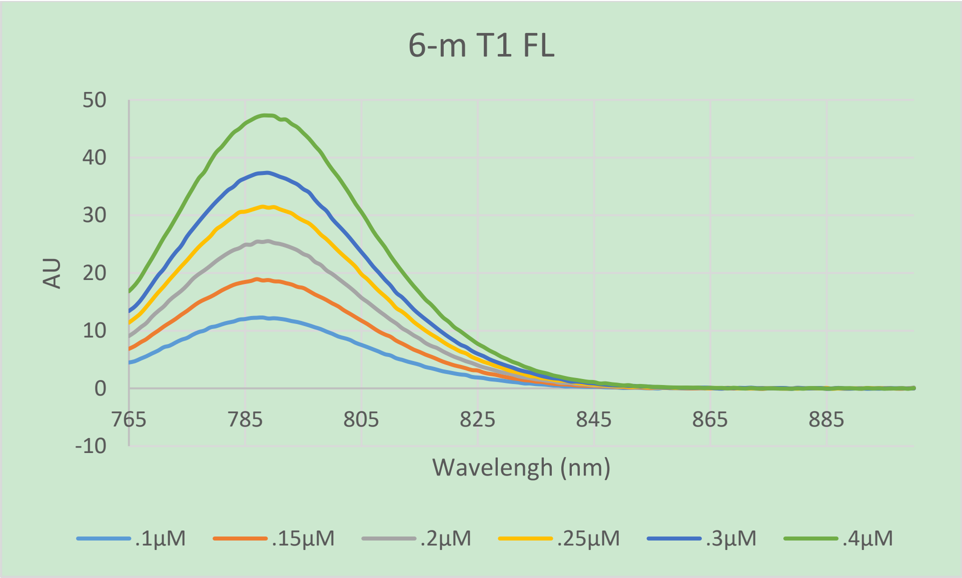
6-m T1 FL
50
40
30
20
10
0
-10
AU
765
785
805
825
845
865
885
Wavelengh (nm)
.1µM
.15µM
.2µM
.25µM
.3µM
.4µM

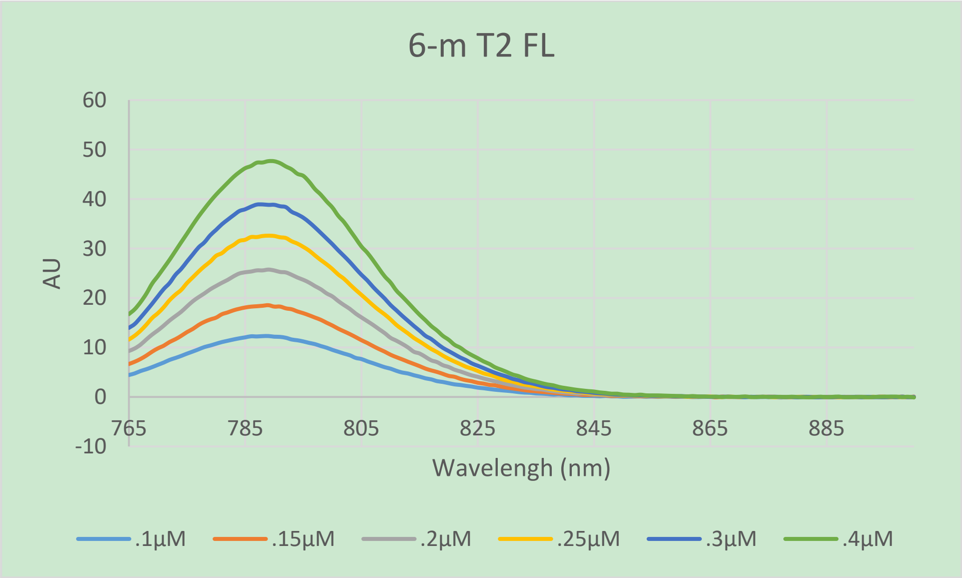
6-m T2 FL
60
50
40
30
20
10
0
-10
AU
765
785
805
825
845
865
885
Wavelengh (nm)
.1µM
.15µM
.2µM
.25µM
.3µM
.4µM

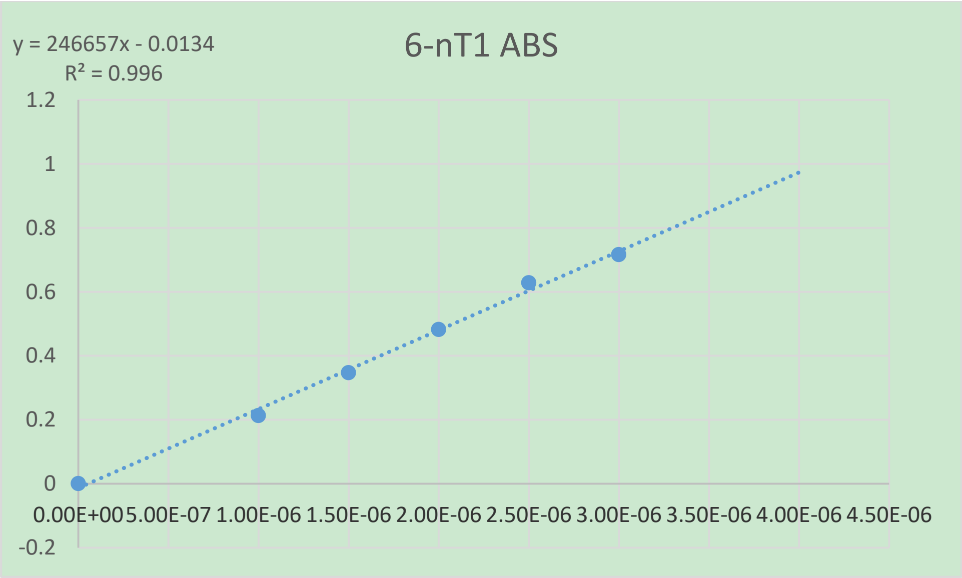
y = 246657x - 0.0134
R² = 0.996
6-nT1 ABS
1.2
1
0.8
0.6
0.4
0.2
0
-0.2
0.00E+00
5.00E-07
1.00E-06
1.50E-06
2.00E-06
2.50E-06
3.00E-06
3.50E-06
4.00E-06
4.50E-06

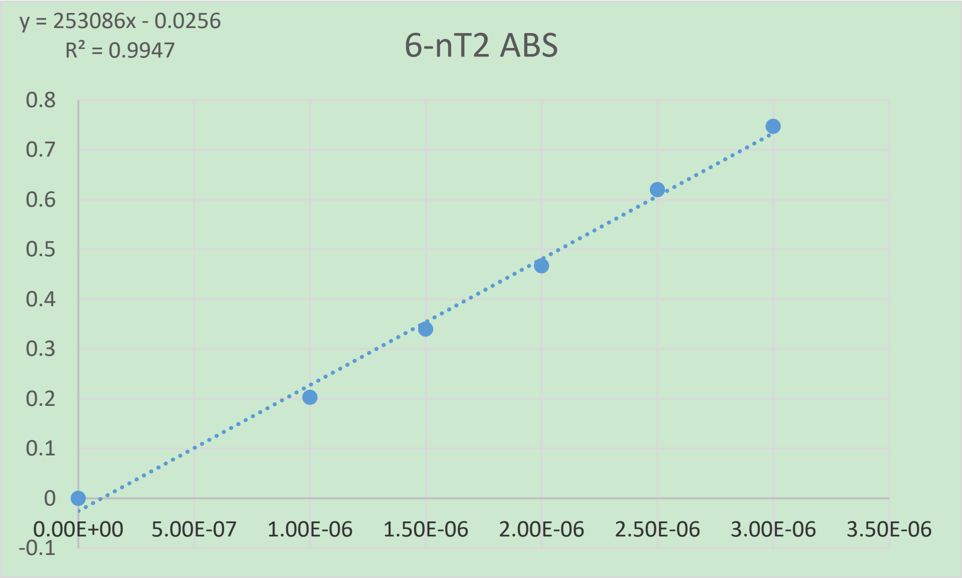
y = 253086x - 0.0256
R² = 0.9947
6-nT2 ABS
0.8
0.7
0.6
0.5
0.4
0.3
0.2
0.1
0
-0.1
0.00E+00
5.00E-07
1.00E-06
1.50E-06
2.00E-06
2.50E-06
3.00E-06
3.50E-06

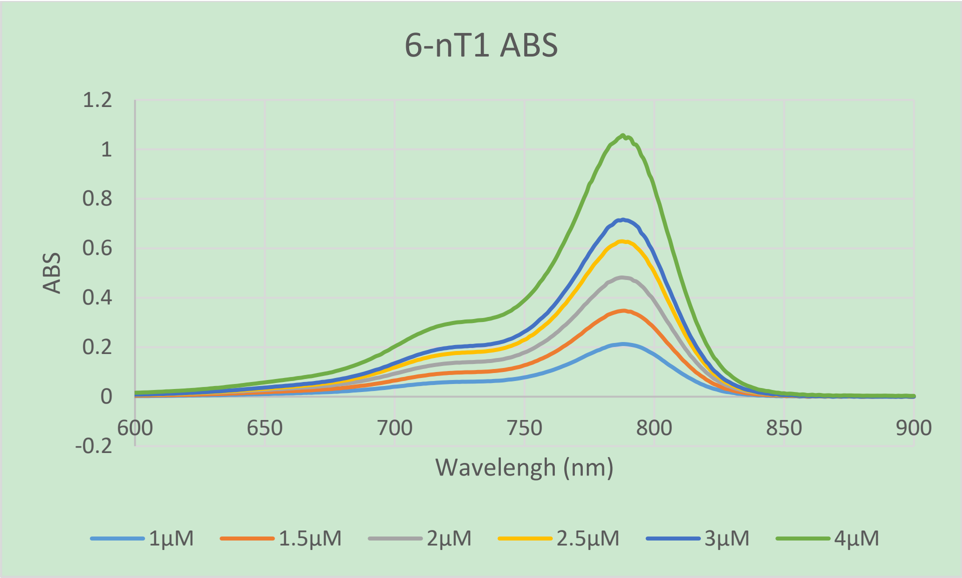
6-nT1 ABS
ABS
1.2
1
0.8
0.6
0.4
0.2
0
-0.2
600
650
700
750
800
850
900
Wavelengh (nm)
1μM
1.5μM
2μM
2.5μM
3μM
4μM

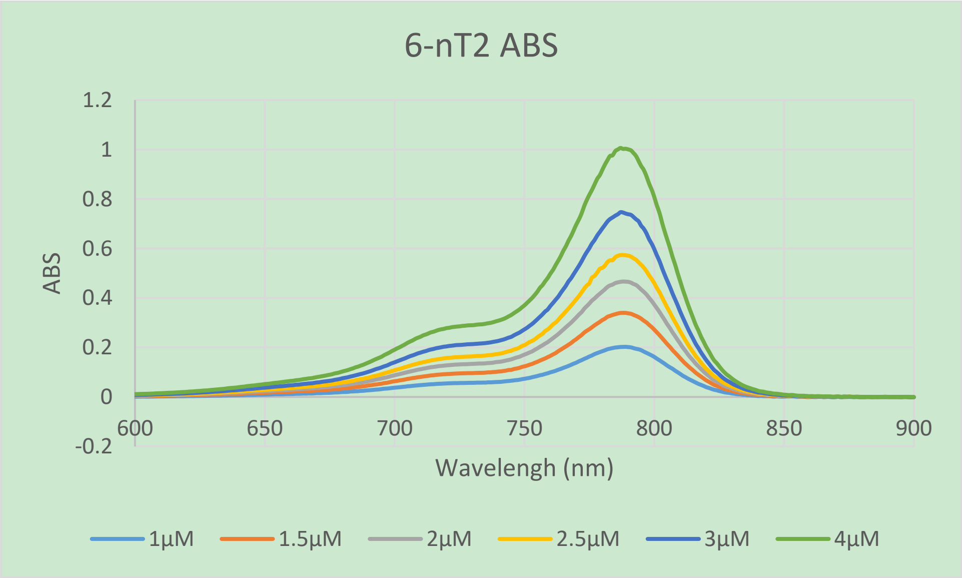
6-nT2 ABS
ABS
1.2
1
0.8
0.6
0.4
0.2
0
-0.2
600
650
700
750
800
850
900
Wavelengh (nm)
1μM
1.5μM
2μM
2.5μM
3μM
4μM

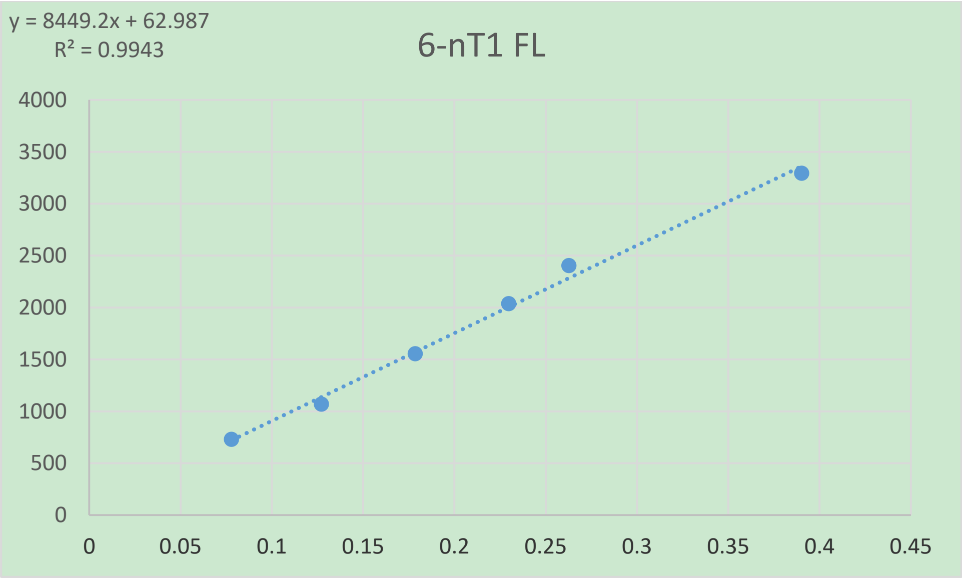
y = 8449.2x + 62.987
R² = 0.9943
6-nT1 FL
4000
3500
3000
2500
2000
1500
1000
500
0
0
0.05
0.1
0.15
0.2
0.25
0.3
0.35
0.4
0.45

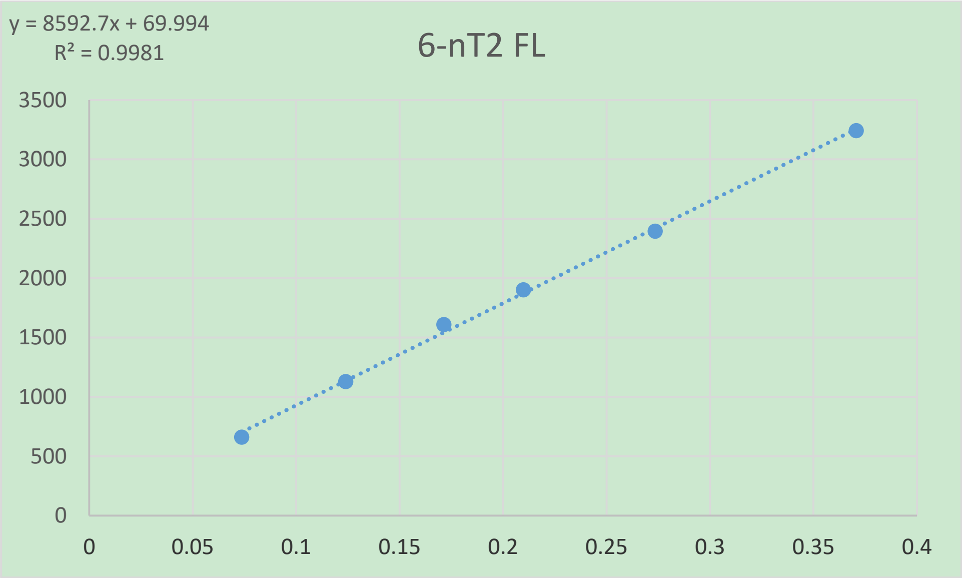
y = 8592.7x + 69.994
R² = 0.9981
6-nT2 FL
3500
3000
2500
2000
1500
1000
500
0
0
0.05
0.1
0.15
0.2
0.25
0.3
0.35
0.4

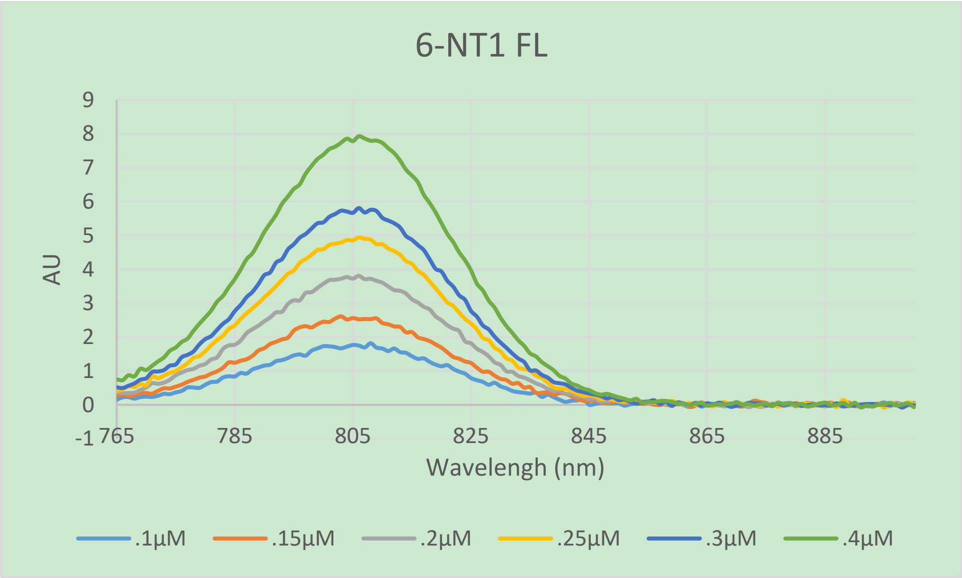
6-NT1 FL
AU
9
8
7
6
5
4
3
2
1
0
-1
765
785
805
825
845
865
885
Wavelengh (nm)
.1µM
.15µM
.2µM
.25µM
.3µM
.4µM

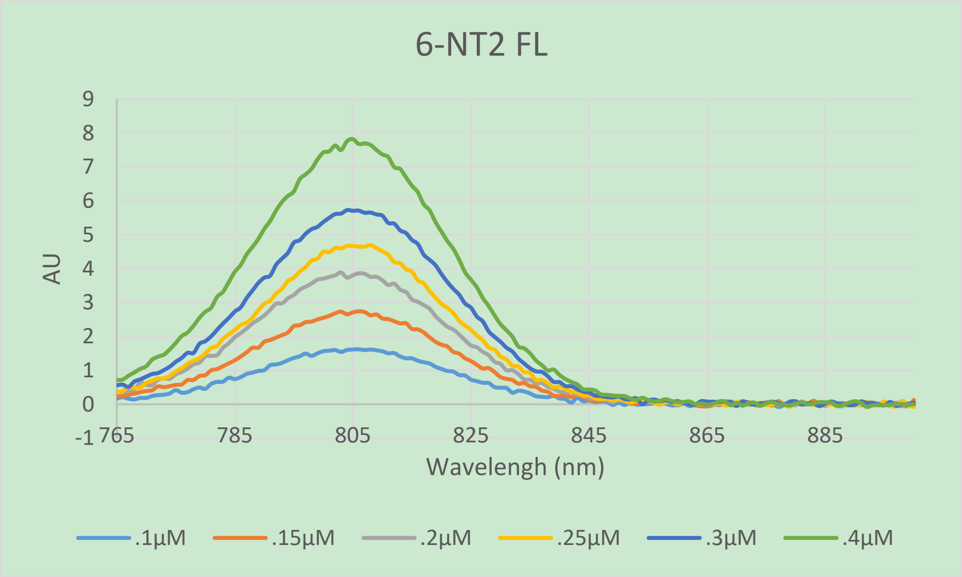
6-NT2 FL
AU
9
8
7
6
5
4
3
2
1
0
-1
765
785
805
825
845
865
885
Wavelengh (nm)
.1µM
.15µM
.2µM
.25µM
.3µM
.4µM

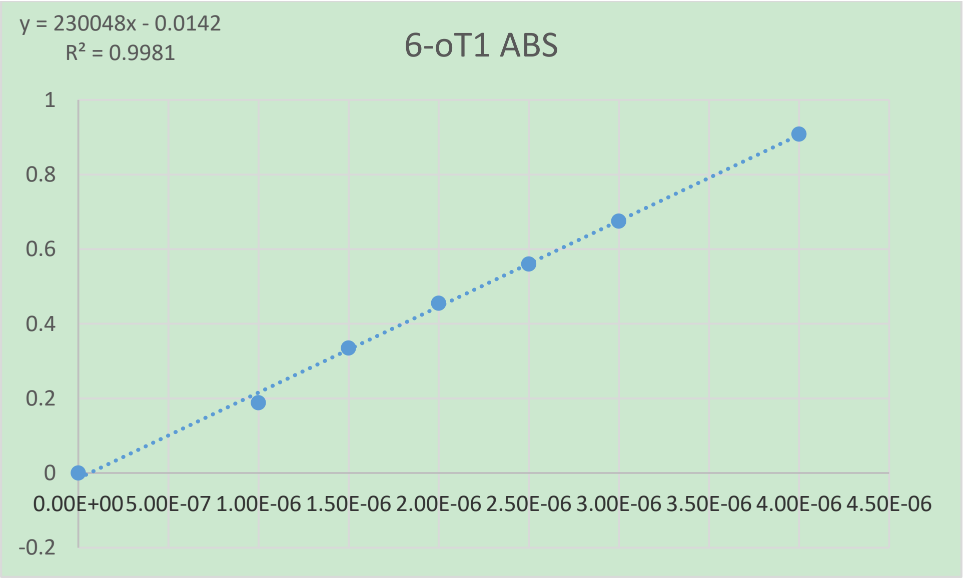
y = 230048x - 0.0142
R² = 0.9981
6-oT1 ABS
1
0.8
0.6
0.4
0.2
0
-0.2
0.00E+00 5.00E-07 1.00E-06 1.50E-06 2.00E-06 2.50E-06 3.00E-06 3.50E-06 4.00E-06 4.50E-06

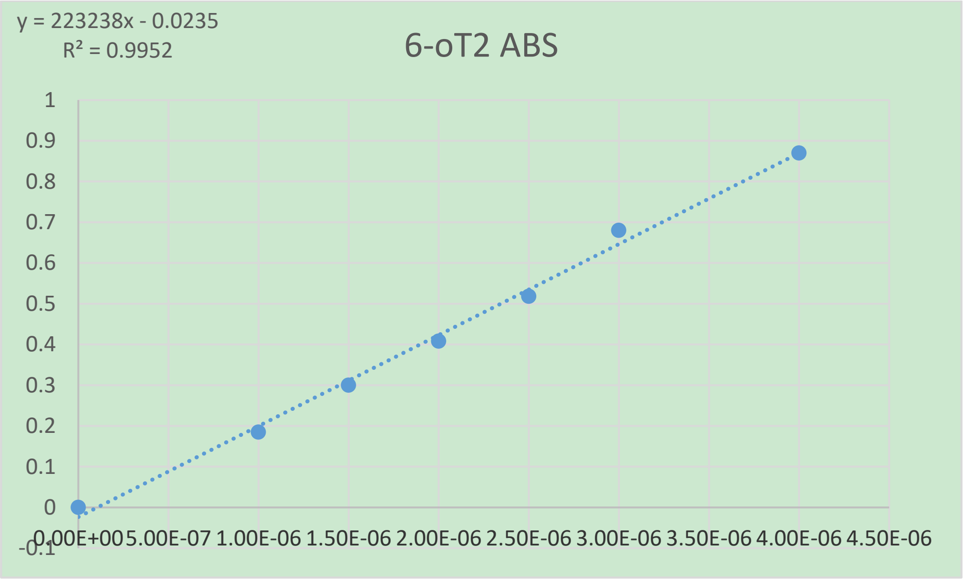
y = 223238x - 0.0235
R² = 0.9952
6-oT2 ABS
1
0.9
0.8
0.7
0.6
0.5
0.4
0.3
0.2
0.1
0
-0.1
0.00E+00 5.00E-07 1.00E-06 1.50E-06 2.00E-06 2.50E-06 3.00E-06 3.50E-06 4.00E-06 4.50E-06

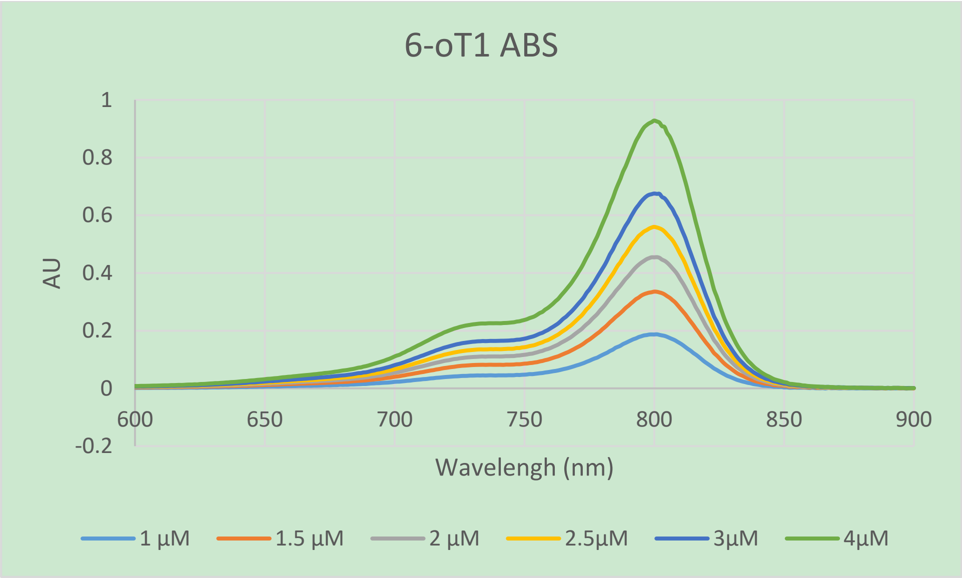
6-oT1 ABS
AU
1
0.8
0.6
0.4
0.2
0
-0.2
600
650
700
750
800
850
900
Wavelengh (nm)
1 µM
1.5 µM
2 µM
2.5µM
3µM
4µM

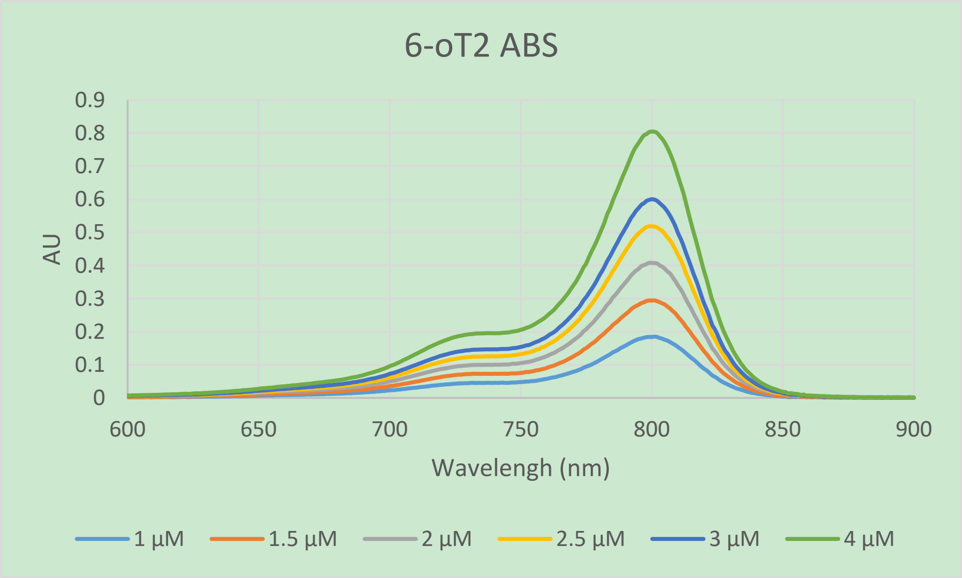
6-oT2 ABS
AU
0.9
0.8
0.7
0.6
0.5
0.4
0.3
0.2
0.1
0
600
650
700
750
800
850
900
Wavelengh (nm)
1 µM
1.5 µM
2 µM
2.5 µM
3 µM
4 µM

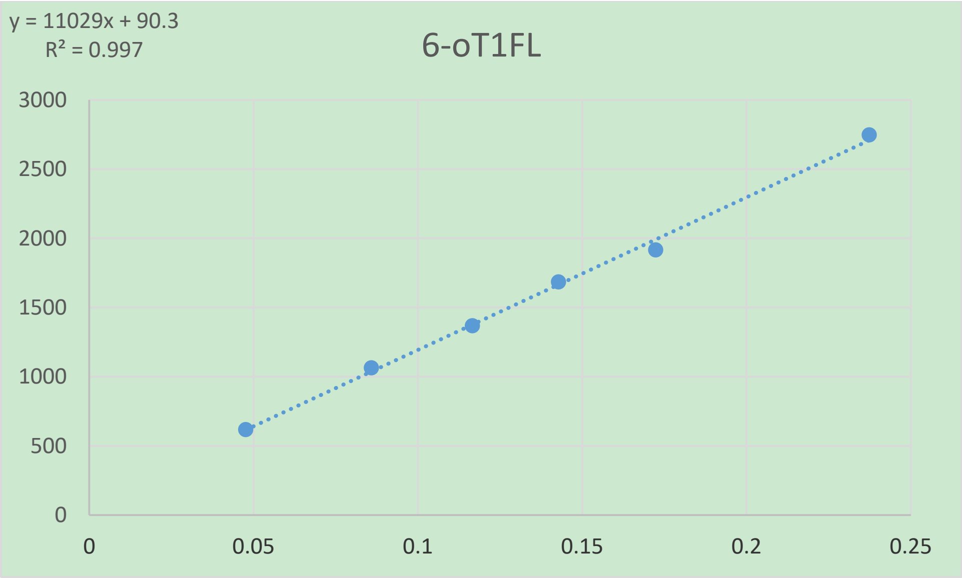
y = 11029x + 90.3
R² = 0.997
6-oT1FL
3000
2500
2000
1500
1000
500
0
0
0.05
0.1
0.15
0.2
0.25

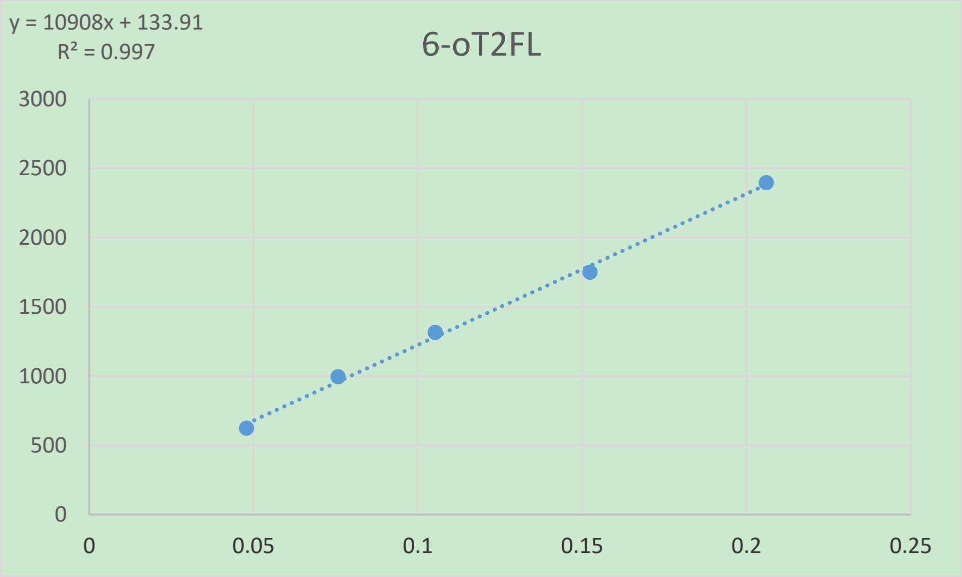
y = 10908x + 133.91
R² = 0.997
6-oT2FL
3000
2500
2000
1500
1000
500
0
0
0.05
0.1
0.15
0.2
0.25

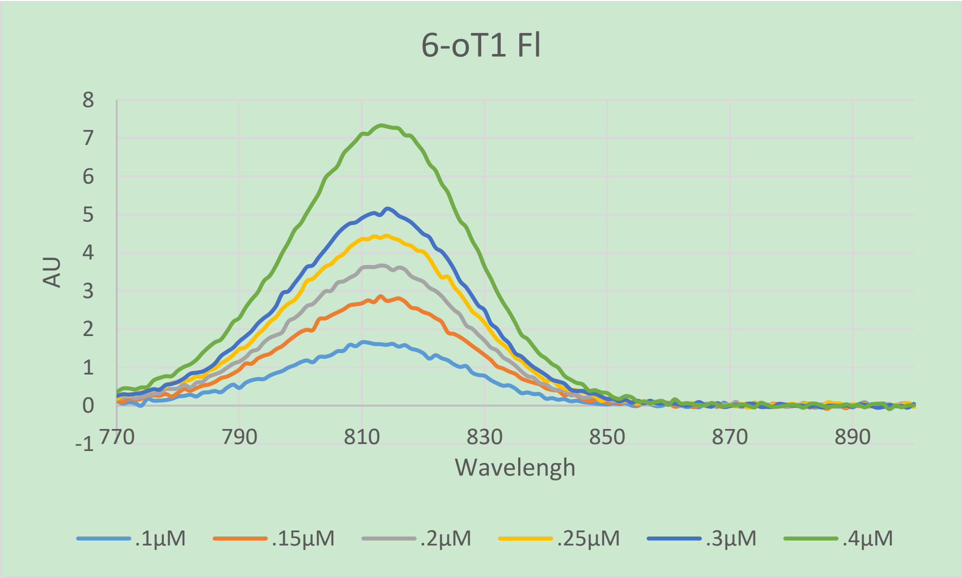
6-oT1 Fl
AU
8
7
6
5
4
3
2
1
0
-1
770
790
810
830
850
870
890
Wavelengh
.1µM
.15µM
.2µM
.25µM
.3µM
.4µM

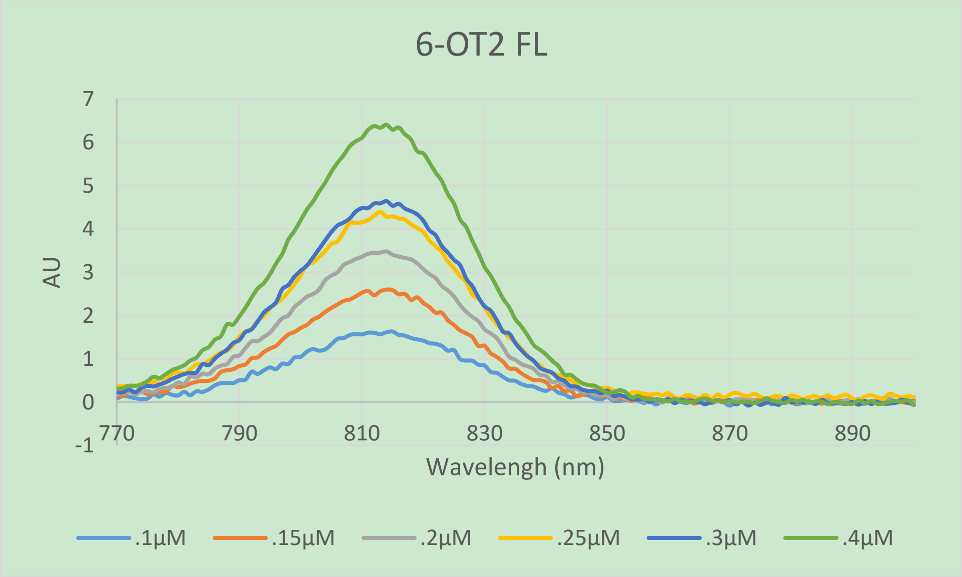
6-OT2 FL
AU
7
6
5
4
3
2
1
0
-1
770
790
810
830
850
870
890
Wavelengh (nm)
.1µM
.15µM
.2µM
.25µM
.3µM
.4µM

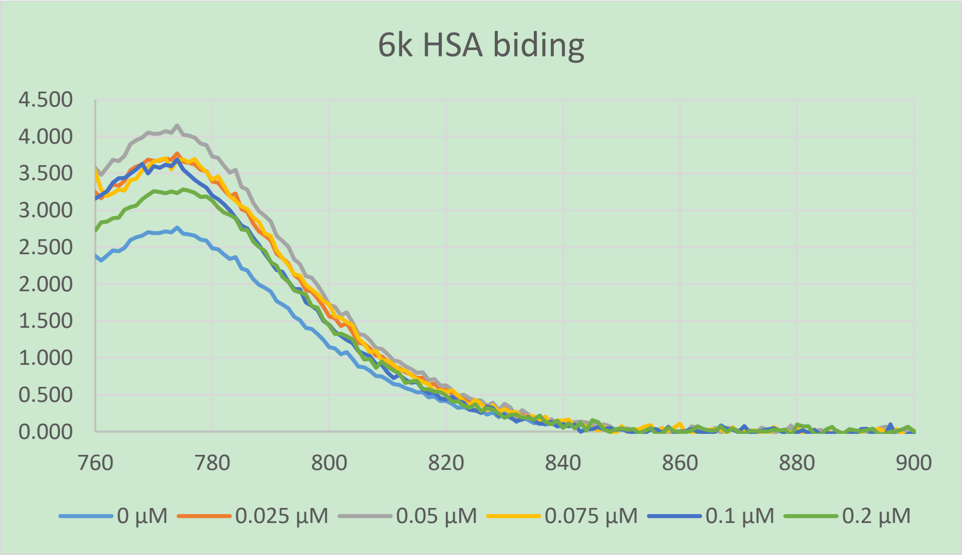
6k HSA biding
4.500
4.000
3.500
3.000
2.500
2.000
1.500
1.000
0.500
0.000
760
780
800
820
840
860
880
900
0 µM
0.025 µM
0.05 µM
0.075 µM
0.1 µM
0.2 µM

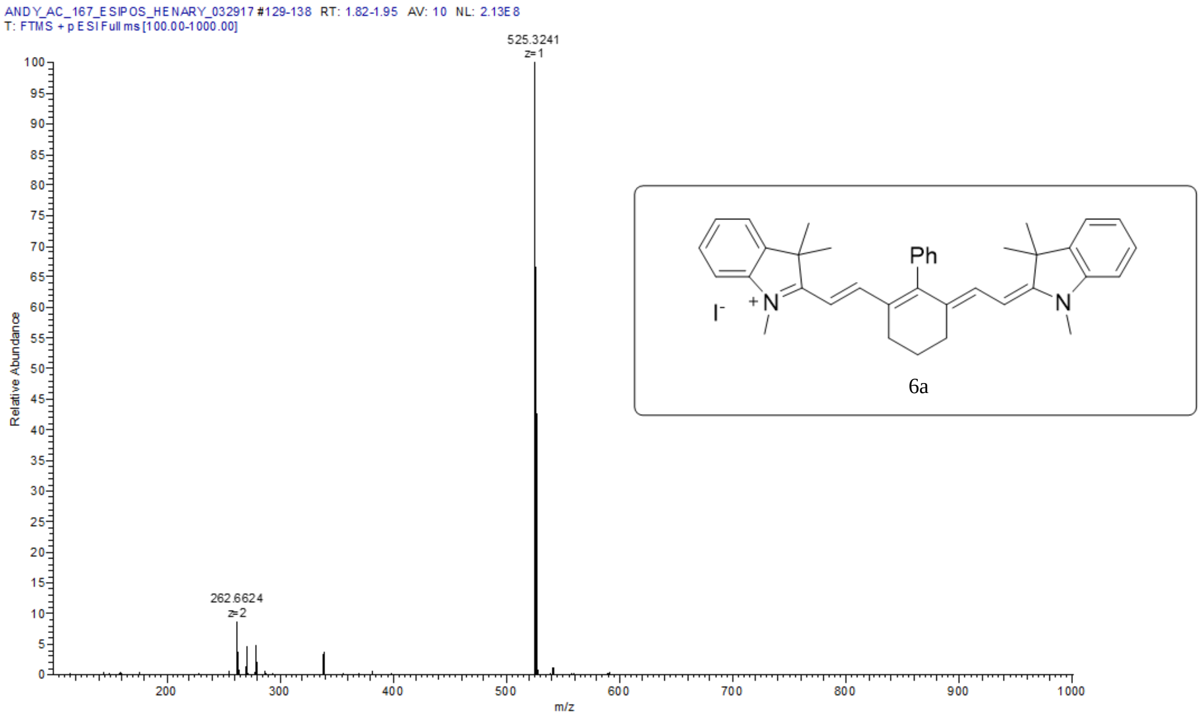
ANDY_AC_167_ESIPOS_HENARY_032917 #129-138 RT: 1.82-1.95 AV: 10 NL: 2.13E8
T: FTMS + p ESI Full ms [100.00-1000.00]
525.3241
z=1
262.6624
z=2
Relative Abundance
m/z
Ph
I⁻
+N
N
6a

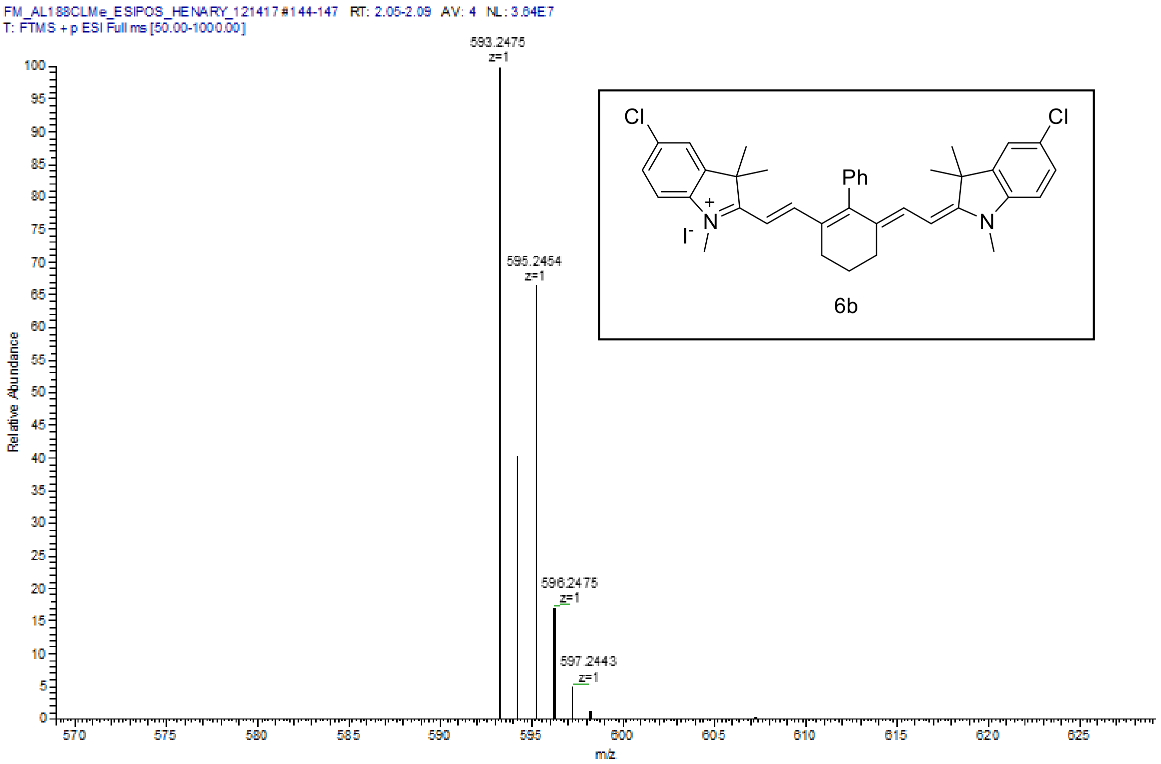
FM_AL188CLMe_ESIPOS_HENARY_121417 #144-147 RT: 2.05-2.09 AV: 4 NL: 3.84E7
T: FTMS + p ESI Full ms [50.00-1000.00]
593.2475
z=1
595.2454
z=1
596.2475
z=1
597.2443
z=1
Relative Abundance
m/z
Cl
Cl
Ph
N
N
I-
6b

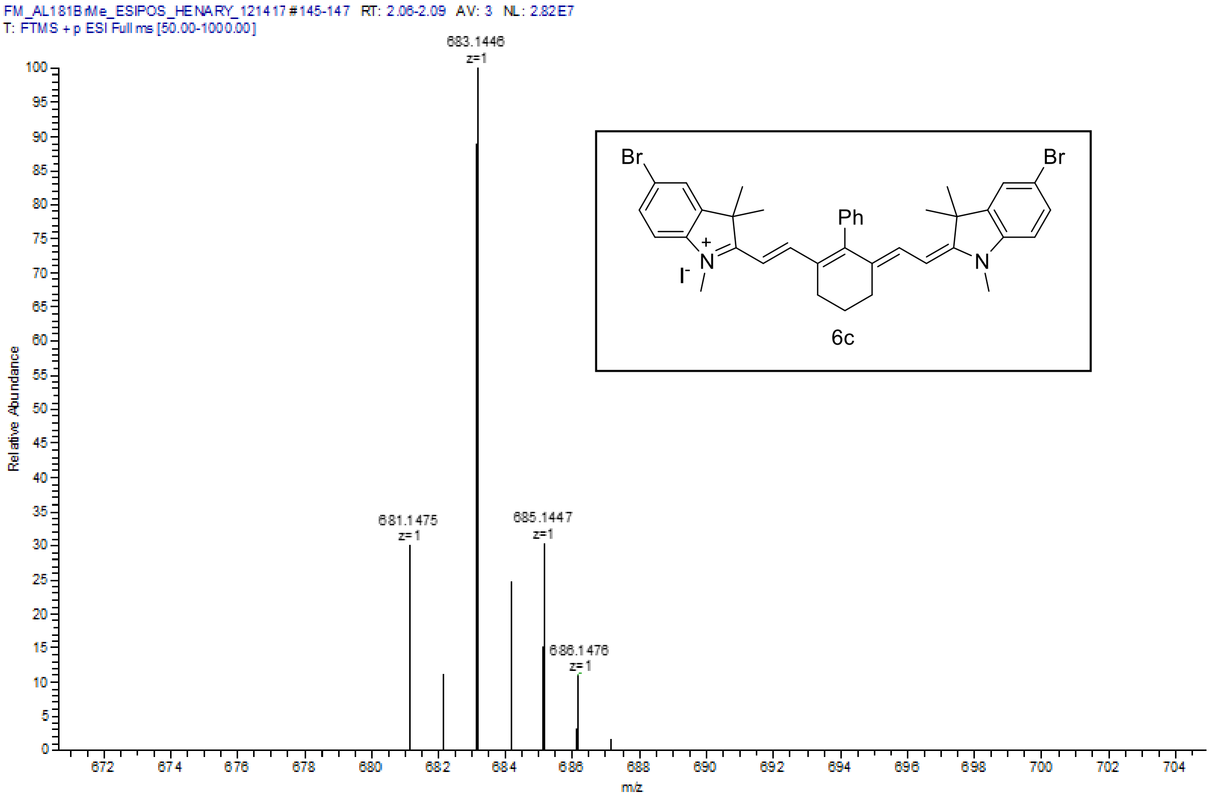
FM_AL181BrMe_ESIPOS_HENARY_121417 #145-147 RT: 2.06-2.09 AV: 3 NL: 2.82E7
T: FTMS + p ESI Full ms [50.00-1000.00]
683.1446
z=1
681.1475
z=1
685.1447
z=1
686.1476
z=1
Relative Abundance
m/z
Br
Br
Ph
N
N
I-
6c

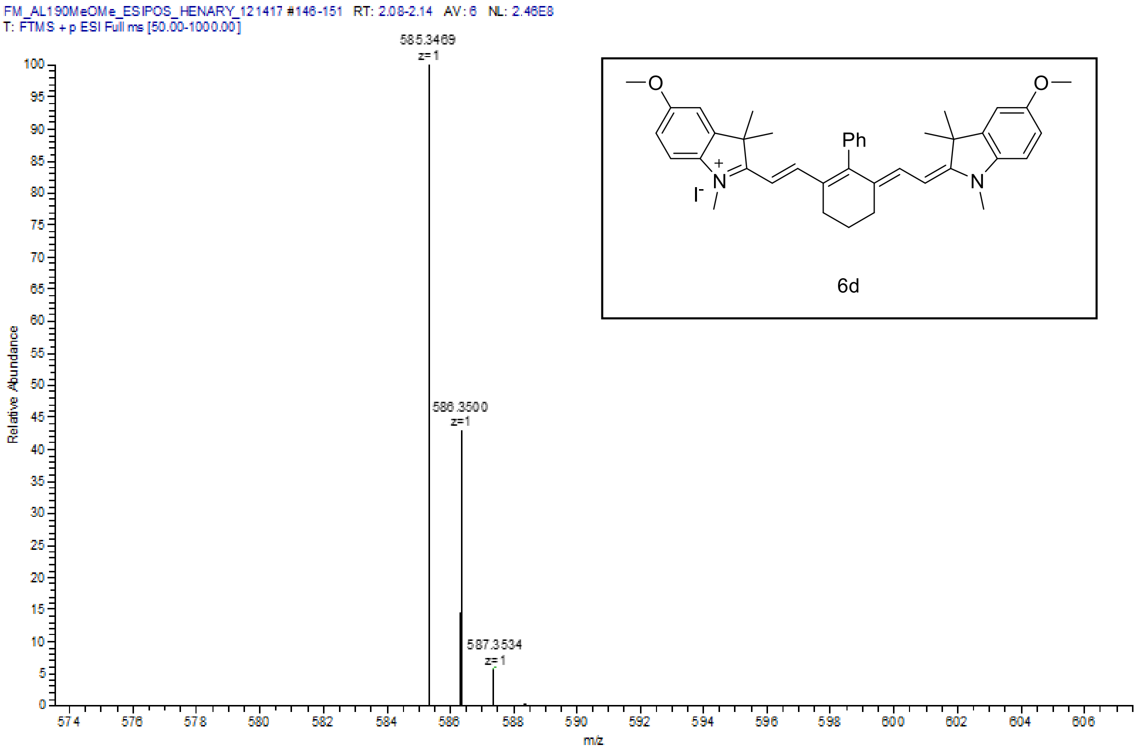
FM_AL190MeOMe_ESIPOS_HENARY_121417 #146-151 RT: 2.08-2.14 AV: 6 NL: 2.46E8
T: FTMS + p ESI Full ms [50.00-1000.00]
585.3469
z=1
586.3500
z=1
587.3534
z=1
Relative Abundance
m/z
Ph
I-
N+
N
6d

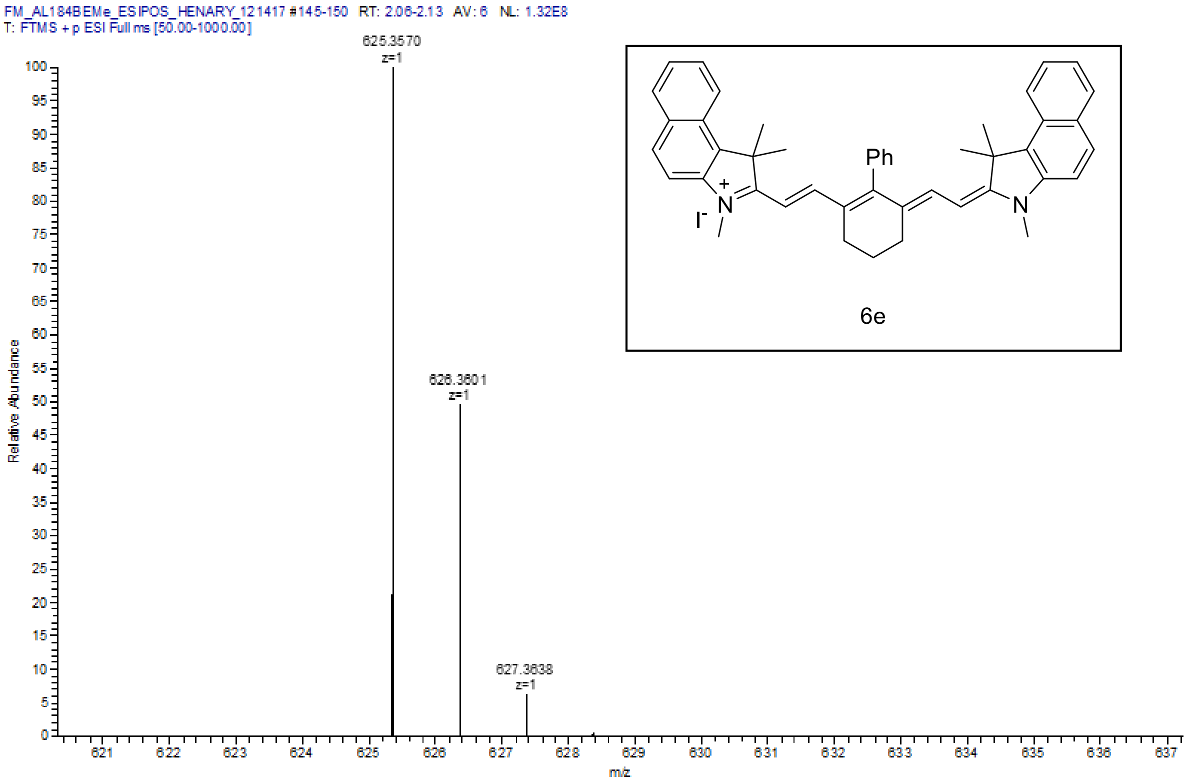
FM_AL184BEMe_ESIPOS_HENARY_121417 #145-150 RT: 2.06-2.13 AV: 6 NL: 1.32E8
T: FTMS + p ESI Full ms [50.00-1000.00]
625.3570
z=1
626.3601
z=1
627.3638
z=1
Relative Abundance
m/z
Ph
I-
N+
N
6e

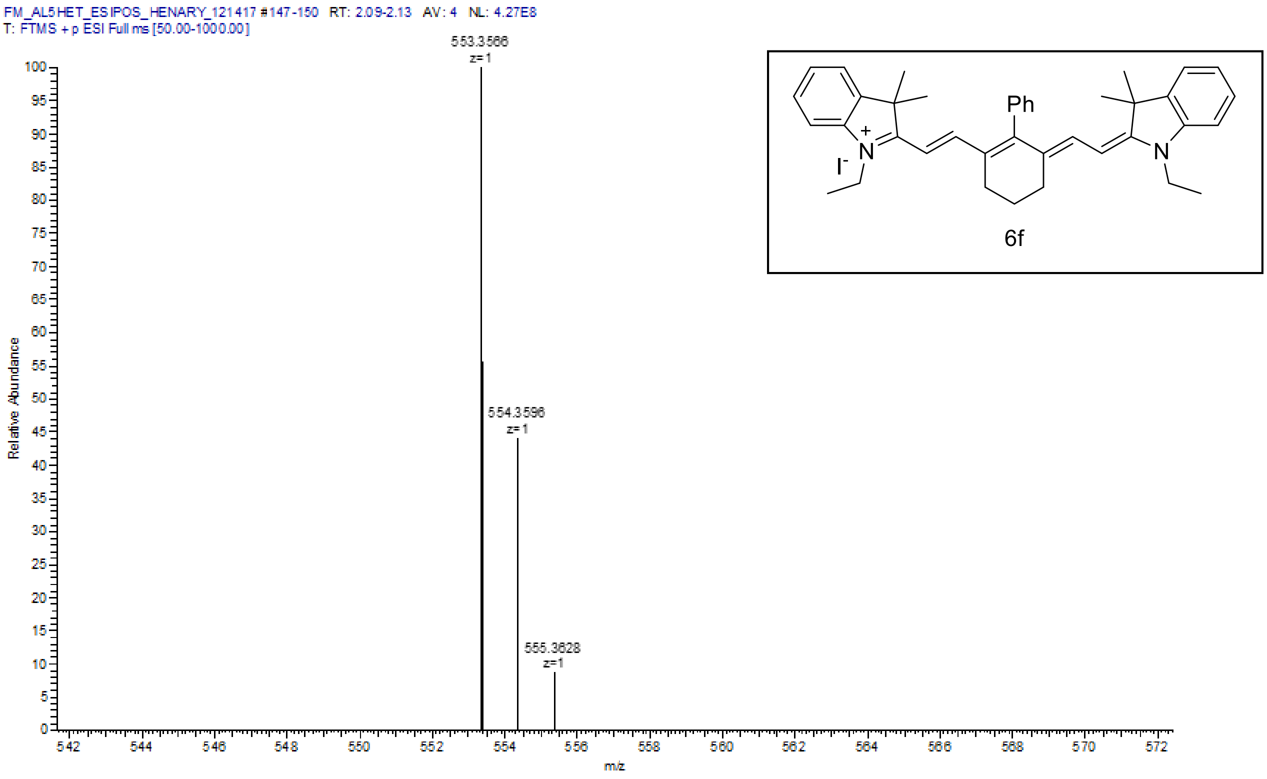
FM_AL5HET_ESIPOS_HENARY_121417 #147-150 RT: 2.09-2.13 AV: 4 NL: 4.27E8
T: FTMS + p ESI Full ms [50.00-1000.00]
553.3566
z=1
554.3596
z=1
555.3628
z=1
Relative Abundance
m/z
Ph
N
N
I-

6f

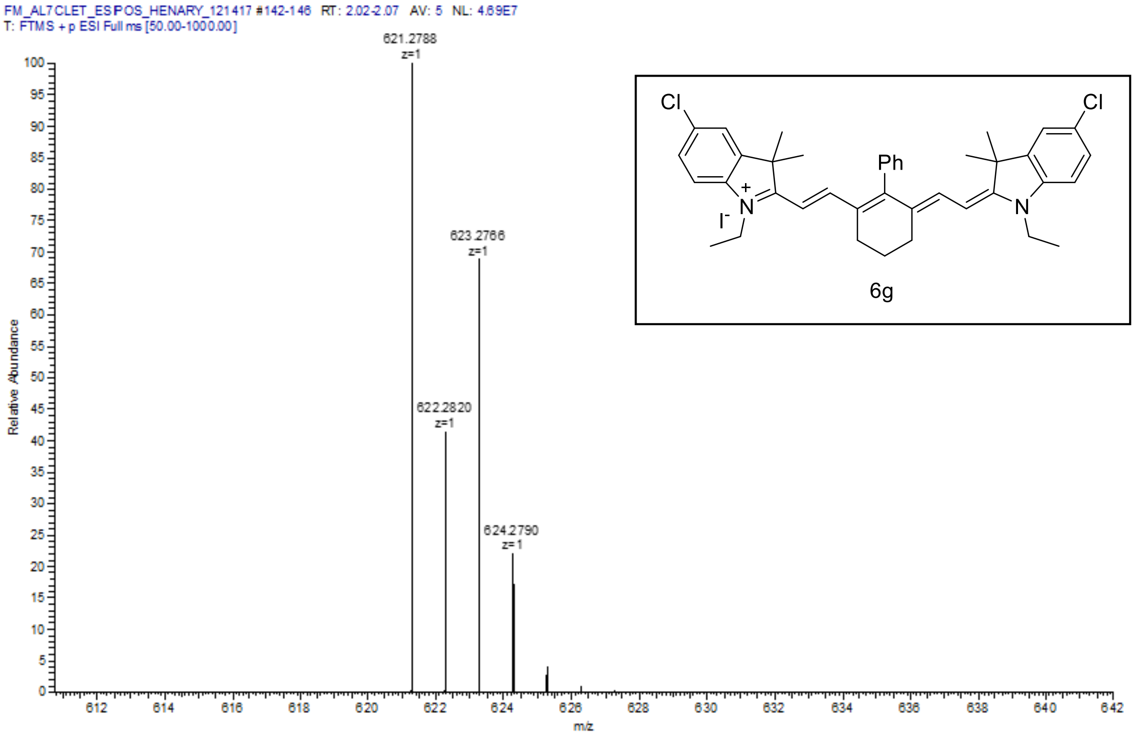
FM_AL7CLET_ESIPOS_HENARY_121417 #142-146 RT: 2.02-2.07 AV: 5 NL: 4.69E7
T: FTMS + p ESI Full ms [50.00-1000.00]
621.2788
z=1
622.2820
z=1
623.2766
z=1
624.2790
z=1
Relative Abundance
m/z
Cl
Cl
Ph
N
N
I-

6g

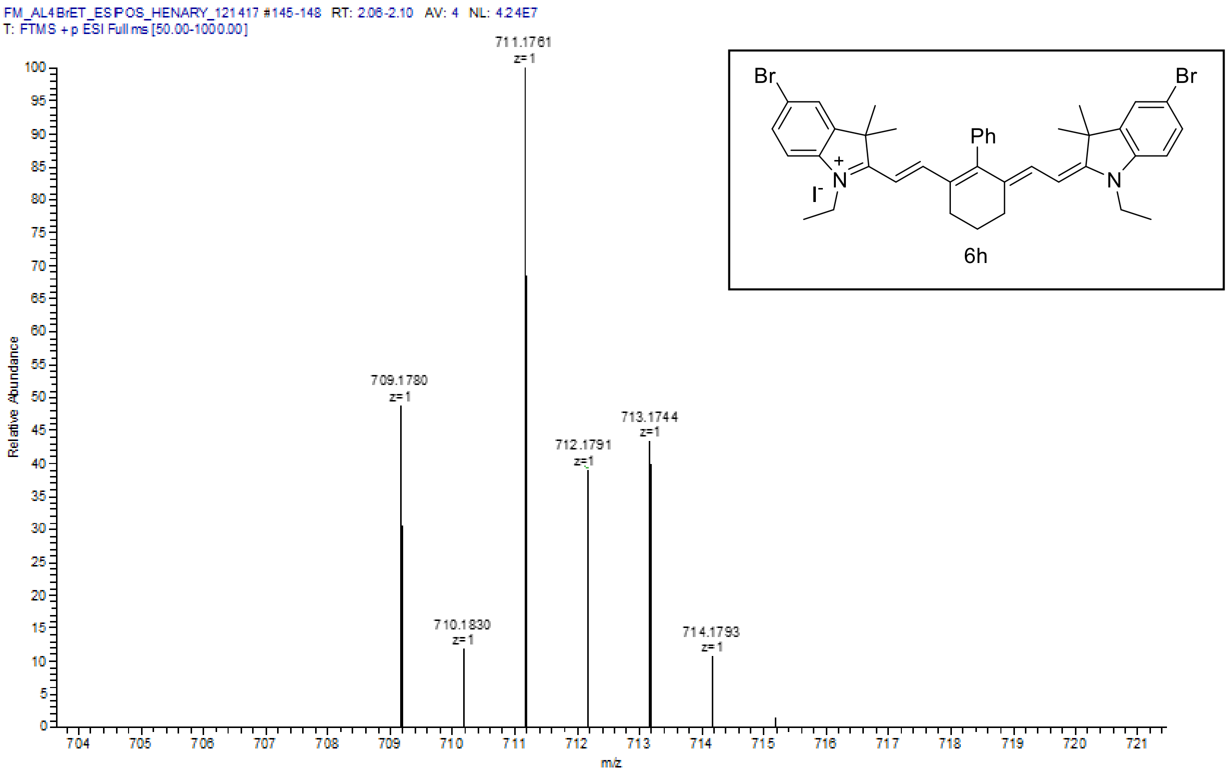
FM_AL4BrET_ESIPOS_HENARY_121417 #145-148 RT: 2.06-2.10 AV: 4 NL: 4.24E7
T: FTMS + p ESI Full ms [50.00-1000.00]
711.1761 z=1
709.1780 z=1
713.1744 z=1
712.1791 z=1
710.1830 z=1
714.1793 z=1
Relative Abundance
m/z
Br
Br
Ph
N
N
I-
6h

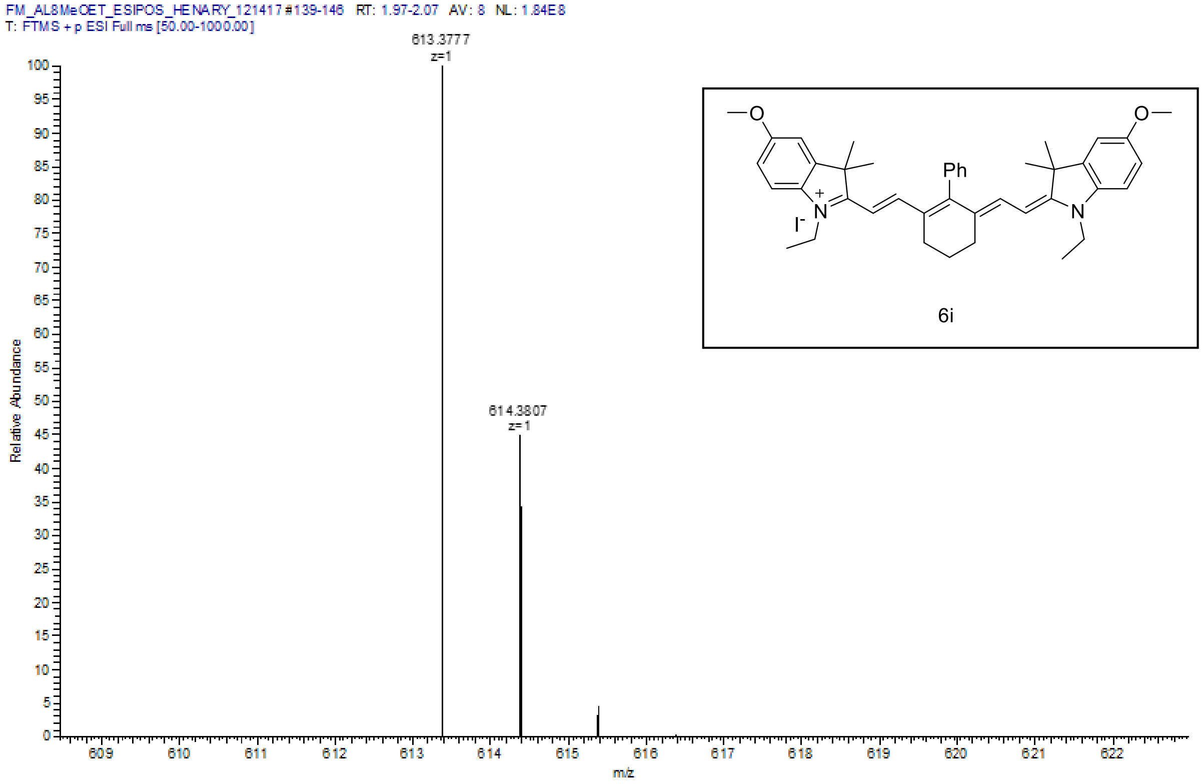
FM_AL8MeOET_ESIPOS_HENARY_121417 #139-146 RT: 1.97-2.07 AV: 8 NL: 1.84E8
T: FTMS + p ESI Full ms [50.00-1000.00]
613.3777 z=1
614.3807 z=1
Relative Abundance
m/z
O
O
Ph
N
N
I-
6i

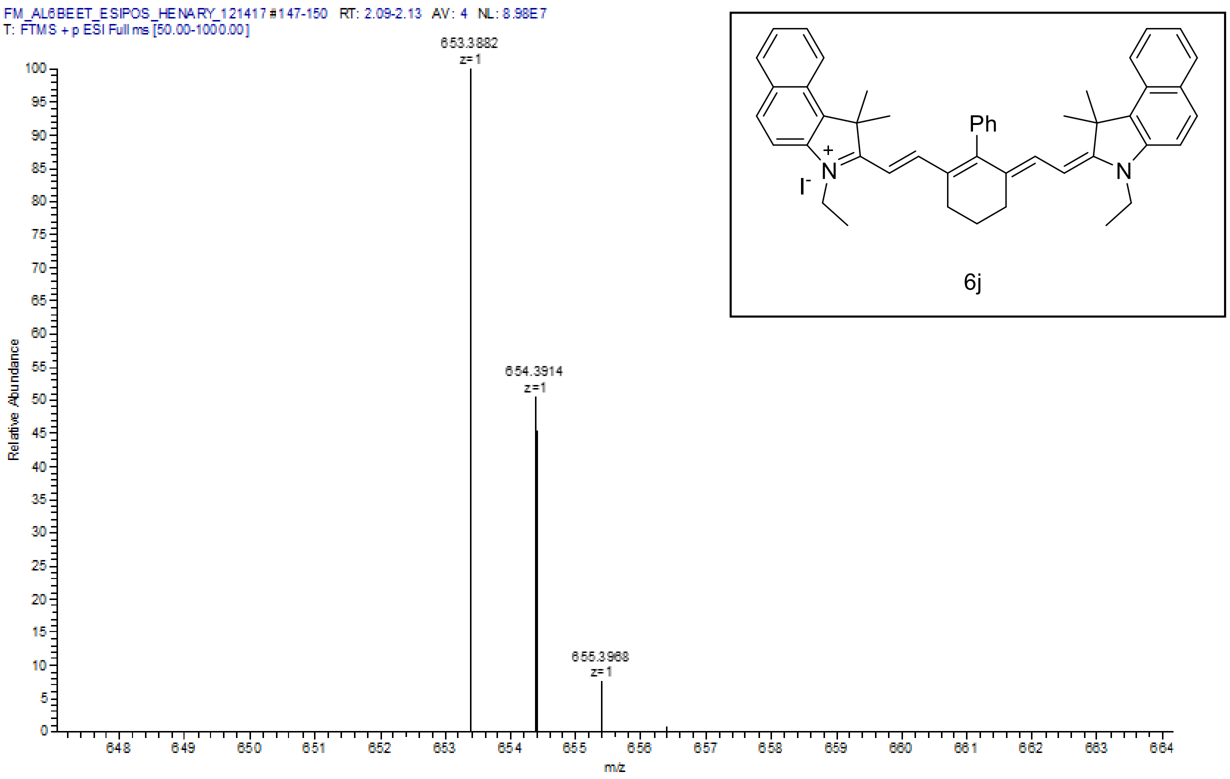
FM_AL6BEET_ESIPOS_HENARY_121417 #147-150 RT: 2.09-2.13 AV: 4 NL: 8.98E7
T: FTMS + p ESI Full ms [50.00-1000.00]
653.3882
z=1
654.3914
z=1
655.3968
z=1
Relative Abundance
m/z
Ph
N
I-
N
6j

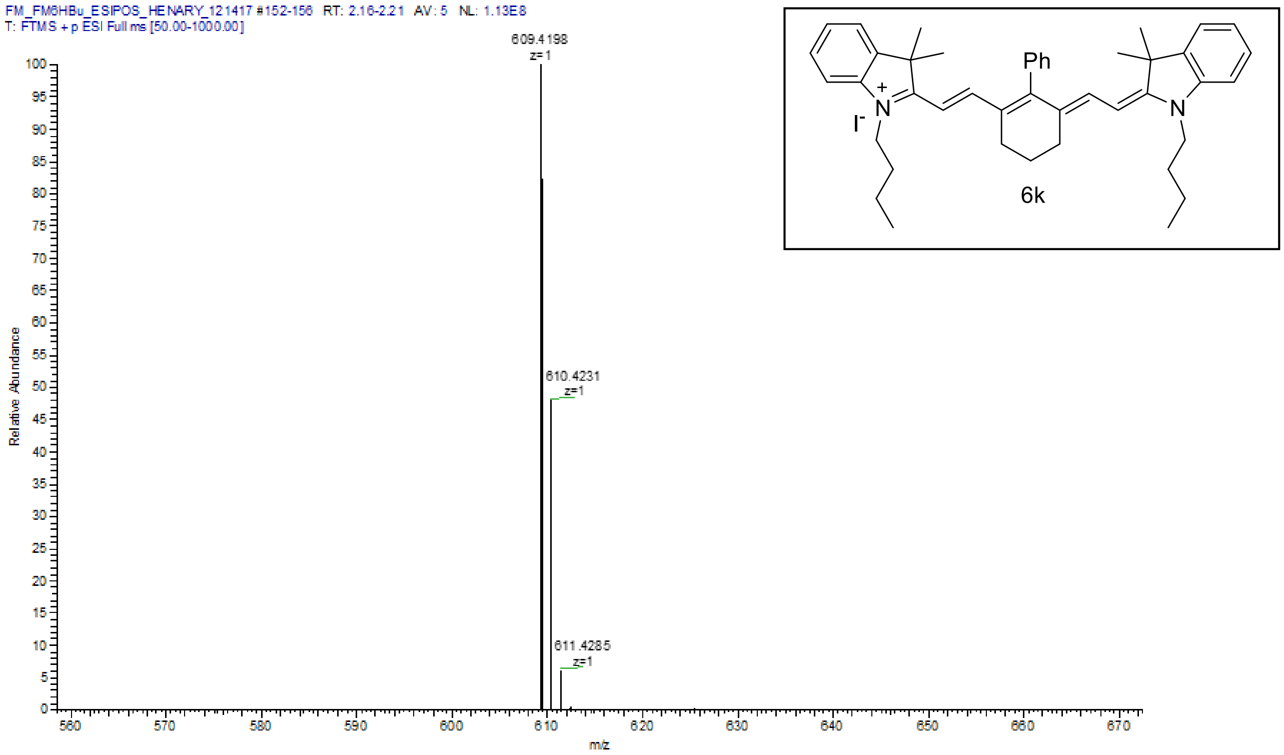
FM_FM6HBu_ESIPOS_HENARY_121417 #152-156 RT: 2.16-2.21 AV: 5 NL: 1.13E8
T: FTMS + p ESI Full ms [50.00-1000.00]
609.4198
z=1
610.4231
z=1
611.4285
z=1
Relative Abundance
m/z
Ph
N
I-
N
6k

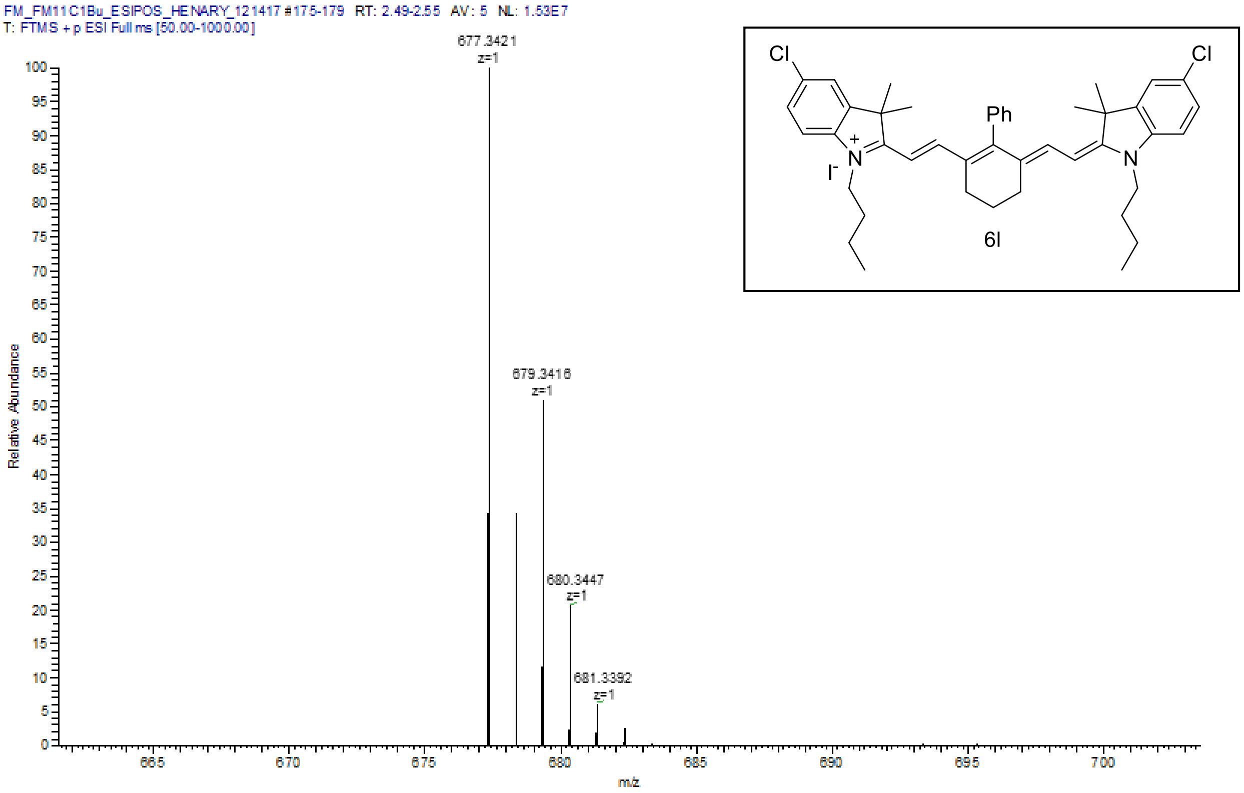
FM_FM11C1Bu_ESIPOS_HENARY_121417 #175-179 RT: 2.49-2.55 AV: 5 NL: 1.53E7
T: FTMS + p ESI Full ms [50.00-1000.00]
677.3421
z=1
679.3416
z=1
680.3447
z=1
681.3392
z=1
Relative Abundance
m/z
Cl
Cl
Ph
N
N
I-
6l

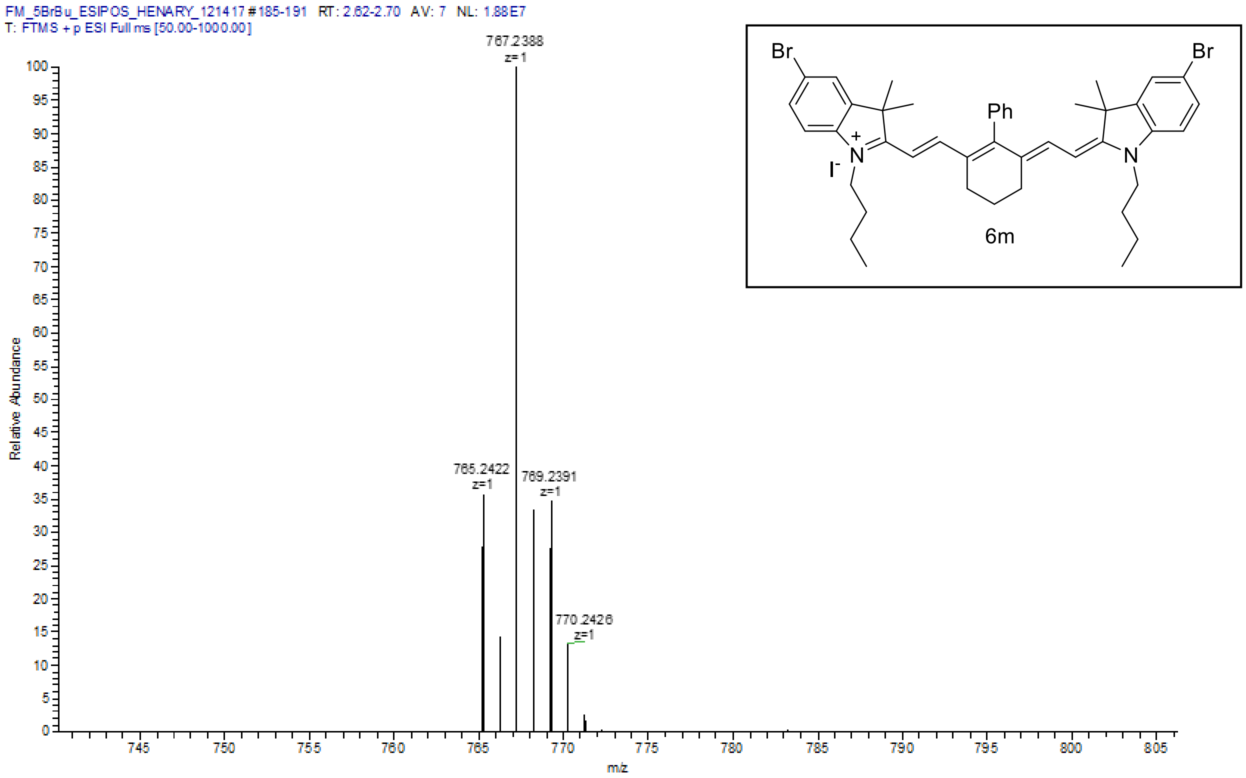
FM_5BrBu_ESIPOS_HENARY_121417 #185-191 RT: 2.62-2.70 AV: 7 NL: 1.88E7
T: FTMS + p ESI Full ms [50.00-1000.00]
767.2388
z=1
765.2422
z=1
769.2391
z=1
770.2426
z=1
Relative Abundance
m/z
Br
Br
Ph
N
N
I-
6m

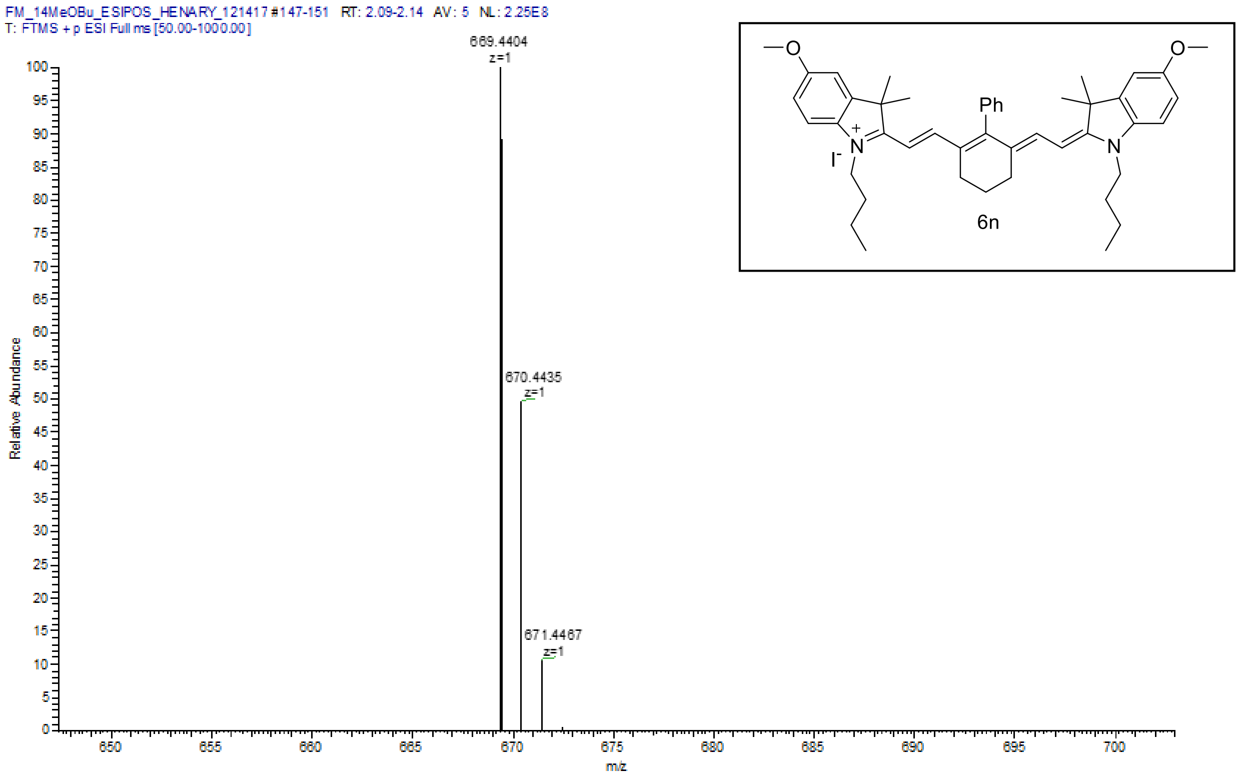
FM_14MeOBu_ESIPOS_HENARY_121417 #147-151 RT: 2.09-2.14 AV: 5 NL: 2.25E8
T: FTMS + p ESI Full ms [50.00-1000.00]
669.4404
z=1
670.4435
z=1
671.4467
z=1
Relative Abundance
m/z
Ph
I-
N+
N
6n

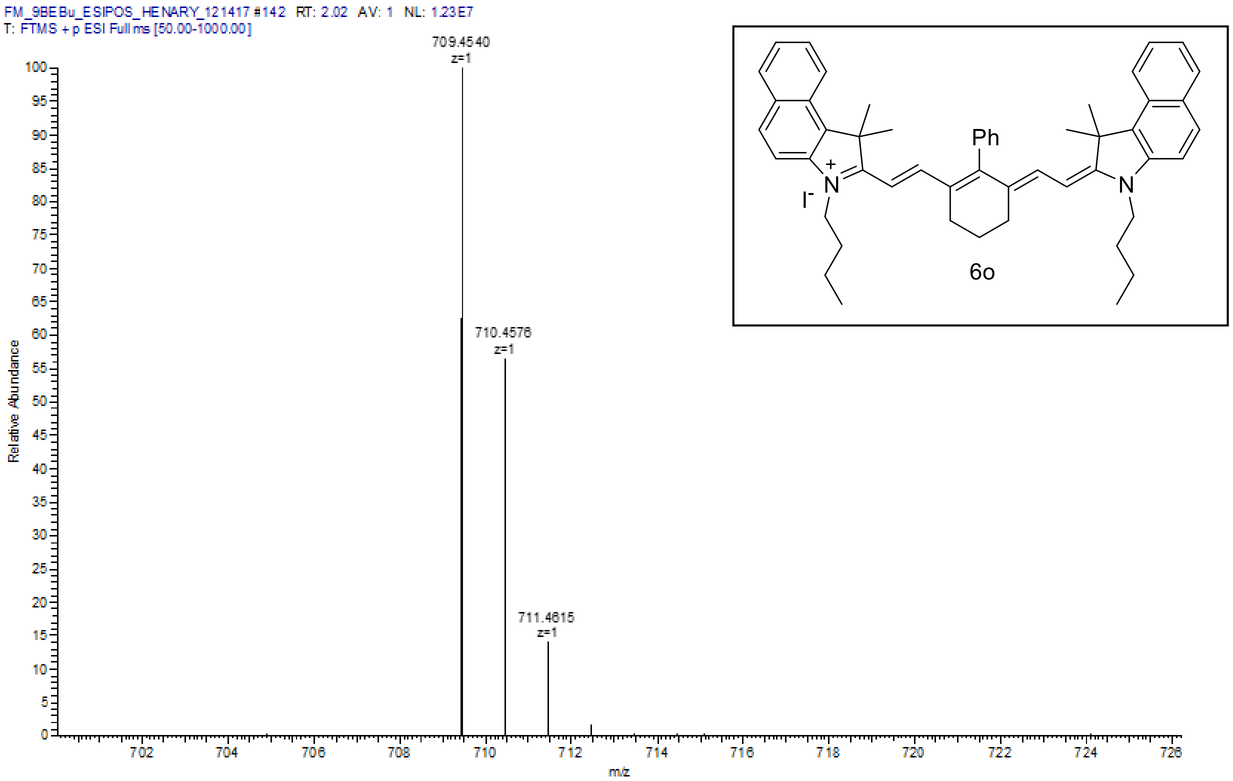
FM_9BEBu_ESIPOS_HENARY_121417 #142 RT: 2.02 AV: 1 NL: 1.23E7
T: FTMS + p ESI Full ms [50.00-1000.00]
709.4540
z=1
710.4576
z=1
711.4615
z=1
Relative Abundance
m/z
Ph
I-
N+
N
6o
